# Harnessing metabolomics and proteomics in a clinical trial for pulmonary arterial hypertension: insights from post-hoc analysis of the REHAB-PH trial

**DOI:** 10.1016/j.ebiom.2026.106191

**Published:** 2026-03-17

**Authors:** Hongyang Pi, Lu Xia, Samuel G. Rayner, Jeffrey L. Probstfield, Kelley R. Branch, Ali Shojaie, Peter J. Leary, Sina A. Gharib

**Affiliations:** a Division of Pulmonary, Critical Care and Sleep Medicine, University of Washington, Seattle, WA, USA; b Department of Statistics and Probability, Michigan State University, USA; c Division of Cardiology, University of Washington, Seattle, WA, USA; d Department of Statistics, University of Washington, USA; e Department of Biostatistics, University of Washington, USA; f Department of Epidemiology, University of Washington, USA

**Keywords:** Pulmonary arterial hypertension, Clinical trial, Longitudinal study, Proteomics, Metabolomics

## Abstract

**Background:**

The significant clinical and molecular heterogeneity of pulmonary arterial hypertension (PAH) poses challenges in identifying effective therapies. Advanced multidimensional profiling offers an opportunity to capture molecular responses and assess biomarker stability, yet its application in randomised trials remains limited.

**Methods:**

We evaluated the multi-omic profiles of participants with PAH in a randomised, placebo-controlled trial of famotidine. Plasma metabolomic and proteomic profiling was performed at enrolment and 24 weeks. Baseline profiles were compared between treatment arms to assess randomisation balance. Intraclass correlation coefficients quantified within-subject stability over time. Linear regression models adjusting for age, sex, body mass index and PAH aetiology evaluated famotidine's molecular effects. False discovery rate was controlled for multiple comparisons.

**Findings:**

For the 79 participants, baseline multi-omic profiles were similar between groups. At 24 weeks, 34 and 37 participants remained in the famotidine and placebo groups respectively. The placebo group showed high molecular stability, while greater variability was observed in the famotidine group. Famotidine treatment was associated with significant changes across 191 proteomic pathways (q-value <0.05), but no metabolomic changes remained significant after multiple-testing correction.

**Interpretation:**

Integrating multi-omics into a prospective clinical trial is feasible and yields stable longitudinal profiles in the absence of intervention. While famotidine did not yield clinical benefit, associated proteomic changes illustrate how molecular profiling can reveal treatment-related biology and inform future trial design. These findings highlight the broader utility of multi-omics for evaluating drug responses and identifying molecular endotypes in PAH and beyond.

**Funding:**

10.13039/100000002US National Institutes of Health.

**Research in context**

**Evidence before this study**

Pulmonary arterial hypertension (PAH) is marked by profound clinical and molecular heterogeneity, which complicates the development of effective therapies. Before conducting this work, we searched PubMed and Embase for studies published in English between July 1, 2019, and November 30, 2025, with the search terms “PAH” AND “trial” AND “omics”. We did not identify any published studies integrating multi-omics profiling into prospective randomised PAH trials. The only relevant reports were abstracts (American Journal of Respiratory and Critical Care Medicine 2024, European Respiratory Journal 2024, and Pneumologie 2025) describing proteomic changes associated with seralutinib in a Phase 2 trial. No studies addressed the longitudinal stability of metabolomic or proteomic profiles in PAH, nor evaluated the role of multi-omics for interpreting trial outcomes beyond direct drug effects.

**Added value of this study**

In this study, we integrated longitudinal metabolomics and proteomics within a prospective randomised PAH trial (REHAB-PH). We demonstrate the feasibility and utility of embedding multi-omics into trial design. We show that omic profiles remain remarkably stable in the absence of intervention, whereas treatment with famotidine, while showing no clinical difference compared with placebo, was associated with meaningful proteomic changes. These findings illustrate how molecular profiling can uncover treatment-related biology, provide mechanistic insights, and inform the inference on a trial.

**Implications of all the available evidence**

PAH continues to impose substantial morbidity and mortality, and the complexity of its molecular underpinnings continues to hinder therapeutic progress. Our study establishes a scalable framework for integrating advanced molecular profiling into randomised trials. By uncovering treatment-related biology even in the absence of clinical efficacy, this approach can help extract insights from otherwise “negative” trials, support drug repurposing, and enable the identification of molecular endotypes that stratify patients for future precision therapies.

## Introduction

Pulmonary arterial hypertension (PAH) is a progressive and life-threatening disease characterised by high morbidity and mortality.^1^ Despite available therapies, disease progression is common, and novel treatment approaches are needed to better understand disease biology and evaluate potential treatments. A major challenge in PAH research is the presence of significant clinical and molecular heterogeneity, which complicates the identification of robust biomarkers and the development of effective therapeutic interventions.^1^^,^^2^

Advanced omics profiling, including metabolomics and proteomics, offers a promising strategy to capture this complexity by providing high-dimensional molecular signatures of disease progression and treatment response.^3^ While there is an increasing literature focused on omics in PAH, most studies are cross-sectional and have been conducted in preclinical models or observational cohorts.^4^ The application of multi-omics in prospective, randomised clinical trials remains rare, and there is limited evidence on how such data can be integrated to inform trial interpretation, optimise study design, and identify candidate biomarkers for future research.

REHAB-PH (REpurposing a Histamine Antagonist to Benefit patients with Pulmonary Hypertension) was a single-centre, randomised, double-blind, placebo-controlled trial evaluating the effects of the histamine H_2_ receptor antagonist (H_2_RA) famotidine on PAH.^5^ Although the trial did not demonstrate clinical benefit with famotidine use, it incorporated longitudinal plasma metabolomics and proteomics at enrolment and study completion. This design provided a unique opportunity to assess both drug-related molecular changes and the stability of omics measures in the placebo group over time.

In this work, we use REHAB-PH as a model to explore the feasibility, analytical considerations, and interpretive value of integrating metabolomics and proteomics in a prospective clinical trial. Our aim is not only to characterise molecular effects of the H_2_RA intervention, but also to derive broader insights into how multi-omics can enhance trial interpretation, even in the absence of significant clinical changes. These findings have implications for the design and analysis of future trials in PAH and other complex diseases, particularly in leveraging omics-based biomarkers to bridge molecular and clinical endpoints.

## Methods

### Study design and participants

The REHAB-PH trial has been previously described and published.^5^ It was approved by the institutional review board at the University of Washington (Team A Identifier: 0005002). Briefly, the study enrolled adults aged 18 to 80 with PAH, all of whom had undergone a right heart catheterisation within the preceding five years to confirm their diagnosis. Demographic data including age, sex assigned at birth, height, and weight were recorded. PAH aetiology was determined through chart review and adjudicated by investigators. Aetiology was classified as idiopathic or into one of several associated PAH groups, including familial, connective tissue disease-associated, congenital heart disease-associated, portopulmonary, or methamphetamine-associated PAH.

Participants were randomised to receive either famotidine (20 mg daily) or placebo over the 24-week trial. Study visits took place at baseline and 24 weeks. Primary and secondary outcomes for this analysis include 6-min walk tests (6MWD), brain natriuretic peptide (BNP), transthoracic echocardiography (TTE), EmPHasis-10 (patient-reported outcome), and New York Heart Association (NYHA) Functional Class. Plasma samples were collected at both time points and stored for omics analysis.

### Multi-omic analyses

Sample preparation and measurements of metabolomics and proteomics were performed by Metabolon, Inc. (Durham, NC, USA) and SomaLogic, Inc. (7k platform, Boulder, CO, USA), respectively. Metabolites were measured as area under the peak curve, normalised to run-day medians, and log_2_-transformed. Missing metabolite data were imputed as previously described and included in the Supplement.^6^ Protein concentrations, measured in relative fluorescence units, were normalised to medians within three dilution level-based subgroups of aptamers, and log_2_-transformed. Aptamers annotated with more than one gene symbol were designated to be associated with the first gene symbol listed.^7^ Both omics datasets were pre-processed and normalised in the R statistical environment (R Core Team 2025).

To assess baseline differences in metabolomic and proteomic profiles between placebo and treatment arms, we first applied Uniform Manifold Approximation and Projection (UMAP) for dimensionality reduction, followed by calculation of silhouette width to quantify overall molecular similarity.^8^, ^9^, ^10^ In parallel, we performed Wilcoxon rank sum tests on individual features to identify any statistically significant differences between groups.

We then assessed intra-individual variability of plasma metabolome and proteome over six months. Intraclass correlation coefficients (ICC) were computed for each analyte using linear mixed effects models with random intercepts, adjusting for age, sex, body mass index (BMI) and PAH aetiology.^11^ ICCs were categorised as follows: <0.40 (low stability), 0.40–0.50 (moderate stability), 0.51–0.74 (moderately high stability), and ≥0.75 (high stability). ICCs were stratified by treatment arm to evaluate the global molecular effects of famotidine over time.

Treatment-related molecular changes were identified using paired analyses via linear regression with the same covariate adjustments. Pathway enrichment analysis was performed using Globaltest for metabolomics and Gene Set Enrichment Analysis (GSEA) for proteomics. For Globaltest, p-values were adjusted using the Benjamini-Hochberg procedure, with significance defined as adjusted p-value <0.05. For GSEA, significance was assessed using a permutation-based false discovery rate (FDR) control procedure with 1000 permutations, with an FDR q-value <0.05 considered significant.

Lastly, we used Weighted Gene Co-expression Network Analysis (WGCNA) to identify baseline molecular endotypes that were associated with responsiveness to famotidine treatment. We constructed baseline metabolite and protein modules and examined their correlations with clinical improvement by different traits (BNP level, 6MWD, RV structure and function on TTE, EmPHasis-10 score and NYHA functional class) over time in both treatment arms. By comparing these correlations between the famotidine and placebo groups, we aimed to generate hypotheses about whether patients with specific molecular endotypes might be more likely to experience benefit or harm with famotidine relative to the overall study population.

Detailed methods, including regression models, are included in the Supplement.

### Role of the funding source

The funders of this study had no role in study design, data collection, analysis, data interpretation, or writing of the report.

## Results

### Cohort characteristics

Between May 2019 and July 2023, 79 participants received study drug (40 received famotidine and 39 received placebo) and underwent baseline plasma collection for multi-omics analysis. Table 1 provides an overview of the cohort characteristics in REHAB-PH. A total of 72 participants completed multi-omics assessments at study completion. One participant's proteomics data was excluded due to measurement error at follow-up, yielding a cohort of 71 (34 famotidine arm and 37 placebo arm) participants with paired samples for use in linear regression and longitudinal analyses.

**Table 1:** Baseline clinical characteristics.

	Famotidine (n = 40) N (%) or mean (SD)	Placebo (n = 39) N (%) or mean (SD)
Age, years	49.9 (13.7)	51.4 (12.7)
Female	32 (80%)	31 (79.5%)
White	37 (92.5%)	35 (89.8%)
Body mass index, kg/m^2^	29.2 (6.2)	31.2 (7.9)
Time since diagnosis, years	4.3 (3.8)	4.2 (3.9)
PAH Aetiology		
Idiopathic	22 (55%)	15 (38.5%)
Familial	0 (0%)	4 (10.3%)
Methamphetamine-associated	10 (25%)	11 (28.2%)
Connective tissue disease-associated	6 (15%)	5 (12.8%)
Congenital heart disease-associated	0 (0%)	3 (7.7%)
Porto-pulmonary hypertension	2 (5%)	1 (2.6%)
Severity at baseline		
NYHA functional Class III/IV	16 (40%)	15 (38.4%)
B-type natriuretic peptide, pg/mL	108 (111)	111 (186)
6-Minute walk distance, m	414 (91)	407 (107)
RV basal diameter (echocardiogram), mm	46 (9)	45 (7)
RV TAPSE (echocardiogram), mm	21 (4)	20 (5)
emPHasis-10 HRQoL, score	19 (12)	18 (9)
Treatments at baseline		
Phosphodiesterate-5 Inhibitors	34 (85%)	38 (97.4%)
Endothelin-receptor antagonists	35 (87.5%)	36 (92.3%)
Prostacyclin-targeted therapy	22 (55%)	22 (56.4%)

Abbreviations: HRQoL, Health-Related Quality of Life; kg, kilogrammes; m, metres; mm, millimetres; NYHA, New York Heart Association; RV, right ventricular; SD, standard deviation; TAPSE, tricuspid annular plane systolic excursion. Percentages may not sum to exactly 100% due to rounding.

### Randomisation balances baseline omic profiles

**Fig. 1:**
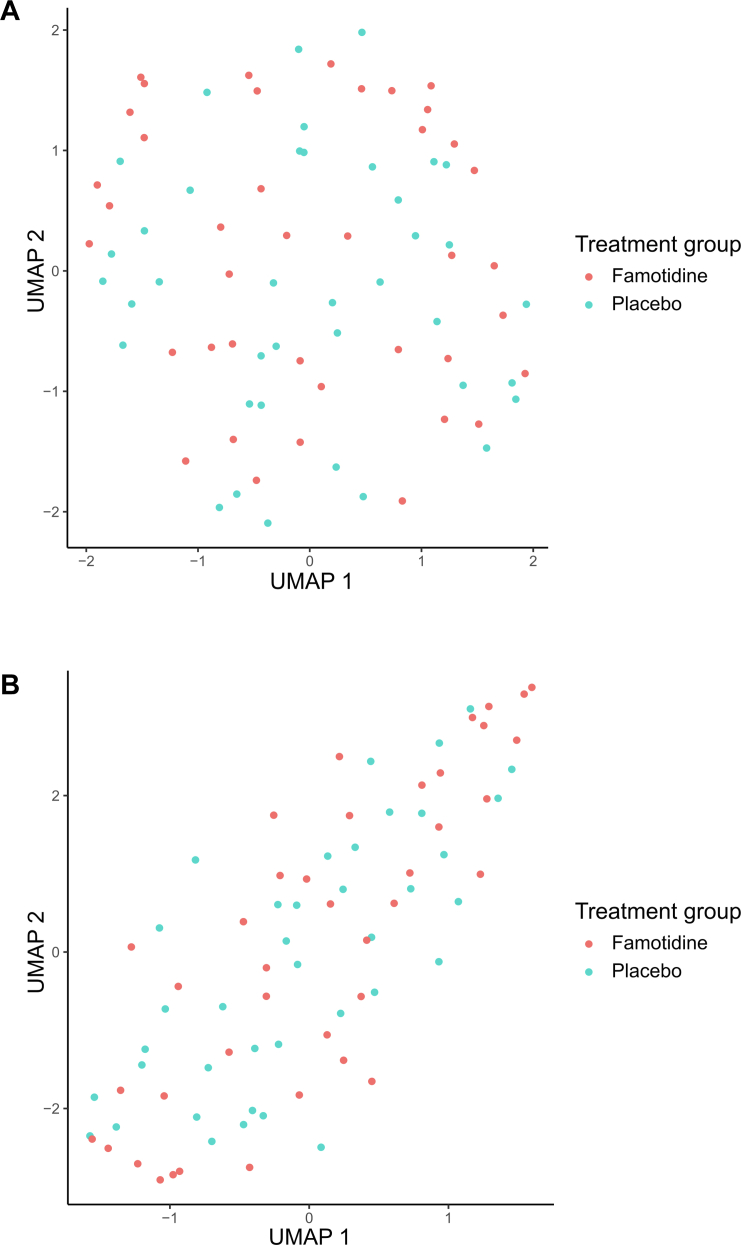
**Uniform Manifold Approximation and Projection visualisation of baseline metabolomic (A) and proteomic (B) profiles between treatment arms.** This analysis assesses whether the famotidine and placebo arms (n = 40 versus 39) were distinct at baseline and evaluates the effectiveness of randomisation at the molecular level. Mean silhouette widths were 0.007 (A) and −0.0005 (B). Overall, the baseline metabolome and proteome do not distinguish between the treatment and placebo arms.

Baseline metabolomic and proteomic analyses of the 79 participants revealed no significant molecular differences between the treatment and placebo groups, either at the individual metabolite/protein level or across broader pathways. UMAP visualisations showed substantial overlap between the two groups in both the metabolomic and proteomic domains, with near-zero mean silhouette widths (0.007 for metabolomics and −0.0005 for proteomics), indicating strong baseline molecular similarity following randomisation (Fig. 1).

### Temporal stability of plasma metabolites and proteins

**Fig. 2:**
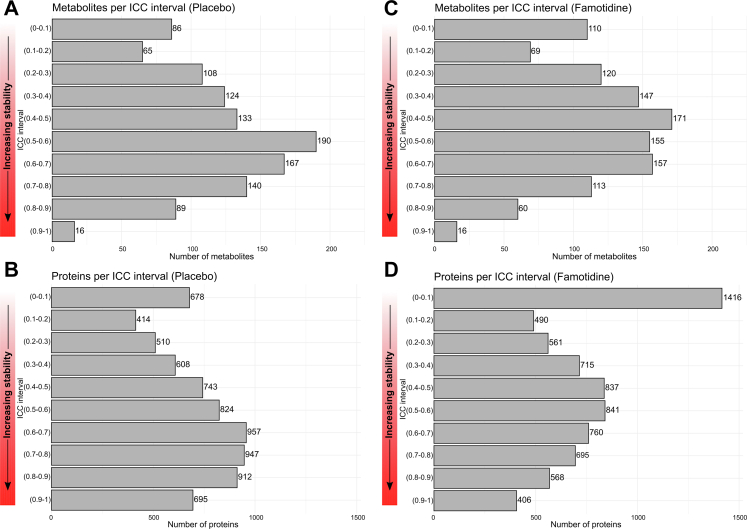
**Distribution of intraclass correlation coefficients (ICC) by treatment arms over 24 weeks.** Panel A. Histogram of ICCs for 1118 plasma metabolites in the placebo arm (n = 37); Panel B. Histogram of ICCs for 7289 plasma proteins in the placebo arm; Panel C. Histogram of ICCs for 1118 plasma metabolites in the famotidine arm (n = 34); Panel D. Histogram of ICCs for 7289 plasma proteins in the famotidine arm. Note that the metabolome and proteome display temporal stability over six months in the placebo group but there is substantial proteomic variability in the famotidine arm.

To better understand the temporal changes in the metabolome and proteome, we examined molecular stability in the placebo arm of the trial. In the absence of intervention, 65.7% of metabolites and 69.7% of proteins showed moderate or high stability over 24 weeks, defined by an ICC above 0.40. Only a small minority of metabolites (7.7%) and proteins (9.3%) had high levels of variability with ICC's < 0.10 (Fig. 2A and B, Supplemental Tables S1 and S2). Famotidine treatment did not appear to globally alter the metabolome: 60.1% of metabolites had moderate ICCs, and only 9.8% showed high levels of variability (Fig. 2C–Supplemental Table S1). In contrast, proteomic stability was more affected by famotidine treatment, with a lower proportion of proteins showing moderate or greater ICCs (56.3%) and a larger proportion exhibiting high variability (19.4%) (Fig. 2D, Supplemental Table S2).

### Treatment associated molecular changes

**Fig. 3:**
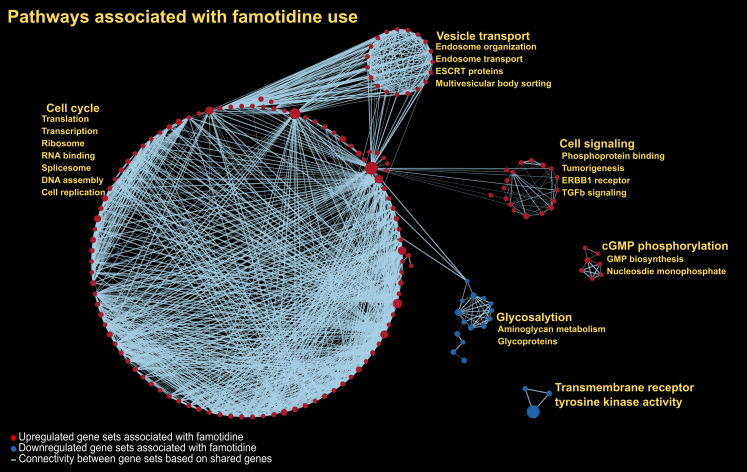
**Network-based depiction of gene set enrichment analysis for famotidine's effects on protein changes over six months in participants with pulmonary arterial hypertension.** Each red sphere corresponds to an upregulated gene set and blue sphere corresponds to a downregulated gene set. Connectivity between the gene sets is based on 50% or greater overlap among their member proteins. Note that the topology of the network is characterised by the emergence of biological modules comprised of highly interconnected pathways with similar functional themes. Representative pathways for each of these modules are highlighted.

For the paired analyses on the 71 participants with complete data, at the analyte level, famotidine treatment was not associated with significant changes in individual metabolite or protein abundances after adjusting for age, sex, BMI and PAH aetiology and controlling for multiple comparisons. There were no metabolomic pathways that were significantly changed in response to famotidine treatment after multiple testing adjustment. In contrast, proteomic-based pathway analysis indicated that famotidine use significantly altered 191 pathways with 171 upregulated and 20 downregulated (q-value <0.05). The upregulated pathways mapped to larger modules involved in cell cycle, cellular signalling (e.g., TGF-β), and vesicle transport, whereas processes involving glycosylation and transmembrane receptor tyrosine kinase (RTK) activity were significantly downregulated (Fig. 3 and Supplemental Table S3). The within-subject expression changes for the top leading-edge RTK proteins across treatment groups were examined and compared to assess whether protein-level trends were consistent with observed pathway enrichment (Supplemental Figure S1).

### Endotype-specific treatment responses

**Fig. 4:**
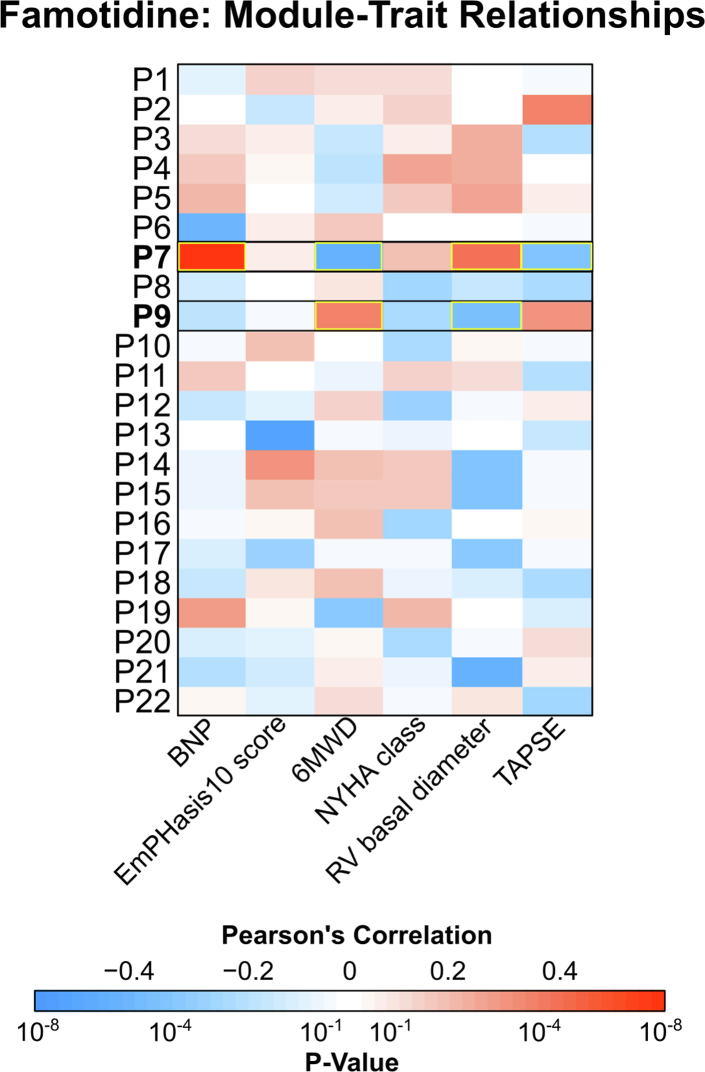
**Weighted Gene Correlation Network Analysis of baseline plasma proteome in the famotidine arm.** Of 22 modules identified representing proteomic endotypes at baseline, two modules (P7 and P9, in bold) in the famotidine arm were associated with clinical response to treatment. The P7 module was associated with clinical worsening in the famotidine arm (increased BNP and RV dilation, decreased 6MWD and TAPSE; all Pearson's correlation p-values <0.05). Conversely, the P9 module was associated with clinical improvement in the famotidine arm (increased 6MWD and reduced RV dilation; Pearson's correlation p-values <0.05).

In exploratory analyses, we used WGCNA to identify baseline metabolites and proteins associated with a beneficial or harmful response to famotidine treatment. Although some patterns could be discerned among metabolites, the associations between the metabolome and clinical response to famotidine were relatively weak and inconsistent (Supplemental Figure S2). In contrast, two distinct proteomic endotypes appeared to have strong and consistent associations with response to famotidine across a range of clinical outcomes. In the famotidine arm, the P7 module (consisting of 73 proteins) correlated with clinical worsening in response to famotidine over 24 weeks including rising BNP, worsening RV dilation, and declines in 6MWD and RV function by TAPSE (Pearson's correlation p-value <0.05) (Fig. 4 and Supplemental Table S4). There were no such associations observed in the placebo arm (Supplemental Figure S3). Over-representation analysis revealed that P7 was enriched for processes related to fat cell differentiation (Supplemental Table S5). Conversely, the P9 module (consisting of 62 proteins) correlated with clinical improvement in the famotidine arm, reflected by increased 6MWD and improved RV dilation (Pearson's correlation p-value <0.05), but showed no consistent correlations in the placebo group (Fig. 4, Supplemental Table S4 and Figure S3). This module mapped to pathways related to axonal transport, extracellular matrix remodelling and positive regulation of immunity (Supplemental Table S5). The robustness of these module-trait relationships was assessed and included in the Supplement (Supplemental Figure S4).

## Discussion

The parent REHAB-PH trial did not meet its primary or secondary clinical endpoints^5^; however, the concurrent multi-omic data collection and analysis highlight several important biologic insights from this trial. First, we found that even in a modest-sized trial of a complex disease, randomisation effectively ameliorates differences in baseline protein and metabolite levels between the treatment and placebo arms. Second, given the paucity of longitudinal multi-omic studies in PAH, leveraging the placebo arm in REHAB-PH indicated that the plasma proteome and metabolome of participants with PAH are relatively stable over 24 weeks in the absence of intervention. Third, even though the famotidine treatment arm did not demonstrate clear clinical benefit, there were measurable alterations in multiple proteomic pathways, demonstrating biologic and molecular impact of H_2_RA therapy in PAH. Finally, our multi-omic analyses identified molecular endotypes that may be associated with enhanced or suppressed responses to famotidine treatment in PAH.

Small clinical trials (n < 100) often face challenges in imbalanced sample size and differences in patient characteristics between treatment and control groups.^12^ This issue is especially important in rare and complex diseases such as PAH, which has heterogeneous causes, advanced medical interventions, and variable clinical trajectories.^1^ By analysing metabolomic and proteomic data collected at enrolment in the REHAB-PH trial, we demonstrated balanced molecular profiles across participants in both treatment arms, highlighting the robustness of randomisation. This was further supported by near-zero mean silhouette widths, suggesting that molecular comparability was maintained despite the limited sample size—an encouraging finding for the design and interpretation of small trials in PAH.

As expected for a complex disease such as PAH, the circulating metabolome and proteome were not static over the 24-week REHAB-PH trial. Our analysis of the placebo arm offers important insight into the longitudinal behaviour of biomarkers in the absence of intervention. Using paired plasma samples collected at baseline and 24 weeks, we quantified within-subject variability for over 8000 proteomic and metabolomic analytes. Individual-level profiles remained largely stable, with fewer than 10% of analytes showing ICCs below 0.10—indicating moderate to high longitudinal stability for the vast majority of proteins and metabolites. This finding is consistent with prior omic studies evaluating protein and metabolite stability over time in non-PAH populations.^11^^,^^13^, ^14^, ^15^ Our results are particularly relevant for the development of biomarkers intended for serial monitoring in PAH, as they suggest that most circulating analytes exhibit modest natural fluctuation over clinically meaningful timeframes in stable patients. Establishing this baseline variability in a biologically undisturbed population is critical for interpreting biomarker changes in future interventional studies and reinforces the importance of incorporating placebo-controlled arms in omics-based biomarker research.

Despite the lack of significant changes in clinical measures with famotidine in REHAB-PH, plasma proteomics suggests substantial molecular perturbations over the trial period. In contrast, global metabolomic changes to famotidine were more subdued and did not reach significance. This finding was reinforced by two observations. First, ICC analysis indicated that only 9.8% of metabolites exhibited low stability (i.e., high variability defined by ICC <0.10) with famotidine treatment, which was similar to that seen in placebo treated participants. In contrast, 19.4% of proteins showed high variability, approximately double that of placebo, underscoring the proteome's greater temporal responsiveness to famotidine. The divergence between omics platforms may reflect inherent turnover kinetics: Metabolites are subject to rapid turnover and strict homoeostatic regulation, which may obscure sustained effects from famotidine, whereas proteins, with slower turnover rates, can accrue cumulative responses to pharmacologic interventions.^16^, ^17^, ^18^ Importantly, these observations may be specific to famotidine and PAH, as other drugs may elicit more pronounced metabolomic effects.^19^ Therefore, if resources permit, we believe metabolomics and proteomics can provide distinct yet complementary information in clinical studies.

Additionally, while famotidine treatment did not show changes in any individual protein after controlling for multiple testing, many proteomic pathways were affected, including those either known to be influenced by H_2_RAs or specifically related to the drug's pharmacokinetics. This suggests that modest, coordinated shifts in individual protein levels converged on shared biological processes and larger functional modules. The biological rationale for this trial stemmed from histamine's ability to activate the Gs-coupled H_2_ receptor, increasing cAMP signalling and thereby influencing cardiac remodelling, endothelial proliferation, and angiogenesis in part through cross-talk with transmembrane RTKs such as VEGFR and EGFR.^20^, ^21^, ^22^, ^23^ The observed downregulation of pathways related to RTK activity is consistent with our hypothesis that H_2_R blockade can attenuate ligand-dependent and ligand-independent RTK activation, potentially dampening signalling cascades (e.g., MAPK/ERK, PI3K/Akt) that support adaptive cardiac responses to stress and endothelial repair after injury.^23^, ^24^, ^25^, ^26^ Ten of the leading-edge proteins contributing to the core enrichment showed nominally significant downregulation (p < 0.05) further suggesting a coordinated suppression of RTK activity with famotidine use (Supplemental Figure S3). Additional pathway changes, including those related to cell turnover, antioxidant and anti-glycation properties, align with known pleiotropic actions of H_2_RAs.^27^, ^28^, ^29^ It is also plausible that the alterations in transmembrane transport pathways reflect famotidine's absorption and distribution characteristics.^30^^,^^31^ While the broader immunological and cellular responses merit further investigation, these proteomic signatures confirm measurable molecular effects of famotidine in treated patients. In the absence of strong single-protein signals, pathway-level findings provide broader context but may be more challenging to directly translate into targeted interventions. Furthermore, the disconnect between molecular and clinical outcomes suggests that, although biologically active, the drug's effects were either insufficient in magnitude or not directly relevant to achieving clinical benefit in participants with PAH.

The goal of the “data-driven” and agnostic WGCNA analyses was to identify putative molecular endotypes that could help define patient subgroups exhibiting either increased or suppressed molecular responsiveness to famotidine. WGCNA on plasma proteomics identified endotypes predictive of famotidine response more effectively than metabolomics. Specifically, it delineated two endotypes of interest in which famotidine was consistently associated with either benefit or harm across a range of clinical outcomes with neither pattern present in the placebo arm. Derangements in pathways related to fat cell differentiation were associated with harm in response to famotidine, while enrichment of immunity and its positive regulation appeared to identify individuals more likely to benefit from famotidine. While our pathway analyses were not granular enough to elucidate how either process influences histamine signalling, the observed associations are biologically plausible given histamine's known roles in both adipogenesis and immune response.^32^^,^^33^ While exploratory, our findings are nonetheless intriguing and may help generate hypotheses for future research.

Our study has several limitations. The sample size of the trial limited our statistical power to detect modest metabolomic and proteomic changes, particularly in a disease as heterogeneous as PAH. To mitigate the risk of false positives, we used a stringent adjusted p-value threshold, which may have filtered out subtle but biologically relevant signals. Nevertheless, the consistency of our findings with known biological processes associated with H_2_RA supports the rigour of our approach. Relatedly, the small sample size and reduced power may have limited our ability to identify significant metabolomic or proteomic differences following randomisation at baseline despite the observation that dimensionality reduction and the near-zero silhouette widths supported balance between the two arms. The lack of significant clinical change in the overall trial also raises questions about the clinical relevance of the observed omics changes. However, these molecular changes provide insight into famotidine's biological activity, which could have longer-term effects that were not captured within the six-month study period. While our endotyping analyses identified potential pathways that could mediate the H_2_RA response and could be theoretically used to enrich a trial for participants most likely to benefit from famotidine, these findings were exploratory and might be difficult to operationalise within the context of enrolment.

In conclusion, we demonstrated the feasibility of integrating metabolomic and proteomic profiling into a prospective clinical trial. Both plasma proteome and metabolome appeared to be stable over six months in the placebo arm, with low within-subject variability, reinforcing the reliability of using omics platforms for longitudinal biomarker discovery. We identified significant proteomic signals related to famotidine treatment despite the lack of clinical differences in this PAH trial. Our findings raise the possibility that specific molecular endotypes may exhibit distinct susceptibilities to famotidine, warranting further investigation. These results highlight the utility of leveraging multi-omics for precision medicine in PAH by uncovering treatment-related biological responses and informing the design and interpretation of future trials.

## Contributors

HP, LX, PJL, and SAG developed the initial study concept, study design and methodology. HP was responsible for data processing and formal analysis. HP wrote the original draft with input and supervision from LX, PJL, and SAG. SGR, JLP, KRB, and AS all contributed to data interpretation. PJL and SAG have accessed and verified the underlying data. All authors reviewed and edited the draft, approved the final version, and had responsibility for the decision to submit the article. PJL acts as guarantor for the work.

## Data sharing statement

De-identified individual participant data and the associated data dictionary will be available at NIH BioData Catalyst (https://biodatacatalyst.nhlbi.nih.gov/). This will be searchable under the parent study (Repurposing a Histamine Antagonist to Benefit Patients With Pulmonary Hypertension Trial (REHAB-PH)). At the time of publication, data deposition is in process and access will be provided in accordance with Catalyst policies and applicable regulatory requirements. Other data will be made available on reasonable request to the corresponding author. A proposal with detailed description of study objectives and statistical analysis plan will be needed for evaluation of the reasonableness of requests.

## Declaration of interests

HP, SGR, SAG and PJL have received research funding from National Institutes of Health. HP has received research funding from the American Heart Association. SGR has received research funding from Bayer Pharmaceuticals and United Therapeutics. KRB has received grants from Kestra, Bayer, Janssen, Amgen, Novartis and Nanox AI. He has received consultation fees for Bayer, Janssen, Amgen and Cleerly, and non-financial support from Bayer. PJL has received grants and research support from the Cystic Fibrosis Foundation, Bayer, and Janssen. He has been a grant review consultant for Bayer and has adjudicated clinical endpoints for Sumitomo Pharma. He is on the scientific leadership council for the Pulmonary Hypertension Association and medical advisory board for Team PHenomenal Hope. All the other authors have no disclosures.
