# Supplementary material for: Harnessing metabolomics and proteomics in a clinical trial for pulmonary arterial hypertension: insights from post-hoc analysis of the REHAB-PH trial
Source: eBioMedicine. 2026 Mar 17;126:106191. doi: 10.1016/j.ebiom.2026.106191 (PMC13014658; doi:10.1016/j.ebiom.2026.106191)
Supplement: Supplementary Materials [file mmc1.docx]

**Supplemental Materials**

**Table of Contents**

**Methods…………………………………………………………………………………...……2-3**

**Tables S1-5………………………………………………………………………………...…4-140**

**Figure S1-4……………………………………………………………………………..…141-144**

**Supplemental Methods**

***Imputation on missing metabolite data***

Missing percentages of each metabolite were first calculated within each aetiology subgroup. Metabolites with more than 50% missingness were excluded from subsequent analyses. For the remaining metabolites, a random value realized from a uniform distribution between 0.5 and 1 times of the minimum observed value was imputed for subgroups where more than 50% values of the metabolite in question were missing, and the remaining missing values were imputed using the K-nearest neighbour approach in the R package “impute”.^1^

***Longitudinal variability of analytes – Intraclass correlation coefficient (ICC)***

For each metabolite and protein, the ratio of variability between individuals and the variability between baseline and follow-up timepoints were estimated with the following linear mixed model. For a given analyte, measurement for timepoint j and subject i was modelled with:

Equation 1: model for computing the ICC

Analyte_ij_ = µ + Random intercept_i_ + AGE_i_ + SEX_i_ + BMI_i_ + PAH AETIOLOGY_i_ + ɛ_ij_

ICC is the ratio of inter-subject variance and total variance, which has been estimated with: ICC = σ^2^_inter_/σ^2^_total_

ICC around 0 means low correlation between observations for the same subject, whereas ICC around 1 means high correlation between observations for the same subject.^2,3^

***Statistical analyses for treatment effects***

Paired analyses were conducted on participants with complete measurements at both timepoints (n=71). For each analyte, the change over time was calculated by subtracting the baseline value from the follow-up value and differences were modelled using multivariate linear regression with the following specification:

Analyte difference ~ treatment + AGE + SEX + BMI + PAH AETIOLOGY

The placebo group was set as reference for the treatment variable. All p-values were adjusted for multiple testing using the Benjamini-Hochberg procedure to control the false discovery rate (FDR), with statistical significance defined as adjusted p-value < 0.05.

***Pathway analysis***

Metabolomic pathway annotations were curated by Metabolon, Inc. The 1,045 named metabolites measured mapped to a total of 104 metabolic pathways within 9 superpathways. The functional class scoring method, globaltest,^4^ was used to identify dysregulated pathways for each outcome of interest. This approach tests whether a set of metabolites within a pathway, as a whole, is associated with an outcome of interest (in our case, the treatment famotidine), rather than individually as with Welch’s two-sample t-test.

For the proteomic pathway analysis using gene set enrichment analysis (GSEA),^5^ aptamers with multiple associated gene symbols were reduced to retain only the first gene symbol and duplicated gene symbols were eliminated with only the aptamers with the largest coefficient of variation remaining. Preranked lists of these resulting gene symbols and their associated statistics from treatment-related linear regression were used. Finally, networks of highly inter-connected pathways (referred to as modules) were constructed and visualized using Cytoscape 3.9.^6^

FDR was controlled by defining significance as Benjamini-Hochberg adjusted p-values < 0.05 for metabolomic pathways and permutation-based FDR q-values < 0.05 (1,000 permutations) for proteomic pathways.

***Weighted Gene Co-expression Network Analysis (WGCNA)***

An expression matrix of baseline samples from 39 participants in the famotidine arm was used to construct weighted co-expression networks for 1,118 metabolites and 7,289 proteins using the WGCNA R package.^7^ Pairwise Pearson correlations were first calculated to generate a co-expression matrix. The “pickSoftThreshold” function was then used to identify an appropriate soft-thresholding power, chosen based on achieving a scale-free topology fit index above 0.8 while maintaining adequate mean connectivity. The resulting adjacency matrix was transformed into the topological overlap matrix (TOM), and modules were identified using hierarchical clustering with the dynamic tree cut method. The minimal module size was set to 20 for metabolites and 50 for proteins. Modules with similar expression profiles were merged using a height cutoff of 0.25.

Module eigengenes (MEs), defined by the first principal component of each module, were computed to summarize expression patterns.^7^ The Pearson correlation between MEs and clinical traits was then calculated to assess the module-trait relationship. Modules showing the strongest positive or negative correlations with at least two clinical traits were designated as key modules. To evaluate consistency across treatment arms, baseline samples from 40 participants in the placebo arm were mapped onto the predefined MEs, and the module-trait correlations were recomputed and compared between the famotidine and placebo groups.

For selected modules of interest, the robustness of the module-trait relationship was further assessed using the MM-GS scatterplots generated by the “verboseScatterplot” function. Module membership (MM) was defined as the correlation of each protein with the ME, and gene significance (GS) as the correlation of each protein with the clinical trait. For each module-trait pair, a correlation between MM and GS was calculated across all proteins in the model, providing a quantitative measure of whether the observed module-trait relationship was broadly supported rather than driven by a few outliers.^7^ The top five genes with the highest values for both metrics were prioritized for further biological investigation.^7^ Correlations between these hub genes and clinical traits were examined to determine the directionality of associations.

Enrichment analysis of the key proteomic modules were done via overrepresentation analysis using the “enrichGO” function in the clusterProfiler (v4.0) R package.^8^ Module gene lists (Entrez IDs) were tested against the Homo sapiens GO annotation (org.Hs.eg.db) across all ontologies (BP, CC, MF). P-values were adjusted by the Benjamini–Hochberg procedure and GO terms with an adjusted p-value < 0.10 were deemed statistically significant.

**References**

1. Hastie T, Tibshirani R, Narasimhan B, Chu G. impute: Imputation for microarray data. 2020. R package version 1.70.0.
2. Koch GG. Intraclass correlation coefficient. In: Kotz S, Johnson N, eds. *Encyclopedia of statistical sciences*. Hoboken, NJ:Wiley, 1983: 213-17.
3. Agueusop I, Musholt PB, Klaus B, et al. Short-term variability of the human serum metabolome depending on nutritional and metabolic health status. *Sci Rep.* 2020;10:16310.
4. Goeman JJ, van de Geer SA, de Kort F, van Houwelingen HC. A global test for groups of genes: testing association with a clinical outcome. *Bioinformatics*. 2004;20:93-9.
5. Subramanian A, Tamayo P, Mootha VK, et al. Gene set enrichment analysis: a knowledge-based approach for interpreting genome-wide expression profiles. *Proc Natl Acad Sci USA*. 2005;102:15545-15550.
6. Shannon P, Markiel A, Ozier O, Baliga NS, Wang JT, Ramage D, Amin N, Schwikowski B, Ideker T. Cytoscape: a software environment for integrated models of biomolecular interaction networks. Genome Res. 2003 Nov;13(11):2498-504.
7. Langfelder P, Horvath S. WGCNA: an R package for weighted correlation network analysis. *BMC Bioinf*. 2008;9:559.
8. Wu T, Hu E, Xu S, et al. clusterProfiler 4.0: A universal enrichment tool for interpreting omics data. *The Innovation*. 2021;2:100141.

**Table S1: Intraclass correlation coefficients for metabolites by treatment arms.**

| **biochemical name** | **placebo ICC** | **famotidine ICC** |
| --- | --- | --- |
| S-1-pyrroline-5-carboxylate | 0.396 | 0.188 |
| spermidine | 0.405 | 0.429 |
| 1-methylnicotinamide | 0.556 | 0.438 |
| 12,13-DiHOME | 0.148 | 0.000 |
| 5-hydroxyindoleacetate | 0.537 | 0.480 |
| alpha-ketoglutarate | 0.775 | 0.689 |
| kynurenate | 0.578 | 0.802 |
| 3-hydroxyisobutyrate | 0.162 | 0.337 |
| 3-hydroxy-3-methylglutarate | 0.677 | 0.558 |
| homovanillate (HVA) | 0.210 | 0.115 |
| 3-phosphoglycerate | 0.303 | 0.212 |
| cholate | 0.537 | 0.349 |
| 4-hydroxyphenylacetate | 0.535 | 0.579 |
| 5,6-dihydrothymine | 0.276 | 0.446 |
| hypoxanthine | 0.116 | 0.128 |
| 9,10-DiHOME | 0.000 | 0.040 |
| linoleate (18:2n6) | 0.159 | 0.000 |
| laurate (12:0) | 0.452 | 0.215 |
| quinolinate | 0.645 | 0.683 |
| N6,N6,N6-trimethyllysine | 0.681 | 0.600 |
| N-acetylputrescine | 0.815 | 0.822 |
| N-formylmethionine | 0.752 | 0.761 |
| S-adenosylhomocysteine (SAH) | 0.684 | 0.494 |
| adenosine 3',5'-cyclic monophosphate (cAMP) | 0.693 | 0.599 |
| ADP | 0.188 | 0.000 |
| AMP | 0.171 | 0.076 |
| 5-methylthioadenosine (MTA) | 0.519 | 0.460 |
| arachidonate (20:4n6) | 0.380 | 0.471 |
| arginine | 0.341 | 0.410 |
| aspartate | 0.160 | 0.393 |
| 2-hydroxyphenylacetate | 0.668 | 0.540 |
| 3-(4-hydroxyphenyl)lactate (HPLA) | 0.751 | 0.766 |
| phenylpyruvate | 0.222 | 0.350 |
| beta-alanine | 0.287 | 0.284 |
| carnosine | 0.000 | 0.405 |
| biliverdin | 0.534 | 0.369 |
| succinate | 0.000 | 0.000 |
| 3-hydroxybutyrate (BHBA) | 0.083 | 0.000 |
| cholesterol | 0.735 | 0.693 |
| phosphocholine | 0.533 | 0.000 |
| corticosterone | 0.000 | 0.418 |
| cortisone | 0.597 | 0.289 |
| creatinine | 0.851 | 0.497 |
| cysteinylglycine | 0.396 | 0.347 |
| cystine | 0.567 | 0.402 |
| sphingosine | 0.399 | 0.307 |
| deoxycholate | 0.261 | 0.634 |
| cystathionine | 0.548 | 0.437 |
| sphinganine | 0.505 | 0.348 |
| fumarate | 0.218 | 0.664 |
| gamma-glutamylglutamate | 0.714 | 0.227 |
| gluconate | 0.755 | 0.754 |
| glutarate (C5-DC) | 0.561 | 0.149 |
| glycine | 0.802 | 0.833 |
| glycocholate | 0.729 | 0.439 |
| guanidinoacetate | 0.698 | 0.717 |
| histidine | 0.689 | 0.361 |
| cortisol | 0.350 | 0.019 |
| hypotaurine | 0.228 | 0.280 |
| inosine | 0.251 | 0.000 |
| myo-inositol | 0.358 | 0.555 |
| isoleucine | 0.333 | 0.198 |
| 2-aminoadipate | 0.343 | 0.448 |
| citrulline | 0.505 | 0.681 |
| lactose | 0.108 | 0.280 |
| leucine | 0.366 | 0.106 |
| lithocholate | 0.504 | 0.553 |
| lysine | 0.362 | 0.332 |
| malate | 0.593 | 0.496 |
| methionine | 0.183 | 0.054 |
| palmitate (16:0) | 0.175 | 0.121 |
| nicotinamide | 0.000 | 0.028 |
| stearate (18:0) | 0.302 | 0.242 |
| ornithine | 0.350 | 0.365 |
| orotate | 0.593 | 0.647 |
| palmitoleate (16:1n7) | 0.309 | 0.046 |
| phenylalanine | 0.523 | 0.289 |
| phosphate | 0.000 | 0.108 |
| phytanate | 0.273 | 0.398 |
| pristanate | 0.493 | 0.378 |
| proline | 0.441 | 0.601 |
| lactate | 0.348 | 0.236 |
| pyridoxal | 0.607 | 0.015 |
| retinol (vitamin A) | 0.806 | 0.755 |
| serine | 0.469 | 0.596 |
| serotonin | 0.227 | 0.000 |
| taurine | 0.380 | 0.292 |
| myristate (14:0) | 0.444 | 0.088 |
| urea | 0.535 | 0.263 |
| uridine | 0.000 | 0.183 |
| 2'-deoxyuridine | 0.296 | 0.378 |
| trans-urocanate | 0.372 | 0.292 |
| glutamate | 0.427 | 0.457 |
| glutamine | 0.564 | 0.474 |
| threonine | 0.319 | 0.409 |
| tryptophan | 0.533 | 0.283 |
| valine | 0.453 | 0.402 |
| glucose | 0.000 | 0.594 |
| alpha-ketobutyrate | 0.040 | 0.325 |
| adenosine | 0.000 | 0.105 |
| betaine | 0.797 | 0.421 |
| cysteine | 0.211 | 0.000 |
| mannose | 0.227 | 0.031 |
| dimethylglycine | 0.781 | 0.768 |
| alanine | 0.591 | 0.216 |
| tyrosine | 0.360 | 0.560 |
| malonate | 0.140 | 0.000 |
| pseudouridine | 0.896 | 0.671 |
| pyruvate | 0.299 | 0.357 |
| uracil | 0.000 | 0.023 |
| xylose | 0.000 | 0.065 |
| cytidine | 0.607 | 0.588 |
| arabinose | 0.219 | 0.093 |
| caffeine | 0.306 | 0.568 |
| thymidine | 0.420 | 0.309 |
| fructose | 0.012 | 0.000 |
| adenine | 0.049 | 0.146 |
| cytosine | 0.462 | 0.146 |
| caprate (10:0) | 0.473 | 0.209 |
| margarate (17:0) | 0.281 | 0.071 |
| nonadecanoate (19:0) | 0.170 | 0.235 |
| arachidate (20:0) | 0.087 | 0.368 |
| maltose | 0.063 | 0.185 |
| asparagine | 0.421 | 0.375 |
| N-stearoyl-sphinganine (d18:0/18:0)* | 0.662 | 0.578 |
| dihydroorotate | 0.252 | 0.483 |
| heptanoate (7:0) | 0.148 | 0.187 |
| caproate (6:0) | 0.161 | 0.180 |
| caprylate (8:0) | 0.131 | 0.244 |
| sucrose | 0.349 | 0.043 |
| pentadecanoate (15:0) | 0.486 | 0.018 |
| hydroxyproline | 0.000 | 0.602 |
| allantoin | 0.194 | 0.350 |
| xanthine | 0.669 | 0.622 |
| 5-oxoproline | 0.000 | 0.715 |
| picolinate | 0.474 | 0.631 |
| sarcosine | 0.061 | 0.218 |
| pantothenate (Vitamin B5) | 0.700 | 0.413 |
| pipecolate | 0.265 | 0.408 |
| phosphoethanolamine (PE) | 0.338 | 0.000 |
| glycerate | 0.507 | 0.630 |
| 3-ureidopropionate | 0.468 | 0.512 |
| N-acetylleucine | 0.068 | 0.347 |
| N-acetylmethionine | 0.597 | 0.753 |
| N-acetylvaline | 0.446 | 0.689 |
| erucate (22:1n9) | 0.130 | 0.016 |
| bilirubin | 0.728 | 0.504 |
| thyroxine | 0.850 | 0.745 |
| guanosine | 0.155 | 0.000 |
| gamma-glutamyltyrosine | 0.557 | 0.628 |
| methyl indole-3-acetate | 0.647 | 0.539 |
| alpha-tocopherol | 0.688 | 0.600 |
| N-acetylalanine | 0.780 | 0.605 |
| vanillylmandelate (VMA) | 0.841 | 0.642 |
| 4-acetamidobutanoate | 0.835 | 0.551 |
| 3-aminoisobutyrate | 0.676 | 0.605 |
| chenodeoxycholate | 0.528 | 0.260 |
| citrate | 0.000 | 0.582 |
| 5,6-dihydrouracil | 0.000 | 0.000 |
| 2-aminobutyrate | 0.459 | 0.306 |
| urate | 0.680 | 0.550 |
| ursodeoxycholate | 0.390 | 0.380 |
| valerate (5:0) | 0.200 | 0.169 |
| oleoyl ethanolamide | 0.027 | 0.328 |
| gamma-glutamylglutamine | 0.413 | 0.460 |
| 4-hydroxyphenylpyruvate | 0.479 | 0.622 |
| butyrate/isobutyrate (4:0) | 0.323 | 0.688 |
| N-acetylneuraminate | 0.841 | 0.972 |
| N-acetylglucosaminylasparagine | 0.560 | 0.307 |
| acetoacetate | 0.173 | 0.000 |
| creatine | 0.536 | 0.081 |
| cys-gly, oxidized | 0.495 | 0.754 |
| dihomolinoleate (20:2n6) | 0.222 | 0.042 |
| gamma-glutamylhistidine | 0.210 | 0.000 |
| 2-hydroxystearate | 0.147 | 0.226 |
| 1-methyladenosine | 0.566 | 0.536 |
| glycerol | 0.308 | 0.314 |
| choline | 0.243 | 0.602 |
| anthranilate | 0.466 | 0.541 |
| gamma-glutamylleucine | 0.496 | 0.361 |
| 3-methoxytyrosine | 0.882 | 0.822 |
| beta-hydroxyisovalerate | 0.623 | 0.256 |
| palmitoyl ethanolamide | 0.229 | 0.369 |
| linoleamide (18:2n6) | 0.517 | 0.445 |
| N-palmitoyl-sphingosine (d18:1/16:0) | 0.531 | 0.627 |
| 1-palmitoyl-2-oleoyl-GPE (16:0/18:1) | 0.684 | 0.568 |
| 1-palmitoyl-2-linoleoyl-GPI (16:0/18:2) | 0.695 | 0.714 |
| 1-palmitoyl-2-linoleoyl-GPC (16:0/18:2) | 0.734 | 0.766 |
| stearoyl sphingomyelin (d18:1/18:0) | 0.564 | 0.730 |
| 1-palmitoyl-2-oleoyl-GPC (16:0/18:1) | 0.724 | 0.570 |
| N-stearoyl-sphingosine (d18:1/18:0)* | 0.432 | 0.625 |
| glycochenodeoxycholate | 0.562 | 0.130 |
| taurochenodeoxycholate | 0.609 | 0.636 |
| taurocholate | 0.654 | 0.599 |
| taurodeoxycholate | 0.494 | 0.426 |
| 2-hydroxyhippurate (salicylurate) | 0.290 | 0.763 |
| azelate (C9-DC) | 0.000 | 0.000 |
| eicosapentaenoate (EPA; 20:5n3) | 0.528 | 0.469 |
| methylsuccinate | 0.455 | 0.690 |
| ethylmalonate | 0.863 | 0.853 |
| carnitine | 0.611 | 0.075 |
| benzoate | 0.000 | 0.024 |
| 3-phenylpropionate (hydrocinnamate) | 0.605 | 0.374 |
| phenylacetate | 0.578 | 0.464 |
| hippurate | 0.221 | 0.452 |
| xanthurenate | 0.338 | 0.243 |
| suberate (C8-DC) | 0.233 | 0.339 |
| 3-methyl-2-oxovalerate | 0.315 | 0.092 |
| methionine sulfoxide | 0.389 | 0.506 |
| 3-methylhistidine | 0.079 | 0.057 |
| 5-hydroxylysine | 0.183 | 0.575 |
| 4-guanidinobutanoate | 0.432 | 0.093 |
| FMN | 0.055 | 0.000 |
| glucuronate | 0.660 | 0.482 |
| glycerol 3-phosphate | 0.681 | 0.358 |
| imidazole lactate | 0.695 | 0.326 |
| kynurenine | 0.616 | 0.571 |
| glycerophosphorylcholine (GPC) | 0.286 | 0.290 |
| allo-threonine | 0.000 | 0.000 |
| maltotetraose | 0.308 | 0.111 |
| maltotriose | 0.105 | 0.317 |
| N-acetylglutamate | 0.490 | 0.557 |
| xanthosine | 0.244 | 0.520 |
| ribitol | 0.522 | 0.605 |
| 2-isopropylmalate | 0.213 | 0.040 |
| glycodeoxycholate | 0.713 | 0.126 |
| theophylline | 0.466 | 0.815 |
| quinate | 0.625 | 0.786 |
| theobromine | 0.215 | 0.491 |
| gentisate | 0.367 | 0.613 |
| paraxanthine | 0.416 | 0.659 |
| indolelactate | 0.809 | 0.593 |
| 3-indoxyl sulfate | 0.811 | 0.762 |
| gamma-glutamylphenylalanine | 0.610 | 0.526 |
| 4-methyl-2-oxopentanoate | 0.337 | 0.045 |
| 1,5-anhydroglucitol (1,5-AG) | 0.911 | 0.823 |
| 1-palmityl-GPC (O-16:0) | 0.646 | 0.707 |
| 1-stearoyl-2-arachidonoyl-GPI (18:0/20:4) | 0.662 | 0.653 |
| sphingosine 1-phosphate | 0.111 | 0.342 |
| 1-stearoyl-2-oleoyl-GPS (18:0/18:1) | 0.126 | 0.000 |
| 1-stearoyl-GPI (18:0) | 0.300 | 0.611 |
| 1,2-dipalmitoyl-GPC (16:0/16:0) | 0.682 | 0.786 |
| docosahexaenoate (DHA; 22:6n3) | 0.440 | 0.544 |
| 1-myristoyl-2-palmitoyl-GPC (14:0/16:0) | 0.506 | 0.412 |
| alpha-hydroxyisocaproate | 0.551 | 0.153 |
| maleate | 0.160 | 0.492 |
| isovalerate (C5) | 0.268 | 0.224 |
| 4-acetylphenyl sulfate | 0.159 | 0.125 |
| 1-methylguanidine | 0.588 | 0.295 |
| 2-hydroxyoctanoate | 0.345 | 0.076 |
| levulinate (4-oxovalerate) | 0.000 | 0.289 |
| 3-hydroxyoctanoate | 0.304 | 0.543 |
| phenyllactate (PLA) | 0.446 | 0.394 |
| palmitoylcarnitine (C16) | 0.329 | 0.432 |
| hexanoylcarnitine (C6) | 0.717 | 0.376 |
| N-acetylaspartate (NAA) | 0.367 | 0.244 |
| dehydroepiandrosterone sulfate (DHEA-S) | 0.957 | 0.919 |
| acetylcarnitine (C2) | 0.588 | 0.334 |
| cysteine s-sulfate | 0.060 | 0.346 |
| tartronate (hydroxymalonate) | 0.000 | 0.143 |
| oxalate (ethanedioate) | 0.466 | 0.819 |
| erythritol | 0.598 | 0.000 |
| 2,3-dihydroxypyridine | 0.397 | 0.704 |
| adipate | 0.192 | 0.195 |
| saccharin | 0.303 | 0.384 |
| 3-hydroxymyristate | 0.514 | 0.385 |
| iminodiacetate (IDA) | 0.035 | 0.298 |
| 1-oleoylglycerol (18:1) | 0.035 | 0.000 |
| 3-methyl-2-oxobutyrate | 0.237 | 0.230 |
| 1,6-anhydroglucose | 0.142 | 0.352 |
| homoarginine | 0.794 | 0.576 |
| homocitrulline | 0.277 | 0.398 |
| 3-hydroxydecanoate | 0.425 | 0.483 |
| citramalate | 0.144 | 0.442 |
| EDTA | 0.004 | 0.000 |
| N-acetylglycine | 0.703 | 0.549 |
| ribonate | 0.607 | 0.645 |
| threonate | 0.548 | 0.806 |
| galactonate | 0.408 | 0.475 |
| beta-sitosterol | 0.116 | 0.460 |
| indoleacetate | 0.698 | 0.697 |
| 1-linoleoylglycerol (18:2) | 0.343 | 0.126 |
| 1-methylhistidine | 0.769 | 0.523 |
| butyrylcarnitine (C4) | 0.864 | 0.741 |
| isobutyrylcarnitine (C4) | 0.616 | 0.718 |
| glycolithocholate | 0.365 | 0.180 |
| taurolithocholate | 0.114 | 0.249 |
| androsterone sulfate | 0.887 | 0.961 |
| 2-pyrrolidinone | 0.725 | 0.629 |
| indolepropionate | 0.659 | 0.610 |
| N-(2-furoyl)glycine | 0.124 | 0.428 |
| trigonelline (N'-methylnicotinate) | 0.699 | 0.684 |
| dodecanedioate (C12) | 0.592 | 0.468 |
| N-acetyltyrosine | 0.585 | 0.141 |
| 1,3-dimethylurate | 0.561 | 0.685 |
| 3-methylxanthine | 0.311 | 0.345 |
| 3-hydroxylaurate | 0.525 | 0.353 |
| pyridoxate | 0.692 | 0.235 |
| gamma-glutamylvaline | 0.270 | 0.371 |
| pyroglutamylglycine | 0.000 | 0.000 |
| O-acetylhomoserine | 0.037 | 0.160 |
| pyroglutamylvaline | 0.563 | 0.694 |
| 3-hydroxysebacate | 0.402 | 0.483 |
| 3-methylcrotonylglycine | 0.641 | 0.336 |
| propionylglycine (C3) | 0.131 | 0.000 |
| 2-methylbutyrylglycine (C5) | 0.355 | 0.205 |
| propionylcarnitine (C3) | 0.604 | 0.345 |
| prolylhydroxyproline | 0.445 | 0.693 |
| 3-hydroxy-2-ethylpropionate | 0.727 | 0.453 |
| 3-carboxy-4-methyl-5-propyl-2-furanpropanoate (CMPF) | 0.864 | 0.698 |
| 2-hydroxymyristate | 0.128 | 0.241 |
| docosapentaenoate (DPA; 22:5n3) | 0.294 | 0.302 |
| docosadienoate (22:2n6) | 0.211 | 0.143 |
| adrenate (22:4n6) | 0.501 | 0.345 |
| docosatrienoate (22:3n3) | 0.165 | 0.142 |
| 10-undecenoate (11:1n1) | 0.604 | 0.294 |
| myristoleate (14:1n5) | 0.475 | 0.080 |
| 4-imidazoleacetate | 0.247 | 0.073 |
| 1-methyl-4-imidazoleacetate | 0.743 | 0.468 |
| sebacate (C10-DC) | 0.450 | 0.462 |
| guanidinosuccinate | 0.702 | 0.461 |
| delta-tocopherol | 0.238 | 0.207 |
| I-urobilinogen | 0.372 | 0.435 |
| stearidonate (18:4n3) | 0.385 | 0.176 |
| 5-dodecenoate (12:1n7) | 0.534 | 0.123 |
| octanoylcarnitine (C8) | 0.663 | 0.389 |
| tauro-beta-muricholate | 0.722 | 0.758 |
| decanoylcarnitine (C10) | 0.618 | 0.291 |
| N-acetylglutamine | 0.716 | 0.734 |
| N-acetyltryptophan | 0.730 | 0.551 |
| N-acetylphenylalanine | 0.600 | 0.464 |
| N-acetylasparagine | 0.733 | 0.528 |
| 1-palmitoyl-GPC (16:0) | 0.615 | 0.408 |
| N-acetylarginine | 0.623 | 0.584 |
| piperine | 0.659 | 0.398 |
| campesterol | 0.000 | 0.443 |
| myristoylcarnitine (C14) | 0.552 | 0.308 |
| 1-stearoyl-GPC (18:0) | 0.635 | 0.372 |
| 1-oleoyl-GPC (18:1) | 0.584 | 0.409 |
| N-acetylthreonine | 0.809 | 0.566 |
| phenylacetylglycine | 0.040 | 0.240 |
| N-acetylisoleucine | 0.348 | 0.281 |
| 10-nonadecenoate (19:1n9) | 0.263 | 0.000 |
| 10-heptadecenoate (17:1n7) | 0.267 | 0.000 |
| hyocholate | 0.243 | 0.115 |
| epiandrosterone sulfate | 0.917 | 0.933 |
| N-acetylhistidine | 0.037 | 0.470 |
| gamma-glutamylglycine | 0.792 | 0.750 |
| gamma-glutamyltryptophan | 0.476 | 0.503 |
| stachydrine | 0.652 | 0.655 |
| alpha-hydroxyisovalerate | 0.850 | 0.626 |
| gamma-glutamylmethionine | 0.359 | 0.137 |
| gamma-glutamylthreonine | 0.468 | 0.528 |
| p-cresol sulfate | 0.678 | 0.856 |
| erythronate* | 0.523 | 0.437 |
| N-acetylproline | 0.168 | 0.416 |
| eicosenoate (20:1n9 or 1n11) | 0.133 | 0.003 |
| linolenate (18:3n3 or 3n6) | 0.089 | 0.000 |
| aconitate [cis or trans] | 0.084 | 0.620 |
| 1-myristoyl-GPC (14:0) | 0.483 | 0.225 |
| 1-arachidoyl-GPC (20:0) | 0.624 | 0.307 |
| heme | 0.262 | 0.093 |
| stearoylcarnitine (C18) | 0.315 | 0.625 |
| laurylcarnitine (C12) | 0.618 | 0.129 |
| isovalerylcarnitine (C5) | 0.470 | 0.269 |
| 1-linoleoyl-GPC (18:2) | 0.673 | 0.414 |
| 7-methylxanthine | 0.280 | 0.683 |
| 1,3,7-trimethylurate | 0.515 | 0.608 |
| 3,7-dimethylurate | 0.405 | 0.414 |
| 1,7-dimethylurate | 0.499 | 0.701 |
| 1-methylurate | 0.712 | 0.762 |
| 5-acetylamino-6-formylamino-3-methyluracil | 0.392 | 0.794 |
| 5-acetylamino-6-amino-3-methyluracil | 0.556 | 0.814 |
| indolebutyrate | 0.629 | 0.370 |
| 1-methylxanthine | 0.720 | 0.530 |
| N1-methylinosine | 0.800 | 0.484 |
| beta-guanidinopropanoate | 0.306 | 0.099 |
| N2,N2-dimethylguanosine | 0.767 | 0.523 |
| N4-acetylcytidine | 0.841 | 0.793 |
| N6-carbamoylthreonyladenosine | 0.800 | 0.735 |
| orotidine | 0.918 | 0.866 |
| phenylacetylglutamine | 0.548 | 0.602 |
| 4-hydroxyhippurate | 0.295 | 0.541 |
| 5,6-dihydrouridine | 0.894 | 0.773 |
| 3-(3-amino-3-carboxypropyl)uridine* | 0.906 | 0.769 |
| cysteine-glutathione disulfide | 0.576 | 0.425 |
| 5-methyluridine (ribothymidine) | 0.550 | 0.578 |
| isovalerylglycine | 0.398 | 0.312 |
| 3-hydroxydodecanedioate* | 0.490 | 0.508 |
| 7-methylguanine | 0.641 | 0.718 |
| 1-stearoyl-GPE (18:0) | 0.615 | 0.654 |
| 1-stearoyl-GPG (18:0) | 0.391 | 0.476 |
| 3-methylcytidine | 0.353 | 0.591 |
| N1-methyl-2-pyridone-5-carboxamide | 0.714 | 0.360 |
| N1-methyl-4-pyridone-3-carboxamide | 0.722 | 0.256 |
| gamma-glutamylisoleucine* | 0.512 | 0.257 |
| oleoylcarnitine (C18:1) | 0.181 | 0.511 |
| gamma-glutamyl-2-aminobutyrate | 0.072 | 0.365 |
| 2-methylbutyrylcarnitine (C5) | 0.603 | 0.281 |
| phenol sulfate | 0.647 | 0.788 |
| 1-palmitoleoyl-GPC (16:1)* | 0.640 | 0.584 |
| hexanoylglycine (C6) | 0.499 | 0.347 |
| 2-amino-4-cyanobutanoate | 0.579 | 0.531 |
| 2-hydroxy-3-methylvalerate | 0.434 | 0.151 |
| homostachydrine* | 0.360 | 0.441 |
| 1-arachidonoyl-GPC (20:4n6)* | 0.753 | 0.905 |
| 1-dihomo-linolenoyl-GPC (20:3n3 or 6)* | 0.549 | 0.421 |
| 1-eicosadienoyl-GPC* (20:2)* | 0.569 | 0.230 |
| 2-arachidonoyl-GPC (20:4)* | 0.580 | 0.750 |
| 2-stearoyl-GPC* (18:0)* | 0.459 | 0.265 |
| 2-oleoyl-GPC (18:1)* | 0.436 | 0.307 |
| 2-linoleoyl-GPC (18:2)* | 0.655 | 0.328 |
| 2-palmitoleoyl-GPC (16:1)* | 0.520 | 0.555 |
| 2-palmitoyl-GPC (16:0)* | 0.400 | 0.300 |
| 2-myristoyl-GPC (14:0)* | 0.497 | 0.234 |
| 2-docosahexaenoyl-GPC (22:6)* | 0.282 | 0.688 |
| 1-docosahexaenoyl-GPC (22:6)* | 0.573 | 0.804 |
| 1-palmitoyl-GPE (16:0) | 0.630 | 0.701 |
| 1-oleoyl-GPE (18:1) | 0.387 | 0.184 |
| 1-linoleoyl-GPE (18:2)* | 0.666 | 0.437 |
| 1-arachidonoyl-GPE (20:4n6)* | 0.534 | 0.773 |
| N-acetylcitrulline | 0.552 | 0.817 |
| 2-hydroxypalmitate | 0.292 | 0.307 |
| docosapentaenoate (n6 DPA; 22:5n6) | 0.466 | 0.417 |
| gulonate* | 0.804 | 0.509 |
| isobutyrylglycine (C4) | 0.524 | 0.436 |
| glutarylcarnitine (C5-DC) | 0.792 | 0.691 |
| beta-hydroxyisovaleroylcarnitine | 0.668 | 0.541 |
| tiglyl carnitine (C5) | 0.445 | 0.666 |
| catechol sulfate | 0.648 | 0.637 |
| cholesterol sulfate | 0.532 | 0.585 |
| 7-HOCA | 0.539 | 0.807 |
| 3beta,7alpha-dihydroxy-5-cholestenoate | 0.639 | 0.541 |
| N-acetyl-aspartyl-glutamate (NAAG) | 0.752 | 0.496 |
| tetradecanedioate (C14) | 0.695 | 0.321 |
| hexadecanedioate (C16) | 0.555 | 0.418 |
| octadecanedioate (C18) | 0.273 | 0.112 |
| undecanedioate (C11-DC) | 0.076 | 0.282 |
| glycerophosphoethanolamine | 0.596 | 0.201 |
| glycerophosphoinositol* | 0.010 | 0.335 |
| 3-(3-hydroxyphenyl)propionate | 0.442 | 0.464 |
| ectoine | 0.245 | 0.000 |
| 2-oleoyl-GPE (18:1)* | 0.403 | 0.237 |
| 1-arachidonoyl-GPI (20:4)* | 0.539 | 0.698 |
| 1-palmitoyl-GPI (16:0) | 0.299 | 0.635 |
| glycolithocholate sulfate* | 0.613 | 0.307 |
| taurolithocholate 3-sulfate | 0.389 | 0.406 |
| deoxycarnitine | 0.825 | 0.515 |
| N6-succinyladenosine | 0.889 | 0.697 |
| 1-ribosyl-imidazoleacetate* | 0.592 | 0.479 |
| 2-arachidonoyl-GPE (20:4)* | 0.500 | 0.531 |
| 3,4-dihydroxybutyrate | 0.616 | 0.290 |
| indoleacetylglutamine | 0.703 | 0.653 |
| hexanoylglutamine | 0.465 | 0.375 |
| N6-acetyllysine | 0.559 | 0.378 |
| dihomolinolenate (20:3n3 or 3n6) | 0.317 | 0.279 |
| mannitol/sorbitol | 0.512 | 0.590 |
| tryptophan betaine | 0.847 | 0.747 |
| 4-vinylphenol sulfate | 0.519 | 0.502 |
| 4-ethylphenyl sulfate | 0.264 | 0.518 |
| thymol sulfate | 0.255 | 0.132 |
| 3-methyladipate | 0.379 | 0.291 |
| pyrraline | 0.099 | 0.000 |
| N6-carboxymethyllysine | 0.610 | 0.459 |
| 2-linoleoyl-GPE (18:2)* | 0.702 | 0.456 |
| 1-oleoyl-GPI (18:1) | 0.482 | 0.595 |
| 1-linoleoyl-GPI* (18:2)* | 0.656 | 0.629 |
| 1-palmitoleoyl-GPE (16:1)* | 0.488 | 0.665 |
| 1-palmitoleoyl-GPI (16:1)* | 0.496 | 0.590 |
| o-cresol sulfate | 0.490 | 0.619 |
| dimethylarginine (ADMA + SDMA) | 0.580 | 0.411 |
| N-acetylserine | 0.774 | 0.659 |
| 1-stearoyl-2-oleoyl-GPE (18:0/18:1) | 0.505 | 0.090 |
| 4-allylphenol sulfate | 0.426 | 0.646 |
| 1-stearoyl-2-arachidonoyl-GPC (18:0/20:4) | 0.791 | 0.832 |
| 1-palmitoyl-2-linoleoyl-GPE (16:0/18:2) | 0.771 | 0.810 |
| 1-stearoyl-2-arachidonoyl-GPS (18:0/20:4) | 0.187 | 0.000 |
| sphinganine-1-phosphate | 0.030 | 0.491 |
| glycosyl-N-nervonoyl-sphingosine (d18:1/24:1)* | 0.654 | 0.519 |
| glycosyl-N-stearoyl-sphingosine (d18:1/18:0) | 0.602 | 0.573 |
| cyclo(leu-pro) | 0.295 | 0.484 |
| cyclo(gly-pro) | 0.186 | 0.625 |
| succinylcarnitine (C4-DC) | 0.798 | 0.617 |
| bilirubin (E,E)* | 0.652 | 0.447 |
| bilirubin (E,Z or Z,E)* | 0.642 | 0.531 |
| N-methylproline | 0.581 | 0.500 |
| beta-cryptoxanthin | 0.549 | 0.591 |
| 5alpha-androstan-3beta,17beta-diol disulfate | 0.880 | 0.846 |
| 5alpha-pregnan-3beta,20alpha-diol disulfate | 0.629 | 0.471 |
| glycocholenate sulfate* | 0.831 | 0.714 |
| taurocholenate sulfate* | 0.721 | 0.820 |
| androstenediol (3beta,17beta) disulfate (1) | 0.934 | 0.944 |
| pregnen-diol disulfate* | 0.900 | 0.789 |
| androstenediol (3beta,17beta) disulfate (2) | 0.936 | 0.862 |
| 21-hydroxypregnenolone disulfate | 0.770 | 0.842 |
| 5alpha-androstan-3alpha,17alpha-diol monosulfate | 0.515 | 0.176 |
| 5alpha-pregnan-3beta,20beta-diol monosulfate (1) | 0.587 | 0.304 |
| 5alpha-pregnan-3beta,20alpha-diol monosulfate (2) | 0.579 | 0.352 |
| 5alpha-pregnan-diol disulfate | 0.195 | 0.000 |
| 5alpha-androstan-3alpha,17beta-diol monosulfate (1) | 0.853 | 0.730 |
| 5alpha-androstan-3beta,17alpha-diol disulfate | 0.583 | 0.718 |
| 5alpha-androstan-3beta,17beta-diol monosulfate (2) | 0.672 | 0.714 |
| androstenediol (3alpha, 17alpha) monosulfate (2) | 0.670 | 0.723 |
| androstenediol (3alpha, 17alpha) monosulfate (3) | 0.841 | 0.877 |
| androstenediol (3beta,17beta) monosulfate (1) | 0.870 | 0.925 |
| androstenediol (3beta,17beta) monosulfate (2) | 0.589 | 0.791 |
| 1-docosahexaenoyl-GPE (22:6)* | 0.586 | 0.788 |
| 2-docosahexaenoyl-GPE (22:6)* | 0.229 | 0.392 |
| pregnenediol sulfate (C21H34O5S)* | 0.860 | 0.722 |
| 2-oleoyl-GPI (18:1)* | 0.392 | 0.371 |
| 2-hydroxyglutarate | 0.662 | 0.321 |
| gamma-CEHC | 0.567 | 0.578 |
| N-acetyl-beta-alanine | 0.420 | 0.000 |
| sphingomyelin (d18:1/18:1, d18:2/18:0) | 0.725 | 0.726 |
| palmitoyl sphingomyelin (d18:1/16:0) | 0.847 | 0.697 |
| cysteine sulfinic acid | 0.000 | 0.493 |
| 3-hydroxyhippurate | 0.379 | 0.039 |
| 16alpha-hydroxy DHEA 3-sulfate | 0.918 | 0.919 |
| pregnenolone sulfate | 0.811 | 0.823 |
| andro steroid monosulfate C19H28O6S (1)* | 0.823 | 0.813 |
| betonicine | 0.690 | 0.285 |
| ergothioneine | 0.693 | 0.662 |
| 1-margaroyl-GPE (17:0)* | 0.397 | 0.430 |
| 1-pentadecanoyl-GPC (15:0)* | 0.445 | 0.292 |
| S-methylmethionine | 0.255 | 0.000 |
| 13-HODE + 9-HODE | 0.157 | 0.562 |
| tridecenedioate (C13:1-DC)* | 0.536 | 0.059 |
| N-acetyl-3-methylhistidine* | 0.343 | 0.000 |
| 4-cholesten-3-one | 0.078 | 0.182 |
| 7-methylurate | 0.570 | 0.538 |
| cinnamoylglycine | 0.573 | 0.337 |
| cis-4-decenoylcarnitine (C10:1) | 0.647 | 0.290 |
| 2S,3R-dihydroxybutyrate | 0.593 | 0.664 |
| (12 or 13)-methylmyristate (a15:0 or i15:0) | 0.170 | 0.239 |
| (16 or 17)-methylstearate (a19:0 or i19:0) | 0.099 | 0.114 |
| 2R,3R-dihydroxybutyrate | 0.746 | 0.381 |
| dexpanthenol | 0.259 | 0.084 |
| alpha-ketoglutaramate* | 0.768 | 0.705 |
| 2,3-dihydroxyisovalerate | 0.444 | 0.603 |
| 3-methylglutaconate | 0.739 | 0.812 |
| 5-(galactosylhydroxy)-lysine | 0.416 | 0.360 |
| cysteinylglycine disulfide* | 0.441 | 0.722 |
| isoursodeoxycholate | 0.577 | 0.605 |
| formiminoglutamate | 0.384 | 0.399 |
| hydantoin-5-propionate | 0.332 | 0.354 |
| sulfate* | 0.658 | 0.482 |
| 4-hydroxy-2-oxoglutaric acid | 0.480 | 0.139 |
| 4-hydroxyglutamate | 0.407 | 0.275 |
| L-urobilin | 0.531 | 0.528 |
| pantoate | 0.347 | 0.153 |
| gamma-carboxyglutamate | 0.744 | 0.601 |
| S-methylcysteine | 0.529 | 0.274 |
| androsterone glucuronide | 0.887 | 0.957 |
| argininate* | 0.298 | 0.217 |
| 2-oxoarginine* | 0.304 | 0.007 |
| cis-4-decenoate (10:1n6)* | 0.374 | 0.312 |
| leukotriene B5 | 0.196 | 0.459 |
| 4-oxo-retinoic acid | 0.489 | 0.713 |
| 1-behenoyl-GPC (22:0) | 0.244 | 0.074 |
| 1-adrenoyl-GPC (22:4)* | 0.787 | 0.805 |
| 1-lignoceroyl-GPC (24:0) | 0.646 | 0.454 |
| 1-nervonoyl-GPC (24:1n9)* | 0.508 | 0.471 |
| 1-(1-enyl-palmitoyl)-GPC (P-16:0)* | 0.731 | 0.682 |
| 1-(1-enyl-oleoyl)-GPC (P-18:1)* | 0.781 | 0.521 |
| 1-(1-enyl-stearoyl)-GPC (P-18:0)* | 0.690 | 0.703 |
| 1-methyl-5-imidazoleacetate | 0.476 | 0.443 |
| glycoursodeoxycholate | 0.711 | 0.572 |
| tauroursodeoxycholate | 0.701 | 0.369 |
| S-methylcysteine sulfoxide | 0.427 | 0.000 |
| (14 or 15)-methylpalmitate (a17:0 or i17:0) | 0.355 | 0.015 |
| eicosanedioate (C20-DC) | 0.480 | 0.457 |
| docosadioate (C22-DC) | 0.275 | 0.270 |
| 16-hydroxypalmitate | 0.445 | 0.049 |
| oleoyl-linoleoyl-glycerol (18:1/18:2) [1] | 0.695 | 0.217 |
| oleoyl-linoleoyl-glycerol (18:1/18:2) [2] | 0.745 | 0.303 |
| 1-(1-enyl-palmitoyl)-GPE (P-16:0)* | 0.588 | 0.502 |
| 1-(1-enyl-stearoyl)-GPE (P-18:0)* | 0.502 | 0.511 |
| 2-stearoyl-GPI (18:0)* | 0.397 | 0.593 |
| alpha-CEHC glucuronide* | 0.794 | 0.404 |
| N-oleoyltaurine | 0.000 | 0.456 |
| linoleoylcarnitine (C18:2)* | 0.220 | 0.517 |
| isoleucylglycine | 0.036 | 0.348 |
| leucylalanine | 0.268 | 0.176 |
| leucylglycine | 0.446 | 0.517 |
| valylleucine | 0.447 | 0.527 |
| N-palmitoyltaurine | 0.000 | 0.423 |
| N-stearoyltaurine | 0.208 | 0.456 |
| 2-O-methylascorbic acid | 0.814 | 0.768 |
| carboxyethyl-GABA | 0.420 | 0.353 |
| beta-citrylglutamate | 0.305 | 0.134 |
| trimethylamine N-oxide | 0.533 | 0.381 |
| N6-methyllysine | 0.975 | 0.985 |
| dihydroferulate | 0.000 | 0.087 |
| imidazole propionate | 0.503 | 0.437 |
| pregnanediol-3-glucuronide | 0.348 | 0.293 |
| alliin | 0.244 | 0.000 |
| phenylalanylglycine | 0.235 | 0.221 |
| valylglycine | 0.051 | 0.364 |
| prolylglycine | 0.345 | 0.077 |
| N-palmitoylglycine | 0.186 | 0.314 |
| mannonate* | 0.855 | 0.645 |
| lanthionine | 0.501 | 0.317 |
| 2-stearoyl-GPE (18:0)* | 0.229 | 0.419 |
| (R)-3-hydroxybutyrylcarnitine | 0.591 | 0.455 |
| N-octanoylglycine | 0.438 | 0.399 |
| N-acetylcarnosine | 0.785 | 0.624 |
| margaroylcarnitine (C17)* | 0.298 | 0.552 |
| histidine betaine (hercynine)* | 0.349 | 0.083 |
| glycohyocholate | 0.648 | 0.612 |
| retinal | 0.339 | 0.011 |
| 2-hydroxydecanoate | 0.000 | 0.090 |
| 3-methyl catechol sulfate (2) | 0.244 | 0.629 |
| 4-methylcatechol sulfate | 0.583 | 0.754 |
| 3-methyl catechol sulfate (1) | 0.497 | 0.620 |
| 3beta-hydroxy-5-cholenoate | 0.584 | 0.800 |
| guaiacol sulfate | 0.422 | 0.609 |
| 2-aminooctanoate | 0.472 | 0.569 |
| gamma-CEHC glucuronide* | 0.687 | 0.623 |
| dimethyl sulfone | 0.323 | 0.628 |
| 2-piperidinone | 0.670 | 0.373 |
| N-acetyl-1-methylhistidine* | 0.729 | 0.533 |
| indolin-2-one | 0.692 | 0.805 |
| 2,8-quinolinediol sulfate | 0.097 | 0.000 |
| 2-aminophenol sulfate | 0.182 | 0.050 |
| 3-acetylphenol sulfate | 0.445 | 0.730 |
| sphingomyelin (d18:1/14:0, d16:1/16:0)* | 0.662 | 0.703 |
| sphingomyelin (d18:2/16:0, d18:1/16:1)* | 0.876 | 0.713 |
| 3-hydroxyadipate | 0.347 | 0.373 |
| 2-hydroxyphytanate* | 0.671 | 0.679 |
| 6-oxopiperidine-2-carboxylate | 0.333 | 0.302 |
| S-allylcysteine | 0.219 | 0.000 |
| N-delta-acetylornithine | 0.735 | 0.609 |
| acisoga | 0.621 | 0.606 |
| 2-aminoheptanoate | 0.646 | 0.465 |
| 1-eicosapentaenoyl-GPE (20:5)* | 0.395 | 0.364 |
| N-formylanthranilic acid | 0.042 | 0.618 |
| N2,N5-diacetylornithine | 0.777 | 0.764 |
| 1H-indole-7-acetic acid | 0.449 | 0.293 |
| 3-methoxytyramine sulfate | 0.354 | 0.362 |
| methionine sulfone | 0.895 | 0.837 |
| 1-linolenoyl-GPC (18:3)* | 0.531 | 0.237 |
| 1-eicosapentaenoyl-GPC (20:5)* | 0.300 | 0.616 |
| 1-eicosenoyl-GPC (20:1)* | 0.586 | 0.493 |
| 1-nonadecanoyl-GPC (19:0) | 0.613 | 0.421 |
| N-acetylalliin | 0.370 | 0.000 |
| 1-dihomo-linolenoyl-GPE (20:3n3 or 6)* | 0.593 | 0.410 |
| 1-(1-enyl-oleoyl)-GPE (P-18:1)* | 0.594 | 0.337 |
| fructosyllysine | 0.471 | 0.226 |
| 1-eicosenoyl-GPE (20:1)* | 0.405 | 0.289 |
| N-methylpipecolate | 0.842 | 0.637 |
| O-sulfo-tyrosine | 0.895 | 0.808 |
| ferulic acid 4-sulfate | 0.219 | 0.230 |
| 3-(3-hydroxyphenyl)propionate sulfate | 0.280 | 0.460 |
| 11-ketoetiocholanolone glucuronide | 0.537 | 0.563 |
| etiocholanolone glucuronide | 0.721 | 0.721 |
| 17alpha-hydroxypregnanolone glucuronide | 0.468 | 0.336 |
| N-acetyltaurine | 0.628 | 0.792 |
| 1-linolenoyl-GPE (18:3)* | 0.509 | 0.248 |
| 1-oleoyl-GPG (18:1)* | 0.615 | 0.577 |
| 1-palmitoyl-GPG (16:0)* | 0.481 | 0.589 |
| 2-oleoyl-GPG (18:1)* | 0.595 | 0.401 |
| 9-hydroxystearate | 0.228 | 0.294 |
| 3-methylglutarylcarnitine (2) | 0.844 | 0.829 |
| methyl glucopyranoside (alpha + beta) | 0.574 | 0.545 |
| 2-keto-3-deoxy-gluconate | 0.635 | 0.741 |
| alpha-CEHC sulfate | 0.836 | 0.676 |
| alpha-CMBHC glucuronide | 0.693 | 0.269 |
| sphingomyelin (d18:2/14:0, d18:1/14:1)* | 0.771 | 0.562 |
| sphingomyelin (d18:1/24:1, d18:2/24:0)* | 0.840 | 0.713 |
| octadecenedioylcarnitine (C18:1-DC)* | 0.355 | 0.430 |
| octadecanedioylcarnitine (C18-DC)* | 0.602 | 0.520 |
| 5alpha-androstan-3alpha,17beta-diol monosulfate (2) | 0.736 | 0.528 |
| myristoleoylcarnitine (C14:1)* | 0.549 | 0.198 |
| N-formylphenylalanine | 0.282 | 0.232 |
| cyclo(pro-val) | 0.421 | 0.543 |
| 4-hydroxychlorothalonil | 0.606 | 0.584 |
| isoeugenol sulfate | 0.266 | 0.480 |
| tyramine O-sulfate | 0.535 | 0.507 |
| 3-hydroxypyridine sulfate | 0.374 | 0.539 |
| 3-hydroxyindolin-2-one sulfate | 0.651 | 0.547 |
| m-tyramine sulfate | 0.000 | 0.154 |
| 4-methylguaiacol sulfate | 0.163 | 0.320 |
| maltol sulfate | 0.292 | 0.082 |
| phenylacetylcarnitine | 0.512 | 0.424 |
| arabonate/xylonate | 0.551 | 0.614 |
| methyl-4-hydroxybenzoate sulfate | 0.325 | 0.339 |
| vanillic alcohol sulfate | 0.166 | 0.400 |
| 4-vinylguaiacol sulfate | 0.000 | 0.259 |
| vanillactate | 0.869 | 0.732 |
| eugenol sulfate | 0.000 | 0.022 |
| pregnanolone/allopregnanolone sulfate | 0.108 | 0.000 |
| 2-methoxyresorcinol sulfate | 0.285 | 0.273 |
| 2-acetamidophenol sulfate | 0.214 | 0.000 |
| p-cresol glucuronide* | 0.495 | 0.350 |
| 6-hydroxyindole sulfate | 0.704 | 0.792 |
| propyl 4-hydroxybenzoate sulfate | 0.264 | 0.294 |
| ethylparaben sulfate | 0.423 | 0.447 |
| umbelliferone sulfate | 0.000 | 0.116 |
| sphingomyelin (d18:1/20:0, d16:1/22:0)* | 0.727 | 0.798 |
| sphingomyelin (d18:1/20:1, d18:2/20:0)* | 0.763 | 0.784 |
| sphingomyelin (d18:1/20:2, d18:2/20:1, d16:1/22:2)* | 0.434 | 0.517 |
| behenoyl sphingomyelin (d18:1/22:0)* | 0.820 | 0.614 |
| sphingomyelin (d18:1/22:1, d18:2/22:0, d16:1/24:1)* | 0.757 | 0.699 |
| sphingomyelin (d18:1/22:2, d18:2/22:1, d16:1/24:2)* | 0.640 | 0.795 |
| lignoceroyl sphingomyelin (d18:1/24:0) | 0.868 | 0.698 |
| sphingomyelin (d17:1/16:0, d18:1/15:0, d16:1/17:0)* | 0.555 | 0.614 |
| dopamine 4-sulfate | 0.000 | 0.073 |
| dopamine 3-O-sulfate | 0.073 | 0.173 |
| 3-hydroxyhexanoate | 0.476 | 0.379 |
| N-carbamoylalanine | 0.420 | 0.212 |
| 3beta-hydroxy-5-cholestenoate | 0.705 | 0.814 |
| 1,2,3-benzenetriol sulfate (2) | 0.422 | 0.513 |
| 3-methoxycatechol sulfate (1) | 0.290 | 0.321 |
| 3-methoxycatechol sulfate (2) | 0.480 | 0.363 |
| N-acetylkynurenine (2) | 0.433 | 0.490 |
| C-glycosyltryptophan | 0.854 | 0.675 |
| arabitol/xylitol | 0.606 | 0.411 |
| N-acetylglucosamine/N-acetylgalactosamine | 0.284 | 0.214 |
| citraconate/glutaconate | 0.488 | 0.698 |
| adipoylcarnitine (C6-DC) | 0.608 | 0.453 |
| 4-hydroxyphenylacetoylcarnitine | 0.512 | 0.422 |
| nonanoylcarnitine (C9) | 0.673 | 0.463 |
| suberoylcarnitine (C8-DC) | 0.522 | 0.503 |
| glycochenodeoxycholate 3-sulfate | 0.782 | 0.556 |
| glycodeoxycholate 3-sulfate | 0.649 | 0.523 |
| taurodeoxycholic acid 3-sulfate | 0.615 | 0.442 |
| trans-3,4-methyleneheptanoate | 0.032 | 0.248 |
| phenol glucuronide | 0.477 | 0.599 |
| linoleoyl ethanolamide | 0.200 | 0.320 |
| cyclo(met-pro) | 0.636 | 0.619 |
| 1,2-dilinoleoyl-GPC (18:2/18:2) | 0.788 | 0.635 |
| 1-stearoyl-2-oleoyl-GPC (18:0/18:1) | 0.493 | 0.216 |
| 1-palmitoyl-2-arachidonoyl-GPC (16:0/20:4n6) | 0.811 | 0.868 |
| 1-palmitoyl-2-docosahexaenoyl-GPC (16:0/22:6) | 0.772 | 0.857 |
| 1-stearoyl-2-docosahexaenoyl-GPC (18:0/22:6) | 0.707 | 0.851 |
| 1-(1-enyl-stearoyl)-2-oleoyl-GPC (P-18:0/18:1) | 0.867 | 0.751 |
| 1-(1-enyl-stearoyl)-2-arachidonoyl-GPC (P-18:0/20:4) | 0.769 | 0.808 |
| 1-(1-enyl-stearoyl)-2-oleoyl-GPE (P-18:0/18:1) | 0.677 | 0.702 |
| sphingomyelin (d18:1/17:0, d17:1/18:0, d19:1/16:0) | 0.614 | 0.556 |
| 1-palmitoyl-2-stearoyl-GPC (16:0/18:0) | 0.700 | 0.615 |
| 2-hydroxybutyrate/2-hydroxyisobutyrate | 0.616 | 0.436 |
| oleate/vaccenate (18:1) | 0.144 | 0.037 |
| 3-hydroxystearate | 0.336 | 0.410 |
| 2-hydroxylaurate | 0.454 | 0.720 |
| 1-palmitoleoylglycerol (16:1)* | 0.246 | 0.353 |
| palmitoyl dihydrosphingomyelin (d18:0/16:0)* | 0.860 | 0.618 |
| tricosanoyl sphingomyelin (d18:1/23:0)* | 0.810 | 0.675 |
| sphingomyelin (d18:2/23:0, d18:1/23:1, d17:1/24:1)* | 0.527 | 0.592 |
| sphingomyelin (d18:2/24:1, d18:1/24:2)* | 0.842 | 0.733 |
| 1-stearoyl-2-linoleoyl-GPE (18:0/18:2)* | 0.682 | 0.491 |
| 1-stearoyl-2-arachidonoyl-GPE (18:0/20:4) | 0.763 | 0.863 |
| 1-stearoyl-2-linoleoyl-GPC (18:0/18:2)* | 0.823 | 0.654 |
| 1-palmitoyl-2-palmitoleoyl-GPC (16:0/16:1)* | 0.735 | 0.653 |
| 1-palmitoyl-2-dihomo-linolenoyl-GPC (16:0/20:3n3 or 6)* | 0.441 | 0.242 |
| 1-palmitoyl-2-eicosapentaenoyl-GPC (16:0/20:5)* | 0.604 | 0.485 |
| 1-palmitoyl-2-arachidonoyl-GPE (16:0/20:4)* | 0.785 | 0.834 |
| 1-palmitoyl-2-docosahexaenoyl-GPE (16:0/22:6)* | 0.877 | 0.844 |
| 1-stearoyl-2-docosahexaenoyl-GPE (18:0/22:6)* | 0.844 | 0.837 |
| 1-palmitoyl-2-arachidonoyl-GPI (16:0/20:4)* | 0.615 | 0.494 |
| 1-stearoyl-2-linoleoyl-GPI (18:0/18:2) | 0.764 | 0.803 |
| 1-palmitoyl-2-palmitoleoyl-GPE (16:0/16:1)* | 0.493 | 0.477 |
| gamma-tocopherol/beta-tocopherol | 0.812 | 0.767 |
| 1-(1-enyl-stearoyl)-2-arachidonoyl-GPE (P-18:0/20:4)* | 0.623 | 0.736 |
| 1-(1-enyl-palmitoyl)-2-docosahexaenoyl-GPE (P-16:0/22:6)* | 0.742 | 0.703 |
| 1-(1-enyl-palmitoyl)-2-arachidonoyl-GPE (P-16:0/20:4)* | 0.661 | 0.519 |
| 1-(1-enyl-oleoyl)-2-linoleoyl-GPE (P-18:1/18:2)* | 0.075 | 0.000 |
| 1-(1-enyl-stearoyl)-2-docosahexaenoyl-GPE (P-18:0/22:6)* | 0.645 | 0.692 |
| 1-(1-enyl-palmitoyl)-2-oleoyl-GPE (P-16:0/18:1)* | 0.463 | 0.359 |
| 1-(1-enyl-palmitoyl)-2-oleoyl-GPC (P-16:0/18:1)* | 0.859 | 0.622 |
| 1-(1-enyl-palmitoyl)-2-docosahexaenoyl-GPC (P-16:0/22:6)* | 0.676 | 0.780 |
| 1-(1-enyl-palmitoyl)-2-linoleoyl-GPC (P-16:0/18:2)* | 0.766 | 0.661 |
| 1-(1-enyl-palmitoyl)-2-arachidonoyl-GPC (P-16:0/20:4)* | 0.805 | 0.856 |
| 1-(1-enyl-stearoyl)-2-docosahexaenoyl-GPC (P-18:0/22:6)* | 0.724 | 0.728 |
| 1-stearyl-2-arachidonoyl-GPC (O-18:0/20:4)* | 0.618 | 0.653 |
| 1-palmityl-2-arachidonoyl-GPC (O-16:0/20:4)* | 0.630 | 0.692 |
| sphingomyelin (d18:1/21:0, d17:1/22:0, d16:1/23:0)* | 0.656 | 0.668 |
| behenoyl dihydrosphingomyelin (d18:0/22:0)* | 0.932 | 0.676 |
| sphingomyelin (d18:0/18:0, d19:0/17:0)* | 0.836 | 0.627 |
| N-palmitoyl-sphinganine (d18:0/16:0) | 0.515 | 0.614 |
| lactosyl-N-palmitoyl-sphingosine (d18:1/16:0) | 0.829 | 0.901 |
| 1-pentadecanoyl-2-linoleoyl-GPC (15:0/18:2)* | 0.798 | 0.731 |
| 1-margaroyl-2-oleoyl-GPC (17:0/18:1)* | 0.363 | 0.254 |
| 1-margaroyl-2-linoleoyl-GPC (17:0/18:2)* | 0.703 | 0.647 |
| myristoyl dihydrosphingomyelin (d18:0/14:0)* | 0.663 | 0.577 |
| 5-hydroxyindole sulfate | 0.595 | 0.713 |
| phenylacetylglutamate | 0.613 | 0.615 |
| 1-stearoyl-2-dihomo-linolenoyl-GPC (18:0/20:3n3 or 6)* | 0.632 | 0.409 |
| palmitoyl-linoleoyl-glycerol (16:0/18:2) [1]* | 0.494 | 0.353 |
| palmitoyl-linoleoyl-glycerol (16:0/18:2) [2]* | 0.797 | 0.278 |
| 1-palmitoyl-2-oleoyl-GPI (16:0/18:1)* | 0.544 | 0.652 |
| 1-stearoyl-2-docosahexaenoyl-GPI (18:0/22:6)* | 0.717 | 0.806 |
| 1-(1-enyl-palmitoyl)-2-linoleoyl-GPE (P-16:0/18:2)* | 0.737 | 0.587 |
| 1-palmitoleoyl-2-linoleoyl-GPC (16:1/18:2)* | 0.407 | 0.068 |
| 1-oleoyl-2-linoleoyl-GPE (18:1/18:2)* | 0.539 | 0.430 |
| 1-pentadecanoyl-2-arachidonoyl-GPC (15:0/20:4)* | 0.729 | 0.679 |
| 1-pentadecanoyl-2-docosahexaenoyl-GPC (15:0/22:6)* | 0.746 | 0.772 |
| 1-margaroyl-2-arachidonoyl-GPC (17:0/20:4)* | 0.760 | 0.871 |
| 1-arachidoyl-2-arachidonoyl-GPC (20:0/20:4)* | 0.772 | 0.806 |
| 1-oleoyl-2-docosahexaenoyl-GPC (18:1/22:6)* | 0.781 | 0.773 |
| 1-linoleoyl-2-arachidonoyl-GPC (18:2/20:4n6)* | 0.741 | 0.768 |
| 1-linoleoyl-2-docosahexaenoyl-GPC (18:2/22:6)* | 0.699 | 0.752 |
| 1-palmityl-2-linoleoyl-GPC (O-16:0/18:2)* | 0.710 | 0.641 |
| 1-(1-enyl-stearoyl)-2-linoleoyl-GPC (P-18:0/18:2)* | 0.846 | 0.730 |
| 1-myristoyl-2-arachidonoyl-GPC (14:0/20:4)* | 0.785 | 0.648 |
| 1-stearoyl-2-docosapentaenoyl-GPC (18:0/22:5n3)* | 0.662 | 0.251 |
| 1-stearoyl-2-docosapentaenoyl-GPC (18:0/22:5n6)* | 0.813 | 0.694 |
| 1-stearoyl-2-adrenoyl-GPC (18:0/22:4)* | 0.725 | 0.296 |
| 1-stearyl-GPC (O-18:0)* | 0.698 | 0.626 |
| 1-oleoyl-2-dihomo-linolenoyl-GPC (18:1/20:3)* | 0.679 | 0.259 |
| 1-stearoyl-2-meadoyl-GPC (18:0/20:3n9)* | 0.561 | 0.176 |
| 1-palmitoyl-2-gamma-linolenoyl-GPC (16:0/18:3n6)* | 0.461 | 0.297 |
| 1-(1-enyl-palmitoyl)-2-palmitoleoyl-GPC (P-16:0/16:1)* | 0.876 | 0.693 |
| 1-(1-enyl-palmitoyl)-2-palmitoyl-GPC (P-16:0/16:0)* | 0.822 | 0.661 |
| 1-stearoyl-2-oleoyl-GPI (18:0/18:1)* | 0.600 | 0.561 |
| 1-stearoyl-2-dihomo-linolenoyl-GPI (18:0/20:3n3 or 6)* | 0.658 | 0.494 |
| 1-stearyl-GPE (O-18:0)* | 0.063 | 0.461 |
| 1,2-dipalmitoyl-GPE (16:0/16:0)* | 0.396 | 0.518 |
| 1-palmitoyl-2-stearoyl-GPE (16:0/18:0)* | 0.080 | 0.248 |
| 1-palmitoyl-2-eicosapentaenoyl-GPE (16:0/20:5)* | 0.622 | 0.464 |
| 1-stearoyl-2-dihomo-linolenoyl-GPE (18:0/20:3n3 or 6)* | 0.799 | 0.566 |
| 1,2-dilinoleoyl-GPE (18:2/18:2)* | 0.261 | 0.189 |
| 1-oleoyl-2-arachidonoyl-GPE (18:1/20:4)* | 0.592 | 0.768 |
| 1-oleoyl-2-docosahexaenoyl-GPE (18:1/22:6)* | 0.709 | 0.736 |
| 1-linoleoyl-2-arachidonoyl-GPE (18:2/20:4)* | 0.523 | 0.312 |
| 1-(1-enyl-stearoyl)-2-linoleoyl-GPE (P-18:0/18:2)* | 0.793 | 0.742 |
| 1-linoleoyl-GPG (18:2)* | 0.649 | 0.561 |
| thioproline | 0.420 | 0.649 |
| palmitoylcholine | 0.180 | 0.476 |
| phenylacetylmethionine | 0.552 | 0.706 |
| 2-methylserine | 0.365 | 0.307 |
| glycocholate glucuronide (1) | 0.615 | 0.599 |
| glycochenodeoxycholate glucuronide (1) | 0.781 | 0.372 |
| glycodeoxycholate glucuronide (1) | 0.288 | 0.361 |
| (S)-3-hydroxybutyrylcarnitine | 0.516 | 0.586 |
| glycosyl-N-palmitoyl-sphingosine (d18:1/16:0) | 0.588 | 0.648 |
| furaneol sulfate | 0.000 | 0.301 |
| ascorbic acid 2-sulfate | 0.559 | 0.814 |
| oleoylcholine | 0.034 | 0.517 |
| arachidonoylcholine | 0.083 | 0.601 |
| caffeic acid sulfate | 0.386 | 0.470 |
| 1-linoleoyl-2-linolenoyl-GPC (18:2/18:3)* | 0.610 | 0.160 |
| 1-palmitoleoyl-2-linolenoyl-GPC (16:1/18:3)* | 0.483 | 0.469 |
| 1-oleoyl-2-docosapentaenoyl-GPC (18:1/22:5n3)* | 0.679 | 0.446 |
| phosphatidylcholine (18:0/20:2, 20:0/18:2)* | 0.734 | 0.561 |
| 1-linoleoyl-2-docosapentaenyol-GPC (18:2/22:5n3)* | 0.645 | 0.379 |
| 1-(1-enyl-oleoyl)-2-docosahexaenoyl-GPE (P-18:1/22:6)* | 0.650 | 0.678 |
| hexadecadienoate (16:2n6) | 0.311 | 0.052 |
| 1-linoleoyl-2-eicosapentaenoyl-GPC (18:2/20:5)* | 0.356 | 0.155 |
| palmitoleoylcarnitine (C16:1)* | 0.551 | 0.302 |
| pimeloylcarnitine/3-methyladipoylcarnitine (C7-DC) | 0.688 | 0.599 |
| 3-methylglutarate/2-methylglutarate | 0.317 | 0.365 |
| 4-hydroxyphenylacetylglutamine | 0.753 | 0.681 |
| 2,3-dihydroxy-2-methylbutyrate | 0.453 | 0.376 |
| 2'-O-methylcytidine | 0.364 | 0.730 |
| 2'-O-methyluridine | 0.492 | 0.647 |
| gamma-glutamyl-alpha-lysine | 0.349 | 0.033 |
| ferulylglycine (1) | 0.000 | 0.000 |
| ferulylglycine (2) | 0.098 | 0.007 |
| palmitoyl-oleoyl-glycerol (16:0/18:1) [1]* | 0.591 | 0.000 |
| palmitoyl-oleoyl-glycerol (16:0/18:1) [2]* | 0.583 | 0.025 |
| oleoyl-oleoyl-glycerol (18:1/18:1) [1]* | 0.441 | 0.000 |
| oleoyl-oleoyl-glycerol (18:1/18:1) [2]* | 0.597 | 0.250 |
| linoleoyl-arachidonoyl-glycerol (18:2/20:4) [2]* | 0.789 | 0.621 |
| palmitoyl-arachidonoyl-glycerol (16:0/20:4) [1]* | 0.368 | 0.145 |
| palmitoyl-arachidonoyl-glycerol (16:0/20:4) [2]* | 0.349 | 0.309 |
| linoleoyl-linolenoyl-glycerol (18:2/18:3) [1]* | 0.544 | 0.278 |
| linoleoyl-linolenoyl-glycerol (18:2/18:3) [2]* | 0.609 | 0.346 |
| linoleoyl-docosahexaenoyl-glycerol (18:2/22:6) [1]* | 0.574 | 0.635 |
| linoleoyl-docosahexaenoyl-glycerol (18:2/22:6) [2]* | 0.656 | 0.571 |
| palmitoleoyl-linoleoyl-glycerol (16:1/18:2) [1]* | 0.528 | 0.167 |
| diacylglycerol (14:0/18:1, 16:0/16:1) [2]* | 0.112 | 0.236 |
| oleoyl-arachidonoyl-glycerol (18:1/20:4) [1]* | 0.552 | 0.480 |
| oleoyl-arachidonoyl-glycerol (18:1/20:4) [2]* | 0.644 | 0.673 |
| palmitoyl-linolenoyl-glycerol (16:0/18:3) [2]* | 0.626 | 0.233 |
| diacylglycerol (16:1/18:2 [2], 16:0/18:3 [1])* | 0.760 | 0.394 |
| linoleoyl-linoleoyl-glycerol (18:2/18:2) [1]* | 0.579 | 0.179 |
| linoleoyl-linoleoyl-glycerol (18:2/18:2) [2]* | 0.730 | 0.322 |
| stearoyl-arachidonoyl-glycerol (18:0/20:4) [1]* | 0.375 | 0.627 |
| stearoyl-arachidonoyl-glycerol (18:0/20:4) [2]* | 0.504 | 0.265 |
| perfluorooctanesulfonate (PFOS) | 0.921 | 0.795 |
| 1-palmityl-GPE (O-16:0)* | 0.146 | 0.329 |
| 1-palmityl-2-stearoyl-GPC (O-16:0/18:0)* | 0.857 | 0.640 |
| 1-palmityl-2-palmitoyl-GPC (O-16:0/16:0)* | 0.845 | 0.795 |
| 1-stearyl-2-docosapentaenoyl-GPC (O-18:0/22:5n3)* | 0.760 | 0.849 |
| 1-stearyl-2-linoleoyl-GPC (O-18:0/18:2)* | 0.799 | 0.676 |
| 1-stearoyl-2-docosapentaenoyl-GPE (18:0/22:5n3)* | 0.489 | 0.579 |
| 1-stearoyl-2-docosapentaenoyl-GPE (18:0/22:5n6)* | 0.867 | 0.776 |
| 1-stearoyl-2-adrenoyl-GPE (18:0/22:4)* | 0.757 | 0.589 |
| 1-(1-enyl-stearoyl)-2-docosapentaenoyl-GPE (P-18:0/22:5n3)* | 0.725 | 0.596 |
| N-palmitoyl-sphingadienine (d18:2/16:0)* | 0.731 | 0.507 |
| lactosyl-N-nervonoyl-sphingosine (d18:1/24:1)* | 0.869 | 0.930 |
| lactosyl-N-behenoyl-sphingosine (d18:1/22:0)* | 0.741 | 0.580 |
| N-behenoyl-sphingadienine (d18:2/22:0)* | 0.498 | 0.673 |
| glycosyl-N-behenoyl-sphingadienine (d18:2/22:0)* | 0.883 | 0.814 |
| N-stearoyl-sphingadienine (d18:2/18:0)* | 0.387 | 0.505 |
| 2-hydroxybehenate | 0.239 | 0.260 |
| 2-hydroxynervonate* | 0.340 | 0.281 |
| N-palmitoylserine | 0.214 | 0.445 |
| N-oleoylserine | 0.218 | 0.696 |
| sphingadienine | 0.014 | 0.295 |
| palmitoleoyl-arachidonoyl-glycerol (16:1/20:4) [2]* | 0.596 | 0.421 |
| myristoyl-linoleoyl-glycerol (14:0/18:2) [1]* | 0.398 | 0.347 |
| myristoyl-linoleoyl-glycerol (14:0/18:2) [2]* | 0.589 | 0.171 |
| 1-stearoyl-2-(hydroxylinoleoyl)-GPC (18:0/18:2(OH))* | 0.520 | 0.458 |
| 1-palmitoyl-2-(hydroxylinoleoyl)-GPC (16:0/18:2(OH))* | 0.645 | 0.765 |
| ceramide (d16:1/24:1, d18:1/22:1)* | 0.422 | 0.413 |
| N-palmitoyl-heptadecasphingosine (d17:1/16:0)* | 0.524 | 0.229 |
| ceramide (d18:1/14:0, d16:1/16:0)* | 0.846 | 0.426 |
| ceramide (d18:1/17:0, d17:1/18:0)* | 0.446 | 0.597 |
| ceramide (d18:2/24:1, d18:1/24:2)* | 0.704 | 0.725 |
| glycosyl ceramide (d18:2/24:1, d18:1/24:2)* | 0.810 | 0.806 |
| glycosyl-N-tricosanoyl-sphingadienine (d18:2/23:0)* | 0.583 | 0.570 |
| glycosyl-N-(2-hydroxynervonoyl)-sphingosine (d18:1/24:1(2OH))* | 0.551 | 0.619 |
| ceramide (d18:1/20:0, d16:1/22:0, d20:1/18:0)* | 0.710 | 0.601 |
| linoleoylcholine* | 0.041 | 0.264 |
| 1-cerotoyl-GPC (26:0)* | 0.000 | 0.248 |
| sphingomyelin (d18:0/20:0, d16:0/22:0)* | 0.916 | 0.706 |
| sphingomyelin (d18:1/19:0, d19:1/18:0)* | 0.734 | 0.778 |
| sphingomyelin (d18:2/18:1)* | 0.747 | 0.608 |
| sphingomyelin (d18:2/24:2)* | 0.755 | 0.806 |
| sphingomyelin (d18:2/21:0, d16:2/23:0)* | 0.801 | 0.788 |
| sphingomyelin (d18:2/23:1)* | 0.754 | 0.736 |
| sphingomyelin (d18:1/25:0, d19:0/24:1, d20:1/23:0, d19:1/24:0)* | 0.674 | 0.553 |
| sphingomyelin (d17:2/16:0, d18:2/15:0)* | 0.734 | 0.727 |
| linolenoylcarnitine (C18:3)* | 0.269 | 0.251 |
| behenoylcarnitine (C22)* | 0.299 | 0.498 |
| arachidoylcarnitine (C20)* | 0.672 | 0.664 |
| lignoceroylcarnitine (C24)* | 0.578 | 0.668 |
| cerotoylcarnitine (C26)* | 0.705 | 0.783 |
| ximenoylcarnitine (C26:1)* | 0.657 | 0.800 |
| arachidonoylcarnitine (C20:4) | 0.323 | 0.706 |
| eicosenoylcarnitine (C20:1)* | 0.265 | 0.547 |
| dihomo-linoleoylcarnitine (C20:2)* | 0.394 | 0.611 |
| dihomo-linolenoylcarnitine (C20:3n3 or 6)* | 0.398 | 0.694 |
| docosahexaenoylcarnitine (C22:6)* | 0.519 | 0.514 |
| nervonoylcarnitine (C24:1)* | 0.556 | 0.443 |
| adrenoylcarnitine (C22:4)* | 0.201 | 0.672 |
| docosapentaenoylcarnitine (C22:5n3)* | 0.336 | 0.621 |
| glycosyl ceramide (d18:1/20:0, d16:1/22:0)* | 0.670 | 0.496 |
| 1-lignoceroyl-2-arachidonoyl-GPC (24:0/20:4)* | 0.536 | 0.356 |
| N,N,N-trimethyl-5-aminovalerate | 0.550 | 0.634 |
| ethyl alpha-glucopyranoside | 0.701 | 0.463 |
| carotene diol (1) | 0.870 | 0.811 |
| carotene diol (2) | 0.840 | 0.804 |
| carotene diol (3) | 0.636 | 0.619 |
| cortolone glucuronide (1) | 0.767 | 0.476 |
| 1-oleyl-2-linoleoyl-GPC (O-18:1/18:2)* | 0.648 | 0.577 |
| 1-palmitoyl-2-pentadecanoyl-GPC (16:0/15:0)* | 0.425 | 0.449 |
| (N(1) + N(8))-acetylspermidine | 0.501 | 0.581 |
| 5-dodecenoylcarnitine (C12:1) | 0.610 | 0.148 |
| hydroxy-CMPF* | 0.867 | 0.869 |
| 3-hydroxypalmitoylcarnitine | 0.232 | 0.532 |
| 3-hydroxyoleoylcarnitine | 0.152 | 0.430 |
| 3-hydroxyphenylacetoylglutamine | 0.220 | 0.399 |
| trans-2-hexenoylglycine | 0.052 | 0.155 |
| 2-hydroxyarachidate* | 0.161 | 0.129 |
| 3-hydroxyarachidate* | 0.118 | 0.308 |
| N-stearoylserine* | 0.114 | 0.185 |
| 3-hydroxyoleate* | 0.377 | 0.384 |
| lyxonate | 0.169 | 0.740 |
| dodecenedioate (C12:1-DC)* | 0.360 | 0.234 |
| hexadecenedioate (C16:1-DC)* | 0.570 | 0.440 |
| octadecenedioate (C18:1-DC) | 0.237 | 0.516 |
| heptenedioate (C7:1-DC)* | 0.570 | 0.194 |
| octadecadienedioate (C18:2-DC)* | 0.115 | 0.504 |
| glucuronide of C12H22O4 (1)* | 0.000 | 0.000 |
| glucuronide of C12H22O4 (2)* | 0.161 | 0.173 |
| glucuronide of C14H26O4 (1)* | 0.129 | 0.000 |
| glucuronide of C10H18O2 (1)* | 0.397 | 0.233 |
| 3-carboxy-4-methyl-5-pentyl-2-furanpropionate (3-CMPFP)** | 0.710 | 0.540 |
| glucuronide of C14H26O4 (2)* | 0.406 | 0.183 |
| glucuronide of C10H18O2 (7)* | 0.540 | 0.085 |
| glucuronide of C10H18O2 (8)* | 0.468 | 0.000 |
| 3-hydroxystachydrine* | 0.562 | 0.316 |
| N-acetyl-2-aminooctanoate* | 0.779 | 0.570 |
| hydroxyasparagine** | 0.826 | 0.725 |
| perfluorooctanoate (PFOA) | 0.927 | 0.857 |
| 3-hydroxybutyroylglycine** | 0.439 | 0.535 |
| glyco-beta-muricholate** | 0.746 | 0.722 |
| N-methylhydroxyproline** | 0.474 | 0.696 |
| N,N,N-trimethyl-alanylproline betaine (TMAP) | 0.604 | 0.282 |
| 3-formylindole | 0.693 | 0.469 |
| 1-(1-enyl-oleoyl)-2-docosahexaenoyl-GPC (P-18:1/22:6)* | 0.639 | 0.584 |
| gamma-glutamylcitrulline* | 0.279 | 0.619 |
| glycine conjugate of C10H12O2* | 0.392 | 0.420 |
| glycine conjugate of C10H14O2 (1)* | 0.308 | 0.608 |
| glutamine conjugate of C7H12O2* | 0.391 | 0.307 |
| glutamine conjugate of C6H10O2 (1)* | 0.503 | 0.374 |
| glutamine conjugate of C6H10O2 (2)* | 0.464 | 0.419 |
| dihydroferulic acid sulfate | 0.286 | 0.261 |
| sphingomyelin (d17:1/14:0, d16:1/15:0)* | 0.595 | 0.494 |
| tetradecadienoate (14:2)* | 0.469 | 0.211 |
| N-carbamoylvaline | 0.385 | 0.258 |
| 8-methoxykynurenate | 0.738 | 0.411 |
| 3-amino-2-piperidone | 0.713 | 0.553 |
| N,N-dimethylalanine | 0.703 | 0.778 |
| 3-indoleglyoxylic acid | 0.567 | 0.535 |
| 2,2'-methylenebis(6-tert-butyl-p-cresol) | 0.000 | 0.000 |
| ethyl beta-glucopyranoside | 0.599 | 0.570 |
| 2-hydroxysebacate | 0.000 | 0.075 |
| ascorbic acid 3-sulfate* | 0.557 | 0.821 |
| 3-hydroxyhippurate sulfate | 0.237 | 0.553 |
| 6-bromotryptophan | 0.806 | 0.648 |
| delta-CEHC | 0.535 | 0.409 |
| N6,N6-dimethyllysine | 0.876 | 0.940 |
| dodecadienoate (12:2)* | 0.374 | 0.133 |
| indoleacetoylcarnitine* | 0.585 | 0.581 |
| delta-CEHC glucuronide | 0.594 | 0.439 |
| N-acetyl-isoputreanine | 0.803 | 0.792 |
| glucuronide of piperine metabolite C17H21NO3 (3)* | 0.513 | 0.346 |
| glucuronide of piperine metabolite C17H21NO3 (4)* | 0.703 | 0.369 |
| glucuronide of piperine metabolite C17H21NO3 (5)* | 0.715 | 0.408 |
| sulfate of piperine metabolite C16H19NO3 (2)* | 0.670 | 0.292 |
| sulfate of piperine metabolite C16H19NO3 (3)* | 0.696 | 0.274 |
| sulfate of piperine metabolite C18H21NO3 (1)* | 0.522 | 0.264 |
| sulfate of piperine metabolite C18H21NO3 (3)* | 0.482 | 0.339 |
| 5-hydroxyindole glucuronide | 0.557 | 0.760 |
| N-acetylhomocitrulline | 0.651 | 0.305 |
| N-acetyltheanine | 0.416 | 0.209 |
| 2-naphthol sulfate | 0.708 | 0.630 |
| (2,4 or 2,5)-dimethylphenol sulfate | 0.257 | 0.439 |
| 4-ethylcatechol sulfate | 0.373 | 0.701 |
| 11beta-hydroxyandrosterone glucuronide | 0.829 | 0.792 |
| 11beta-hydroxyetiocholanolone glucuronide* | 0.665 | 0.443 |
| N2-acetyl,N6,N6-dimethyllysine | 0.951 | 0.948 |
| N2-acetyl,N6-methyllysine | 0.867 | 0.838 |
| cholic acid glucuronide | 0.596 | 0.598 |
| deoxycholic acid glucuronide | 0.828 | 0.736 |
| 4-allylcatechol sulfate | 0.393 | 0.395 |
| 3-hydroxypyridine glucuronide | 0.266 | 0.622 |
| methyl vanillate sulfate | 0.037 | 0.427 |
| 4-methylhexanoylglutamine | 0.302 | 0.289 |
| 1-nonadecenoyl-GPC (19:1)* | 0.325 | 0.350 |
| 1-nisinoyl-GPC (24:6)* | 0.063 | 0.225 |
| glycoursodeoxycholic acid sulfate (1) | 0.784 | 0.486 |
| dihydrocaffeate sulfate (2) | 0.537 | 0.407 |
| lithocholate sulfate (1) | 0.463 | 0.534 |
| 3-hydroxyhexanoylcarnitine (1) | 0.480 | 0.531 |
| 2-ketocaprylate | 0.293 | 0.218 |
| N,N-dimethyl-5-aminovalerate | 0.324 | 0.557 |
| tauroursodeoxycholic acid sulfate (1) | 0.756 | 0.373 |
| 3-ethylcatechol sulfate (1) | 0.308 | 0.462 |
| 3-ethylcatechol sulfate (2) | 0.142 | 0.244 |
| 4-acetylcatechol sulfate (1) | 0.307 | 0.618 |
| 2,3-dihydroxy-5-methylthio-4-pentenoate (DMTPA)* | 0.833 | 0.648 |
| 5-hydroxy-2-methylpyridine sulfate | 0.075 | 0.350 |
| 3-hydroxy-2-methylpyridine sulfate | 0.042 | 0.442 |
| cyclo(pro-sulfo-tyr)* | 0.262 | 0.594 |
| hydroxypalmitoyl sphingomyelin (d18:1/16:0(OH))** | 0.831 | 0.924 |
| cyclo(pro-arg)* | 0.543 | 0.478 |
| taurochenodeoxycholic acid 3-sulfate | 0.708 | 0.730 |
| tetradecadienedioate (C14:2-DC)* | 0.674 | 0.194 |
| pregnenetriol sulfate* | 0.870 | 0.892 |
| pregnenetriol disulfate* | 0.795 | 0.887 |
| eicosenedioate (C20:1-DC)* | 0.671 | 0.736 |
| hydroxy-N6,N6,N6-trimethyllysine* | 0.780 | 0.394 |
| undecenoylcarnitine (C11:1) | 0.544 | 0.375 |
| 3-decenoylcarnitine | 0.492 | 0.390 |
| levulinoylcarnitine | 0.388 | 0.232 |
| 3-hydroxydecanoylcarnitine | 0.514 | 0.285 |
| palmitoyl-sphingosine-phosphoethanolamine (d18:1/16:0) | 0.804 | 0.645 |
| picolinoylglycine | 0.539 | 0.400 |
| 4-vinylcatechol sulfate | 0.259 | 0.394 |
| 4-vinylguaiacol glucuronide | 0.061 | 0.000 |
| succinoyltaurine | 0.578 | 0.715 |
| leucylhydroxyproline* | 0.328 | 0.642 |
| isoleucylhydroxyproline* | 0.192 | 0.634 |
| 3,5-dichloro-2,6-dihydroxybenzoic acid | 0.828 | 0.690 |
| (S)-a-amino-omega-caprolactam | 0.732 | 0.715 |
| metabolonic lactone sulfate | 0.886 | 0.821 |
| vanilloylglycine | 0.012 | 0.446 |
| 2-hydroxy-4-(methylthio)butanoic acid | 0.548 | 0.402 |
| branched chain 14:0 dicarboxylic acid** | 0.429 | 0.318 |
| (2-butoxyethoxy)acetic acid | 0.264 | 0.419 |
| pentose acid* | 0.283 | 0.306 |
| N-succinyl-phenylalanine | 0.286 | 0.314 |
| 1-methyl-5-imidazolelactate | 0.516 | 0.192 |
| (2 or 3)-decenoate (10:1n7 or n8) | 0.347 | 0.000 |
| S-carboxyethylcysteine | 0.609 | 0.012 |
| isoursodeoxycholate sulfate (1) | 0.700 | 0.106 |
| chenodeoxycholic acid sulfate (1) | 0.538 | 0.415 |
| branched-chain, straight-chain, or cyclopropyl 10:1 fatty acid (1)* | 0.576 | 0.277 |
| carnitine of C10H14O2 (5)* | 0.697 | 0.406 |
| 3-bromo-5-chloro-2,6-dihydroxybenzoic acid* | 0.768 | 0.708 |
| branched-chain, straight-chain, or cyclopropyl 12:1 fatty acid* | 0.467 | 0.090 |
| decadienedioic acid (C10:2-DC)** | 0.734 | 0.613 |
| deoxycholic acid 12-sulfate* | 0.485 | 0.516 |
| GlcNAc sulfate conjugate of C21H34O2 steroid** | 0.618 | 0.395 |
| cis-3,4-methyleneheptanoate | 0.486 | 0.536 |
| cis-3,4-methyleneheptanoylcarnitine | 0.477 | 0.501 |
| N-acetyl-2-aminoadipate | 0.422 | 0.285 |
| 2-methoxyhydroquinone sulfate (1) | 0.287 | 0.203 |
| 2-methoxyhydroquinone sulfate (2) | 0.232 | 0.147 |
| 2,4-di-tert-butylphenol | 0.589 | 0.504 |
| 3-hydroxyoctanoylcarnitine (1) | 0.495 | 0.288 |
| 3-hydroxyoctanoylcarnitine (2) | 0.529 | 0.338 |
| cis-3,4-methyleneheptanoylglycine | 0.507 | 0.360 |
| bilirubin degradation product, C16H18N2O5 (1)** | 0.663 | 0.483 |
| bilirubin degradation product, C16H18N2O5 (2)** | 0.616 | 0.535 |
| bilirubin degradation product, C17H18N2O4 (1)** | 0.627 | 0.413 |
| bilirubin degradation product, C17H18N2O4 (2)** | 0.580 | 0.480 |
| bilirubin degradation product, C17H18N2O4 (3)** | 0.537 | 0.456 |
| bilirubin degradation product, C17H20N2O5 (1)** | 0.526 | 0.473 |
| bilirubin degradation product, C17H20N2O5 (2)** | 0.531 | 0.514 |
| tetrahydrocortisol glucuronide | 0.757 | 0.406 |
| bilirubin degradation product, C16H18N2O5 (3)** | 0.544 | 0.525 |
| bilirubin degradation product, C16H18N2O5 (4)** | 0.566 | 0.520 |
| N,N-dimethyl-pro-pro | 0.709 | 0.554 |
| oxindolylalanine | 0.549 | 0.421 |
| tetrahydrocortisone glucuronide (5) | 0.751 | 0.685 |
| perfluorohexanesulfonate (PFHxS) | 0.979 | 0.901 |
| menthol glucuronide | 0.378 | 0.373 |
| N-lactoyl isoleucine | 0.155 | 0.407 |
| N-lactoyl leucine | 0.274 | 0.453 |
| N-lactoyl phenylalanine | 0.387 | 0.444 |
| N-lactoyl tyrosine | 0.247 | 0.440 |
| N-lactoyl valine | 0.253 | 0.468 |
| 1-oleyl-GPC (O-18:1)* | 0.698 | 0.671 |

**Table S2: Intraclass correlation coefficients for proteins by treatment arms.**

| **Aptamer Name** | **Target Full Name** | **placebo ICC** | **famotidine ICC** |
| --- | --- | --- | --- |
| seq.10000.28 | Beta-crystallin B2 | 0.600 | 0.965 |
| seq.10001.7 | RAF proto-oncogene serine/threonine-protein kinase | 0.306 | 0.000 |
| seq.10003.15 | Zinc finger protein 41 | 0.000 | 0.616 |
| seq.10006.25 | ETS domain-containing protein Elk-1 | 0.422 | 0.486 |
| seq.10008.43 | Guanylyl cyclase-activating protein 1 | 0.335 | 0.000 |
| seq.10010.10 | Beclin-1 | 0.641 | 0.607 |
| seq.10011.65 | Inositol polyphosphate 5-phosphatase OCRL-1 | 0.536 | 0.453 |
| seq.10012.5 | SAM pointed domain-containing Ets transcription factor | 0.201 | 0.482 |
| seq.10014.31 | Zinc finger protein SNAI2 | 0.975 | 0.851 |
| seq.10015.119 | Voltage-gated potassium channel subunit beta-2 | 0.580 | 0.753 |
| seq.10022.207 | DNA polymerase eta | 0.187 | 0.683 |
| seq.10023.32 | Vitamin D3 receptor | 0.406 | 0.673 |
| seq.10024.44 | 4-hydroxy-2-oxoglutarate aldolase, mitochondrial | 0.759 | 0.869 |
| seq.10025.1 | Dihydrolipoyl dehydrogenase, mitochondrial | 0.775 | 0.510 |
| seq.10030.8 | Adenine DNA glycosylase | 0.331 | 0.441 |
| seq.10035.6 | Dual specificity protein phosphatase 4 | 0.378 | 0.632 |
| seq.10036.201 | Zinc fingers and homeoboxes protein 3 | 0.505 | 0.475 |
| seq.10037.98 | Sialic acid-binding Ig-like lectin 12:Ig-like V-type 2 domain, Isoform long | 0.823 | 0.866 |
| seq.10039.32 | Purine nucleoside phosphorylase | 0.239 | 0.000 |
| seq.10040.63 | Tumor protein 63 | 0.431 | 0.817 |
| seq.10041.3 | Hepatocyte nuclear factor 4-alpha | 0.789 | 0.782 |
| seq.10042.8 | Serine/threonine-protein kinase Sgk3 | 0.886 | 0.000 |
| seq.10043.31 | Bromodomain-containing protein 4 | 0.364 | 0.580 |
| seq.10044.12 | Protein Wnt-10a | 0.282 | 0.000 |
| seq.10045.47 | Cullin-3 | 0.206 | 0.152 |
| seq.10046.55 | Baculoviral IAP repeat-containing protein 2 | 0.892 | 0.945 |
| seq.10047.12 | Neutrophil cytosol factor 2 | 0.000 | 0.284 |
| seq.10048.7 | Core-binding factor subunit beta | 0.856 | 0.446 |
| seq.10049.112 | Telomeric repeat-binding factor 1 | 0.224 | 0.517 |
| seq.10053.5 | Integrin-linked protein kinase | 0.221 | 0.000 |
| seq.10054.3 | Gigaxonin | 0.820 | 0.729 |
| seq.10056.5 | Forkhead box protein M1 | 0.773 | 0.374 |
| seq.10058.1 | UV excision repair protein RAD23 homolog A | 0.193 | 0.064 |
| seq.10063.10 | E3 ubiquitin-protein ligase FANCL | 0.633 | 0.811 |
| seq.10064.12 | Putative hydrolase RBBP9 | 0.466 | 0.202 |
| seq.10069.2 | Peptidyl-prolyl cis-trans isomerase NIMA-interacting 1 | 0.523 | 0.303 |
| seq.10070.22 | 1-phosphatidylinositol 4,5-bisphosphate phosphodiesterase gamma-2 | 0.134 | 0.012 |
| seq.10073.22 | Tyrosyl-DNA phosphodiesterase 1 | 0.259 | 0.228 |
| seq.10074.128 | Bromodomain-containing protein 2 | 0.761 | 0.181 |
| seq.10075.75 | Acyl-CoA-binding domain-containing protein 6 | 0.051 | 0.000 |
| seq.10076.1 | AP-4 complex subunit mu-1 | 0.360 | 0.000 |
| seq.10078.5 | BAG family molecular chaperone regulator 3 | 0.612 | 0.237 |
| seq.10080.9 | Translation initiation factor eIF-2B subunit alpha | 0.229 | 0.000 |
| seq.10081.17 | DNA-binding protein SATB2 | 0.911 | 0.540 |
| seq.10082.251 | Neurofilament light polypeptide | 0.660 | 0.656 |
| seq.10085.25 | Steroidogenic acute regulatory protein, mitochondrial | 0.119 | 0.571 |
| seq.10086.39 | Cystathionine beta-synthase | 0.956 | 0.868 |
| seq.10087.10 | Alpha-crystallin A chain | 0.517 | 0.753 |
| seq.10088.37 | Adenine phosphoribosyltransferase | 0.000 | 0.000 |
| seq.10089.7 | N-acetylserotonin O-methyltransferase-like protein | 0.622 | 0.732 |
| seq.10336.3 | E3 ubiquitin-protein ligase CHIP | 0.637 | 0.020 |
| seq.10339.48 | Gamma-enolase | 0.410 | 0.270 |
| seq.10342.55 | E3 SUMO-protein ligase PIAS4 | 0.258 | 0.732 |
| seq.10344.334 | Interleukin-10 receptor subunit alpha | 0.542 | 0.373 |
| seq.10346.5 | Signal transducer and activator of transcription 3 | 0.010 | 0.000 |
| seq.10351.51 | Interferon regulatory factor 1 | 0.893 | 0.877 |
| seq.10354.57 | Signal transducer and activator of transcription 3 | 0.230 | 0.000 |
| seq.10356.21 | Transcription factor AP-1 | 0.185 | 0.483 |
| seq.10361.25 | 2'-5'-oligoadenylate synthase 1 | 0.970 | 0.273 |
| seq.10362.35 | Myc proto-oncogene protein | 0.943 | 0.650 |
| seq.10363.13 | Mothers against decapentaplegic homolog 3 | 0.175 | 0.000 |
| seq.10364.6 | Mothers against decapentaplegic homolog 2 | 0.032 | 0.000 |
| seq.10365.132 | Interleukin-23 | 0.815 | 0.681 |
| seq.10366.11 | Platelet-derived growth factor receptor alpha | 0.974 | 0.753 |
| seq.10367.62 | Interleukin-12 | 0.597 | 0.547 |
| seq.10370.21 | Signal transducer and activator of transcription 1-alpha/beta | 0.436 | 0.000 |
| seq.10372.18 | Signal transducer and activator of transcription 6 | 0.000 | 0.000 |
| seq.10382.1 | Angiopoietin-related protein 3 | 0.513 | 0.597 |
| seq.10390.21 | E3 ubiquitin-protein ligase ZNRF3 | 0.849 | 0.827 |
| seq.10391.1 | Angiopoietin-related protein 3 | 0.606 | 0.813 |
| seq.10396.6 | Induced myeloid leukemia cell differentiation protein Mcl-1 | 0.827 | 0.296 |
| seq.10398.110 | Ankyrin repeat domain-containing protein 1 | 0.433 | 0.295 |
| seq.10418.36 | Syntaxin-12 | 0.317 | 0.000 |
| seq.10419.1 | Scavenger receptor class A member 5 | 0.539 | 0.417 |
| seq.10420.30 | Fas apoptotic inhibitory molecule 1 | 0.000 | 0.000 |
| seq.10424.31 | Neural proliferation differentiation and control protein 1 | 0.495 | 0.288 |
| seq.10425.3 | Beta-1,4-galactosyltransferase 5 | 0.530 | 0.333 |
| seq.10426.21 | Golgi SNAP receptor complex member 2 | 0.178 | 0.000 |
| seq.10428.1 | Killer cell immunoglobulin-like receptor 2DS2 | 0.821 | 0.873 |
| seq.10430.31 | 4F2 cell-surface antigen heavy chain:Cytoplasmic domain | 0.796 | 0.921 |
| seq.10432.3 | Uncharacterized protein KIAA1644 | 0.839 | 0.000 |
| seq.10435.2 | N(4)-(beta-N-acetylglucosaminyl)-L-asparaginase | 0.476 | 0.243 |
| seq.10438.19 | Granulocyte-macrophage colony-stimulating factor receptor subunit alpha | 0.737 | 0.600 |
| seq.10439.57 | Alpha-amylase 2B | 0.667 | 0.596 |
| seq.10440.26 | CXADR-like membrane protein:Extracellular domain | 0.661 | 0.670 |
| seq.10442.1 | Transmembrane protein 190 | 0.828 | 0.855 |
| seq.10445.20 | Apolipoprotein M | 0.041 | 0.322 |
| seq.10447.18 | Polyadenylate-binding protein 3 | 0.109 | 0.000 |
| seq.10449.31 | Protein shisa-2 homolog | 0.492 | 0.496 |
| seq.10451.11 | Nucleobindin-1 | 0.258 | 0.134 |
| seq.10453.7 | Carcinoembryonic antigen-related cell adhesion molecule 20 | 0.976 | 0.857 |
| seq.10454.99 | DnaJ homolog subfamily C member 16 | 0.384 | 0.000 |
| seq.10455.196 | Interleukin-31 | 0.394 | 0.691 |
| seq.10457.3 | Interleukin-18 receptor accessory protein | 0.918 | 0.393 |
| seq.10460.1 | Chitotriosidase-1 | 0.888 | 0.915 |
| seq.10462.14 | Insulin-like peptide INSL5 | 0.493 | 0.636 |
| seq.10463.23 | Intestinal-type alkaline phosphatase | 0.246 | 0.496 |
| seq.10464.6 | Anthrax toxin receptor 1 | 0.621 | 0.490 |
| seq.10470.34 | Thioredoxin domain-containing protein 11:C-term | 0.103 | 0.000 |
| seq.10472.53 | Tumor necrosis factor receptor superfamily member 14 | 0.577 | 0.508 |
| seq.10473.2 | Trefoil factor 2 | 0.863 | 0.604 |
| seq.10479.18 | Stromelysin-2 | 0.587 | 0.486 |
| seq.10480.33 | CD59 glycoprotein | 0.627 | 0.443 |
| seq.10485.56 | Melanoma-associated antigen 4 | 0.008 | 0.000 |
| seq.10490.3 | Dolichyl-diphosphooligosaccharide--protein glycosyltransferase subunit 1:Cytoplasmic domain | 0.488 | 0.166 |
| seq.10496.11 | Calcium-activated chloride channel regulator 1 | 0.889 | 0.920 |
| seq.10499.1 | Transmembrane protein 106A | 0.867 | 0.777 |
| seq.10505.12 | E3 ubiquitin-protein ligase RNF43 | 0.913 | 0.541 |
| seq.10506.53 | Membrane-associated progesterone receptor component 2 | 0.906 | 0.489 |
| seq.10507.166 | Acrosomal protein SP-10 | 0.954 | 0.531 |
| seq.10510.62 | SLP adapter and CSK-interacting membrane protein | 0.559 | 0.727 |
| seq.10511.10 | Collagen alpha-3(VI) chain:isoform 3 | 0.074 | 0.000 |
| seq.10512.13 | Cytokine receptor common subunit beta:Extracellular domain | 0.634 | 0.562 |
| seq.10513.13 | Calsenilin | 0.931 | 0.790 |
| seq.10514.5 | Prostaglandin-H2 D-isomerase | 0.627 | 0.043 |
| seq.10521.10 | Matrix-remodeling-associated protein 8:Extracellular domain | 0.780 | 0.325 |
| seq.10527.22 | ATP-dependent RNA helicase A | 0.529 | 0.898 |
| seq.10528.2 | Dipeptidase 1 | 0.954 | 0.612 |
| seq.10530.8 | Proteasome subunit beta type-6 | 0.815 | 0.372 |
| seq.10531.18 | GTPase NRas | 0.488 | 0.000 |
| seq.10534.40 | Poly [ADP-ribose] polymerase 1:BRCA1 C-terminal:BRCA1 C-terminus domian | 0.226 | 0.575 |
| seq.10535.25 | Cytochrome c oxidase subunit 6A2, mitochondrial | 0.954 | 0.655 |
| seq.10539.30 | CD99 antigen-like protein 2 | 0.452 | 0.815 |
| seq.10546.2 | Thrombospondin-type laminin G domain and EAR repeat-containing protein | 0.781 | 0.763 |
| seq.10550.37 | Bone morphogenetic protein receptor type-1B | 0.378 | 0.590 |
| seq.10551.7 | Linker for activation of T-cells family member 1 | 0.985 | 0.955 |
| seq.10552.88 | NKG2-F type II integral membrane protein | 0.248 | 0.336 |
| seq.10553.8 | Torsin-1A-interacting protein 2 | 0.649 | 0.068 |
| seq.10554.23 | Beta-galactosidase | 0.050 | 0.000 |
| seq.10557.6 | Testis-expressed sequence 29 protein | 0.529 | 0.741 |
| seq.10558.26 | Protocadherin-9 | 0.655 | 0.429 |
| seq.10560.1 | Integral membrane protein 2C:N-term | 0.636 | 0.684 |
| seq.10561.5 | Peptidoglycan recognition protein 3 | 0.352 | 0.357 |
| seq.10562.42 | Neuropilin and tolloid-like protein 2 | 0.538 | 0.605 |
| seq.10563.13 | LysM and putative peptidoglycan-binding domain-containing protein 3 | 0.628 | 0.899 |
| seq.10565.19 | SLIT and NTRK-like protein 3 | 0.887 | 0.839 |
| seq.10569.28 | Microfibrillar-associated protein 2 | 0.881 | 0.308 |
| seq.10571.14 | Alpha-1,3-mannosyl-glycoprotein 2-beta-N-acetylglucosaminyltransferase | 0.758 | 0.887 |
| seq.10572.65 | Cystatin-8 | 0.749 | 0.443 |
| seq.10574.10 | Beta-2-microglobulin | 0.908 | 0.917 |
| seq.10575.31 | Poly(U)-binding-splicing factor PUF60 | 0.384 | 0.536 |
| seq.10576.7 | Plexin domain-containing protein 2:Extracellular domain | 0.703 | 0.327 |
| seq.10580.14 | Surfactant-associated protein 2 | 0.261 | 0.748 |
| seq.10582.36 | Protein O-mannose kinase | 0.692 | 0.648 |
| seq.10583.1 | Transmembrane protein 108 | 0.028 | 0.195 |
| seq.10584.7 | NADH dehydrogenase [ubiquinone] iron-sulfur protein 4, mitochondrial | 0.696 | 0.618 |
| seq.10588.39 | Lactosylceramide alpha-2,3-sialyltransferase | 0.538 | 0.951 |
| seq.10589.7 | Retinoic acid early transcript 1L protein | 0.741 | 0.787 |
| seq.10600.24 | Calmegin | 0.016 | 0.000 |
| seq.10603.1 | Histatin-3 | 0.507 | 0.398 |
| seq.10605.22 | Adipocyte plasma membrane-associated protein | 0.754 | 0.717 |
| seq.10606.34 | Torsin-1A-interacting protein 1:Perinuclear domain | 0.686 | 0.156 |
| seq.10608.9 | Histatin-1 | 0.878 | 0.768 |
| seq.10612.18 | Procollagen-lysine,2-oxoglutarate 5-dioxygenase 3 | 0.374 | 0.583 |
| seq.10613.33 | Protein CASC4 | 0.250 | 0.426 |
| seq.10615.18 | Myelin protein P0 | 0.674 | 0.876 |
| seq.10616.67 | Podocalyxin-like protein 2 | 0.930 | 0.297 |
| seq.10618.190 | Low-density lipoprotein receptor-related protein 2 | 0.712 | 0.738 |
| seq.10620.21 | Beta-microseminoprotein | 0.877 | 0.818 |
| seq.10621.26 | Protein Largen | 0.936 | 0.869 |
| seq.10623.19 | Mucin-1:region 1 | 0.336 | 0.830 |
| seq.10624.45 | Serine protease inhibitor Kazal-type 13 | 0.648 | 0.258 |
| seq.10626.116 | Alpha-N-acetylgalactosaminide alpha-2,6-sialyltransferase 3 | 0.855 | 0.000 |
| seq.10627.87 | Amyloid-like protein 2 | 0.683 | 0.443 |
| seq.10630.5 | Oxidoreductase HTATIP2 | 0.910 | 0.811 |
| seq.10631.9 | Membrane-associated progesterone receptor component 2 | 0.622 | 0.599 |
| seq.10636.1 | Cell surface glycoprotein CD200 receptor 1:Isoform 4, Cytoplasmic Domain | 0.803 | 0.685 |
| seq.10637.50 | UPF0577 protein KIAA1324:Extracellular domain | 0.500 | 0.361 |
| seq.10638.1 | TERF1-interacting nuclear factor 2 | 0.095 | 0.685 |
| seq.10643.16 | Noelin-3:Isoform 2, C-term | 0.000 | 0.894 |
| seq.10647.18 | Activating signal cointegrator 1 complex subunit 1 | 0.726 | 0.450 |
| seq.10663.42 | Insulin-induced gene 1 protein | 0.727 | 0.892 |
| seq.10666.7 | N-acetylglucosamine-1-phosphotransferase subunit gamma | 0.522 | 0.428 |
| seq.10667.78 | Uncharacterized protein C1orf185 | 0.513 | 0.297 |
| seq.10668.5 | Syntaxin-4 | 0.355 | 0.000 |
| seq.10670.26 | Semaphorin-3E | 0.000 | 0.000 |
| seq.10672.75 | Pulmonary surfactant-associated protein B | 0.830 | 0.798 |
| seq.10675.223 | Thioredoxin-related transmembrane protein 2 | 0.404 | 0.102 |
| seq.10677.9 | NADH dehydrogenase [ubiquinone] 1 beta subcomplex subunit 4 | 0.543 | 0.505 |
| seq.10693.43 | Killer cell lectin-like receptor subfamily G member 2:C-term | 0.821 | 0.917 |
| seq.10695.12 | Protein WFDC10B | 0.820 | 0.439 |
| seq.10699.52 | Prolow-density lipoprotein receptor-related protein 1 | 0.761 | 0.652 |
| seq.10700.10 | Immunoglobulin superfamily member 11:Cytoplasmic domain | 0.937 | 0.866 |
| seq.10702.1 | Collagen alpha-1(XXVIII) chain | 0.728 | 0.577 |
| seq.10703.203 | Sin3 histone deacetylase corepressor complex component SDS3 | 0.968 | 0.520 |
| seq.10704.91 | Lymphoid-restricted membrane protein | 0.937 | 0.739 |
| seq.10705.14 | Alpha-N-acetylgalactosaminide alpha-2,6-sialyltransferase 3 | 0.696 | 0.800 |
| seq.10708.3 | Progonadoliberin-2 | 0.914 | 0.554 |
| seq.10710.23 | Zona pellucida-binding protein 2 | 0.705 | 0.310 |
| seq.10713.151 | CMRF35-like molecule 7 | 0.472 | 0.756 |
| seq.10714.7 | Angiotensin-converting enzyme | 0.732 | 0.415 |
| seq.10716.35 | 26S proteasome non-ATPase regulatory subunit 5 | 0.827 | 0.863 |
| seq.10721.76 | Heat shock 70 kDa protein 1A | 0.266 | 0.565 |
| seq.10722.13 | Tyrosine-protein kinase SYK:Protein kinase domain | 0.157 | 0.000 |
| seq.10731.10 | Heparan sulfate glucosamine 3-O-sulfotransferase 5 | 0.346 | 0.402 |
| seq.10734.339 | Galactose-3-O-sulfotransferase 2 | 0.392 | 0.000 |
| seq.10737.96 | Leukocyte elastase inhibitor | 0.634 | 0.420 |
| seq.10738.11 | Fibulin-5 | 0.000 | 0.170 |
| seq.10741.22 | Macrophage colony-stimulating factor 1 | 0.864 | 0.840 |
| seq.10743.13 | SLIT and NTRK-like protein 1 | 0.795 | 0.672 |
| seq.10746.24 | Dickkopf-related protein 3 | 0.284 | 0.226 |
| seq.10748.216 | Protocadherin beta-2 | 0.833 | 0.432 |
| seq.10749.18 | Heat shock 70 kDa protein 1A | 0.397 | 0.485 |
| seq.10752.8 | Chromodomain Y-like protein 2 | 0.000 | 0.704 |
| seq.10753.31 | Disintegrin and metalloproteinase domain-containing protein 7 | 0.897 | 0.884 |
| seq.10754.113 | Prokineticin-2 | 0.756 | 0.351 |
| seq.10756.34 | Urocortin-3 | 0.035 | 0.190 |
| seq.10758.2 | Keratocan | 0.671 | 0.123 |
| seq.10760.107 | Glutamate receptor 4 | 0.442 | 0.000 |
| seq.10761.5 | Transmembrane emp24 domain-containing protein 2 | 0.603 | 0.963 |
| seq.10762.2 | Killer cell lectin-like receptor subfamily G member 2:N-term | 0.342 | 0.449 |
| seq.10767.52 | G-protein coupled receptor 64 | 0.906 | 0.437 |
| seq.10772.21 | Chondroitin sulfate N-acetylgalactosaminyltransferase 2 | 0.980 | 0.762 |
| seq.10780.10 | Ras-related protein Rab-3D | 0.610 | 0.622 |
| seq.10781.19 | C-type lectin domain family 4 member G | 0.028 | 0.777 |
| seq.10785.8 | Leucine-rich repeat-containing protein 52 | 0.789 | 0.804 |
| seq.10798.4 | CMRF35-like molecule 2 | 0.201 | 0.347 |
| seq.10800.15 | Serpin H1 | 0.141 | 0.000 |
| seq.10801.11 | Ephrin-A2 | 0.674 | 0.210 |
| seq.10803.22 | Heat shock 70 kDa protein 1A | 0.402 | 0.577 |
| seq.10809.14 | Killer cell lectin-like receptor subfamily B member 1 | 0.581 | 0.943 |
| seq.10814.7 | T-cell surface glycoprotein CD1a | 0.392 | 0.579 |
| seq.10815.2 | Intracellular hyaluronan-binding protein 4 | 0.937 | 0.576 |
| seq.10816.150 | Paired immunoglobulin-like type 2 receptor alpha isoform FDF03-M14 | 0.942 | 0.927 |
| seq.10817.26 | TOMM20-like protein 1 | 0.634 | 0.360 |
| seq.10818.36 | Sphingomyelin phosphodiesterase | 0.788 | 0.732 |
| seq.10819.108 | Fibulin-1 | 0.140 | 0.087 |
| seq.10825.12 | V-set and immunoglobulin domain-containing protein 10 | 0.299 | 0.426 |
| seq.10827.67 | Leucine-rich repeat and immunoglobulin-like domain-containing nogo receptor-interacting protein 3 | 0.490 | 0.569 |
| seq.10830.5 | EF-hand calcium-binding domain-containing protein 14:N-term | 0.239 | 0.709 |
| seq.10832.24 | Beta-1,4-galactosyltransferase 6 | 0.839 | 0.564 |
| seq.10833.64 | Hedgehog-interacting protein | 0.651 | 0.414 |
| seq.10835.25 | Alpha-1,4-N-acetylglucosaminyltransferase | 0.775 | 0.982 |
| seq.10842.7 | Beta-1,3-galactosyl-O-glycosyl-glycoprotein beta-1,6-N-acetylglucosaminyltransferase 4 | 0.057 | 0.389 |
| seq.10847.1 | Sialic acid-binding Ig-like lectin 15 | 0.688 | 0.940 |
| seq.10848.137 | Butyrophilin-like protein 3 | 0.472 | 0.106 |
| seq.10851.77 | Interleukin-27 subunit beta | 0.902 | 0.418 |
| seq.10852.114 | Heterogeneous nuclear ribonucleoprotein D-like | 0.188 | 0.091 |
| seq.10854.15 | Beta-1,3-galactosyl-O-glycosyl-glycoprotein beta-1,6-N-acetylglucosaminyltransferase 4 | 0.506 | 0.890 |
| seq.10855.55 | Plexin-B2 | 0.952 | 0.809 |
| seq.10866.60 | Serine/threonine-protein phosphatase 4 regulatory subunit 3A | 0.417 | 0.761 |
| seq.10870.32 | Spastin:Microtubule interacting and trafficking domain | 0.210 | 0.000 |
| seq.10876.300 | BRCA1-associated ATM activator 1 | 0.804 | 0.580 |
| seq.10880.38 | Protein FAM163B | 0.359 | 0.371 |
| seq.10882.12 | Desmocollin-1 | 0.236 | 0.450 |
| seq.10885.36 | Bombesin receptor-activated protein C6orf89 | 0.789 | 0.446 |
| seq.10889.2 | Melanocortin-2 receptor accessory protein 2 | 0.976 | 0.889 |
| seq.10890.135 | Lactase-like protein | 0.399 | 0.909 |
| seq.10892.8 | Oncostatin-M-specific receptor subunit beta | 0.789 | 0.845 |
| seq.10894.25 | Secretagogin | 0.490 | 0.659 |
| seq.10895.28 | Transmembrane protease serine 11B | 0.419 | 0.920 |
| seq.10900.272 | Stathmin-2 | 0.105 | 0.333 |
| seq.10902.53 | Retroviral-like aspartic protease 1 | 0.271 | 0.340 |
| seq.10903.50 | Syntaxin-8 | 0.098 | 0.000 |
| seq.10907.116 | Neurotrimin | 0.540 | 0.245 |
| seq.10908.2 | Polypeptide N-acetylgalactosaminyltransferase 13 | 0.817 | 0.707 |
| seq.10910.6 | Carcinoembryonic antigen-related cell adhesion molecule 4 | 0.986 | 0.375 |
| seq.10916.44 | Secretory phospholipase A2 receptor | 0.517 | 0.306 |
| seq.10917.40 | Guanine nucleotide-binding protein G(I)/G(S)/G(O) subunit gamma-T2 | 0.978 | 0.756 |
| seq.10924.258 | Neuferricin | 0.606 | 0.664 |
| seq.10933.107 | Calcium-binding protein 8 | 0.065 | 0.000 |
| seq.10938.13 | Lymphocyte function-associated antigen 3 | 0.612 | 0.603 |
| seq.10939.16 | Pleckstrin homology domain-containing family A member 4 | 0.581 | 0.614 |
| seq.10940.25 | Sarcalumenin | 0.713 | 0.305 |
| seq.10945.11 | Syntaxin-6 | 0.669 | 0.832 |
| seq.10948.14 | Phospholipase D3 | 0.620 | 0.219 |
| seq.10949.59 | 60S acidic ribosomal protein P2 | 0.193 | 0.481 |
| seq.10953.14 | C-type lectin domain family 2 member A | 0.265 | 0.339 |
| seq.10955.4 | C-type lectin domain family 10 member A | 0.545 | 0.005 |
| seq.10956.82 | Glycine cleavage system H protein, mitochondrial | 0.427 | 0.579 |
| seq.10959.125 | BET1-like protein | 0.585 | 0.426 |
| seq.10961.15 | Retinoic acid receptor responder protein 3 | 0.880 | 0.678 |
| seq.10966.1 | Alpha-2-HS-glycoprotein | 0.939 | 0.935 |
| seq.10967.12 | Prolactin | 0.377 | 0.374 |
| seq.10970.3 | Ecto-ADP-ribosyltransferase 3 | 0.748 | 0.484 |
| seq.10974.20 | Serine protease inhibitor Kazal-type 7 | 0.723 | 0.434 |
| seq.10975.59 | Mucin-like protein 1 | 0.676 | 0.828 |
| seq.10976.44 | Mucin-1:region 3 | 0.802 | 0.705 |
| seq.10977.55 | Unique cartilage matrix-associated protein | 0.796 | 0.854 |
| seq.10978.39 | Growth hormone variant | 0.520 | 0.365 |
| seq.10980.11 | Acetylcholinesterase | 0.643 | 0.417 |
| seq.10981.56 | Pro-neuregulin-3, membrane-bound isoform | 0.978 | 0.741 |
| seq.10990.21 | Leucine-rich repeat serine/threonine-protein kinase 2 | 0.000 | 0.820 |
| seq.11067.13 | Osteocalcin | 0.958 | 0.972 |
| seq.11071.1 | Interleukin-5 | 0.756 | 0.877 |
| seq.11081.1 | Glycerol-3-phosphate dehydrogenase [NAD(+)], cytoplasmic | 0.475 | 0.607 |
| seq.11083.23 | Gamma-enolase | 0.850 | 0.342 |
| seq.11089.7 | Immunoglobulin A | 0.877 | 0.927 |
| seq.11094.104 | Galectin-10 | 0.586 | 0.878 |
| seq.11096.57 | HemK methyltransferase family member 2 | 0.600 | 0.000 |
| seq.11098.1 | Pyridoxal kinase | 0.216 | 0.702 |
| seq.11101.18 | Toll-like receptor 4 | 0.775 | 0.921 |
| seq.11102.22 | Regenerating islet-derived protein 4 | 0.618 | 0.548 |
| seq.11103.24 | Heat shock protein beta-1 | 0.256 | 0.000 |
| seq.11104.13 | Chitinase-3-like protein 1 | 0.933 | 0.920 |
| seq.11105.171 | Alpha-enolase | 0.287 | 0.000 |
| seq.11107.25 | Teneurin-3 | 0.584 | 0.959 |
| seq.11109.56 | Sushi, von Willebrand factor type A, EGF and pentraxin domain-containing protein 1:Sushi 15-18 | 0.685 | 0.558 |
| seq.11110.4 | Transmembrane protein 119 | 0.925 | 0.806 |
| seq.11112.18 | Protein kish-A | 0.231 | 0.783 |
| seq.11116.16 | Uncharacterized protein C11orf87 | 0.560 | 0.444 |
| seq.11117.2 | Spermatogenesis-associated protein 20 | 0.282 | 0.792 |
| seq.11118.107 | Ras-related protein Rab-17 | 0.546 | 0.511 |
| seq.11120.49 | N-acetyltransferase 14 | 0.599 | 0.559 |
| seq.11121.56 | Brorin | 0.696 | 0.026 |
| seq.11122.97 | Maspardin | 0.084 | 0.000 |
| seq.11126.102 | Triple functional domain protein | 0.758 | 0.806 |
| seq.11128.29 | Transmembrane protein 132C:Cytoplasmic domain | 0.979 | 0.749 |
| seq.11129.66 | Bone morphogenetic protein 15 | 0.811 | 0.951 |
| seq.11130.158 | Voltage-dependent L-type calcium channel subunit beta-4 | 0.392 | 0.222 |
| seq.11134.30 | Peptide chain release factor 1-like, mitochondrial | 0.347 | 0.483 |
| seq.11135.5 | 39S ribosomal protein L55, mitochondrial | 0.310 | 0.041 |
| seq.11137.43 | Cytokine receptor common subunit beta:Cytoplasmic domain | 0.606 | 0.819 |
| seq.11138.16 | Runt-related transcription factor 3 | 0.805 | 0.680 |
| seq.11140.56 | Collagen alpha-1(I) chain:C-term propeptide | 0.548 | 0.598 |
| seq.11142.11 | Angiopoietin-related protein 1:C-Term, Fibrinogen domian | 0.544 | 0.415 |
| seq.11143.32 | Noelin-3:Isoform 2, N-term | 0.000 | 0.534 |
| seq.11144.10 | Beta-defensin 116 | 0.585 | 0.471 |
| seq.11145.72 | UPF0606 protein KIAA1549L | 0.753 | 0.531 |
| seq.11146.4 | T-box transcription factor TBX22 | 0.814 | 0.067 |
| seq.11147.17 | Trem-like transcript 1 protein:immunoreceptor tyrosine-based inhibition motif | 0.534 | 0.612 |
| seq.11149.3 | Toll-like receptor 1:Cytoplasmic domain | 0.926 | 0.439 |
| seq.11150.3 | Collagen alpha-1(VI) chain | 0.081 | 0.169 |
| seq.11152.46 | Kallikrein-13 | 0.487 | 0.541 |
| seq.11154.3 | Nuclear factor erythroid 2-related factor 1 | 0.957 | 0.835 |
| seq.11155.16 | Collagen alpha-5(VI) chain | 0.851 | 0.270 |
| seq.11157.35 | Heat shock 70 kDa protein 1A | 0.188 | 0.426 |
| seq.11158.40 | Bicaudal D-related protein 1 | 0.935 | 0.186 |
| seq.11159.14 | Mitochondrial ubiquitin ligase activator of NFKB 1:Cytoplasmic domain | 0.939 | 0.468 |
| seq.11160.56 | RING finger protein 122 | 0.054 | 0.555 |
| seq.11161.5 | Spartin | 0.242 | 0.000 |
| seq.11162.37 | Equatorin | 0.808 | 0.537 |
| seq.11163.7 | Protein FAM162B | 0.169 | 0.000 |
| seq.11164.7 | Teneurin-2 | 0.269 | 0.059 |
| seq.11167.6 | Myotubularin-related protein 1 | 0.891 | 0.817 |
| seq.11168.3 | Multiple epidermal growth factor-like domains protein 10 | 0.812 | 0.961 |
| seq.11171.25 | Filamin-A:Calponin Homology 2 | 0.182 | 0.000 |
| seq.11173.29 | Allergin-1 | 0.669 | 0.964 |
| seq.11174.8 | Thrombospondin type-1 domain-containing protein 7A:Thrombospondin type-1 domain 6 | 0.901 | 0.618 |
| seq.11175.45 | Lysophosphatidylcholine acyltransferase 2 | 0.376 | 0.525 |
| seq.11177.16 | Keratin, type II cytoskeletal 5 | 0.315 | 0.335 |
| seq.11178.21 | Sushi, von Willebrand factor type A, EGF and pentraxin domain-containing protein 1:EGF-like domains 4-6 | 0.648 | 0.523 |
| seq.11179.7 | Testis-specific serine/threonine-protein kinase 1 | 0.884 | 0.904 |
| seq.11180.17 | SWI/SNF complex subunit SMARCC1 | 0.861 | 0.414 |
| seq.11184.51 | Chromogranin-A | 0.650 | 0.676 |
| seq.11185.145 | GTP cyclohydrolase 1 | 0.525 | 0.605 |
| seq.11186.12 | Transmembrane protein 52 | 0.971 | 0.668 |
| seq.11187.11 | C-type lectin domain family 12 member A | 0.979 | 0.959 |
| seq.11190.129 | ATP-dependent zinc metalloprotease YME1L1 | 0.820 | 0.715 |
| seq.11192.168 | Tubulointerstitial nephritis antigen-like | 0.137 | 0.276 |
| seq.11193.27 | Hepatocyte nuclear factor 1-alpha | 0.982 | 0.900 |
| seq.11194.6 | Signaling threshold-regulating transmembrane adapter 1 | 0.960 | 0.912 |
| seq.11196.31 | Collagen alpha-3(VI) chain:Bovine pancreatic trypsin inhibitor/Kunitz inhibitor domain, isoform 1 | 0.776 | 0.572 |
| seq.11198.37 | Cyclic AMP-responsive element-binding protein 3-like protein 1 | 0.855 | 0.154 |
| seq.11200.52 | Complement component C1q receptor | 0.665 | 0.682 |
| seq.11201.19 | tRNA pseudouridine synthase A, mitochondrial | 0.716 | 0.179 |
| seq.11202.70 | T-box transcription factor TBX5 | 0.947 | 0.387 |
| seq.11203.97 | Pyruvate kinase PKLR | 0.479 | 0.290 |
| seq.11204.80 | Coxsackievirus and adenovirus receptor | 0.549 | 0.215 |
| seq.11205.10 | Integrin beta-7 | 0.543 | 0.320 |
| seq.11207.3 | Macrophage scavenger receptor types I and II:Cytoplasmic domain | 0.769 | 0.168 |
| seq.11208.15 | N-acetylglucosamine-1-phosphodiester alpha-N-acetylglucosaminidase | 0.488 | 0.655 |
| seq.11211.7 | Tubulin-specific chaperone E | 0.000 | 0.000 |
| seq.11212.7 | Thioredoxin domain-containing protein 5 | 0.840 | 0.541 |
| seq.11214.40 | DnaJ homolog subfamily B member 9 | 0.408 | 0.452 |
| seq.11215.6 | Cadherin-15:Cytoplasmic domain | 0.932 | 0.000 |
| seq.11217.16 | C-terminal-binding protein 1 | 0.351 | 0.119 |
| seq.11218.84 | Thiopurine S-methyltransferase | 0.561 | 0.469 |
| seq.11219.95 | Fibroblast growth factor-binding protein 3 | 0.757 | 0.598 |
| seq.11220.53 | Uncharacterized protein C10orf105 | 0.445 | 0.006 |
| seq.11223.1 | Transmembrane protein 154 | 0.771 | 0.426 |
| seq.11226.16 | Ubiquitin-protein ligase E3A | 0.456 | 0.000 |
| seq.11227.31 | Transducin beta-like protein 2 | 0.024 | 0.000 |
| seq.11228.37 | Protein RIC-3 | 0.169 | 0.959 |
| seq.11229.16 | Serine/threonine-protein kinase/endoribonuclease IRE1 | 0.965 | 0.500 |
| seq.11231.12 | Adrenodoxin-like protein, mitochondrial | 0.979 | 0.277 |
| seq.11232.46 | Transcobalamin-1 | 0.790 | 0.453 |
| seq.11237.49 | Procollagen C-endopeptidase enhancer 1 | 0.450 | 0.793 |
| seq.11239.49 | Transmembrane protease serine 6 | 0.726 | 0.913 |
| seq.11241.8 | Argininosuccinate lyase | 0.563 | 0.671 |
| seq.11242.33 | Protein-glutamine gamma-glutamyltransferase K | 0.454 | 0.949 |
| seq.11243.90 | Adhesion G-protein coupled receptor F1 | 0.103 | 0.466 |
| seq.11245.43 | Filamin-A:Calponin Homology 1 | 0.199 | 0.008 |
| seq.11246.3 | BPI fold-containing family B member 1 | 0.919 | 0.784 |
| seq.11247.20 | N-acetylglutamate synthase, mitochondrial | 0.873 | 0.506 |
| seq.11248.43 | Uroporphyrinogen-III synthase | 0.418 | 0.422 |
| seq.11252.30 | Leucine-rich repeat and calponin homology domain-containing protein 4:Calponin Homology | 0.598 | 0.668 |
| seq.11254.13 | Papilin | 0.625 | 0.889 |
| seq.11257.1 | Dihydropteridine reductase | 0.455 | 0.000 |
| seq.11258.41 | Mucosal addressin cell adhesion molecule 1 | 0.225 | 0.066 |
| seq.11260.47 | SUN domain-containing protein 5 | 0.794 | 0.978 |
| seq.11262.39 | TraB domain-containing protein | 0.186 | 0.299 |
| seq.11263.57 | Calsequestrin-1 | 0.652 | 0.238 |
| seq.11264.33 | Xanthine dehydrogenase/oxidase | 0.923 | 0.887 |
| seq.11265.8 | Retinal dehydrogenase 1 | 0.572 | 0.013 |
| seq.11266.8 | P-selectin glycoprotein ligand 1:Extracellular domain | 0.633 | 0.762 |
| seq.11270.17 | Coiled-coil-helix-coiled-coil-helix domain-containing protein 10, mitochondrial | 0.942 | 0.770 |
| seq.11273.176 | Glutathione S-transferase theta-2B | 0.578 | 0.100 |
| seq.11275.94 | Low-density lipoprotein receptor-related protein 1B | 0.403 | 0.589 |
| seq.11277.23 | Cyclic AMP-dependent transcription factor ATF-6 alpha | 0.697 | 0.600 |
| seq.11278.4 | Collagen alpha-2(XI) chain | 0.649 | 0.730 |
| seq.11279.42 | Gamma-aminobutyric acid type B receptor subunit 1 | 0.917 | 0.211 |
| seq.11280.6 | Glutamate decarboxylase 1 | 0.676 | 0.630 |
| seq.11281.6 | Growth factor receptor-bound protein 7 | 0.330 | 0.549 |
| seq.11282.16 | Macrophage scavenger receptor types I and II:Extracellular domain | 0.545 | 0.749 |
| seq.11283.13 | DDB1- and CUL4-associated factor 5 | 0.173 | 0.709 |
| seq.11284.24 | Leukocyte-associated immunoglobulin-like receptor 1 | 0.679 | 0.143 |
| seq.11285.8 | Hematopoietic progenitor cell antigen CD34 | 0.002 | 0.576 |
| seq.11286.78 | Selenoprotein S | 0.105 | 0.664 |
| seq.11287.14 | Cytochrome b5 | 0.641 | 0.563 |
| seq.11288.26 | Cytosolic purine 5'-nucleotidase | 0.191 | 0.353 |
| seq.11289.31 | Casein kinase I isoform delta | 0.370 | 0.668 |
| seq.11292.13 | Scavenger receptor class A member 3:region 2 | 0.000 | 0.466 |
| seq.11293.14 | Leucine-rich repeat neuronal protein 1:Cytoplasmic domain | 0.890 | 0.720 |
| seq.11294.7 | Transmembrane and coiled-coil domains protein 3:region 2 | 0.553 | 0.481 |
| seq.11297.54 | Neurogenic locus notch homolog protein 2 | 0.670 | 0.370 |
| seq.11300.32 | Sortilin | 0.875 | 0.969 |
| seq.11302.237 | Tenascin-R | 0.439 | 0.594 |
| seq.11303.7 | Deoxynucleoside triphosphate triphosphohydrolase SAMHD1 | 0.634 | 0.955 |
| seq.11307.33 | NEDD4-like E3 ubiquitin-protein ligase WWP1 | 0.059 | 0.000 |
| seq.11308.8 | Cyclic AMP-responsive element-binding protein 3-like protein 4 | 0.735 | 0.578 |
| seq.11310.8 | Desmoglein-3 | 0.144 | 0.302 |
| seq.11311.79 | V(D)J recombination-activating protein 1 | 0.591 | 0.205 |
| seq.11312.40 | Mismatch repair endonuclease PMS2 | 0.892 | 0.919 |
| seq.11313.100 | Pterin-4-alpha-carbinolamine dehydratase | 0.448 | 0.434 |
| seq.11315.148 | Protein phosphatase 1D | 0.971 | 0.722 |
| seq.11318.20 | Apolipoprotein A-V | 0.751 | 0.491 |
| seq.11319.106 | Double-strand break repair protein MRE11 | 0.475 | 0.827 |
| seq.11320.29 | E3 ubiquitin-protein ligase CHFR | 0.000 | 0.465 |
| seq.11324.3 | Peroxidasin-like protein | 0.540 | 0.699 |
| seq.11325.8 | Protein BTG2 | 0.203 | 0.545 |
| seq.11327.56 | Dual specificity protein kinase CLK2 | 0.538 | 0.679 |
| seq.11328.9 | U6 snRNA phosphodiesterase | 0.652 | 0.502 |
| seq.11330.15 | Casein kinase II subunit beta | 0.255 | 0.863 |
| seq.11333.82 | Rho GTPase-activating protein 25 | 0.108 | 0.027 |
| seq.11334.7 | Leukocyte immunoglobulin-like receptor subfamily B member 3 | 0.729 | 0.974 |
| seq.11336.9 | Fanconi anemia group F protein | 0.917 | 0.000 |
| seq.11338.49 | Tyrosine-protein kinase BLK | 0.000 | 0.785 |
| seq.11342.59 | Plexin domain-containing protein 2:Cytoplasmic domain | 0.362 | 0.000 |
| seq.11347.9 | Transaldolase | 0.259 | 0.448 |
| seq.11348.132 | Prolyl 4-hydroxylase subunit alpha-2 | 0.869 | 0.290 |
| seq.11350.30 | E3 ubiquitin-protein ligase CHIP | 0.483 | 0.000 |
| seq.11351.233 | Non-homologous end-joining factor 1 | 0.826 | 0.505 |
| seq.11352.42 | Titin | 0.593 | 0.000 |
| seq.11353.143 | Mothers against decapentaplegic homolog 2 | 0.324 | 0.322 |
| seq.11354.21 | 1-phosphatidylinositol 4,5-bisphosphate phosphodiesterase beta-1 | 0.397 | 0.326 |
| seq.11355.10 | Eukaryotic translation initiation factor 5A-2 | 0.096 | 0.000 |
| seq.11356.19 | Protein DGCR14 | 0.509 | 0.105 |
| seq.11358.15 | Growth factor receptor-bound protein 10 | 0.036 | 0.000 |
| seq.11360.39 | Ribonucleoside-diphosphate reductase large subunit | 0.591 | 0.255 |
| seq.11361.73 | Thymidine phosphorylase | 0.187 | 0.000 |
| seq.11363.58 | Aquaporin-4 | 0.409 | 0.802 |
| seq.11364.18 | Mitofusin-1 | 0.970 | 0.931 |
| seq.11365.17 | Teneurin-4 | 0.728 | 0.000 |
| seq.11368.32 | Adenylate kinase 2, mitochondrial | 0.001 | 0.000 |
| seq.11369.23 | Alcohol dehydrogenase class-3 | 0.055 | 0.000 |
| seq.11370.20 | 72 kDa inositol polyphosphate 5-phosphatase | 0.637 | 0.772 |
| seq.11371.1 | Probable G-protein coupled receptor 101 | 0.835 | 0.294 |
| seq.11372.2 | Zinc finger protein 18 | 0.331 | 0.301 |
| seq.11375.49 | Forkhead box protein L2 | 0.562 | 0.878 |
| seq.11377.19 | Alcohol dehydrogenase class 4 mu/sigma chain | 0.637 | 0.723 |
| seq.11378.37 | Tyrosine-protein kinase SYK:Src Homology domain | 0.526 | 0.434 |
| seq.11380.84 | RNA-binding protein 24 | 0.669 | 0.807 |
| seq.11381.56 | Ribose-phosphate pyrophosphokinase 1 | 0.792 | 0.881 |
| seq.11382.5 | Biliverdin reductase A | 0.231 | 0.037 |
| seq.11383.41 | Keratin, type II cytoskeletal 7 | 0.499 | 0.416 |
| seq.11387.3 | Cyclic AMP-dependent transcription factor ATF-6 beta | 0.899 | 0.000 |
| seq.11388.75 | WAP four-disulfide core domain protein 2 | 0.715 | 0.439 |
| seq.11390.24 | Carbonic anhydrase-related protein | 0.614 | 0.000 |
| seq.11391.69 | Mevalonate kinase | 0.816 | 0.744 |
| seq.11395.5 | Protein-tyrosine kinase 2-beta:4.1 protein, ezrin, radixin, moesin domain | 0.480 | 0.000 |
| seq.11396.39 | Dynein intermediate chain 1, axonemal | 0.568 | 0.408 |
| seq.11401.181 | E3 ubiquitin-protein ligase RNF146 | 0.440 | 0.264 |
| seq.11402.17 | Histone-lysine N-methyltransferase 2C | 0.728 | 0.617 |
| seq.11405.150 | Caspase recruitment domain-containing protein 9 | 0.240 | 0.246 |
| seq.11406.82 | Isobutyryl-CoA dehydrogenase, mitochondrial | 0.807 | 0.944 |
| seq.11407.57 | Phospholipid scramblase 3 | 0.570 | 0.914 |
| seq.11416.23 | F-box/LRR-repeat protein 4:Leucine-rich repeats 4 and 5 | 0.360 | 0.030 |
| seq.11421.10 | EH domain-containing protein 4 | 0.584 | 0.000 |
| seq.11422.2 | Homeobox protein DLX-3 | 0.886 | 0.395 |
| seq.11424.4 | Fumarylacetoacetase | 0.720 | 0.741 |
| seq.11425.31 | RUN and FYVE domain-containing protein 1 | 0.000 | 0.405 |
| seq.11428.31 | PDZ and LIM domain protein 1 | 0.088 | 0.000 |
| seq.11429.80 | Heterogeneous nuclear ribonucleoproteins C1/C2 | 0.430 | 0.770 |
| seq.11430.49 | E3 ubiquitin-protein ligase DTX1 | 0.450 | 0.567 |
| seq.11431.235 | ATP-dependent DNA helicase Q1 | 0.209 | 0.295 |
| seq.11432.11 | Polyglutamine-binding protein 1 | 0.434 | 0.691 |
| seq.11433.11 | Tectonic-2 | 0.886 | 0.813 |
| seq.11436.6 | Alpha-internexin | 0.814 | 0.941 |
| seq.11438.6 | DnaJ homolog subfamily B member 2 | 0.885 | 0.393 |
| seq.11439.88 | Rhophilin-2 | 0.192 | 0.794 |
| seq.11440.58 | Suppressor of cytokine signaling 3 | 0.654 | 0.898 |
| seq.11441.11 | Glycogen phosphorylase, liver form | 0.374 | 0.000 |
| seq.11442.1 | Delta and Notch-like epidermal growth factor-related receptor:Cytoplasmic domain | 0.770 | 0.728 |
| seq.11444.49 | DNA-directed RNA polymerase III subunit RPC6 | 0.520 | 0.406 |
| seq.11448.34 | Galactokinase | 0.269 | 0.000 |
| seq.11449.22 | F-actin-capping protein subunit alpha-1 | 0.322 | 0.000 |
| seq.11450.110 | Protein disulfide-isomerase | 0.434 | 0.808 |
| seq.11454.87 | Eukaryotic translation initiation factor 3 subunit G | 0.304 | 0.000 |
| seq.11456.2 | Melanoma-associated antigen B10 | 0.384 | 0.145 |
| seq.11457.53 | UDP-glucose 4-epimerase | 0.383 | 0.520 |
| seq.11458.30 | Poly(rC)-binding protein 1 | 0.000 | 0.034 |
| seq.11459.81 | RNA polymerase II elongation factor ELL | 0.369 | 0.322 |
| seq.11462.8 | RNA binding protein fox-1 homolog 2 | 0.359 | 0.197 |
| seq.11464.9 | Transcription factor RelB | 0.566 | 0.553 |
| seq.11465.4 | Probable G-protein coupled receptor 135 | 0.707 | 0.570 |
| seq.11468.15 | Probable RNA-binding protein 19 | 0.715 | 0.157 |
| seq.11476.43 | Vacuolar protein sorting-associated protein 4A | 0.240 | 0.000 |
| seq.11480.1 | Aldehyde dehydrogenase, dimeric NADP-preferring | 0.358 | 0.085 |
| seq.11481.25 | Hepatitis A virus cellular receptor 2 | 0.930 | 0.977 |
| seq.11486.26 | Zinc finger protein 174 | 0.715 | 0.773 |
| seq.11487.4 | Testican-1 | 0.925 | 0.540 |
| seq.11490.42 | Ubiquitin-like protein 4A | 0.378 | 0.000 |
| seq.11493.169 | Dynein light chain 2, cytoplasmic | 0.000 | 0.000 |
| seq.11494.4 | RNA polymerase II elongation factor ELL2 | 0.770 | 0.512 |
| seq.11510.31 | Apolipoprotein L1 | 0.579 | 0.716 |
| seq.11510.51 | Apolipoprotein L1 | 0.609 | 0.806 |
| seq.11513.92 | ADP-ribosyl cyclase/cyclic ADP-ribose hydrolase 1 | 0.393 | 0.254 |
| seq.11514.196 | CD59 glycoprotein | 0.647 | 0.088 |
| seq.11516.7 | Fatty acid-binding protein, liver | 0.660 | 0.520 |
| seq.11530.37 | Porphobilinogen deaminase | 0.423 | 0.133 |
| seq.11531.24 | V-type immunoglobulin domain-containing suppressor of T-cell activation:Cytoplasmic domain | 0.466 | 0.000 |
| seq.11534.6 | Leucine-rich repeat, immunoglobulin-like domain and transmembrane domain-containing protein 3 | 0.834 | 0.071 |
| seq.11536.9 | Histone H2A deubiquitinase MYSM1 | 0.821 | 0.793 |
| seq.11537.12 | Transferrin receptor protein 2 | 0.970 | 0.619 |
| seq.11538.216 | Malonyl-CoA decarboxylase, mitochondrial | 0.525 | 0.107 |
| seq.11539.4 | ATP synthase subunit f, mitochondrial | 0.452 | 0.598 |
| seq.11540.37 | Forkhead box protein O3 | 0.652 | 0.795 |
| seq.11542.11 | Transmembrane protein 230 | 0.993 | 0.885 |
| seq.11543.84 | LIM domain and actin-binding protein 1 | 0.869 | 0.380 |
| seq.11544.39 | PHD finger protein 3 | 0.887 | 0.385 |
| seq.11545.9 | Tumor necrosis factor receptor superfamily member 14 | 0.347 | 0.433 |
| seq.11546.7 | Cytoglobin | 0.474 | 0.463 |
| seq.11547.84 | Muscle, skeletal receptor tyrosine-protein kinase | 0.925 | 0.584 |
| seq.11548.84 | M-phase inducer phosphatase 1 | 0.815 | 0.649 |
| seq.11549.6 | Insulin gene enhancer protein ISL-1 | 0.348 | 0.000 |
| seq.11551.16 | SWI/SNF-related matrix-associated actin-dependent regulator of chromatin subfamily E member 1-related | 0.855 | 0.687 |
| seq.11556.19 | ADP-ribosylation factor GTPase-activating protein 1 | 0.478 | 0.507 |
| seq.11557.3 | E3 ubiquitin-protein ligase SMURF1 | 0.881 | 0.286 |
| seq.11560.76 | Nuclear factor of activated T-cells, cytoplasmic 4 | 0.498 | 0.633 |
| seq.11562.9 | DNA polymerase epsilon subunit 2 | 0.889 | 0.240 |
| seq.11563.51 | Synaptotagmin-like protein 4:Ca2+-dependent membrane-targeting module 2 | 0.000 | 0.684 |
| seq.11565.58 | Zinc finger protein 23 | 0.776 | 0.881 |
| seq.11566.48 | Keratin, type II cytoskeletal 72 | 0.362 | 0.000 |
| seq.11567.23 | Zinc finger protein 10 | 0.546 | 0.588 |
| seq.11568.2 | Peptidyl-prolyl cis-trans isomerase FKBP1B | 0.321 | 0.000 |
| seq.11570.94 | B-cell receptor-associated protein 29 | 0.496 | 0.551 |
| seq.11571.75 | NKG2-E type II integral membrane protein:Isoform E, Extracellular domain | 0.538 | 0.208 |
| seq.11572.4 | Dynamin-2 | 0.079 | 0.000 |
| seq.11573.3 | Serine/arginine-rich splicing factor 6 | 0.745 | 0.507 |
| seq.11582.63 | DnaJ homolog subfamily A member 2 | 0.650 | 0.032 |
| seq.11586.2 | Leucine-rich repeat neuronal protein 1:Extracellular domain | 0.368 | 0.488 |
| seq.11587.5 | MAX gene-associated protein | 0.563 | 0.722 |
| seq.11590.5 | Probable RNA-binding protein 23 | 0.263 | 0.239 |
| seq.11591.43 | Protein regulator of cytokinesis 1 | 0.569 | 0.551 |
| seq.11592.1 | ELAV-like protein 1 | 0.396 | 0.871 |
| seq.11593.21 | C-X-C motif chemokine 9 | 0.319 | 0.121 |
| seq.11596.47 | Zinc finger protein 75D | 0.732 | 0.100 |
| seq.11601.26 | ATP-dependent RNA helicase DHX8 | 0.364 | 0.785 |
| seq.11602.12 | Copine-1:Von Willebrand factor type A domain | 0.278 | 0.651 |
| seq.11606.22 | DnaJ homolog subfamily B member 6 | 0.559 | 0.328 |
| seq.11607.15 | Bromodomain-containing protein 1 | 0.410 | 0.124 |
| seq.11608.5 | Microtubule-associated proteins 1A/1B light chain 3B | 0.000 | 0.000 |
| seq.11614.29 | Enhancer of rudimentary homolog | 0.326 | 0.255 |
| seq.11615.16 | Dual adapter for phosphotyrosine and 3-phosphotyrosine and 3-phosphoinositide | 0.028 | 0.000 |
| seq.11616.9 | Heat shock factor protein 1 | 0.498 | 0.381 |
| seq.11617.1 | Integrin alpha-L | 0.000 | 0.686 |
| seq.11618.83 | Transcriptional activator Myb | 0.697 | 0.300 |
| seq.11626.7 | Ubiquitin-conjugating enzyme E2 variant 1 | 0.547 | 0.050 |
| seq.11629.36 | TNF receptor-associated factor 4 | 0.407 | 0.390 |
| seq.11633.89 | Activator of 90 kDa heat shock protein ATPase homolog 1 | 0.035 | 0.000 |
| seq.11634.32 | Regulator of G-protein signaling 10 | 0.000 | 0.000 |
| seq.11636.33 | Transgelin-2 | 0.016 | 0.391 |
| seq.11638.42 | Beta-1,3-galactosyltransferase 2 | 0.843 | 0.591 |
| seq.11643.73 | E3 ubiquitin-protein ligase DTX3L | 0.957 | 0.452 |
| seq.11645.9 | Prolyl 4-hydroxylase subunit alpha-1 | 0.207 | 0.054 |
| seq.11646.4 | Carbohydrate sulfotransferase 9 | 0.822 | 0.705 |
| seq.11647.6 | Frizzled-10:Cytoplasmic domain | 0.278 | 0.687 |
| seq.11649.3 | Stromal membrane-associated protein 1 | 0.093 | 0.000 |
| seq.11651.24 | Protein argonaute-1 | 0.590 | 0.382 |
| seq.11653.69 | Sodium- and chloride-dependent glycine transporter 1 | 0.561 | 0.127 |
| seq.11654.77 | Neurensin-1 | 0.557 | 0.158 |
| seq.11656.110 | Ena/VASP-like protein | 0.726 | 0.419 |
| seq.11657.86 | Suppressor of cytokine signaling 7 | 0.467 | 0.581 |
| seq.11659.31 | Clathrin interactor 1 | 0.130 | 0.000 |
| seq.11661.11 | NACHT, LRR and PYD domains-containing protein 1 | 0.586 | 0.756 |
| seq.11664.32 | ADP-ribosylation factor GTPase-activating protein 2 | 0.000 | 0.000 |
| seq.11666.72 | Regulator of G-protein signaling 8 | 0.456 | 0.440 |
| seq.11667.29 | Tensin-2 | 0.849 | 0.000 |
| seq.11669.39 | Solute carrier organic anion transporter family member 5A1 | 0.955 | 0.837 |
| seq.11670.18 | Probable G-protein coupled receptor 101 | 0.412 | 0.748 |
| seq.11671.19 | Peregrin | 0.053 | 0.119 |
| seq.11672.17 | Kinesin-like protein KIF16B | 0.192 | 0.481 |
| seq.11677.17 | Probable palmitoyltransferase ZDHHC14 | 0.606 | 0.972 |
| seq.11678.105 | Gap junction delta-2 protein | 0.772 | 0.799 |
| seq.11681.8 | Arf-GAP domain and FG repeat-containing protein 1 | 0.000 | 0.000 |
| seq.11682.7 | Thioredoxin-interacting protein | 0.179 | 0.615 |
| seq.11683.19 | ADP-ribosylation factor-binding protein GGA3 | 0.091 | 0.000 |
| seq.11690.47 | Anaphase-promoting complex subunit 7 | 0.816 | 0.896 |
| seq.11692.21 | SHC-transforming protein 4 | 0.415 | 0.270 |
| seq.11696.7 | Cellular retinoic acid-binding protein 2 | 0.648 | 0.347 |
| seq.11699.16 | Protein tyrosine phosphatase type IVA 2 | 0.374 | 0.000 |
| seq.11708.2 | Lipocalin-1 | 0.764 | 0.810 |
| seq.11709.29 | Carnitine O-palmitoyltransferase 1, muscle isoform | 0.928 | 0.391 |
| seq.11712.207 | Protein unc-45 homolog A | 0.081 | 0.000 |
| seq.11715.1 | POU domain, class 2, transcription factor 1 | 0.376 | 0.592 |
| seq.11716.28 | Leucine-rich repeat, immunoglobulin-like domain and transmembrane domain-containing protein 2 | 0.214 | 0.455 |
| seq.11814.29 | Hepatocyte growth factor receptor | 0.739 | 0.172 |
| seq.11816.84 | Tyrosine-protein kinase JAK2 | 0.000 | 0.000 |
| seq.11817.1 | Ribosomal protein S6 kinase beta-1 | 0.338 | 0.000 |
| seq.11825.27 | Peroxisome proliferator-activated receptor gamma coactivator 1-alpha | 0.051 | 0.627 |
| seq.11827.7 | Nuclear receptor ROR-gamma | 0.695 | 0.352 |
| seq.11830.48 | Tyrosine-protein phosphatase non-receptor type 11 | 0.000 | 0.000 |
| seq.11833.83 | Frataxin, mitochondrial | 0.897 | 0.825 |
| seq.11836.144 | Epithelial discoidin domain-containing receptor 1 | 0.589 | 0.471 |
| seq.11837.7 | Tumor necrosis factor receptor superfamily member 18 | 0.636 | 0.682 |
| seq.11838.130 | Piezo-type mechanosensitive ion channel component 1 | 0.527 | 0.741 |
| seq.11851.21 | Triggering receptor expressed on myeloid cells 2 | 0.759 | 0.421 |
| seq.11872.9 | Protocadherin gamma-B1 | 0.988 | 0.890 |
| seq.11910.27 | Homeobox protein DLX-4 | 0.142 | 0.000 |
| seq.11911.13 | Leucine-rich repeat-containing protein 4B:Cytoplasmic domain | 0.943 | 0.799 |
| seq.11926.23 | Alpha- and gamma-adaptin-binding protein p34 | 0.364 | 0.273 |
| seq.11934.9 | Integrator complex subunit 3 | 0.984 | 0.864 |
| seq.11949.25 | Epidermal growth factor:Cytoplasmic domain | 0.747 | 0.622 |
| seq.11952.1 | Immunoglobulin superfamily DCC subclass member 3:Cytoplasmic domain | 0.860 | 0.194 |
| seq.11955.1 | Rho GTPase-activating protein 1:Rho-GTPase activating protein domain | 0.364 | 0.314 |
| seq.11967.23 | Cellular retinoic acid-binding protein 1 | 0.577 | 0.454 |
| seq.11969.5 | Protein S100-A2 | 0.417 | 0.483 |
| seq.11988.24 | Receptor-type tyrosine-protein phosphatase H | 0.805 | 0.565 |
| seq.11989.35 | ER membrane protein complex subunit 1 | 0.990 | 0.620 |
| seq.12001.7 | Tight junction protein ZO-1 | 0.871 | 0.634 |
| seq.12008.3 | T-cell antigen CD7 | 0.523 | 0.109 |
| seq.12014.19 | 6-pyruvoyl tetrahydrobiopterin synthase | 0.735 | 0.847 |
| seq.12016.60 | E3 ubiquitin-protein ligase CBL | 0.023 | 0.000 |
| seq.12018.84 | Thiamin pyrophosphokinase 1 | 0.580 | 0.843 |
| seq.12020.39 | Bisphosphoglycerate mutase | 0.436 | 0.044 |
| seq.12022.12 | Mothers against decapentaplegic homolog 4 | 0.502 | 0.233 |
| seq.12030.82 | Desmin | 0.719 | 0.443 |
| seq.12033.3 | Mitotic checkpoint serine/threonine-protein kinase BUB1 | 0.838 | 0.847 |
| seq.12034.28 | Adenylyl cyclase-associated protein 1 | 0.000 | 0.000 |
| seq.12041.33 | Heat shock 70 kDa protein 1-like | 0.642 | 0.583 |
| seq.12046.51 | TAR DNA-binding protein 43 | 0.288 | 0.411 |
| seq.12077.32 | Growth/differentiation factor 8 | 0.875 | 0.567 |
| seq.12329.21 | Ribosomal protein S6 kinase alpha-1 | 0.790 | 0.847 |
| seq.12332.7 | Eukaryotic elongation factor 2 kinase | 0.370 | 0.666 |
| seq.12333.87 | Ribose-5-phosphate isomerase | 0.654 | 0.517 |
| seq.12334.25 | Serine hydroxymethyltransferase, cytosolic | 0.411 | 0.081 |
| seq.12338.27 | Pikachurin | 0.480 | 0.352 |
| seq.12340.17 | Alanine--tRNA ligase, cytoplasmic | 0.000 | 0.000 |
| seq.12341.8 | Dual specificity protein phosphatase 6 | 0.737 | 0.152 |
| seq.12343.14 | Arf-GAP with coiled-coil, ANK repeat and PH domain-containing protein 2 | 0.782 | 0.000 |
| seq.12345.4 | Anaphase-promoting complex subunit 10 | 0.734 | 0.566 |
| seq.12347.29 | Cerebral cavernous malformations 2 protein | 0.275 | 0.000 |
| seq.12348.46 | Serine--tRNA ligase, mitochondrial | 0.362 | 0.069 |
| seq.12350.86 | Protein C-ets-2 | 0.587 | 0.790 |
| seq.12351.25 | Signal transducer and activator of transcription 1-alpha/beta | 0.362 | 0.000 |
| seq.12352.70 | Arrestin domain-containing protein 3 | 0.849 | 0.578 |
| seq.12355.223 | Ribosome-recycling factor, mitochondrial | 0.567 | 0.528 |
| seq.12356.65 | Sorcin | 0.495 | 0.177 |
| seq.12357.41 | Synaptosomal-associated protein 29 | 0.811 | 0.638 |
| seq.12358.6 | Immunoglobulin-binding protein 1 | 0.291 | 0.001 |
| seq.12361.102 | Ras-related protein R-Ras2 | 0.614 | 0.827 |
| seq.12363.70 | Tribbles homolog 2 | 0.496 | 0.619 |
| seq.12365.108 | RuvB-like 1 | 0.405 | 0.423 |
| seq.12366.16 | Gamma-crystallin D | 0.451 | 0.014 |
| seq.12367.52 | Macoilin | 0.699 | 0.084 |
| seq.12368.18 | Histone acetyltransferase KAT2B | 0.520 | 0.222 |
| seq.12370.30 | Apolipoprotein F | 0.813 | 0.597 |
| seq.12372.50 | Tropomyosin alpha-3 chain | 0.578 | 0.596 |
| seq.12373.73 | Transformer-2 protein homolog beta | 0.631 | 0.407 |
| seq.12374.8 | Platelet-activating factor acetylhydrolase IB subunit gamma | 0.470 | 0.075 |
| seq.12376.85 | Cyclin-dependent kinase 4 inhibitor D | 0.000 | 0.000 |
| seq.12378.71 | Tapasin | 0.671 | 0.684 |
| seq.12381.26 | Carbonyl reductase [NADPH] 1 | 0.566 | 0.313 |
| seq.12382.2 | Probable ATP-dependent RNA helicase DDX58 | 0.143 | 0.817 |
| seq.12384.92 | COP9 signalosome complex subunit 7b | 0.881 | 0.877 |
| seq.12385.4 | Calpain-3 | 0.800 | 0.572 |
| seq.12386.11 | Aminopeptidase B | 0.453 | 0.509 |
| seq.12387.7 | PDZ and LIM domain protein 4 | 0.887 | 0.732 |
| seq.12389.4 | Origin recognition complex subunit 6 | 0.496 | 0.368 |
| seq.12391.27 | PDZ domain-containing protein 7 | 0.129 | 0.024 |
| seq.12392.30 | ADP-ribosylation factor-like protein 1 | 0.259 | 0.405 |
| seq.12394.53 | Transmembrane protein C16orf54 | 0.887 | 0.855 |
| seq.12395.86 | Aspartate--tRNA ligase, mitochondrial | 0.124 | 0.000 |
| seq.12396.19 | 3-hydroxyisobutyryl-CoA hydrolase, mitochondrial | 0.213 | 0.000 |
| seq.12398.15 | Paired box protein Pax-4 | 0.799 | 0.720 |
| seq.12399.194 | Coiled-coil domain-containing protein 50 | 0.774 | 0.000 |
| seq.12400.25 | Ubiquitin-conjugating enzyme E2 T | 0.255 | 0.672 |
| seq.12401.3 | AMSH-like protease | 0.760 | 0.652 |
| seq.12403.30 | Ras-related protein Rab-39B | 0.426 | 0.771 |
| seq.12406.119 | GTP-binding protein Di-Ras3 | 0.387 | 0.920 |
| seq.12408.333 | Ras-related protein Rab-22A | 0.368 | 0.000 |
| seq.12409.90 | Ras-related protein Rab-7b | 0.568 | 0.699 |
| seq.12411.60 | Protein max | 0.089 | 0.000 |
| seq.12414.31 | 14-3-3 protein beta/alpha | 0.000 | 0.000 |
| seq.12415.122 | Endothelial differentiation-related factor 1 | 0.117 | 0.473 |
| seq.12417.46 | EKC/KEOPS complex subunit TPRKB | 0.000 | 0.775 |
| seq.12420.10 | Glycerol-3-phosphate dehydrogenase 1-like protein | 0.423 | 0.461 |
| seq.12422.143 | Arachidonate 15-lipoxygenase B | 0.620 | 0.569 |
| seq.12423.38 | APOBEC1 complementation factor | 0.535 | 0.588 |
| seq.12424.107 | Thymocyte nuclear protein 1 | 0.764 | 0.813 |
| seq.12425.104 | ADP-ribosylation factor 6 | 0.224 | 0.158 |
| seq.12426.19 | MOB kinase activator 1A | 0.418 | 0.000 |
| seq.12427.8 | M-phase inducer phosphatase 2 | 0.000 | 0.373 |
| seq.12428.2 | Lysophospholipase-like protein 1 | 0.670 | 0.205 |
| seq.12430.78 | Polyadenylate-binding protein-interacting protein 1 | 0.781 | 0.038 |
| seq.12431.13 | Protein pelota homolog | 0.328 | 0.806 |
| seq.12432.23 | Calcyclin-binding protein | 0.005 | 0.000 |
| seq.12433.8 | ADP-ribosylation factor-like protein 11 | 0.873 | 0.000 |
| seq.12434.25 | IST1 homolog | 0.152 | 0.000 |
| seq.12436.84 | Glutathione S-transferase omega-1 | 0.663 | 0.479 |
| seq.12437.18 | Serine/threonine-protein kinase ULK3 | 0.645 | 0.117 |
| seq.12438.127 | DNA-3-methyladenine glycosylase | 0.485 | 0.552 |
| seq.12439.67 | Interferon regulatory factor 9 | 0.391 | 0.000 |
| seq.12442.4 | Rho-related GTP-binding protein RhoD | 0.949 | 0.281 |
| seq.12444.39 | Nuclear receptor subfamily 5 group A member 2 | 0.989 | 0.851 |
| seq.12445.50 | Ankyrin repeat domain-containing protein 27 | 0.549 | 0.707 |
| seq.12446.49 | Glutathione S-transferase A1 | 0.351 | 0.174 |
| seq.12448.246 | Glycylpeptide N-tetradecanoyltransferase 1 | 0.000 | 0.000 |
| seq.12449.16 | Peptidyl-prolyl cis-trans isomerase H | 0.205 | 0.000 |
| seq.12450.42 | Phosphomevalonate kinase | 0.225 | 0.000 |
| seq.12451.62 | Transcription regulator protein BACH1 | 0.343 | 0.500 |
| seq.12452.32 | Histone-lysine N-methyltransferase SUV420H2 | 0.525 | 0.308 |
| seq.12453.161 | Nuclear RNA export factor 1 | 0.398 | 0.671 |
| seq.12454.105 | N-terminal Xaa-Pro-Lys N-methyltransferase 1 | 0.772 | 0.328 |
| seq.12455.48 | Myoneurin | 0.309 | 0.593 |
| seq.12456.5 | 6-phosphofructo-2-kinase/fructose-2,6-bisphosphatase 3 | 0.505 | 0.741 |
| seq.12457.10 | 1,2-dihydroxy-3-keto-5-methylthiopentene dioxygenase | 0.442 | 0.765 |
| seq.12458.79 | Calcineurin B homologous protein 1 | 0.896 | 0.121 |
| seq.12459.13 | Pleckstrin homology domain-containing family A member 1 | 0.400 | 0.293 |
| seq.12460.18 | Proteasome subunit alpha type-7 | 0.499 | 0.184 |
| seq.12461.8 | NAD-dependent protein deacylase sirtuin-5, mitochondrial | 0.960 | 0.771 |
| seq.12462.20 | Histone-lysine N-methyltransferase SETMAR | 0.518 | 0.111 |
| seq.12463.7 | Kelch-like protein 13 | 0.561 | 0.538 |
| seq.12466.7 | Heterogeneous nuclear ribonucleoprotein A1 | 0.003 | 0.177 |
| seq.12469.19 | Microtubule-associated protein RP/EB family member 1 | 0.018 | 0.000 |
| seq.12471.47 | Double-stranded RNA-binding protein Staufen homolog 1 | 0.516 | 0.223 |
| seq.12473.48 | BTB/POZ domain-containing protein KCTD5 | 0.554 | 0.298 |
| seq.12475.48 | Chloride intracellular channel protein 5 | 0.547 | 0.293 |
| seq.12476.50 | Fructose-2,6-bisphosphatase TIGAR | 0.486 | 0.876 |
| seq.12477.42 | Translin | 0.401 | 0.820 |
| seq.12478.15 | 60S ribosomal protein L30 | 0.554 | 0.522 |
| seq.12479.50 | cAMP-dependent protein kinase type I-beta regulatory subunit | 0.729 | 0.837 |
| seq.12480.9 | OTU domain-containing protein 5 | 0.374 | 0.272 |
| seq.12482.5 | Nucleosome-remodeling factor subunit BPTF | 0.968 | 0.000 |
| seq.12483.62 | Nuclear receptor ROR-beta | 0.956 | 0.962 |
| seq.12484.67 | Phosphatidylinositol transfer protein beta isoform | 0.252 | 0.461 |
| seq.12486.8 | Glutaredoxin-2, mitochondrial | 0.590 | 0.000 |
| seq.12488.9 | Malignant T-cell-amplified sequence 1 | 0.901 | 0.716 |
| seq.12490.92 | Ragulator complex protein LAMTOR3 | 0.755 | 0.369 |
| seq.12491.23 | Chloride intracellular channel protein 4 | 0.000 | 0.000 |
| seq.12493.42 | Ubiquitin thioesterase OTUB2 | 0.528 | 0.528 |
| seq.12494.99 | Gamma-aminobutyric acid receptor-associated protein-like 2 | 0.000 | 0.059 |
| seq.12497.29 | Tudor-interacting repair regulator protein | 0.622 | 0.000 |
| seq.12498.12 | Tax1-binding protein 3 | 0.228 | 0.000 |
| seq.12499.108 | Endophilin-A1 | 0.000 | 0.000 |
| seq.12500.88 | SUMO-activating enzyme subunit 2 | 0.163 | 0.000 |
| seq.12501.10 | Tubulin-specific chaperone A | 0.897 | 0.773 |
| seq.12503.5 | Ovarian cancer G-protein coupled receptor 1 | 0.602 | 0.903 |
| seq.12504.26 | Leiomodin-1 | 0.666 | 0.904 |
| seq.12507.16 | Inositol-trisphosphate 3-kinase C | 0.840 | 0.633 |
| seq.12508.9 | Charged multivesicular body protein 3 | 0.040 | 0.000 |
| seq.12509.115 | COMM domain-containing protein 1 | 0.945 | 0.937 |
| seq.12510.3 | Signal-transducing adaptor protein 1 | 0.716 | 0.270 |
| seq.12511.83 | Solute carrier family 41 member 2 | 0.879 | 0.256 |
| seq.12513.8 | Glycolipid transfer protein | 0.956 | 0.925 |
| seq.12514.16 | tRNA (guanine-N(7)-)-methyltransferase | 0.679 | 0.356 |
| seq.12515.45 | Uridine-cytidine kinase 2 | 0.945 | 0.099 |
| seq.12516.13 | Transcriptional enhancer factor TEF-3 | 0.289 | 0.034 |
| seq.12517.52 | Programmed cell death protein 5 | 0.230 | 0.151 |
| seq.12518.289 | Protein polybromo-1 | 0.755 | 0.763 |
| seq.12521.3 | Cyclin-dependent kinase 4 inhibitor C | 0.398 | 0.407 |
| seq.12522.6 | UV excision repair protein RAD23 homolog B | 0.160 | 0.000 |
| seq.12524.18 | Diamine acetyltransferase 2 | 0.864 | 0.707 |
| seq.12527.50 | Thyroid hormone receptor alpha | 0.553 | 0.491 |
| seq.12528.40 | ATPase WRNIP1 | 0.241 | 0.159 |
| seq.12529.32 | Inactive peptidyl-prolyl cis-trans isomerase FKBP6 | 0.186 | 0.753 |
| seq.12530.14 | Cyclin-dependent kinases regulatory subunit 1 | 0.388 | 0.713 |
| seq.12531.5 | Endogenous retrovirus group V member 1 Env polyprotein | 0.690 | 0.650 |
| seq.12532.28 | Ubiquitin-conjugating enzyme E2 R1 | 0.667 | 0.830 |
| seq.12533.135 | Cytohesin-2 | 0.502 | 0.862 |
| seq.12534.10 | Calcium-binding and coiled-coil domain-containing protein 2 | 0.513 | 0.874 |
| seq.12535.2 | DNA repair protein XRCC1 | 0.905 | 0.280 |
| seq.12536.46 | Myotubularin-related protein 6 | 0.414 | 0.156 |
| seq.12537.88 | Transcriptional activator protein Pur-alpha | 0.365 | 0.048 |
| seq.12538.19 | Regulator of G-protein signaling 7 | 0.653 | 0.442 |
| seq.12540.25 | Rho-related GTP-binding protein RhoG | 0.479 | 0.329 |
| seq.12543.76 | Zinc finger protein 560 | 0.905 | 0.908 |
| seq.12546.1 | Smoothelin | 0.189 | 0.000 |
| seq.12548.75 | Ras-related GTP-binding protein C | 0.789 | 0.575 |
| seq.12549.33 | Hematopoietic prostaglandin D synthase | 0.823 | 0.756 |
| seq.12551.3 | E3 ubiquitin-protein ligase Itchy homolog | 0.961 | 0.921 |
| seq.12553.5 | Serine/threonine-protein kinase VRK1 | 0.325 | 0.448 |
| seq.12554.10 | DNA repair protein RAD51 homolog 4 | 0.637 | 0.257 |
| seq.12556.7 | Ubiquitin-conjugating enzyme E2 C | 0.770 | 0.539 |
| seq.12557.18 | RNA-binding protein Nova-1 | 0.962 | 0.799 |
| seq.12558.3 | Ubiquitin-associated and SH3 domain-containing protein B | 0.104 | 0.000 |
| seq.12560.9 | 5'(3')-deoxyribonucleotidase, cytosolic type | 0.687 | 0.424 |
| seq.12562.1 | Serine/threonine-protein kinase N1 | 0.412 | 0.455 |
| seq.12563.2 | Tumor necrosis factor alpha-induced protein 8 | 0.036 | 0.000 |
| seq.12564.9 | 60S ribosome subunit biogenesis protein NIP7 homolog | 0.786 | 0.669 |
| seq.12568.14 | Kelch-like ECH-associated protein 1 | 0.647 | 0.538 |
| seq.12569.25 | T-complex protein 1 subunit epsilon | 0.449 | 0.126 |
| seq.12571.14 | ADP-ribosylation factor-like protein 3 | 0.137 | 0.000 |
| seq.12572.236 | Embryonal Fyn-associated substrate | 0.459 | 0.694 |
| seq.12573.80 | Tripartite motif-containing protein 3 | 0.501 | 0.000 |
| seq.12574.36 | Endothelin-2 | 0.807 | 0.719 |
| seq.12575.30 | C-1-tetrahydrofolate synthase, cytoplasmic | 0.524 | 0.034 |
| seq.12576.21 | Melanoma-associated antigen 3 | 0.763 | 0.706 |
| seq.12577.100 | Flap endonuclease 1 | 0.684 | 0.796 |
| seq.12578.13 | ADP-ribosylation factor 3 | 0.970 | 0.769 |
| seq.12580.7 | Proteasome subunit beta type-5 | 0.899 | 0.554 |
| seq.12581.39 | Inositol monophosphatase 2 | 0.446 | 0.722 |
| seq.12583.77 | Serine/threonine-protein kinase A-Raf | 0.561 | 0.216 |
| seq.12585.39 | DNA excision repair protein ERCC-1 | 0.720 | 0.593 |
| seq.12587.65 | ADP-ribosylation factor-like protein 2 | 0.793 | 0.319 |
| seq.12591.27 | Oligophrenin-1 | 0.296 | 0.000 |
| seq.12593.33 | p53 and DNA damage-regulated protein 1 | 0.678 | 0.377 |
| seq.12594.5 | Grancalcin | 0.575 | 0.583 |
| seq.12595.11 | Tropomodulin-1 | 0.840 | 0.070 |
| seq.12597.68 | Autophagy protein 5 | 0.549 | 0.510 |
| seq.12599.10 | Fermitin family homolog 3 | 0.190 | 0.000 |
| seq.12603.87 | Polyadenylate-binding protein 4 | 0.000 | 0.000 |
| seq.12604.16 | Polycomb protein SCMH1 | 0.696 | 0.834 |
| seq.12605.1 | Exosome complex component RRP40 | 0.945 | 0.841 |
| seq.12612.37 | Proteasome subunit beta type-1 | 0.557 | 0.318 |
| seq.12616.45 | Nuclear receptor-binding protein | 0.595 | 0.146 |
| seq.12617.2 | Serine/threonine-protein kinase 24 | 0.000 | 0.000 |
| seq.12618.50 | Aldo-keto reductase family 1 member C1 | 0.189 | 0.563 |
| seq.12619.14 | Protein phosphatase 1A | 0.180 | 0.954 |
| seq.12620.3 | Septin-11 | 0.064 | 0.092 |
| seq.12621.55 | Serine/threonine-protein phosphatase 2A 65 kDa regulatory subunit A alpha isoform | 0.336 | 0.067 |
| seq.12622.96 | Histone-lysine N-methyltransferase ASH1L | 0.834 | 0.156 |
| seq.12623.84 | Protein Jumonji | 0.889 | 0.211 |
| seq.12625.138 | Kelch-like protein 7 | 0.798 | 0.947 |
| seq.12626.6 | Sentrin-specific protease 7 | 0.577 | 0.561 |
| seq.12627.97 | Ubiquitin-like modifier-activating enzyme ATG7 | 0.000 | 0.347 |
| seq.12628.31 | LanC-like protein 2 | 0.320 | 0.214 |
| seq.12630.8 | Arfaptin-2 | 0.812 | 0.747 |
| seq.12632.14 | Arylamine N-acetyltransferase 1 | 0.238 | 0.561 |
| seq.12633.3 | Tyrosine-protein phosphatase non-receptor type 9 | 0.972 | 0.957 |
| seq.12634.79 | Breast cancer anti-estrogen resistance protein 3:Guanine Nucleotide Exchange Factor Domain | 0.654 | 0.709 |
| seq.12635.9 | tRNA (cytosine(38)-C(5))-methyltransferase | 0.578 | 0.468 |
| seq.12636.113 | N-lysine methyltransferase SMYD2 | 0.679 | 0.179 |
| seq.12637.7 | Carnitine O-acetyltransferase | 0.235 | 0.000 |
| seq.12641.3 | Type II inositol 1,4,5-trisphosphate 5-phosphatase | 0.000 | 0.000 |
| seq.12643.4 | Beta-arrestin-1 | 0.000 | 0.000 |
| seq.12644.63 | Adenylosuccinate synthetase isozyme 2 | 0.905 | 0.445 |
| seq.12646.2 | Ribulose-phosphate 3-epimerase | 0.321 | 0.033 |
| seq.12647.52 | Histone-lysine N-methyltransferase SETD2 | 0.725 | 0.164 |
| seq.12649.80 | Malate dehydrogenase, mitochondrial | 0.234 | 0.000 |
| seq.12650.43 | Guanine nucleotide-binding protein G(k) subunit alpha | 0.115 | 0.364 |
| seq.12651.21 | [Pyruvate dehydrogenase (acetyl-transferring)] kinase isozyme 2, mitochondrial | 0.904 | 0.000 |
| seq.12652.37 | Adenosylhomocysteinase 2 | 0.121 | 0.000 |
| seq.12653.13 | Casein kinase I isoform gamma-2 | 0.971 | 0.597 |
| seq.12655.30 | Beta-soluble NSF attachment protein | 0.000 | 0.831 |
| seq.12656.1 | Kinesin light chain 1 | 0.000 | 0.000 |
| seq.12657.2 | GDP-L-fucose synthase | 0.874 | 0.630 |
| seq.12658.72 | Pantothenate kinase 3 | 0.000 | 0.477 |
| seq.12659.13 | Obg-like ATPase 1 | 0.263 | 0.000 |
| seq.12661.44 | Gamma-aminobutyric acid receptor-associated protein-like 1 | 0.293 | 0.514 |
| seq.12662.82 | Delta(3,5)-Delta(2,4)-dienoyl-CoA isomerase, mitochondrial | 0.499 | 0.250 |
| seq.12663.1 | Thiosulfate sulfurtransferase | 0.691 | 0.669 |
| seq.12664.19 | Tyrosine-protein phosphatase non-receptor type 13 | 0.674 | 0.821 |
| seq.12665.16 | Interleukin enhancer-binding factor 2 | 0.843 | 0.697 |
| seq.12667.2 | Guanine deaminase | 0.200 | 0.338 |
| seq.12668.7 | Vacuolar protein sorting-associated protein 4B | 0.166 | 0.000 |
| seq.12669.30 | E3 ubiquitin-protein ligase HECW1 | 0.863 | 0.000 |
| seq.12670.15 | Cell cycle checkpoint protein RAD1 | 0.985 | 0.880 |
| seq.12671.35 | Sulfotransferase family cytosolic 1B member 1 | 0.522 | 0.811 |
| seq.12675.14 | Aspartate--tRNA ligase, cytoplasmic | 0.000 | 0.000 |
| seq.12676.1 | Protein kinase C and casein kinase substrate in neurons protein 1 | 0.530 | 0.000 |
| seq.12677.164 | Protein flightless-1 homolog | 0.177 | 0.000 |
| seq.12678.66 | U1 small nuclear ribonucleoprotein A | 0.110 | 0.298 |
| seq.12681.63 | Ubiquitin carboxyl-terminal hydrolase 21 | 0.657 | 0.253 |
| seq.12682.5 | Kynurenine--oxoglutarate transaminase 3 | 0.935 | 0.181 |
| seq.12683.156 | Dihydropyrimidinase-related protein 5 | 0.612 | 0.373 |
| seq.12684.5 | Adseverin | 0.508 | 0.433 |
| seq.12685.57 | Homer protein homolog 2 | 0.542 | 0.000 |
| seq.12686.15 | 3-mercaptopyruvate sulfurtransferase | 0.436 | 0.580 |
| seq.12687.2 | 2,4-dienoyl-CoA reductase, mitochondrial | 0.417 | 0.000 |
| seq.12688.115 | Ribosomal protein S6 kinase alpha-6 | 0.159 | 0.133 |
| seq.12689.56 | Actin-related protein 2/3 complex subunit 1B | 0.111 | 0.214 |
| seq.12690.33 | Septin-10 | 0.541 | 0.567 |
| seq.12691.44 | Serine hydroxymethyltransferase, mitochondrial | 0.285 | 0.000 |
| seq.12692.56 | Histone-lysine N-methyltransferase, H3 lysine-79 specific | 0.721 | 0.663 |
| seq.12693.2 | SH3 domain-binding glutamic acid-rich-like protein | 0.044 | 0.276 |
| seq.12694.28 | Probable dimethyladenosine transferase | 0.948 | 0.463 |
| seq.12695.62 | Kelch-like protein 12 | 0.926 | 0.426 |
| seq.12696.166 | Protein arginine N-methyltransferase 3 | 0.844 | 0.734 |
| seq.12697.30 | Phosphatidylinositol 5-phosphate 4-kinase type-2 alpha | 0.060 | 0.000 |
| seq.12698.72 | Importin subunit alpha-3 | 0.875 | 0.121 |
| seq.12699.28 | T-complex protein 1 subunit theta | 0.439 | 0.038 |
| seq.12700.9 | ATP-citrate synthase | 0.634 | 0.872 |
| seq.12701.1 | Eukaryotic translation initiation factor 1b | 0.000 | 0.000 |
| seq.12702.13 | E3 ubiquitin-protein ligase pellino homolog 2 | 0.882 | 0.783 |
| seq.12703.6 | Serine/threonine-protein kinase Nek7 | 0.093 | 0.075 |
| seq.12704.26 | SH2 domain-containing protein 3C | 0.025 | 0.839 |
| seq.12705.9 | Probable E3 ubiquitin-protein ligase HERC1 | 0.544 | 0.432 |
| seq.12706.2 | Serine/threonine-protein kinase MRCK alpha | 0.693 | 0.753 |
| seq.12707.26 | Dihydropyrimidinase-related protein 3 | 0.593 | 0.000 |
| seq.12708.91 | Lethal(3)malignant brain tumor-like protein 2 | 0.470 | 0.167 |
| seq.12709.63 | Histone H1x | 0.280 | 0.553 |
| seq.12711.19 | Gap junction alpha-8 protein | 0.726 | 0.845 |
| seq.12712.9 | High mobility group protein 20A | 0.824 | 0.906 |
| seq.12713.365 | Regulator of G-protein signaling 19 | 0.364 | 0.398 |
| seq.12714.38 | AP-1 complex subunit gamma-like 2 | 0.344 | 0.000 |
| seq.12715.30 | mRNA-decapping enzyme 1B | 0.117 | 0.017 |
| seq.12716.3 | Zinc finger protein 175 | 0.312 | 0.927 |
| seq.12717.65 | TOX high mobility group box family member 3 | 0.447 | 0.331 |
| seq.12718.43 | Peptidyl-prolyl cis-trans isomerase NIMA-interacting 4 | 0.244 | 0.000 |
| seq.12720.71 | Ubiquilin-4 | 0.050 | 0.000 |
| seq.12721.4 | Tax1-binding protein 1 | 0.980 | 0.916 |
| seq.12724.81 | Cold-inducible RNA-binding protein | 0.000 | 0.000 |
| seq.12726.3 | Regulatory factor X-associated protein | 0.455 | 0.502 |
| seq.12727.7 | Prostaglandin F2 receptor negative regulator | 0.736 | 0.388 |
| seq.12729.12 | Proteasome assembly chaperone 3 | 0.666 | 0.449 |
| seq.12730.3 | Kinetochore protein NDC80 homolog | 0.000 | 0.660 |
| seq.12731.12 | Pleckstrin homology domain-containing family A member 7 | 0.568 | 0.753 |
| seq.12732.13 | MAGUK p55 subfamily member 7 | 0.943 | 0.771 |
| seq.12734.112 | Kinesin-like protein KIF22 | 0.561 | 0.788 |
| seq.12735.39 | Cold shock domain-containing protein E1 | 0.582 | 0.429 |
| seq.12737.12 | Plastin-1 | 0.848 | 0.901 |
| seq.12738.43 | Nischarin | 0.273 | 0.587 |
| seq.12740.55 | Protein FEV | 0.378 | 0.000 |
| seq.12742.160 | Transmembrane protein 8B | 0.789 | 0.951 |
| seq.12743.18 | BAG family molecular chaperone regulator 5 | 0.942 | 0.693 |
| seq.12746.4 | Cytohesin-4 | 0.528 | 0.458 |
| seq.12747.89 | RNA-binding protein 3 | 0.243 | 0.000 |
| seq.12748.6 | Bromodomain testis-specific protein | 0.935 | 0.583 |
| seq.12750.9 | Integrin beta-2 | 0.912 | 0.348 |
| seq.12751.26 | Zinc fingers and homeoboxes protein 1 | 0.797 | 0.525 |
| seq.12753.6 | Amyloid beta A4 precursor protein-binding family B member 2:Phosphotyrosine Interaction Domain 1 | 0.533 | 0.368 |
| seq.12754.14 | Cold shock domain-containing protein C2 | 0.677 | 0.949 |
| seq.12756.3 | Transcription regulator protein BACH2 | 0.690 | 0.900 |
| seq.12758.47 | Glutamate receptor ionotropic, delta-2 | 0.807 | 0.800 |
| seq.12759.47 | Interleukin enhancer-binding factor 3 | 0.510 | 0.852 |
| seq.12760.34 | Zinc finger protein 774 | 0.395 | 0.664 |
| seq.12761.12 | Amyloid beta A4 precursor protein-binding family B member 2:Phosphotyrosine Interaction Domain 2 | 0.921 | 0.440 |
| seq.12763.69 | Zinc finger protein 334 | 0.355 | 0.863 |
| seq.12764.3 | Engulfment and cell motility protein 1 | 0.789 | 0.939 |
| seq.12766.33 | Probable G-protein coupled receptor 142 | 0.911 | 0.936 |
| seq.12768.3 | Protein phosphatase 1 regulatory subunit 3B | 0.945 | 0.731 |
| seq.12771.19 | Zinc finger protein 180 | 0.968 | 0.409 |
| seq.12772.8 | Nuclear pore complex protein Nup98-Nup96 | 0.962 | 0.716 |
| seq.12774.12 | Potassium/sodium hyperpolarization-activated cyclic nucleotide-gated channel 1 | 0.651 | 0.439 |
| seq.12775.6 | High mobility group protein B3 | 0.196 | 0.532 |
| seq.12777.11 | Splicing factor 1 | 0.516 | 0.459 |
| seq.12779.30 | PR domain zinc finger protein 4 | 0.397 | 0.578 |
| seq.12781.2 | RISC-loading complex subunit TARBP2 | 0.807 | 0.249 |
| seq.12783.29 | Heterogeneous nuclear ribonucleoprotein M | 0.473 | 0.548 |
| seq.12784.10 | Amyloid beta A4 precursor protein-binding family B member 3:Phosphotyrosine Interaction Domain 1,Isoform II | 0.617 | 0.971 |
| seq.12785.49 | Transcriptional regulator Kaiso | 0.707 | 0.000 |
| seq.12786.61 | Glycerophosphocholine phosphodiesterase GPCPD1 | 0.703 | 0.744 |
| seq.12787.47 | Zinc finger protein 134 | 0.842 | 0.874 |
| seq.12788.6 | SAGA-associated factor 29 homolog | 0.553 | 0.344 |
| seq.12790.10 | Coiled-coil domain-containing protein 51 | 0.317 | 0.359 |
| seq.12793.4 | Piwi-like protein 1 | 0.832 | 0.860 |
| seq.12794.6 | NACHT, LRR and PYD domains-containing protein 4 | 0.785 | 0.476 |
| seq.12795.2 | Zinc finger protein 566 | 0.445 | 0.467 |
| seq.12796.44 | Diphosphoinositol polyphosphate phosphohydrolase 1 | 0.090 | 0.121 |
| seq.12798.46 | Electroneutral sodium bicarbonate exchanger 1 | 0.527 | 0.758 |
| seq.12799.65 | DnaJ homolog subfamily C member 27 | 0.483 | 0.846 |
| seq.12800.5 | Tubulin polymerization-promoting protein family member 2 | 0.579 | 0.624 |
| seq.12801.33 | Interferon regulatory factor 2 | 0.820 | 0.733 |
| seq.12803.9 | Zinc finger protein 329 | 0.572 | 0.180 |
| seq.12804.5 | Glucocorticoid modulatory element-binding protein 2 | 0.804 | 0.381 |
| seq.12807.89 | Rho GTPase-activating protein 30 | 0.501 | 0.692 |
| seq.12808.103 | Calcium-regulated heat-stable protein 1 | 0.533 | 0.389 |
| seq.12811.55 | Zinc finger protein 415 | 0.850 | 0.577 |
| seq.12812.25 | Acylphosphatase-2 | 0.382 | 0.000 |
| seq.12813.18 | EH domain-binding protein 1 | 0.725 | 0.319 |
| seq.12814.17 | RNA-binding protein 40 | 0.591 | 0.311 |
| seq.12815.9 | Isoleucine--tRNA ligase, cytoplasmic | 0.621 | 0.892 |
| seq.12817.1 | GTP-binding protein GEM | 0.390 | 0.804 |
| seq.12818.159 | Urea transporter 2 | 0.734 | 0.618 |
| seq.12820.1 | GRB2-related adapter protein | 0.375 | 0.000 |
| seq.12821.6 | NACHT, LRR and PYD domains-containing protein 10 | 0.967 | 0.901 |
| seq.12822.34 | Amyloid beta A4 precursor protein-binding family B member 1:Phosphotyrosine Interaction Domain 1 | 0.596 | 0.782 |
| seq.12825.18 | DCC-interacting protein 13-alpha | 0.838 | 0.797 |
| seq.12826.5 | Sodium/iodide cotransporter | 0.981 | 0.897 |
| seq.12827.37 | Regulator of G-protein signaling 3 | 0.502 | 0.796 |
| seq.12830.4 | Histone deacetylase complex subunit SAP18 | 0.662 | 0.451 |
| seq.12831.21 | Calcineurin B homologous protein 3 | 0.426 | 0.305 |
| seq.12832.10 | Set1/Ash2 histone methyltransferase complex subunit ASH2 | 0.421 | 0.156 |
| seq.12834.3 | Potassium voltage-gated channel subfamily F member 1 | 0.527 | 0.325 |
| seq.12835.101 | Pyrin domain-containing protein 1 | 0.783 | 0.840 |
| seq.12838.28 | Dual specificity protein phosphatase 15 | 0.987 | 0.935 |
| seq.12842.43 | Syntaxin-10 | 0.949 | 0.635 |
| seq.12843.6 | Zinc finger protein 410 | 0.853 | 0.730 |
| seq.12844.10 | BAG family molecular chaperone regulator 4 | 0.621 | 0.517 |
| seq.12845.18 | Sorting nexin-17 | 0.692 | 0.111 |
| seq.12846.3 | F-box/LRR-repeat protein 5 | 0.311 | 0.106 |
| seq.12847.27 | mRNA-capping enzyme | 0.575 | 0.917 |
| seq.12848.9 | Rho guanine nucleotide exchange factor 2 | 0.629 | 0.888 |
| seq.12849.25 | GSK3-beta interaction protein | 0.840 | 0.572 |
| seq.12851.5 | DNA-directed DNA/RNA polymerase mu | 0.658 | 0.836 |
| seq.12853.112 | Tropomodulin-2 | 0.160 | 0.000 |
| seq.12854.3 | Transcriptional enhancer factor TEF-5 | 0.993 | 0.862 |
| seq.12855.16 | Cas scaffolding protein family member 4 | 0.462 | 0.000 |
| seq.12856.14 | Transmembrane protein 237 | 0.944 | 0.690 |
| seq.12859.33 | Enoyl-CoA delta isomerase 2, mitochondrial | 0.475 | 0.000 |
| seq.12860.7 | cAMP-regulated phosphoprotein 21 | 0.684 | 0.686 |
| seq.12861.13 | Tropomodulin-3 | 0.080 | 0.000 |
| seq.12862.14 | Enhancer of filamentation 1 | 0.246 | 0.000 |
| seq.12864.9 | Magnesium transporter NIPA4 | 0.651 | 0.866 |
| seq.12867.40 | Dynein light chain Tctex-type 3 | 0.937 | 0.174 |
| seq.12869.68 | Probable ATP-dependent RNA helicase DDX6 | 0.000 | 0.000 |
| seq.12871.10 | Twinfilin-1 | 0.000 | 0.333 |
| seq.12872.35 | Cyclic nucleotide-gated olfactory channel | 0.758 | 0.404 |
| seq.12873.11 | Leukosialin | 0.734 | 0.492 |
| seq.12875.28 | Ubiquitin domain-containing protein 2 | 0.656 | 0.527 |
| seq.12876.39 | Sperm flagellar protein 1 | 0.932 | 0.037 |
| seq.12878.60 | Oxysterol-binding protein-related protein 11 | 0.891 | 0.956 |
| seq.12879.5 | Retinoblastoma-like protein 1 | 0.693 | 0.596 |
| seq.12880.1 | Synaptic vesicle glycoprotein 2A | 0.859 | 0.437 |
| seq.12881.17 | Retinol dehydrogenase 16 | 0.645 | 0.776 |
| seq.12882.7 | Poly [ADP-ribose] polymerase 11 | 0.518 | 0.199 |
| seq.12885.42 | Nuclear receptor subfamily 1 group D member 2 | 0.365 | 0.540 |
| seq.12888.18 | Histone deacetylase complex subunit SAP30 | 0.954 | 0.723 |
| seq.12891.1 | MICAL-like protein 2 | 0.018 | 0.595 |
| seq.12892.10 | Synaptotagmin-like protein 1 | 0.681 | 0.603 |
| seq.12893.159 | Gamma-interferon-inducible protein 16:Isoform 2, Hematopoietic expression, interferon-inducible nature, and nuclear localization 2 | 0.121 | 0.499 |
| seq.12894.3 | PAX-interacting protein 1 | 0.771 | 0.580 |
| seq.12895.28 | Diacylglycerol kinase beta | 0.509 | 0.191 |
| seq.12904.180 | cubilin | 0.466 | 0.725 |
| seq.12916.3 | Protein p13 MTCP-1 | 0.514 | 0.394 |
| seq.12923.51 | Keratin, type I cytoskeletal 17 | 0.235 | 0.296 |
| seq.12925.105 | Axin-2 | 0.408 | 0.725 |
| seq.12931.16 | Mineralocorticoid receptor | 0.976 | 0.585 |
| seq.12933.17 | Ecto-NOX disulfide-thiol exchanger 1 | 0.477 | 0.233 |
| seq.12934.1 | E3 ISG15--protein ligase HERC5 | 0.455 | 0.601 |
| seq.12936.38 | Retinaldehyde-binding protein 1 | 0.262 | 0.789 |
| seq.12939.1 | DNA-directed RNA polymerases I and III subunit RPAC1 | 0.490 | 0.533 |
| seq.12940.35 | Aldehyde dehydrogenase family 3 member B1 | 0.796 | 0.535 |
| seq.12945.33 | Ras-related protein Rab-18 | 0.483 | 0.103 |
| seq.12954.71 | Peroxisome proliferator-activated receptor alpha | 0.306 | 0.641 |
| seq.12956.40 | KIF1-binding protein | 0.000 | 0.000 |
| seq.12957.62 | Cytochrome b-c1 complex subunit 7 | 0.645 | 0.455 |
| seq.12960.9 | Glucokinase | 0.000 | 0.178 |
| seq.12963.1 | Protein GPR107 | 0.213 | 0.649 |
| seq.12968.2 | Cysteine and glycine-rich protein 2 | 0.403 | 0.031 |
| seq.12970.35 | Double-stranded RNA-binding protein Staufen homolog 2 | 0.500 | 0.840 |
| seq.12975.11 | Keratin, type I cytoskeletal 20 | 0.409 | 0.578 |
| seq.12976.49 | Vinexin b | 0.648 | 0.696 |
| seq.12980.31 | Pre-mRNA-splicing factor RBM22 | 0.763 | 0.964 |
| seq.12986.12 | Protein lin-7 homolog C | 0.488 | 0.105 |
| seq.12987.12 | Serine/arginine-rich splicing factor 7 | 0.551 | 0.451 |
| seq.12988.49 | RNA-binding protein EWS | 0.269 | 0.620 |
| seq.12990.39 | Mitogen-activated protein kinase kinase kinase 3 | 0.565 | 0.941 |
| seq.12991.49 | Cullin-9 | 0.884 | 0.447 |
| seq.12993.21 | Nuclear protein localization protein 4 homolog | 0.106 | 0.000 |
| seq.13007.66 | Epididymal-specific lipocalin-10 | 0.545 | 0.449 |
| seq.13011.20 | 40S ribosomal protein S10 | 0.423 | 0.245 |
| seq.13013.41 | E3 ubiquitin-protein ligase parkin | 0.098 | 0.969 |
| seq.13022.20 | Tumor protein p53-inducible protein 11 | 0.812 | 0.446 |
| seq.13025.4 | Fibroblast growth factor receptor substrate 2 | 0.695 | 0.583 |
| seq.13027.20 | Chromobox protein homolog 7 | 0.916 | 0.281 |
| seq.13032.1 | Beclin-1 | 0.678 | 0.246 |
| seq.13039.1 | Nuclear inhibitor of protein phosphatase 1 | 0.608 | 0.848 |
| seq.13041.47 | TNF receptor-associated factor 4 | 0.263 | 0.210 |
| seq.13042.7 | Potassium voltage-gated channel subfamily A member 10 | 0.274 | 0.609 |
| seq.13043.157 | ATPase family AAA domain-containing protein 2 | 0.454 | 0.697 |
| seq.13044.5 | Tumor susceptibility gene 101 protein | 0.000 | 0.000 |
| seq.13053.6 | Sodium- and chloride-dependent neutral and basic amino acid transporter B(0+) | 0.562 | 0.516 |
| seq.13054.87 | Profilin-2 | 0.104 | 0.471 |
| seq.13055.53 | PH and SEC7 domain-containing protein 1 | 0.462 | 0.466 |
| seq.13056.18 | Orphan sodium- and chloride-dependent neurotransmitter transporter NTT5 | 0.935 | 0.873 |
| seq.13059.33 | Riboflavin kinase | 0.583 | 0.238 |
| seq.13062.4 | Glia maturation factor gamma | 0.302 | 0.069 |
| seq.13066.42 | Enhancer of mRNA-decapping protein 4 | 0.079 | 0.534 |
| seq.13067.5 | cGMP-dependent protein kinase 1, beta isozyme | 0.592 | 0.515 |
| seq.13068.139 | Copper chaperone for superoxide dismutase | 0.442 | 0.124 |
| seq.13073.14 | Acidic leucine-rich nuclear phosphoprotein 32 family member A | 0.252 | 0.326 |
| seq.13076.4 | Fragile X mental retardation syndrome-related protein 1 | 0.745 | 0.348 |
| seq.13078.3 | Centrin-2 | 0.000 | 0.238 |
| seq.13082.9 | Protein unc-45 homolog A | 0.017 | 0.000 |
| seq.13083.18 | Valine--tRNA ligase | 0.555 | 0.599 |
| seq.13085.18 | Glucagon-like peptide 1 receptor:Cytoplasmic domain | 0.564 | 0.030 |
| seq.13088.397 | Betacellulin | 0.713 | 0.571 |
| seq.13089.6 | Hypoxia-inducible factor 1-alpha | 0.863 | 0.963 |
| seq.13090.17 | Protein S100-A6 | 0.339 | 0.127 |
| seq.13093.6 | Secreted and transmembrane protein 1 | 0.630 | 0.652 |
| seq.13094.75 | R-spondin-3 | 0.730 | 0.454 |
| seq.13095.51 | Lithostathine-1-alpha | 0.727 | 0.646 |
| seq.13097.11 | Bcl-2-like protein 2 | 0.594 | 0.469 |
| seq.13098.93 | Vascular endothelial growth factor D | 0.670 | 0.388 |
| seq.13101.60 | Sclerostin | 0.505 | 0.295 |
| seq.13102.1 | Protein FAM3D | 0.816 | 0.742 |
| seq.13103.125 | Chorionic somatomammotropin hormone | 0.050 | 0.176 |
| seq.13104.32 | Ephrin-B1 | 0.832 | 0.694 |
| seq.13105.7 | Synaptosomal-associated protein 25 | 0.932 | 0.660 |
| seq.13107.9 | Ly6/PLAUR domain-containing protein 3 | 0.868 | 0.488 |
| seq.13109.82 | Neuronal growth regulator 1 | 0.618 | 0.275 |
| seq.13111.79 | B-cell lymphoma 6 protein | 0.525 | 0.823 |
| seq.13112.179 | Follistatin-related protein 1 | 0.488 | 0.290 |
| seq.13113.7 | Osteopontin | 0.759 | 0.645 |
| seq.13114.50 | Lumican | 0.471 | 0.596 |
| seq.13116.25 | CD177 antigen | 0.830 | 0.806 |
| seq.13117.232 | Choline/ethanolamine kinase | 0.214 | 0.910 |
| seq.13118.5 | SPARC-related modular calcium-binding protein 1 | 0.850 | 0.924 |
| seq.13119.26 | Protein Z-dependent protease inhibitor | 0.851 | 0.544 |
| seq.13122.19 | Leucine-rich repeat transmembrane protein FLRT2 | 0.528 | 0.397 |
| seq.13123.3 | Leucine-rich repeat transmembrane protein FLRT3:Extracellular domain | 0.850 | 0.461 |
| seq.13124.20 | Immunoglobulin superfamily containing leucine-rich repeat protein 2 | 0.672 | 0.250 |
| seq.13125.45 | Vitronectin | 0.974 | 0.978 |
| seq.13126.52 | Desmocollin-2 | 0.776 | 0.388 |
| seq.13129.40 | Low-density lipoprotein receptor | 0.940 | 0.692 |
| seq.13130.150 | Hexokinase-2 | 0.476 | 0.486 |
| seq.13131.5 | Hexokinase-1 | 0.304 | 0.569 |
| seq.13132.14 | Semaphorin-5A | 0.685 | 0.454 |
| seq.13133.73 | Latent-transforming growth factor beta-binding protein 4 | 0.465 | 0.443 |
| seq.13228.75 | E3 ubiquitin-protein ligase Mdm2 | 0.010 | 0.813 |
| seq.13229.20 | Protein Mdm4 | 0.033 | 0.917 |
| seq.13230.174 | Ig gamma-2, Kappa | 0.865 | 0.695 |
| seq.13231.90 | Ig gamma-4, Kappa | 0.887 | 0.341 |
| seq.13236.25 | Protein Wnt-3a | 0.870 | 0.500 |
| seq.13240.170 | SH3 and multiple ankyrin repeat domains protein 1 | 0.869 | 0.323 |
| seq.13242.134 | SH3 and multiple ankyrin repeat domains protein 3 | 0.692 | 0.086 |
| seq.13256.21 | SH3 and multiple ankyrin repeat domains protein 1 | 0.801 | 0.692 |
| seq.13268.45 | Protein Wnt-5a | 0.833 | 0.226 |
| seq.13374.4 | Beta-defensin 113 | 0.784 | 0.936 |
| seq.13375.48 | Synaptogyrin-3 | 0.699 | 0.809 |
| seq.13377.3 | Cytochrome b561 domain-containing protein 1 | 0.754 | 0.395 |
| seq.13378.80 | Glutaredoxin-like protein C5orf63 | 0.627 | 0.622 |
| seq.13381.49 | Beta-1,4-galactosyltransferase 1 | 0.526 | 0.531 |
| seq.13384.110 | Fumarate hydratase, mitochondrial | 0.791 | 0.000 |
| seq.13386.248 | E3 ubiquitin-protein ligase RNF34 | 0.319 | 0.147 |
| seq.13387.55 | ETS homologous factor | 0.926 | 0.880 |
| seq.13388.57 | Neuroendocrine convertase 1 | 0.779 | 0.565 |
| seq.13392.13 | Sodium/potassium-transporting ATPase subunit beta-1 | 0.224 | 0.425 |
| seq.13393.46 | Derlin-1 | 0.426 | 0.069 |
| seq.13397.88 | Beta-defensin 4A | 0.815 | 0.846 |
| seq.13399.33 | RELT-like protein 1 | 0.513 | 0.870 |
| seq.13403.5 | ETS translocation variant 5 | 0.028 | 0.095 |
| seq.13405.61 | Serine protease inhibitor Kazal-type 2 | 0.908 | 0.604 |
| seq.13406.161 | CMRF35-like molecule 6 | 0.842 | 0.799 |
| seq.13408.23 | WAP, Kazal, immunoglobulin, Kunitz and NTR domain-containing protein 2 | 0.800 | 0.342 |
| seq.13411.21 | E3 ubiquitin-protein ligase NRDP1 | 0.495 | 0.556 |
| seq.13412.5 | WAP four-disulfide core domain protein 6 | 0.818 | 0.957 |
| seq.13416.8 | Transmembrane protein 132D | 0.640 | 0.262 |
| seq.13421.17 | Protein kish-B | 0.401 | 0.342 |
| seq.13422.66 | Ecto-NOX disulfide-thiol exchanger 2 | 0.177 | 0.093 |
| seq.13423.94 | Redox-regulatory protein FAM213A | 0.166 | 0.608 |
| seq.13427.66 | Mannosyl-oligosaccharide 1,2-alpha-mannosidase IC | 0.659 | 0.480 |
| seq.13429.3 | WAP four-disulfide core domain protein 10A | 0.433 | 0.633 |
| seq.13430.50 | Urea transporter 1 | 0.009 | 0.202 |
| seq.13431.74 | Membrane protein FAM159A | 0.938 | 0.770 |
| seq.13432.9 | Protrudin:Cytoplasmic domain, region 1, isoform 6 | 0.817 | 0.782 |
| seq.13434.172 | Alpha-parvin | 0.237 | 0.380 |
| seq.13435.31 | Interleukin-20 receptor subunit beta | 0.789 | 0.895 |
| seq.13436.54 | Myc-associated zinc finger protein | 0.771 | 0.552 |
| seq.13438.115 | Chordin | 0.931 | 0.706 |
| seq.13439.6 | IQ domain-containing protein F3 | 0.461 | 0.547 |
| seq.13441.30 | Dixin | 0.000 | 0.930 |
| seq.13447.42 | Shadow of prion protein | 0.338 | 0.393 |
| seq.13449.25 | Splicing factor 3B subunit 4 | 0.540 | 0.741 |
| seq.13450.49 | Ubiquitin carboxyl-terminal hydrolase 8 | 0.059 | 0.203 |
| seq.13451.2 | Fibronectin type III domain-containing protein 4 | 0.914 | 0.586 |
| seq.13452.113 | Small integral membrane protein 13 | 0.406 | 0.268 |
| seq.13453.2 | 39S ribosomal protein L33, mitochondrial | 0.744 | 0.466 |
| seq.13457.33 | ETS-related transcription factor Elf-5 | 0.846 | 0.211 |
| seq.13459.30 | Torsin-4A | 0.367 | 0.379 |
| seq.13460.4 | Chondroadherin | 0.692 | 0.835 |
| seq.13463.1 | Peroxidasin homolog | 0.794 | 0.719 |
| seq.13464.8 | Nutritionally-regulated adipose and cardiac enriched protein homolog | 0.844 | 0.751 |
| seq.13465.5 | Calcipressin-1 | 0.460 | 0.721 |
| seq.13468.5 | Neuralized-like protein 4 | 0.000 | 0.303 |
| seq.13470.43 | Parathyroid hormone/parathyroid hormone-related peptide receptor | 0.919 | 0.414 |
| seq.13472.35 | Haloacid dehalogenase-like hydrolase domain-containing protein 2 | 0.137 | 0.020 |
| seq.13473.55 | Inositol-trisphosphate 3-kinase A | 0.157 | 0.059 |
| seq.13474.40 | Glutathione S-transferase kappa 1 | 0.032 | 0.000 |
| seq.13475.10 | Ubiquitin-conjugating enzyme E2 D4 | 0.410 | 0.000 |
| seq.13476.16 | DNA/RNA-binding protein KIN17 | 0.885 | 0.454 |
| seq.13477.65 | Leukotriene B4 receptor 1 | 0.550 | 0.000 |
| seq.13479.8 | Protein FAM171A2 | 0.000 | 0.103 |
| seq.13481.24 | Transcription elongation factor A protein 2 | 0.534 | 0.327 |
| seq.13482.14 | CCR4-NOT transcription complex subunit 1 | 0.146 | 0.000 |
| seq.13484.69 | Collagen alpha-1(I) chain:N-term propeptide | 0.942 | 0.752 |
| seq.13485.20 | Transmembrane protein 87B | 0.798 | 0.189 |
| seq.13486.9 | Polycystin-2:Cytoplasmic domain 1 | 0.753 | 0.589 |
| seq.13487.24 | Protein unc-93 homolog B1 | 0.896 | 0.877 |
| seq.13488.3 | Arfaptin-1 | 0.277 | 0.000 |
| seq.13490.1 | MAGUK p55 subfamily member 6 | 0.260 | 0.346 |
| seq.13491.40 | Retinal rod rhodopsin-sensitive cGMP 3',5'-cyclic phosphodiesterase subunit delta | 0.934 | 0.844 |
| seq.13492.44 | Stress-70 protein, mitochondrial | 0.702 | 0.715 |
| seq.13493.5 | Glutamate receptor ionotropic, delta-1 | 0.738 | 0.780 |
| seq.13494.6 | Ceramide synthase 5 | 0.239 | 0.000 |
| seq.13495.48 | Hydroxycarboxylic acid receptor 2 | 0.682 | 0.745 |
| seq.13496.19 | Hydroxymethylglutaryl-CoA synthase, cytoplasmic | 0.614 | 0.815 |
| seq.13497.34 | Eukaryotic translation initiation factor 3 subunit J | 0.373 | 0.000 |
| seq.13498.1 | Inositol oxygenase | 0.828 | 0.957 |
| seq.13499.30 | Coagulation Factor VIII | 0.181 | 0.192 |
| seq.13501.10 | Solute carrier family 35 member G2 | 0.245 | 0.621 |
| seq.13502.2 | Sodium-independent sulfate anion transporter | 0.753 | 0.401 |
| seq.13503.19 | PRA1 family protein 3 | 0.183 | 0.000 |
| seq.13504.147 | Heterogeneous nuclear ribonucleoprotein R | 0.248 | 0.342 |
| seq.13506.10 | tRNA-dihydrouridine(20) synthase [NAD(P)+]-like | 0.319 | 0.303 |
| seq.13507.51 | Tumor necrosis factor receptor type 1-associated DEATH domain protein | 0.678 | 0.101 |
| seq.13509.5 | Secretory carrier-associated membrane protein 5 | 0.018 | 0.674 |
| seq.13510.7 | Sarcoplasmic/endoplasmic reticulum calcium ATPase 3 | 0.518 | 0.281 |
| seq.13511.29 | DNA-binding protein SATB1 | 0.236 | 0.687 |
| seq.13512.28 | Heterogeneous nuclear ribonucleoprotein R | 0.563 | 0.022 |
| seq.13513.174 | E3 SUMO-protein ligase PIAS3 | 0.937 | 0.698 |
| seq.13514.121 | Ras-related protein Rab-35 | 0.547 | 0.501 |
| seq.13515.8 | Regulation of nuclear pre-mRNA domain-containing protein 1A | 0.125 | 0.448 |
| seq.13516.46 | ER membrane protein complex subunit 4 | 0.375 | 0.614 |
| seq.13517.3 | Patched domain-containing protein 3 | 0.958 | 0.735 |
| seq.13518.5 | Arf-GAP with SH3 domain, ANK repeat and PH domain-containing protein 2 | 0.153 | 0.784 |
| seq.13519.112 | Rho guanine nucleotide exchange factor 25 | 0.527 | 0.167 |
| seq.13522.20 | Visinin-like protein 1 | 0.781 | 0.868 |
| seq.13524.25 | Heparan-sulfate 6-O-sulfotransferase 2 | 0.147 | 0.385 |
| seq.13525.17 | Potassium voltage-gated channel subfamily G member 4 | 0.866 | 0.572 |
| seq.13526.5 | Lupus La protein:RNA recognition motif | 0.589 | 0.132 |
| seq.13529.39 | Nucleosome assembly protein 1-like 2 | 0.400 | 0.355 |
| seq.13530.5 | Urotensin-2 receptor | 0.485 | 0.282 |
| seq.13532.25 | Nuclear envelope phosphatase-regulatory subunit 1 | 0.857 | 0.952 |
| seq.13534.20 | Myomesin-2 | 0.857 | 0.638 |
| seq.13535.2 | Collagen type IV alpha-3-binding protein:PH domain | 0.841 | 0.000 |
| seq.13536.56 | DNA polymerase iota | 0.205 | 0.088 |
| seq.13539.131 | Small conductance calcium-activated potassium channel protein 1 | 0.297 | 0.285 |
| seq.13540.1 | G-protein coupled receptor 26 | 0.241 | 0.881 |
| seq.13541.1 | Myc target protein 1 | 0.633 | 0.741 |
| seq.13543.7 | Prostaglandin reductase 1 | 0.771 | 0.232 |
| seq.13544.9 | Rho GTPase-activating protein 45 | 0.095 | 0.000 |
| seq.13545.97 | Probable RNA-binding protein EIF1AD | 0.528 | 0.063 |
| seq.13547.5 | 5-hydroxytryptamine receptor 7 | 0.981 | 0.722 |
| seq.13548.53 | Phosphatidate phosphatase PPAPDC1A | 0.445 | 0.220 |
| seq.13549.15 | Disintegrin and metalloproteinase domain-containing protein 29 | 0.564 | 0.034 |
| seq.13552.7 | Switch-associated protein 70 | 0.335 | 0.847 |
| seq.13553.4 | DCN1-like protein 3 | 0.727 | 0.775 |
| seq.13554.78 | Replication initiator 1 | 0.809 | 0.453 |
| seq.13556.28 | 5-hydroxytryptamine receptor 2A | 0.462 | 0.512 |
| seq.13557.3 | Nectin-3 | 0.768 | 0.957 |
| seq.13561.5 | 5-hydroxytryptamine receptor 6 | 0.084 | 0.151 |
| seq.13563.259 | Acyl-CoA-binding domain-containing protein 7 | 0.938 | 0.697 |
| seq.13565.2 | Retinoblastoma-like protein 2 | 0.538 | 0.509 |
| seq.13566.2 | RNA polymerase II subunit A C-terminal domain phosphatase SSU72 | 0.639 | 0.888 |
| seq.13567.1 | Dihydropyrimidinase-related protein 2 | 0.887 | 0.952 |
| seq.13568.30 | 26S proteasome non-ATPase regulatory subunit 4 | 0.358 | 0.674 |
| seq.13570.43 | Vigilin | 0.754 | 0.640 |
| seq.13572.43 | 26S proteasome non-ATPase regulatory subunit 11 | 0.000 | 0.011 |
| seq.13573.5 | Actin-related protein 2/3 complex subunit 3 | 0.195 | 0.152 |
| seq.13574.50 | Rho-related GTP-binding protein Rho6 | 0.964 | 0.929 |
| seq.13575.40 | Segment polarity protein dishevelled homolog DVL-2 | 0.623 | 0.099 |
| seq.13576.15 | Glutathione S-transferase P | 0.050 | 0.978 |
| seq.13577.25 | Splicing factor U2AF 65 kDa subunit | 0.851 | 0.399 |
| seq.13578.98 | Actin-binding LIM protein 3 | 0.855 | 0.153 |
| seq.13580.2 | UDP-N-acetylhexosamine pyrophosphorylase | 0.926 | 0.237 |
| seq.13583.19 | Apoptotic protease-activating factor 1 | 0.741 | 0.000 |
| seq.13587.10 | Rac GTPase-activating protein 1 | 0.911 | 0.464 |
| seq.13588.11 | Annexin A9 | 0.885 | 0.924 |
| seq.13589.10 | Amyloid beta A4 precursor protein-binding family B member 3:Phosphotyrosine Interaction Domain 2, Isoform IV | 0.561 | 0.915 |
| seq.13590.1 | Oligoribonuclease, mitochondrial | 0.404 | 0.139 |
| seq.13591.31 | DNA primase small subunit | 0.980 | 0.602 |
| seq.13594.158 | ADP-ribosylation factor-binding protein GGA1 | 0.000 | 0.000 |
| seq.13595.20 | Thiamine-triphosphatase | 0.576 | 0.186 |
| seq.13596.3 | Ras-related protein Rab-27B | 0.250 | 0.252 |
| seq.13597.20 | Ras-related protein Rab-31 | 0.256 | 0.000 |
| seq.13599.15 | Rab9 effector protein with kelch motifs | 0.393 | 0.505 |
| seq.13602.6 | NHP2-like protein 1 | 0.000 | 0.735 |
| seq.13603.7 | Rieske domain-containing protein | 0.728 | 0.001 |
| seq.13604.27 | E3 ubiquitin-protein ligase NEURL1 | 0.838 | 0.218 |
| seq.13605.16 | Annexin A10 | 0.450 | 0.758 |
| seq.13609.11 | General transcription factor II-I | 0.601 | 0.813 |
| seq.13610.9 | Melanoma-associated antigen 10 | 0.845 | 0.441 |
| seq.13612.7 | Cytohesin-interacting protein | 0.893 | 0.793 |
| seq.13613.23 | Adenylate kinase isoenzyme 5 | 0.404 | 0.091 |
| seq.13614.6 | CREB-binding protein | 0.404 | 0.416 |
| seq.13615.60 | Cytoplasmic protein NCK2 | 0.000 | 0.000 |
| seq.13618.15 | Mitotic spindle assembly checkpoint protein MAD1 | 0.951 | 0.828 |
| seq.13620.10 | ATPase ASNA1 | 0.614 | 0.297 |
| seq.13621.31 | AP-2 complex subunit alpha-2 | 0.036 | 0.026 |
| seq.13622.16 | Serine/threonine-protein phosphatase 2A 56 kDa regulatory subunit alpha isoform | 0.000 | 0.000 |
| seq.13623.4 | Histone-lysine N-methyltransferase 2D | 0.553 | 0.205 |
| seq.13624.17 | NAD kinase | 0.657 | 0.599 |
| seq.13625.19 | Lupus La protein:HTH La-type RNA-binding domain | 0.091 | 0.496 |
| seq.13628.58 | Growth factor receptor-bound protein 14 | 0.000 | 0.341 |
| seq.13629.25 | Cysteine protease ATG4B | 0.859 | 0.640 |
| seq.13631.1 | Retinoblastoma-binding protein 5 | 0.467 | 0.383 |
| seq.13632.10 | Zyxin | 0.302 | 0.213 |
| seq.13634.209 | Pirin | 0.649 | 0.839 |
| seq.13636.20 | Nucleosome assembly protein 1-like 1 | 0.000 | 0.000 |
| seq.13639.101 | General vesicular transport factor p115 | 0.138 | 0.000 |
| seq.13640.5 | B-cell lymphoma 6 protein | 0.851 | 0.470 |
| seq.13642.90 | Interferon-induced protein with tetratricopeptide repeats 3 | 0.911 | 0.826 |
| seq.13644.30 | Hepatocyte growth factor-regulated tyrosine kinase substrate | 0.096 | 0.000 |
| seq.13645.14 | Pre-mRNA-splicing factor ATP-dependent RNA helicase PRP16 | 0.452 | 0.765 |
| seq.13650.11 | Kv channel-interacting protein 1 | 0.900 | 0.601 |
| seq.13651.54 | E3 ubiquitin-protein ligase ZFP91 | 0.810 | 0.539 |
| seq.13652.2 | TOM1-like protein 1 | 0.711 | 0.879 |
| seq.13653.335 | O-acetyl-ADP-ribose deacetylase MACROD1 | 0.412 | 0.482 |
| seq.13654.1 | Rho-associated protein kinase 2 | 0.094 | 0.000 |
| seq.13655.34 | Nucleolin | 0.676 | 0.946 |
| seq.13657.2 | Bifunctional polynucleotide phosphatase/kinase | 0.224 | 0.000 |
| seq.13658.31 | Platelet-derived growth factor C | 0.032 | 0.482 |
| seq.13659.36 | Manganese-transporting ATPase 13A1 | 0.559 | 0.011 |
| seq.13660.76 | Angiopoietin-2 | 0.698 | 0.563 |
| seq.13661.193 | Cystatin-D | 0.791 | 0.701 |
| seq.13663.2 | Interleukin-4 | 0.536 | 0.325 |
| seq.13665.35 | Serine/threonine-protein phosphatase 2A regulatory subunit B'' subunit alpha | 0.478 | 0.222 |
| seq.13666.222 | Carbonic anhydrase-related protein 10 | 0.732 | 0.452 |
| seq.13668.44 | Tyrosine-protein kinase receptor TYRO3 | 0.908 | 0.449 |
| seq.13669.6 | Fibroblast growth factor receptor 3:Extracellular domain | 0.276 | 0.171 |
| seq.13670.81 | Group IIE secretory phospholipase A2 | 0.586 | 0.726 |
| seq.13671.40 | Neutrophil elastase | 0.466 | 0.544 |
| seq.13672.3 | Heat shock 70 kDa protein 6 | 0.271 | 0.662 |
| seq.13673.21 | T-complex protein 1 subunit eta | 0.422 | 0.256 |
| seq.13676.46 | Inhibin beta B chain | 0.605 | 0.518 |
| seq.13678.169 | Complement factor D | 0.214 | 0.081 |
| seq.13680.3 | NGFI-A-binding protein 2 | 0.251 | 0.051 |
| seq.13681.173 | Casein kinase II subunit alpha' | 0.250 | 0.283 |
| seq.13682.47 | Macrophage colony-stimulating factor 1 receptor | 0.781 | 0.851 |
| seq.13683.18 | Chloride channel protein ClC-Kb | 0.397 | 0.033 |
| seq.13686.2 | Interleukin-5 receptor subunit alpha | 0.919 | 0.799 |
| seq.13687.5 | C-C motif chemokine 1 | 0.510 | 0.612 |
| seq.13688.2 | Calcyphosin-like protein | 0.677 | 0.420 |
| seq.13689.2 | Ornithine decarboxylase | 0.684 | 0.345 |
| seq.13690.26 | Biglycan | 0.805 | 0.375 |
| seq.13691.10 | Sodium-coupled monocarboxylate transporter 1 | 0.620 | 0.024 |
| seq.13692.154 | WNT1-inducible-signaling pathway protein 1 | 0.753 | 0.629 |
| seq.13693.5 | Cerebral dopamine neurotrophic factor | 0.428 | 0.118 |
| seq.13694.24 | Cytokine receptor-like factor 2 | 0.507 | 0.671 |
| seq.13697.51 | Glycerol-3-phosphate dehydrogenase [NAD(+)], cytoplasmic | 0.557 | 0.800 |
| seq.13698.28 | Dyslexia-associated protein KIAA0319-like protein | 0.290 | 0.825 |
| seq.13699.6 | Prostate-specific antigen | 0.822 | 0.964 |
| seq.13700.10 | Annexin A2 | 0.371 | 0.406 |
| seq.13701.2 | C-X-C motif chemokine 13 | 0.463 | 0.648 |
| seq.13704.5 | Hydroxymethylglutaryl-CoA synthase, mitochondrial | 0.124 | 0.130 |
| seq.13706.12 | Interleukin-12 receptor subunit beta-1 | 0.440 | 0.163 |
| seq.13707.27 | SPARC-like protein 1 | 0.599 | 0.052 |
| seq.13708.56 | Kallikrein-8 | 0.683 | 0.502 |
| seq.13710.6 | Plasma protease C1 inhibitor | 0.707 | 0.591 |
| seq.13711.10 | Clathrin heavy chain 1 | 0.399 | 0.447 |
| seq.13712.104 | Granzyme A | 0.620 | 0.637 |
| seq.13713.164 | Vesicle-trafficking protein SEC22a | 0.962 | 0.000 |
| seq.13717.15 | Ficolin-2 | 0.856 | 0.725 |
| seq.13719.19 | Serine/threonine-protein kinase PAK 4 | 0.145 | 0.087 |
| seq.13720.95 | Myeloblastin | 0.853 | 0.753 |
| seq.13722.105 | Complement component C9 | 0.378 | 0.659 |
| seq.13723.6 | Interleukin-10 | 0.775 | 0.504 |
| seq.13724.27 | Fibroblast growth factor 19 | 0.356 | 0.253 |
| seq.13725.3 | Fibroblast growth factor 16 | 0.205 | 0.228 |
| seq.13726.4 | T-lymphocyte activation antigen CD80 | 0.487 | 0.309 |
| seq.13727.44 | Speckle-type POZ protein | 0.308 | 0.351 |
| seq.13728.19 | Endoplasmic reticulum resident protein 29 | 0.035 | 0.000 |
| seq.13729.26 | Sideroflexin-5 | 0.277 | 0.163 |
| seq.13730.18 | Dipeptidyl peptidase 1 | 0.780 | 0.634 |
| seq.13731.14 | Complement component C7 | 0.806 | 0.694 |
| seq.13732.79 | Cardiotrophin-1 | 0.624 | 0.682 |
| seq.13733.5 | Interleukin-12 subunit beta | 0.839 | 0.359 |
| seq.13734.22 | Interferon lambda-1 | 0.801 | 0.900 |
| seq.13735.1 | Rap1 GTPase-activating protein 1 | 0.415 | 0.691 |
| seq.13738.8 | Inhibin beta A chain | 0.704 | 0.330 |
| seq.13739.3 | Leucine-rich repeat transmembrane protein FLRT1 | 0.687 | 0.302 |
| seq.13740.51 | Secreted frizzled-related protein 3 | 0.806 | 0.592 |
| seq.13741.36 | Insulin-like growth factor-binding protein 1 | 0.677 | 0.597 |
| seq.13742.66 | Interleukin-22 | 0.527 | 0.489 |
| seq.13743.56 | Cullin-4B | 0.569 | 0.915 |
| seq.13744.37 | Interleukin-3 receptor subunit alpha | 0.788 | 0.858 |
| seq.13745.10 | Polycystin-2:Cytoplasmic domain 4 | 0.852 | 0.754 |
| seq.13747.9 | Carbonic anhydrase 6 | 0.873 | 0.730 |
| seq.13748.4 | C-C motif chemokine 8 | 0.760 | 0.766 |
| seq.13924.13 | Myocardial zonula adherens protein | 0.495 | 0.341 |
| seq.13926.1 | RecQ-mediated genome instability protein 1 | 0.377 | 0.283 |
| seq.13929.27 | Peroxisomal carnitine O-octanoyltransferase | 0.603 | 0.681 |
| seq.13930.3 | DNA dC->dU-editing enzyme APOBEC-3G | 0.314 | 0.000 |
| seq.13931.22 | 26S proteasome non-ATPase regulatory subunit 9 | 0.334 | 0.100 |
| seq.13932.45 | Rho guanine nucleotide exchange factor 7 | 0.161 | 0.618 |
| seq.13933.276 | NGFI-A-binding protein 1 | 0.000 | 0.343 |
| seq.13934.3 | Guanine nucleotide exchange factor DBS | 0.826 | 0.696 |
| seq.13936.24 | Phosphoglycerate kinase 2 | 0.371 | 0.259 |
| seq.13937.75 | Cullin-associated NEDD8-dissociated protein 1 | 0.570 | 0.000 |
| seq.13939.14 | UTP--glucose-1-phosphate uridylyltransferase | 0.534 | 0.579 |
| seq.13940.19 | Gamma-interferon-inducible protein 16:Isoform 2, Hematopoietic expression, interferon-inducible nature, and nuclear localization 1 | 0.333 | 0.742 |
| seq.13941.82 | Phenylalanine--tRNA ligase, mitochondrial | 0.935 | 0.956 |
| seq.13942.140 | SPRY domain-containing SOCS box protein 1 | 0.000 | 0.339 |
| seq.13943.38 | Protein dpy-30 homolog | 0.948 | 0.349 |
| seq.13944.3 | Sulfotransferase 1A3 | 0.199 | 0.000 |
| seq.13946.8 | Probable ATP-dependent RNA helicase DDX23 | 0.000 | 0.520 |
| seq.13947.371 | Peroxisomal NADH pyrophosphatase NUDT12 | 0.708 | 0.284 |
| seq.13948.50 | Gamma-aminobutyric acid type B receptor subunit 2:Extracellular domain | 0.617 | 0.368 |
| seq.13950.9 | Collagen type IV alpha-3-binding protein:StAR-related lipid-transfer domain, isoform 2 | 0.019 | 0.000 |
| seq.13954.9 | Glucosamine 6-phosphate N-acetyltransferase | 0.063 | 0.000 |
| seq.13955.33 | Death-associated protein kinase 1 | 0.000 | 0.000 |
| seq.13958.5 | Probable RNA-binding protein 46 | 0.969 | 0.233 |
| seq.13959.7 | Cytosol aminopeptidase | 0.221 | 0.000 |
| seq.13960.15 | Arf-GAP with GTPase, ANK repeat and PH domain-containing protein 3 | 0.918 | 0.800 |
| seq.13961.18 | Kinesin-like protein KIF3A | 0.000 | 0.378 |
| seq.13963.7 | Toll-interacting protein | 0.000 | 0.000 |
| seq.13966.30 | Myomesin-3 | 0.643 | 0.470 |
| seq.13967.14 | Thioredoxin reductase 1, cytoplasmic | 0.000 | 0.000 |
| seq.13969.24 | Importin subunit alpha-7 | 0.233 | 0.000 |
| seq.13972.4 | 17-beta-hydroxysteroid dehydrogenase 14 | 0.909 | 0.681 |
| seq.13973.62 | Tubulin--tyrosine ligase | 0.116 | 0.000 |
| seq.13975.56 | Liprin-alpha-1 | 0.142 | 0.000 |
| seq.13976.9 | Rho guanine nucleotide exchange factor 1 | 0.194 | 0.000 |
| seq.13977.28 | BRCA1-associated RING domain protein 1 | 0.828 | 0.530 |
| seq.13978.122 | T-box transcription factor TBX3 | 0.913 | 0.252 |
| seq.13979.3 | Anion exchange transporter | 0.671 | 0.499 |
| seq.13982.33 | Regulator of G-protein signaling 18 | 0.567 | 0.637 |
| seq.13983.27 | Quinone oxidoreductase | 0.769 | 0.839 |
| seq.13984.23 | ATP-dependent RNA helicase DDX25 | 0.329 | 0.025 |
| seq.13985.12 | E3 ubiquitin-protein ligase SMURF2 | 0.510 | 0.689 |
| seq.13986.6 | LanC-like protein 1 | 0.429 | 0.168 |
| seq.13988.67 | NmrA-like family domain-containing protein 1 | 0.942 | 0.229 |
| seq.13990.1 | Pyruvate carboxylase, mitochondrial | 0.456 | 0.125 |
| seq.13991.47 | Eukaryotic translation initiation factor 4 gamma 3 | 0.818 | 0.536 |
| seq.13992.12 | Vesicle-fusing ATPase | 0.330 | 0.391 |
| seq.13993.20 | Band 4.1-like protein 1 | 0.412 | 0.100 |
| seq.13994.1 | rRNA 2'-O-methyltransferase fibrillarin | 0.231 | 0.307 |
| seq.13996.16 | Phosphopantothenoylcysteine decarboxylase | 0.656 | 0.427 |
| seq.13998.26 | Adenylosuccinate synthetase isozyme 1 | 0.723 | 0.332 |
| seq.14002.18 | Dedicator of cytokinesis protein 9 | 0.871 | 0.619 |
| seq.14005.2 | Chromodomain-helicase-DNA-binding protein 7 | 0.666 | 0.000 |
| seq.14006.36 | Glycine N-methyltransferase | 0.513 | 0.469 |
| seq.14007.22 | Bifunctional 3'-phosphoadenosine 5'-phosphosulfate synthase 1 | 0.521 | 0.000 |
| seq.14008.22 | mRNA-decapping enzyme 1A | 0.628 | 0.107 |
| seq.14009.65 | Tumor necrosis factor alpha-induced protein 3 | 0.422 | 0.633 |
| seq.14011.17 | Protein S100-A11 | 0.246 | 0.624 |
| seq.14012.17 | Probable ATP-dependent RNA helicase DHX58 | 0.943 | 0.603 |
| seq.14013.11 | TRAF family member-associated NF-kappa-B activator | 0.280 | 0.000 |
| seq.14019.73 | Envoplakin | 0.986 | 0.851 |
| seq.14021.81 | Mediator of RNA polymerase II transcription subunit 4 | 0.682 | 0.570 |
| seq.14022.17 | Interleukin-17B | 0.862 | 0.422 |
| seq.14023.84 | Bone sialoprotein 2 | 0.005 | 0.630 |
| seq.14024.196 | Ectodysplasin-A, secreted form | 0.499 | 0.407 |
| seq.14025.18 | Tumor necrosis factor receptor superfamily member 9 | 0.596 | 0.170 |
| seq.14026.24 | Interleukin-17F | 0.738 | 0.697 |
| seq.14028.22 | Phosphatidylinositol 4-phosphate 3-kinase C2 domain-containing subunit alpha | 0.613 | 0.497 |
| seq.14029.42 | COP9 signalosome complex subunit 2 | 0.000 | 0.376 |
| seq.14030.21 | Tumor necrosis factor ligand superfamily member 4 | 0.447 | 0.252 |
| seq.14031.18 | Fibroblast growth factor 7 | 0.383 | 0.146 |
| seq.14032.2 | Vascular endothelial growth factor A, isoform 121 | 0.871 | 0.575 |
| seq.14034.22 | Tumor-associated calcium signal transducer 2 | 0.255 | 0.047 |
| seq.14035.13 | T-lymphoma invasion and metastasis-inducing protein 1 | 0.737 | 0.650 |
| seq.14036.116 | Multiple PDZ domain protein | 0.822 | 0.591 |
| seq.14037.18 | Ran-binding protein 3 | 0.392 | 0.144 |
| seq.14038.130 | Cystatin-F | 0.917 | 0.437 |
| seq.14039.33 | Kallikrein-5 | 0.165 | 0.802 |
| seq.14041.13 | Granzyme B | 0.518 | 0.570 |
| seq.14042.11 | Secreted frizzled-related protein 1 | 0.651 | 0.579 |
| seq.14043.12 | Early endosome antigen 1 | 0.556 | 0.551 |
| seq.14045.12 | Nuclear receptor coactivator 2 | 0.979 | 0.908 |
| seq.14047.78 | Brain-derived neurotrophic factor | 0.724 | 0.311 |
| seq.14048.7 | Interleukin-1 Receptor accessory protein | 0.829 | 0.879 |
| seq.14049.17 | Interleukin-7 | 0.746 | 0.334 |
| seq.14050.61 | Ephrin-A4 | 0.637 | 0.663 |
| seq.14051.54 | Forkhead box protein C2 | 0.166 | 0.642 |
| seq.14052.26 | Protein unc-13 homolog A | 0.749 | 0.000 |
| seq.14054.17 | Interleukin-15 receptor subunit alpha | 0.820 | 0.592 |
| seq.14056.4 | Glycoprotein hormones alpha chain | 0.412 | 0.684 |
| seq.14057.68 | Tumor necrosis factor ligand superfamily member 15 | 0.572 | 0.852 |
| seq.14060.67 | Neutrophil cytosol factor 4 | 0.220 | 0.000 |
| seq.14061.48 | Tumor necrosis factor ligand superfamily member 11 | 0.247 | 0.386 |
| seq.14063.17 | Oncostatin-M | 0.380 | 0.815 |
| seq.14064.21 | Peptidoglycan recognition protein 1 | 0.828 | 0.605 |
| seq.14065.11 | T-cell surface glycoprotein CD5:Cytoplasmic domain | 0.829 | 0.472 |
| seq.14066.49 | Membrane-associated guanylate kinase, WW and PDZ domain-containing protein 2 | 0.501 | 0.824 |
| seq.14067.6 | Plakophilin-2 | 0.896 | 0.000 |
| seq.14068.29 | C-C motif chemokine 25 | 0.694 | 0.717 |
| seq.14069.61 | Carbonic anhydrase 4 | 0.346 | 0.401 |
| seq.14070.56 | Intersectin-1 | 0.861 | 0.745 |
| seq.14072.9 | Translationally-controlled tumor protein | 0.380 | 0.204 |
| seq.14073.31 | Hemopexin | 0.410 | 0.210 |
| seq.14074.2 | SHC-transforming protein 2 | 0.856 | 0.653 |
| seq.14076.74 | Cystatin-S | 0.627 | 0.716 |
| seq.14077.6 | Ficolin-3 | 0.571 | 0.666 |
| seq.14078.69 | Lymphotactin | 0.804 | 0.765 |
| seq.14079.14 | Interleukin-18 receptor 1 | 0.657 | 0.543 |
| seq.14081.5 | NKG2D ligand 1 | 0.491 | 0.918 |
| seq.14082.56 | Talin-2 | 0.078 | 0.000 |
| seq.14083.25 | Selenide, water dikinase 1 | 0.164 | 0.033 |
| seq.14085.28 | Interleukin-13 | 0.274 | 0.634 |
| seq.14086.11 | Acid sphingomyelinase-like phosphodiesterase 3a | 0.720 | 0.539 |
| seq.14088.38 | Insulin-like growth factor-binding protein 6 | 0.119 | 0.003 |
| seq.14090.23 | Differentially expressed in FDCP 6 homolog | 0.327 | 0.159 |
| seq.14091.42 | Carbonyl reductase [NADPH] 3 | 0.605 | 0.385 |
| seq.14093.10 | Fms-related tyrosine kinase 3 ligand | 0.830 | 0.544 |
| seq.14094.29 | Heparin-binding EGF-like growth factor | 0.749 | 0.271 |
| seq.14095.1 | NKG2-D type II integral membrane protein | 0.349 | 0.728 |
| seq.14097.86 | Carbohydrate sulfotransferase 15 | 0.689 | 0.284 |
| seq.14098.28 | Cysteine--tRNA ligase, cytoplasmic | 0.261 | 0.000 |
| seq.14099.20 | Proteasome subunit alpha type-4 | 0.964 | 0.727 |
| seq.14100.63 | Complement C1q subcomponent subunit C | 0.786 | 0.852 |
| seq.14101.2 | Ciliary neurotrophic factor receptor subunit alpha | 0.522 | 0.318 |
| seq.14102.6 | Granulysin | 0.890 | 0.835 |
| seq.14103.12 | Tryptase gamma | 0.220 | 0.001 |
| seq.14104.1 | TLR4 interactor with leucine rich repeats:Cytoplasmic domain | 0.427 | 0.957 |
| seq.14105.5 | Kallistatin | 0.471 | 0.659 |
| seq.14106.46 | Rap1 GTPase-GDP dissociation stimulator 1 | 0.410 | 0.000 |
| seq.14107.1 | 5-formyltetrahydrofolate cyclo-ligase | 0.024 | 0.000 |
| seq.14108.15 | Transforming growth factor beta-1 | 0.667 | 0.886 |
| seq.14109.15 | C-C motif chemokine 15 | 0.839 | 0.762 |
| seq.14110.200 | SPARC | 0.493 | 0.336 |
| seq.14111.15 | Thrombospondin-2 | 0.812 | 0.824 |
| seq.14112.40 | Tumor necrosis factor receptor superfamily member 19L | 0.135 | 0.537 |
| seq.14114.18 | PILR alpha-associated neural protein | 0.811 | 0.143 |
| seq.14115.34 | Adrenomedullin | 0.536 | 0.219 |
| seq.14116.129 | Protein S100-A4 | 0.465 | 0.665 |
| seq.14120.2 | E3 ubiquitin-protein ligase RNF43 | 0.809 | 0.480 |
| seq.14121.24 | Tumor necrosis factor receptor superfamily member 10D | 0.833 | 0.317 |
| seq.14122.132 | E3 ubiquitin-protein ligase ZNRF3 | 0.644 | 0.463 |
| seq.14123.34 | V-type immunoglobulin domain-containing suppressor of T-cell activation:Extracellular domain | 0.843 | 0.137 |
| seq.14124.6 | Ephrin-A2 | 0.683 | 0.158 |
| seq.14125.5 | Apolipoprotein M | 0.853 | 0.343 |
| seq.14127.240 | Interferon beta | 0.967 | 0.846 |
| seq.14128.121 | Interferon alpha-10 | 0.848 | 0.701 |
| seq.14129.1 | Interferon alpha-7 | 0.317 | 0.409 |
| seq.14131.37 | Ephrin-B2:Extracellular domain | 0.619 | 0.265 |
| seq.14132.21 | HERV-H LTR-associating protein 2 | 0.039 | 0.870 |
| seq.14133.93 | Interleukin-1 receptor type 2 | 0.672 | 0.796 |
| seq.14134.49 | Amphoterin-induced protein 2 | 0.708 | 0.453 |
| seq.14135.3 | Relaxin receptor 1 | 0.305 | 0.384 |
| seq.14136.234 | Complement component C1q receptor | 0.722 | 0.674 |
| seq.14139.16 | Neuregulin-4 | 0.436 | 0.904 |
| seq.14143.8 | Histone H2B type 2-E | 0.624 | 0.662 |
| seq.14144.3 | Histone H2A type 3 | 0.519 | 0.629 |
| seq.14146.92 | Histone H3.1 | 0.698 | 0.460 |
| seq.14148.2 | Ubiquitin-like protein ISG15 | 0.640 | 0.860 |
| seq.14149.9 | Interleukin-36 beta | 0.686 | 0.127 |
| seq.14150.7 | Interleukin-36 alpha | 0.516 | 0.000 |
| seq.14151.4 | Ubiquitin-like protein ISG15 | 0.581 | 0.646 |
| seq.14153.8 | Ephrin-A3 | 0.718 | 0.585 |
| seq.14156.33 | 14-3-3 protein beta/alpha | 0.000 | 0.000 |
| seq.14157.21 | 14-3-3 protein epsilon | 0.305 | 0.129 |
| seq.14158.17 | Annexin A5 | 0.967 | 0.397 |
| seq.14175.78 | SCP2 sterol-binding domain-containing protein 1 | 0.884 | 0.840 |
| seq.14178.18 | Cyclin-dependent kinase inhibitor 3 | 0.597 | 0.942 |
| seq.14186.13 | E3 ubiquitin-protein ligase rififylin | 0.966 | 0.828 |
| seq.14192.31 | Phosducin-like protein 2 | 0.304 | 0.103 |
| seq.14197.2 | PR domain zinc finger protein 1 | 0.582 | 0.380 |
| seq.14203.3 | Annexin A7 | 0.000 | 0.000 |
| seq.14204.55 | Forkhead box protein J2 | 0.876 | 0.863 |
| seq.14205.6 | Protein HEXIM2 | 0.970 | 0.000 |
| seq.14206.28 | Amyloid beta A4 precursor protein-binding family B member 1:Phosphotyrosine Interaction Domain 2 | 0.948 | 0.239 |
| seq.14208.3 | Retinoid-binding protein 7 | 0.145 | 0.558 |
| seq.14216.35 | Probable ATP-dependent RNA helicase DDX46 | 0.707 | 0.669 |
| seq.14226.120 | S-methylmethionine--homocysteine S-methyltransferase BHMT2 | 0.000 | 0.596 |
| seq.14227.21 | Myosin light chain 6B | 0.908 | 0.580 |
| seq.14229.5 | Adenylyltransferase and sulfurtransferase MOCS3 | 0.582 | 0.392 |
| seq.14237.1 | Heat shock 70 kDa protein 1A | 0.746 | 0.572 |
| seq.14245.195 | Sorting nexin-7 | 0.464 | 0.298 |
| seq.14246.50 | Docking protein 2 | 0.085 | 0.000 |
| seq.14249.68 | Probable E3 ubiquitin-protein ligase MID2 | 0.648 | 0.772 |
| seq.14250.115 | Bleomycin hydrolase | 0.532 | 0.129 |
| seq.14254.27 | Tyrosine-protein phosphatase non-receptor type 4 | 0.621 | 0.331 |
| seq.14260.112 | Neuroepithelial cell-transforming gene 1 protein | 0.696 | 0.900 |
| seq.14268.4 | Sulfiredoxin-1 | 0.000 | 0.523 |
| seq.14271.23 | Ras-related protein Rab-6B | 0.094 | 0.000 |
| seq.14273.19 | Prolyl endopeptidase | 0.170 | 0.000 |
| seq.14283.12 | Ras-related protein Rab-14 | 0.434 | 0.622 |
| seq.14284.23 | eIF-2-alpha kinase GCN2 | 0.018 | 0.063 |
| seq.14286.2 | Nuclear factor of activated T-cells, cytoplasmic 1 | 0.717 | 0.468 |
| seq.14287.6 | Ras-related protein Rab-5C | 0.000 | 0.000 |
| seq.14291.53 | Arf-GAP with GTPase, ANK repeat and PH domain-containing protein 2 | 0.398 | 0.000 |
| seq.14294.61 | Methyl-CpG-binding domain protein 1 | 0.529 | 0.739 |
| seq.14309.8 | Heterogeneous nuclear ribonucleoprotein H | 0.974 | 0.512 |
| seq.14314.6 | Peptidyl-prolyl cis-trans isomerase-like 2 | 0.670 | 0.874 |
| seq.14318.1 | Vacuolar protein sorting-associated protein 29 | 0.960 | 0.861 |
| seq.14324.52 | Structural maintenance of chromosomes protein 3 | 0.161 | 0.453 |
| seq.14326.4 | Ubiquitin-conjugating enzyme E2 E1 | 0.253 | 0.128 |
| seq.14329.4 | Death-inducer obliterator 1 | 0.505 | 0.827 |
| seq.14331.262 | Dynein light chain Tctex-type 1 | 0.786 | 0.917 |
| seq.14332.3 | Ras-related protein Ral-A | 0.003 | 0.000 |
| seq.14334.3 | Recoverin | 0.457 | 0.422 |
| seq.14337.1 | Trafficking protein particle complex subunit 3 | 0.946 | 0.837 |
| seq.14341.8 | Protein FAM69C | 0.183 | 0.125 |
| seq.14583.49 | Growth/differentiation factor 8 | 0.000 | 0.000 |
| seq.14587.16 | Growth/differentiation factor 11 | 0.926 | 0.683 |
| seq.14593.152 | Protein FAM210A | 0.091 | 0.428 |
| seq.14597.5 | Semaphorin-6C, cytoplasmic | 0.727 | 0.145 |
| seq.14599.18 | Stabilin-1 | 0.467 | 0.501 |
| seq.14603.51 | Uncharacterized protein KIAA0040 | 0.142 | 0.049 |
| seq.14614.41 | Noncompact myelin-associated protein | 0.603 | 0.262 |
| seq.14615.46 | Keratin-associated protein 2-4 | 0.768 | 0.000 |
| seq.14616.16 | Zinc finger protein 382 | 0.931 | 0.298 |
| seq.14618.26 | Vesicular, overexpressed in cancer, prosurvival protein 1 | 0.758 | 0.493 |
| seq.14619.8 | Zinc finger protein 526 | 0.788 | 0.326 |
| seq.14623.26 | Small ubiquitin-related modifier 3 | 0.000 | 0.000 |
| seq.14624.51 | Transcriptional repressor CTCF | 0.511 | 0.841 |
| seq.14628.72 | F-box only protein 3 | 0.888 | 0.956 |
| seq.14631.22 | Dual specificity protein phosphatase 16 | 0.850 | 0.683 |
| seq.14633.26 | Transmembrane protein 185A | 0.891 | 0.610 |
| seq.14634.13 | Lutropin-choriogonadotropic hormone receptor | 0.804 | 0.781 |
| seq.14636.25 | Ribonuclease UK114 | 0.487 | 0.439 |
| seq.14645.253 | Glutathione S-transferase A4 | 0.508 | 0.663 |
| seq.14655.1 | DnaJ homolog subfamily C member 17 | 0.690 | 0.093 |
| seq.14662.6 | Myeloid zinc finger 1 | 0.619 | 0.296 |
| seq.14663.44 | E3 ubiquitin-protein ligase RNF8 | 0.000 | 0.000 |
| seq.14670.1 | Ski-like protein | 0.228 | 0.000 |
| seq.14674.63 | Protein kinase C and casein kinase substrate in neurons protein 3 | 0.777 | 0.551 |
| seq.14675.20 | Eukaryotic translation initiation factor 4B | 0.064 | 0.000 |
| seq.14684.17 | Calpain-2 catalytic subunit | 0.230 | 0.585 |
| seq.14685.17 | RAC-beta serine/threonine-protein kinase | 0.384 | 0.568 |
| seq.14687.6 | Perilipin-3 | 0.646 | 0.610 |
| seq.14688.6 | Tyrosine-protein phosphatase non-receptor type 7 | 0.687 | 0.769 |
| seq.14689.3 | Protein SEC13 homolog | 0.498 | 0.287 |
| seq.14692.3 | Zinc finger protein 276 | 0.631 | 0.506 |
| seq.14696.45 | Tryptase beta-2 | 0.898 | 0.889 |
| seq.14703.6 | Prokineticin-1 | 0.639 | 0.493 |
| seq.14705.1 | Vascular endothelial growth factor D | 0.811 | 0.253 |
| seq.14708.59 | Complement component C8 gamma chain | 0.548 | 0.231 |
| seq.14711.27 | Cystatin-M | 0.232 | 0.153 |
| seq.14713.46 | Azurocidin | 0.876 | 0.477 |
| seq.14747.9 | Cytokine receptor-like factor 1 | 0.898 | 0.718 |
| seq.14748.31 | Rho GTPase-activating protein 5 | 0.242 | 0.000 |
| seq.14755.4 | Lactotransferrin | 0.850 | 0.691 |
| seq.14756.29 | Intercellular adhesion molecule 2 | 0.653 | 0.420 |
| seq.14757.144 | Fibroblast growth factor 8 isoform B | 0.056 | 0.000 |
| seq.14759.149 | Cadherin-1 | 0.535 | 0.322 |
| seq.15295.81 | Insulin-like growth factor II:Mature | 0.121 | 0.000 |
| seq.15297.3 | Protein S100-A3 | 0.233 | 0.401 |
| seq.15298.199 | Neuropilin and tolloid-like protein 1 | 0.453 | 0.146 |
| seq.15299.102 | LDLR chaperone MESD | 0.000 | 0.000 |
| seq.15300.66 | Coiled-coil domain-containing protein 134 | 0.481 | 0.000 |
| seq.15301.24 | Acrosomal protein SP-10 | 0.297 | 0.660 |
| seq.15303.63 | Protein S100-A5 | 0.335 | 0.000 |
| seq.15304.1 | Regenerating islet-derived protein 3-alpha | 0.882 | 0.812 |
| seq.15305.7 | Secretagogin | 0.657 | 0.768 |
| seq.15306.20 | Immunoglobulin J chain | 0.956 | 0.566 |
| seq.15308.108 | Brorin | 0.707 | 0.161 |
| seq.15310.61 | Protein regulator of cytokinesis 1 | 0.391 | 0.147 |
| seq.15312.14 | Protein argonaute-1 | 0.328 | 0.039 |
| seq.15313.28 | Mitotic spindle assembly checkpoint protein MAD2A | 0.176 | 0.985 |
| seq.15314.49 | Chloride intracellular channel protein 4 | 0.000 | 0.000 |
| seq.15315.64 | Copper homeostasis protein cutC homolog | 0.822 | 0.740 |
| seq.15316.262 | Thioredoxin-like protein 4B | 0.142 | 0.000 |
| seq.15318.75 | Protein S100-A10 | 0.378 | 0.364 |
| seq.15319.226 | Cyclin-A1 | 0.412 | 0.179 |
| seq.15321.8 | Complexin-2 | 0.513 | 0.229 |
| seq.15322.35 | Death domain-containing protein CRADD | 0.000 | 0.889 |
| seq.15323.112 | Protein argonaute-3 | 0.447 | 0.582 |
| seq.15324.58 | Ferritin light chain | 0.780 | 0.710 |
| seq.15325.14 | Neuromodulin | 0.000 | 0.000 |
| seq.15326.64 | Guanylate-binding protein 1 | 0.882 | 0.756 |
| seq.15329.167 | Ragulator complex protein LAMTOR2 | 0.412 | 0.295 |
| seq.15331.47 | Histone-binding protein RBBP4 | 0.344 | 0.308 |
| seq.15333.11 | Stromal cell-derived factor 2 | 0.874 | 0.882 |
| seq.15336.7 | Selenoprotein M | 0.603 | 0.331 |
| seq.15339.32 | Cofilin-2 | 0.296 | 0.000 |
| seq.15343.337 | Kininogen, HMW, Two Chain | 0.798 | 0.575 |
| seq.15346.31 | Interferon gamma | 0.630 | 0.730 |
| seq.15347.12 | Hemopexin | 0.403 | 0.064 |
| seq.15358.28 | Neuroendocrine protein 7B2 | 0.531 | 0.505 |
| seq.15361.37 | Ankyrin repeat domain-containing protein 1 | 0.653 | 0.333 |
| seq.15363.32 | Apolipoprotein A-V | 0.663 | 0.532 |
| seq.15364.101 | Apolipoprotein C-I | 0.429 | 0.179 |
| seq.15365.41 | Adhesion G protein-coupled receptor B3 | 0.404 | 0.702 |
| seq.15367.38 | BPI fold-containing family B member 1 | 0.838 | 0.780 |
| seq.15368.3 | BMP-binding endothelial regulator protein | 0.721 | 0.593 |
| seq.15370.5 | BolA-like protein 1 | 0.630 | 0.439 |
| seq.15372.43 | Bromodomain-containing protein 2 | 0.672 | 0.729 |
| seq.15374.15 | Gastrokine-1 | 0.827 | 0.910 |
| seq.15375.49 | Carboxypeptidase B | 0.536 | 0.639 |
| seq.15376.134 | Cathepsin E | 0.527 | 0.462 |
| seq.15381.45 | Discoidin domain-containing receptor 2 | 0.529 | 0.268 |
| seq.15383.200 | Endothelin-3 | 0.559 | 0.247 |
| seq.15384.15 | Klotho | 0.641 | 0.440 |
| seq.15385.116 | Fatty acid-binding protein, intestinal | 0.682 | 0.730 |
| seq.15386.7 | Fatty acid-binding protein, adipocyte | 0.701 | 0.331 |
| seq.15387.44 | Neuropilin-2 | 0.234 | 0.236 |
| seq.15388.24 | Low affinity immunoglobulin gamma Fc region receptor III-A | 0.940 | 0.946 |
| seq.15389.1 | Frataxin, mitochondrial | 0.941 | 0.919 |
| seq.15390.3 | Galanin | 0.000 | 0.685 |
| seq.15391.114 | Growth arrest-specific protein 6 | 0.735 | 0.334 |
| seq.15394.79 | Netrin receptor UNC5B | 0.581 | 0.497 |
| seq.15395.15 | Glutathione S-transferase Mu 1 | 0.911 | 0.713 |
| seq.15398.2 | FAD-linked sulfhydryl oxidase ALR | 0.618 | 0.053 |
| seq.15402.2 | DNA-binding protein inhibitor ID-1 | 0.817 | 0.985 |
| seq.15403.53 | DNA-binding protein inhibitor ID-2 | 0.607 | 0.430 |
| seq.15404.3 | Interferon alpha-21 | 0.943 | 0.635 |
| seq.15405.23 | Interferon alpha-4 | 0.844 | 0.000 |
| seq.15412.40 | Baculoviral IAP repeat-containing protein 7 | 0.867 | 0.879 |
| seq.15413.3 | Phosphatidylcholine-sterol acyltransferase | 0.047 | 0.834 |
| seq.15414.316 | L-lactate dehydrogenase A chain | 0.079 | 0.193 |
| seq.15416.54 | Lysozyme g-like protein 2 | 0.672 | 0.310 |
| seq.15417.3 | Serpin B5 | 0.364 | 0.364 |
| seq.15418.25 | Mitogen-activated protein kinase 10 | 0.013 | 0.000 |
| seq.15419.15 | Matrix metalloproteinase-20 | 0.481 | 0.440 |
| seq.15422.12 | Myelin P2 protein | 0.760 | 0.400 |
| seq.15426.5 | Sialidase-1 | 0.706 | 0.094 |
| seq.15427.35 | Lysyl oxidase homolog 3 | 0.766 | 0.137 |
| seq.15430.165 | Opticin | 0.193 | 0.453 |
| seq.15431.31 | Ornithine carbamoyltransferase, mitochondrial | 0.292 | 0.661 |
| seq.15432.1 | Otoraplin | 0.799 | 0.564 |
| seq.15433.4 | Proto-oncogene tyrosine-protein kinase Src | 0.095 | 0.000 |
| seq.15434.5 | Prepronociceptin | 0.096 | 0.432 |
| seq.15435.4 | Purine nucleoside phosphorylase | 0.301 | 0.026 |
| seq.15436.40 | Receptor activity-modifying protein 1 | 0.328 | 0.724 |
| seq.15437.11 | Receptor activity-modifying protein 3 | 0.917 | 0.798 |
| seq.15439.21 | Rho-related GTP-binding protein RhoE | 0.574 | 0.636 |
| seq.15440.57 | Neuroendocrine convertase 2 | 0.810 | 0.258 |
| seq.15441.6 | Ganglioside GM2 activator | 0.811 | 0.272 |
| seq.15444.45 | Serpin B4 | 0.971 | 0.893 |
| seq.15446.25 | Neuregulin-1, sensory and motor neuron-derived factor isoform | 0.550 | 0.867 |
| seq.15447.45 | Sorbitol dehydrogenase | 0.225 | 0.371 |
| seq.15448.47 | Sequestosome-1 | 0.193 | 0.480 |
| seq.15449.33 | T-cell immunoglobulin and mucin domain-containing protein 4 | 0.624 | 0.738 |
| seq.15452.5 | 5'-Nucleotidase | 0.741 | 0.512 |
| seq.15453.3 | Alpha-1-microglobulin | 0.554 | 0.480 |
| seq.15455.40 | Disintegrin and metalloproteinase domain-containing protein 15:Extracellular domain | 0.705 | 0.415 |
| seq.15457.14 | Aminopeptidase N | 0.047 | 0.838 |
| seq.15460.9 | Dipeptidyl peptidase 4 | 0.518 | 0.345 |
| seq.15462.28 | T-cell surface glycoprotein CD8 alpha chain | 0.658 | 0.443 |
| seq.15465.79 | Protein canopy homolog 4 | 0.236 | 0.000 |
| seq.15466.30 | Collagen alpha-1(IX) chain | 0.938 | 0.779 |
| seq.15467.10 | Collagen triple helix repeat-containing protein 1 | 0.676 | 0.518 |
| seq.15468.14 | Complement factor H-related protein 1 | 0.632 | 0.553 |
| seq.15470.11 | Beta-hexosaminidase subunit beta | 0.773 | 0.609 |
| seq.15471.29 | Pancreatic lipase-related protein 2 | 0.808 | 0.860 |
| seq.15472.16 | Low-density lipoprotein receptor-related protein 11 | 0.787 | 0.556 |
| seq.15474.7 | Peptidyl-prolyl cis-trans isomerase NIMA-interacting 1 | 0.086 | 0.000 |
| seq.15475.4 | Phospholipid transfer protein | 0.659 | 0.525 |
| seq.15476.6 | Regenerating islet-derived protein 3-gamma | 0.703 | 0.525 |
| seq.15480.2 | Vascular non-inflammatory molecule 2 | 0.847 | 0.682 |
| seq.15481.45 | Antibacterial protein LL-37 | 0.719 | 0.677 |
| seq.15482.12 | Alpha-2-macroglobulin-like protein 1 | 0.777 | 0.951 |
| seq.15483.377 | Agrin | 0.644 | 0.476 |
| seq.15486.126 | Amiloride-sensitive amine oxidase [copper-containing] | 0.897 | 0.799 |
| seq.15487.164 | Liver carboxylesterase 1 | 0.714 | 0.352 |
| seq.15491.20 | Endosialin | 0.563 | 0.459 |
| seq.15492.1 | Cysteine-rich motor neuron 1 protein:Extracellular domain | 0.175 | 0.000 |
| seq.15494.11 | Fibroblast growth factor-binding protein 1 | 0.605 | 0.561 |
| seq.15495.9 | Folate receptor gamma | 0.984 | 0.923 |
| seq.15497.9 | Heparan sulfate glucosamine 3-O-sulfotransferase 1 | 0.346 | 0.000 |
| seq.15499.11 | Attractin | 0.350 | 0.174 |
| seq.15503.15 | Left-right determination factor 2 | 0.691 | 0.513 |
| seq.15503.20 | Left-right determination factor 2 | 0.703 | 0.736 |
| seq.15504.39 | Baculoviral IAP repeat-containing protein 7 Isoform beta | 0.321 | 0.147 |
| seq.15506.34 | Low-density lipoprotein receptor-related protein 12 | 0.631 | 0.337 |
| seq.15509.2 | Alpha-N-acetylglucosaminidase | 0.793 | 0.684 |
| seq.15511.37 | Neuronal pentraxin receptor | 0.495 | 0.454 |
| seq.15513.108 | Prostasin | 0.788 | 0.738 |
| seq.15514.26 | Cholinesterase | 0.716 | 0.638 |
| seq.15515.2 | Serum amyloid A-1 protein | 0.686 | 0.337 |
| seq.15516.12 | Serum amyloid A-4 protein | 0.597 | 0.768 |
| seq.15521.4 | Calsyntenin-1 | 0.824 | 0.424 |
| seq.15522.2 | Golgi-associated plant pathogenesis-related protein 1 | 0.406 | 0.513 |
| seq.15523.9 | Delta-aminolevulinic acid dehydratase | 0.557 | 0.087 |
| seq.15524.30 | Phosphoglycerate mutase 2 | 0.154 | 0.000 |
| seq.15525.294 | Alcohol dehydrogenase 1C | 0.149 | 0.367 |
| seq.15526.33 | Glutathione synthetase | 0.688 | 0.731 |
| seq.15527.90 | Dihydrolipoyl dehydrogenase, mitochondrial | 0.803 | 0.713 |
| seq.15529.33 | Cysteine and glycine-rich protein 1 | 0.038 | 0.000 |
| seq.15530.33 | Ephrin type-B receptor 4 | 0.576 | 0.301 |
| seq.15533.97 | Macrophage scavenger receptor types I and II:Extracellular domain | 0.764 | 0.503 |
| seq.15534.26 | Malate dehydrogenase, mitochondrial | 0.223 | 0.000 |
| seq.15535.3 | Serine protease 27 | 0.808 | 0.477 |
| seq.15539.15 | SLIT and NTRK-like protein 1 | 0.474 | 0.303 |
| seq.15540.6 | Vimentin | 0.657 | 0.306 |
| seq.15542.19 | Creatine kinase U-type, mitochondrial | 0.193 | 0.334 |
| seq.15544.25 | Kallikrein-14 | 0.463 | 0.626 |
| seq.15545.13 | Calcineurin subunit B type 1 | 0.676 | 0.289 |
| seq.15548.35 | D-3-phosphoglycerate dehydrogenase | 0.647 | 0.538 |
| seq.15553.22 | Acetylcholinesterase | 0.968 | 0.330 |
| seq.15556.49 | Alpha-amylase 2B | 0.745 | 0.633 |
| seq.15558.63 | Glutamyl aminopeptidase | 0.263 | 0.000 |
| seq.15559.5 | Anthrax toxin receptor 2 | 0.844 | 0.479 |
| seq.15560.52 | Transcobalamin-2 | 0.912 | 0.934 |
| seq.15562.24 | Beta-glucuronidase | 0.735 | 0.704 |
| seq.15565.102 | Mucin-16 | 0.813 | 0.625 |
| seq.15566.10 | Calponin-1 | 0.473 | 0.039 |
| seq.15567.2 | T-cell surface glycoprotein CD3 zeta chain | 0.681 | 0.036 |
| seq.15569.15 | Collagen Type II | 0.317 | 0.357 |
| seq.15570.99 | Complement receptor type 2 | 0.804 | 0.804 |
| seq.15573.110 | Neurocan core protein | 0.624 | 0.300 |
| seq.15574.37 | Cyclin-A2 | 0.458 | 0.000 |
| seq.15576.158 | Eosinophil cationic protein | 0.713 | 0.629 |
| seq.15579.26 | Ectonucleotide pyrophosphatase/phosphodiesterase family member 6 | 0.912 | 0.807 |
| seq.15580.2 | Ephrin type-A receptor 7 | 0.824 | 0.634 |
| seq.15581.16 | Fibronectin type 3 and ankyrin repeat domains protein 1 | 0.825 | 0.494 |
| seq.15582.25 | Ficolin-1 | 0.751 | 0.440 |
| seq.15583.18 | Fc receptor-like B | 0.955 | 0.682 |
| seq.15584.9 | Complement factor H-related protein 2 | 0.970 | 0.989 |
| seq.15585.304 | Fibulin-5 | 0.372 | 0.331 |
| seq.15587.20 | Folate receptor beta | 0.764 | 0.527 |
| seq.15588.17 | Alpha-galactosidase A | 0.949 | 0.665 |
| seq.15589.1 | Vitamin D-binding protein | 0.348 | 0.434 |
| seq.15591.28 | Glutathione peroxidase 1 | 0.523 | 0.043 |
| seq.15594.47 | Serine protease HTRA1 | 0.623 | 0.265 |
| seq.15596.7 | Protein HEXIM1 | 0.048 | 0.000 |
| seq.15602.43 | Interleukin-6 receptor subunit alpha | 0.914 | 0.619 |
| seq.15603.20 | Integrin alpha-2 | 0.817 | 0.846 |
| seq.15604.18 | Mitogen-activated protein kinase 9 | 0.668 | 0.000 |
| seq.15606.19 | Keratin, type I cytoskeletal 19 | 0.480 | 0.467 |
| seq.15607.56 | Pyruvate kinase PKLR | 0.474 | 0.283 |
| seq.15608.5 | Ribosomal protein S6 kinase beta-1 | 0.292 | 0.000 |
| seq.15610.72 | Cytosol aminopeptidase | 0.293 | 0.574 |
| seq.15612.5 | Protein lin-7 homolog B | 0.324 | 0.640 |
| seq.15613.16 | Pancreatic triacylglycerol lipase | 0.402 | 0.659 |
| seq.15614.168 | Leukocyte immunoglobulin-like receptor subfamily A member 2 | 0.360 | 0.817 |
| seq.15615.8 | Leukocyte immunoglobulin-like receptor subfamily B member 3 | 0.989 | 0.905 |
| seq.15617.8 | Cytokine SCM-1 beta | 0.627 | 0.958 |
| seq.15619.49 | Myelin-oligodendrocyte glycoprotein | 0.921 | 0.224 |
| seq.15620.4 | Neuroligin-1 | 0.482 | 0.000 |
| seq.15622.13 | Opioid-binding protein/cell adhesion molecule | 0.411 | 0.083 |
| seq.15623.1 | Programmed cell death protein 1 | 0.644 | 0.918 |
| seq.15626.223 | Basement membrane-specific heparan sulfate proteoglycan core protein | 0.000 | 0.285 |
| seq.15627.83 | RAC-alpha serine/threonine-protein kinase | 0.010 | 0.000 |
| seq.15631.18 | Pregnancy-specific beta-1-glycoprotein 1 | 0.189 | 0.699 |
| seq.15633.6 | Retinol-binding protein 4 | 0.678 | 0.798 |
| seq.15634.139 | Slit homolog 1 protein | 0.614 | 0.658 |
| seq.15635.4 | SPARC-related modular calcium-binding protein 2 | 0.802 | 0.634 |
| seq.15636.49 | VPS10 domain-containing receptor SorCS1 | 0.442 | 0.000 |
| seq.15637.38 | VPS10 domain-containing receptor SorCS3 | 0.829 | 0.532 |
| seq.15640.54 | Transgelin | 0.523 | 0.405 |
| seq.15641.20 | Tomoregulin-1 | 0.490 | 0.680 |
| seq.15644.1 | Biotinidase | 0.660 | 0.664 |
| seq.15653.9 | Collagen alpha-1(X) chain | 0.924 | 0.865 |
| seq.15666.21 | Bone morphogenetic protein 2 | 0.576 | 0.000 |
| seq.15667.39 | Bone morphogenetic protein 4 | 0.691 | 0.244 |
| seq.15668.19 | Bone morphogenetic protein 8B | 0.453 | 0.529 |
| seq.15669.7 | Serine/threonine-protein kinase B-raf | 0.667 | 0.798 |
| seq.15670.15 | Complement C1q-like protein 2 | 0.546 | 0.623 |
| seq.15674.3 | B-cell antigen receptor complex-associated protein alpha chain | 0.704 | 0.818 |
| seq.15675.3 | CCAAT/enhancer-binding protein beta | 0.837 | 0.338 |
| seq.15678.71 | Dickkopf-related protein 2 | 0.750 | 0.574 |
| seq.15686.49 | Inhibin beta C chain | 0.964 | 0.859 |
| seq.15688.30 | Protein ERGIC-53 | 0.574 | 0.921 |
| seq.15692.300 | Nodal homolog | 0.694 | 0.000 |
| seq.15693.9 | Progesterone receptor | 0.139 | 0.227 |
| seq.15698.6 | Alpha-taxilin | 0.341 | 0.977 |
| seq.16015.19 | Alanine aminotransferase 1 | 0.343 | 0.766 |
| seq.16021.30 | Cadherin-8 | 0.487 | 0.285 |
| seq.16035.8 | Vascular endothelial growth factor receptor 3 | 0.458 | 0.381 |
| seq.16043.30 | SHC-transforming protein 1:Phosphotyrosine Interaction Domain | 0.000 | 0.000 |
| seq.16049.43 | Oxidized low-density lipoprotein receptor 1 | 0.064 | 0.000 |
| seq.16055.3 | Complement factor H-related protein 5 | 0.744 | 0.514 |
| seq.16057.6 | Cation-independent mannose-6-phosphate receptor | 0.692 | 0.549 |
| seq.16060.99 | Nidogen-2 | 0.433 | 0.442 |
| seq.16070.7 | Wnt inhibitory factor 1 | 0.732 | 0.548 |
| seq.16074.12 | GRB2-related adapter protein 2 | 0.531 | 0.000 |
| seq.16079.2 | Tyrosine-protein kinase Tec | 0.000 | 0.000 |
| seq.16081.38 | Aldo-keto reductase family 1 member B10 | 0.563 | 0.452 |
| seq.16288.17 | Ephrin type-A receptor 4 | 0.700 | 0.000 |
| seq.16292.288 | Gastric inhibitory polypeptide | 0.303 | 0.286 |
| seq.16293.1 | Somatoliberin | 0.947 | 0.541 |
| seq.16296.43 | Leucine-rich repeat-containing G-protein coupled receptor 5 | 0.727 | 0.494 |
| seq.16297.14 | Roundabout homolog 4 | 0.758 | 0.818 |
| seq.16298.84 | Group IID secretory phospholipase A2 | 0.859 | 0.827 |
| seq.16299.13 | T-cell immunoreceptor with Ig and ITIM domains | 0.616 | 0.124 |
| seq.16300.4 | Triggering receptor expressed on myeloid cells 2 | 0.623 | 0.314 |
| seq.16302.11 | BPI fold-containing family A member 2 | 0.357 | 0.556 |
| seq.16304.6 | Leucine-rich repeat-containing G-protein coupled receptor 4 | 0.962 | 0.692 |
| seq.16305.10 | Cadherin-11:Extracellular domain | 0.890 | 0.813 |
| seq.16307.22 | Netrin receptor UNC5D | 0.554 | 0.282 |
| seq.16308.14 | B- and T-lymphocyte attenuator | 0.435 | 0.320 |
| seq.16309.30 | Dickkopf-like protein 1 | 0.460 | 0.780 |
| seq.16312.45 | Cadherin-6 | 0.155 | 0.318 |
| seq.16315.105 | Vascular endothelial growth factor receptor 1 | 0.520 | 0.577 |
| seq.16317.20 | Desmoglein-3 | 0.778 | 0.509 |
| seq.16318.12 | Serine/threonine-protein kinase receptor R3 | 0.505 | 0.417 |
| seq.16320.139 | CD320 antigen | 0.947 | 0.657 |
| seq.16322.10 | Marginal zone B- and B1-cell-specific protein | 0.829 | 0.669 |
| seq.16323.8 | Neurexin-3 | 0.664 | 0.185 |
| seq.16324.38 | Toll-like receptor 1:Extracellular domain | 0.307 | 0.614 |
| seq.16536.3 | High mobility group protein HMG-I/HMG-Y | 0.596 | 0.377 |
| seq.16551.14 | Signal transducer and activator of transcription 5B | 0.875 | 0.410 |
| seq.16558.2 | Myocilin | 0.836 | 0.608 |
| seq.16561.9 | Alpha-1B-glycoprotein | 0.337 | 0.430 |
| seq.16583.8 | Bis(5'-nucleosyl)-tetraphosphatase [asymmetrical] | 0.252 | 0.015 |
| seq.16585.16 | N-acetyllactosaminide beta-1,3-N-acetylglucosaminyltransferase 4 | 0.331 | 0.288 |
| seq.16587.1 | Beta-casein | 0.125 | 0.000 |
| seq.16588.10 | 78 kDa glucose-regulated protein | 0.053 | 0.000 |
| seq.16591.71 | Acidic mammalian chitinase | 0.179 | 0.717 |
| seq.16593.3 | FAS-associated death domain protein | 0.691 | 0.872 |
| seq.16594.44 | Fas apoptotic inhibitory molecule 1 | 0.629 | 0.749 |
| seq.16596.25 | Glutaredoxin-3 | 0.247 | 0.079 |
| seq.16597.11 | Glutaredoxin-related protein 5, mitochondrial | 0.925 | 0.699 |
| seq.16599.38 | GPN-loop GTPase 1 | 0.455 | 0.054 |
| seq.16605.2 | Complement C1q and tumor necrosis factor-related protein 9A | 0.986 | 0.694 |
| seq.16606.85 | Aldose reductase | 0.000 | 0.035 |
| seq.16607.78 | Gelsolin | 0.467 | 0.168 |
| seq.16609.106 | Kin of IRRE-like protein 2 | 0.954 | 0.704 |
| seq.16610.13 | Low-density lipoprotein receptor-related protein 10 | 0.541 | 0.134 |
| seq.16612.28 | Syndecan-3 | 0.695 | 0.455 |
| seq.16613.3 | Cadherin-17 | 0.870 | 0.674 |
| seq.16614.27 | R-spondin-1 | 0.600 | 0.392 |
| seq.16616.137 | Beta-enolase | 0.777 | 0.524 |
| seq.16617.14 | Caspase-14 | 0.226 | 0.199 |
| seq.16618.7 | Early activation antigen CD69 | 0.630 | 0.639 |
| seq.16620.26 | Lymphocyte antigen 75 | 0.827 | 0.816 |
| seq.16621.77 | NAD(P)H-hydrate epimerase | 0.288 | 0.000 |
| seq.16746.12 | Activin B | 0.647 | 0.592 |
| seq.16748.1 | Bone morphogenetic protein 3 | 0.613 | 0.348 |
| seq.16749.79 | Growth/differentiation factor 10 | 0.464 | 0.394 |
| seq.16751.15 | Natriuretic peptides B | 0.822 | 0.629 |
| seq.16753.46 | Collagen alpha-2(VI) chain | 0.623 | 0.768 |
| seq.16754.40 | Peripheral plasma membrane protein CASK | 0.762 | 0.193 |
| seq.16755.195 | Growth/differentiation factor 3 | 0.757 | 0.062 |
| seq.16756.30 | Growth/differentiation factor 7 | 0.318 | 0.690 |
| seq.16758.96 | Hepatoma-derived growth factor | 0.453 | 0.161 |
| seq.16760.2 | Interleukin-26 | 0.229 | 0.831 |
| seq.16763.11 | Leukocyte cell-derived chemotaxin-2 | 0.811 | 0.856 |
| seq.16765.52 | Platelet basic protein | 0.723 | 0.339 |
| seq.16768.3 | Junction plakoglobin | 0.970 | 0.790 |
| seq.16769.20 | Ras-related C3 botulinum toxin substrate 3 | 0.365 | 0.000 |
| seq.16770.3 | Lithostathine-1-beta | 0.689 | 0.605 |
| seq.16773.29 | Signal peptide, CUB and EGF-like domain-containing protein 3 | 0.881 | 0.466 |
| seq.16780.6 | Heat shock 70 kDa protein 1A | 0.365 | 0.474 |
| seq.16781.2 | Cytosolic endo-beta-N-acetylglucosaminidase | 0.946 | 0.756 |
| seq.16785.45 | Defensin-5 | 0.596 | 0.596 |
| seq.16792.4 | Sialic acid-binding Ig-like lectin 5 | 0.912 | 0.943 |
| seq.16802.31 | OCIA domain-containing protein 1 | 0.750 | 0.790 |
| seq.16803.4 | Calretinin | 0.958 | 0.884 |
| seq.16805.5 | cGMP-specific 3',5'-cyclic phosphodiesterase | 0.000 | 0.000 |
| seq.16807.35 | Troponin T, fast skeletal muscle | 0.579 | 0.503 |
| seq.16809.1 | Nucleoside diphosphate kinase, mitochondrial | 0.252 | 0.000 |
| seq.16810.3 | Rab GDP dissociation inhibitor alpha | 0.399 | 0.042 |
| seq.16814.13 | Four and a half LIM domains protein 1 | 0.482 | 0.862 |
| seq.16818.200 | CUB domain-containing protein 1 | 0.782 | 0.623 |
| seq.16823.75 | Apolipoprotein L3 | 0.586 | 0.829 |
| seq.16825.20 | Ataxin-3 | 0.365 | 0.080 |
| seq.16828.8 | Collagen alpha-1(VI) chain | 0.890 | 0.905 |
| seq.16831.7 | Docking protein 1 | 0.000 | 0.000 |
| seq.16836.1 | Complement factor H-related protein 3 | 0.675 | 0.110 |
| seq.16837.20 | CASP8 and FADD-like apoptosis regulator | 0.000 | 0.000 |
| seq.16845.15 | Myelin protein zero-like protein 1 | 0.693 | 0.000 |
| seq.16847.39 | Ras-related protein Rab-3B | 0.829 | 0.682 |
| seq.16850.5 | Regulator of G-protein signaling 5 | 0.347 | 0.201 |
| seq.16851.50 | Protein SCO2 homolog, mitochondrial | 0.847 | 0.000 |
| seq.16852.10 | Na(+)/H(+) exchange regulatory cofactor NHE-RF3 | 0.041 | 0.689 |
| seq.16853.5 | Nicotinamide riboside kinase 1 | 0.764 | 0.877 |
| seq.16854.17 | U6 snRNA-associated Sm-like protein LSm4 | 0.394 | 0.441 |
| seq.16856.79 | Microtubule-associated protein RP/EB family member 2 | 0.430 | 0.000 |
| seq.16857.2 | Ras-related protein Rab-2A | 0.755 | 0.000 |
| seq.16858.384 | Reticulocalbin-3 | 0.582 | 0.396 |
| seq.16859.100 | Endophilin-B1 | 0.832 | 0.869 |
| seq.16863.47 | N-acylneuraminate-9-phosphatase | 0.638 | 0.733 |
| seq.16865.62 | TATA-binding protein-associated factor 2N | 0.288 | 0.000 |
| seq.16867.76 | Cyclin-dependent kinase 16 | 0.360 | 0.105 |
| seq.16872.248 | Maleylacetoacetate isomerase | 0.942 | 0.692 |
| seq.16875.13 | Protein mago nashi homolog 2 | 0.332 | 0.608 |
| seq.16877.19 | Methionine-R-sulfoxide reductase B2, mitochondrial | 0.756 | 0.034 |
| seq.16882.27 | 14 kDa phosphohistidine phosphatase | 0.272 | 0.186 |
| seq.16883.57 | Ras-related protein Rab-4A | 0.105 | 0.417 |
| seq.16885.49 | Microtubule-associated protein RP/EB family member 3 | 0.087 | 0.054 |
| seq.16887.29 | Peptidyl-tRNA hydrolase 2, mitochondrial | 0.673 | 0.487 |
| seq.16890.37 | ADAMTS-like protein 1 | 0.520 | 0.324 |
| seq.16892.23 | Ectonucleotide pyrophosphatase/phosphodiesterase family member 2 | 0.371 | 0.773 |
| seq.16899.59 | Oxysterol-binding protein-related protein 9 | 0.726 | 0.589 |
| seq.16900.29 | MAM domain-containing glycosylphosphatidylinositol anchor protein 1 | 0.917 | 0.793 |
| seq.16902.17 | Lysosome-associated membrane glycoprotein 1 | 0.936 | 0.867 |
| seq.16907.3 | Cell adhesion molecule 2 | 0.181 | 0.039 |
| seq.16908.5 | Oligodendrocyte-myelin glycoprotein | 0.199 | 0.240 |
| seq.16913.8 | Ribonuclease T2 | 0.867 | 0.282 |
| seq.16914.104 | Monocyte differentiation antigen CD14, soluble | 0.511 | 0.225 |
| seq.16915.153 | Semaphorin-4A | 0.780 | 0.176 |
| seq.16916.19 | SLIT and NTRK-like protein 6 | 0.614 | 0.726 |
| seq.16918.198 | Toll-like receptor 3 | 0.659 | 0.617 |
| seq.16919.1 | Acyl-CoA-binding protein | 0.380 | 0.015 |
| seq.16922.53 | Plexin-B3 | 0.946 | 0.693 |
| seq.16923.20 | Profilin-2 | 0.438 | 0.134 |
| seq.16926.44 | Alkaline phosphatase, tissue-nonspecific isozyme | 0.755 | 0.511 |
| seq.16927.9 | Coagulation factor XIII | 0.187 | 0.642 |
| seq.16932.5 | Estrogen sulfotransferase | 0.772 | 0.880 |
| seq.17137.160 | Hemoglobin subunit beta | 0.708 | 0.792 |
| seq.17138.8 | Glutathione S-transferase A1 | 0.350 | 0.317 |
| seq.17140.57 | Platelet-derived growth factor D | 0.765 | 0.559 |
| seq.17145.1 | Protein S100-A8/A9 heterodimer | 0.000 | 0.115 |
| seq.17148.7 | Flavin reductase (NADPH) | 0.455 | 0.020 |
| seq.17150.8 | 10 kDa heat shock protein, mitochondrial | 0.270 | 0.000 |
| seq.17151.84 | Interferon regulatory factor 3 | 0.000 | 0.000 |
| seq.17152.10 | Killer cell immunoglobulin-like receptor 2DS4 | 0.988 | 0.363 |
| seq.17153.46 | Killer cell immunoglobulin-like receptor 2DL3 | 0.768 | 0.717 |
| seq.17154.2 | Prostaglandin E synthase 3 | 0.722 | 0.501 |
| seq.17155.1 | Vacuolar protein sorting-associated protein 28 homolog | 0.622 | 0.513 |
| seq.17156.72 | Serine/threonine-protein kinase DCLK1 | 0.733 | 0.477 |
| seq.17158.17 | Ubiquitin-conjugating enzyme E2 G1 | 0.577 | 0.000 |
| seq.17161.1 | Dolichyl-diphosphooligosaccharide--protein glycosyltransferase 48 kDa subunit | 0.114 | 0.092 |
| seq.17163.117 | Annexin A3 | 0.536 | 0.341 |
| seq.17164.15 | Annexin A4 | 0.367 | 0.000 |
| seq.17165.1 | Beta-thromboglobulin | 0.701 | 0.133 |
| seq.17166.4 | Fibroblast growth factor 8 isoform F | 0.908 | 0.340 |
| seq.17170.15 | Calcitonin gene-related peptide 2 | 0.917 | 0.165 |
| seq.17172.19 | Statherin | 0.939 | 0.892 |
| seq.17175.5 | Dual specificity mitogen-activated protein kinase kinase 6 | 0.914 | 0.778 |
| seq.17176.13 | PC4 and SFRS1-interacting protein | 0.447 | 0.197 |
| seq.17195.43 | BAG family molecular chaperone regulator 1 | 0.294 | 0.310 |
| seq.17196.5 | ATP-dependent Clp protease proteolytic subunit, mitochondrial | 0.252 | 0.344 |
| seq.17199.43 | Hypoxia-inducible factor 1-alpha inhibitor | 0.533 | 0.178 |
| seq.17200.50 | Killer cell immunoglobulin-like receptor 2DL1 | 0.657 | 0.409 |
| seq.17202.37 | Programmed cell death protein 4 | 0.773 | 0.067 |
| seq.17204.17 | Phenylethanolamine N-methyltransferase | 0.473 | 0.064 |
| seq.17205.21 | Ras-related protein Rab-5A | 0.000 | 0.482 |
| seq.17209.27 | Sulfotransferase family cytosolic 2B member 1 | 0.574 | 0.508 |
| seq.17210.2 | T-cell leukemia/lymphoma protein 1A | 0.787 | 0.645 |
| seq.17224.12 | Mimecan | 0.522 | 0.319 |
| seq.17231.1 | Plastin-2 | 0.586 | 0.505 |
| seq.17319.1 | Selenoprotein H | 0.352 | 0.208 |
| seq.17320.19 | L-aminoadipate-semialdehyde dehydrogenase-phosphopantetheinyl transferase | 0.005 | 0.000 |
| seq.17325.10 | Guanylate kinase | 0.139 | 0.022 |
| seq.17326.44 | Dual specificity protein phosphatase 23 | 0.960 | 0.660 |
| seq.17327.3 | Protein canopy homolog 3 | 0.792 | 0.637 |
| seq.17329.2 | 3-hydroxybutyrate dehydrogenase type 2 | 0.586 | 0.387 |
| seq.17331.138 | Kremen protein 1 | 0.429 | 0.233 |
| seq.17332.3 | Poly(ADP-ribose) glycohydrolase ARH3 | 0.163 | 0.154 |
| seq.17333.20 | Medium-chain specific acyl-CoA dehydrogenase, mitochondrial | 0.203 | 0.000 |
| seq.17335.18 | Vesicle-associated membrane protein 2 | 0.555 | 0.869 |
| seq.17336.54 | Cyclic AMP-responsive element-binding protein 3-like protein 2 | 0.791 | 0.737 |
| seq.17337.1 | Transcriptional repressor protein YY1 | 0.679 | 0.320 |
| seq.17341.89 | Acetyl-CoA acetyltransferase, cytosolic | 0.434 | 0.164 |
| seq.17342.13 | Anterior gradient protein 3 | 0.661 | 0.492 |
| seq.17343.6 | Syntaphilin | 0.871 | 0.595 |
| seq.17344.23 | Stomatin-like protein 1 | 0.347 | 0.283 |
| seq.17345.12 | Prostaglandin reductase 3 | 0.288 | 0.000 |
| seq.17346.61 | Follicular dendritic cell secreted peptide | 0.712 | 0.798 |
| seq.17347.80 | Axin interactor, dorsalization-associated protein | 0.176 | 0.116 |
| seq.17348.5 | Synaptojanin-2-binding protein | 0.404 | 0.899 |
| seq.17350.13 | Charged multivesicular body protein 2b | 0.020 | 0.000 |
| seq.17355.56 | Synaptotagmin-4 | 0.379 | 0.000 |
| seq.17356.34 | Interleukin-1 family member 10 | 0.716 | 0.260 |
| seq.17357.33 | O-phosphoseryl-tRNA(Sec) selenium transferase | 0.535 | 0.922 |
| seq.17362.5 | Von Hippel-Lindau disease tumor suppressor | 0.839 | 0.811 |
| seq.17364.8 | U2 small nuclear ribonucleoprotein B'' | 0.078 | 0.409 |
| seq.17365.7 | Kynurenine--oxoglutarate transaminase 1 | 0.453 | 0.707 |
| seq.17366.6 | DCN1-like protein 1 | 0.643 | 0.408 |
| seq.17367.5 | Stathmin | 0.311 | 0.788 |
| seq.17370.186 | Mycophenolic acid acyl-glucuronide esterase, mitochondrial | 0.294 | 0.000 |
| seq.17372.5 | Eukaryotic translation initiation factor 4E-binding protein 1 | 0.524 | 0.855 |
| seq.17377.1 | Aldo-keto reductase family 1 member C3 | 0.355 | 0.868 |
| seq.17380.2 | Ubiquitin-conjugating enzyme E2 K | 0.000 | 0.008 |
| seq.17383.4 | StAR-related lipid transfer protein 5 | 0.611 | 0.962 |
| seq.17384.110 | ATP-dependent 6-phosphofructokinase, muscle type | 0.125 | 0.047 |
| seq.17387.27 | NF-kappa-B inhibitor beta | 0.789 | 0.816 |
| seq.17391.10 | Splicing factor 45 | 0.298 | 0.000 |
| seq.17393.13 | Short-chain specific acyl-CoA dehydrogenase, mitochondrial | 0.314 | 0.370 |
| seq.17396.23 | Alcohol dehydrogenase 1A | 0.175 | 0.321 |
| seq.17397.8 | Estradiol 17-beta-dehydrogenase 11 | 0.850 | 0.000 |
| seq.17398.55 | Heme oxygenase 1 | 0.802 | 0.617 |
| seq.17400.71 | Acyl-coenzyme A thioesterase 8 | 0.635 | 0.732 |
| seq.17403.14 | Short/branched chain specific acyl-CoA dehydrogenase, mitochondrial | 0.671 | 0.000 |
| seq.17404.5 | ADP-ribosylation factor-like protein 5B | 0.636 | 0.153 |
| seq.17405.2 | Ubiquitin carboxyl-terminal hydrolase isozyme L5 | 0.550 | 0.081 |
| seq.17408.2 | Phosphomannomutase 1 | 0.516 | 0.933 |
| seq.17410.5 | Centrin-3 | 0.800 | 0.401 |
| seq.17411.55 | Ubiquitin recognition factor in ER-associated degradation protein 1 | 0.000 | 0.000 |
| seq.17419.17 | Testin | 0.926 | 0.790 |
| seq.17427.26 | Neuroligin-3 | 0.598 | 0.498 |
| seq.17432.25 | Adenylate kinase 4, mitochondrial | 0.528 | 0.374 |
| seq.17435.43 | Electron transfer flavoprotein subunit alpha, mitochondrial | 0.586 | 0.676 |
| seq.17436.193 | Casein kinase I isoform gamma-2 | 0.087 | 0.000 |
| seq.17441.4 | Intestinal-type alkaline phosphatase | 0.653 | 0.840 |
| seq.17447.52 | Secreted frizzled-related protein 4 | 0.653 | 0.606 |
| seq.17449.23 | CD9 antigen | 0.494 | 0.537 |
| seq.17450.51 | Histidine--tRNA ligase, cytoplasmic | 0.000 | 0.000 |
| seq.17451.13 | Bcl-2-like protein 11 | 0.755 | 0.094 |
| seq.17453.34 | Ceruloplasmin | 0.567 | 0.484 |
| seq.17454.15 | Epidermal growth factor-like protein 6 | 0.751 | 0.594 |
| seq.17455.42 | Folate receptor alpha | 0.537 | 0.307 |
| seq.17456.53 | Golgi membrane protein 1 | 0.679 | 0.452 |
| seq.17460.51 | Interferon-induced GTP-binding protein Mx1 | 0.677 | 0.559 |
| seq.17462.19 | Interferon regulatory factor 1 | 0.668 | 0.265 |
| seq.17466.72 | Acyl-CoA synthetase family member 2, mitochondrial | 0.685 | 0.000 |
| seq.17467.1 | Dehydrogenase/reductase SDR family member 9 | 0.476 | 0.670 |
| seq.17468.1 | Suppressor of fused homolog | 0.496 | 0.045 |
| seq.17474.106 | YEATS domain-containing protein 4 | 0.288 | 0.552 |
| seq.17475.18 | Lymphokine-activated killer T-cell-originated protein kinase | 0.658 | 0.778 |
| seq.17490.4 | SH3 domain-binding glutamic acid-rich-like protein 3 | 0.000 | 0.000 |
| seq.17495.141 | NAD-dependent protein deacetylase sirtuin-3, mitochondrial | 0.502 | 0.551 |
| seq.17505.125 | N-acetylated-alpha-linked acidic dipeptidase-like protein | 0.002 | 0.428 |
| seq.17509.6 | 4-hydroxyphenylpyruvate dioxygenase | 0.330 | 0.834 |
| seq.17510.7 | Signal recognition particle 14 kDa protein | 0.539 | 0.690 |
| seq.17511.10 | Ribonuclease P protein subunit p30 | 0.523 | 0.764 |
| seq.17512.2 | N-myc-interactor | 0.388 | 0.000 |
| seq.17513.11 | Annexin A11 | 0.215 | 0.000 |
| seq.17514.48 | Ras-related protein Rab-21 | 0.000 | 0.000 |
| seq.17515.6 | Heat shock 70 kDa protein 13 | 0.800 | 0.438 |
| seq.17516.7 | Ras-related protein Rab-3A | 0.342 | 0.417 |
| seq.17671.58 | Inhibitor of growth protein 4 | 0.874 | 0.634 |
| seq.17672.184 | Gastric intrinsic factor | 0.725 | 0.617 |
| seq.17673.34 | Carbonic anhydrase 5B, mitochondrial | 0.682 | 0.665 |
| seq.17675.17 | Acyl-coenzyme A thioesterase 13 | 0.128 | 0.000 |
| seq.17676.13 | Cdc42-interacting protein 4 | 0.016 | 0.000 |
| seq.17677.47 | CD160 antigen | 0.121 | 0.171 |
| seq.17678.28 | V-set and immunoglobulin domain-containing protein 4 | 0.786 | 0.520 |
| seq.17680.12 | Ephrin type-B receptor 1 | 0.809 | 0.511 |
| seq.17682.1 | Membrane cofactor protein | 0.728 | 0.563 |
| seq.17683.2 | COMM domain-containing protein 9 | 0.362 | 0.000 |
| seq.17685.9 | Apolipoprotein A-IV | 0.413 | 0.538 |
| seq.17686.27 | Tubulin-folding cofactor B | 0.000 | 0.000 |
| seq.17691.1 | Tripeptidyl-peptidase 1 | 0.595 | 0.623 |
| seq.17692.2 | Butyrophilin subfamily 3 member A3 | 0.838 | 0.380 |
| seq.17693.2 | Septin-6 | 0.800 | 0.266 |
| seq.17694.32 | Proteasome activator complex subunit 2 | 0.456 | 0.073 |
| seq.17696.1 | TIR domain-containing adapter molecule 2 | 0.636 | 0.089 |
| seq.17697.2 | Esterase OVCA2 | 0.000 | 0.000 |
| seq.17698.15 | WW domain-binding protein 2 | 0.791 | 0.782 |
| seq.17699.43 | Protein timeless homolog | 0.807 | 0.869 |
| seq.17702.53 | UDP-glucuronosyltransferase 1-1 | 0.522 | 0.852 |
| seq.17703.40 | Geminin | 0.718 | 0.935 |
| seq.17704.74 | Heat shock protein 105 kDa | 0.754 | 0.449 |
| seq.17706.4 | Protein phosphatase 1 regulatory subunit 1A | 0.695 | 0.825 |
| seq.17710.40 | Tumor protein D53 | 0.150 | 0.156 |
| seq.17711.13 | Vesicle-associated membrane protein 1 | 0.919 | 0.982 |
| seq.17712.7 | Isopentenyl-diphosphate Delta-isomerase 1 | 0.363 | 0.105 |
| seq.17721.82 | Growth arrest-specific protein 7 | 0.608 | 0.427 |
| seq.17722.5 | Peptidyl-prolyl cis-trans isomerase FKBP4 | 0.502 | 0.236 |
| seq.17724.3 | WW domain-containing oxidoreductase | 0.894 | 0.717 |
| seq.17725.37 | Harmonin | 0.688 | 0.545 |
| seq.17726.3 | GTP-binding protein SAR1a | 0.050 | 0.000 |
| seq.17727.1 | Securin | 0.812 | 0.935 |
| seq.17728.61 | Charged multivesicular body protein 2a | 0.011 | 0.000 |
| seq.17729.20 | Ubiquitin-conjugating enzyme E2 S | 0.346 | 0.000 |
| seq.17734.13 | Alpha-endosulfine | 0.092 | 0.160 |
| seq.17735.130 | Gamma-aminobutyric acid receptor-associated protein | 0.308 | 0.437 |
| seq.17736.105 | Aprataxin | 0.953 | 0.446 |
| seq.17737.7 | Isovaleryl-CoA dehydrogenase, mitochondrial | 0.091 | 0.000 |
| seq.17738.7 | Cornulin | 0.447 | 0.805 |
| seq.17739.1 | Hydroxyacyl-coenzyme A dehydrogenase, mitochondrial | 0.182 | 0.000 |
| seq.17742.2 | Ras-related protein R-Ras | 0.471 | 0.393 |
| seq.17743.14 | Ubiquitin-conjugating enzyme E2 A | 0.000 | 0.257 |
| seq.17744.31 | Ras-related protein Rab-11A | 0.015 | 0.000 |
| seq.17746.77 | Mitochondrial fission 1 protein | 0.247 | 0.000 |
| seq.17747.45 | TNF receptor-associated factor 1 | 0.620 | 0.430 |
| seq.17748.21 | Quinone oxidoreductase PIG3 | 0.385 | 0.354 |
| seq.17750.8 | Ribonucleoside-diphosphate reductase subunit M2 | 0.393 | 0.809 |
| seq.17751.68 | Beta-crystallin B1 | 0.923 | 0.178 |
| seq.17752.24 | Arginase-2, mitochondrial | 0.816 | 0.224 |
| seq.17755.5 | UDP-glucose 6-dehydrogenase | 0.296 | 0.000 |
| seq.17756.69 | Deoxycytidylate deaminase | 0.140 | 0.000 |
| seq.17757.86 | Calcium-binding protein 39 | 0.050 | 0.000 |
| seq.17758.79 | L-xylulose reductase | 0.406 | 0.140 |
| seq.17760.128 | WD repeat-containing protein 5 | 0.554 | 0.162 |
| seq.17761.2 | ADP-sugar pyrophosphatase | 0.607 | 0.373 |
| seq.17764.108 | Rho-related GTP-binding protein RhoC | 0.000 | 0.000 |
| seq.17765.3 | Snurportin-1 | 0.000 | 0.113 |
| seq.17766.5 | Neutrophil cytosol factor 1 | 0.000 | 0.621 |
| seq.17768.50 | Hydroxyacid oxidase 1 | 0.371 | 0.625 |
| seq.17769.28 | PEST proteolytic signal-containing nuclear protein | 0.224 | 0.000 |
| seq.17770.42 | ER membrane protein complex subunit 8 | 0.799 | 0.744 |
| seq.17771.35 | DNA-directed RNA polymerase III subunit RPC9 | 0.396 | 0.508 |
| seq.17772.7 | Ubiquitin D | 0.410 | 0.420 |
| seq.17773.26 | Gamma-soluble NSF attachment protein | 0.172 | 0.000 |
| seq.17774.38 | Small nuclear ribonucleoprotein Sm D3 | 0.237 | 0.593 |
| seq.17775.8 | Transgelin-3 | 0.316 | 0.060 |
| seq.17776.15 | Peroxisomal trans-2-enoyl-CoA reductase | 0.682 | 0.282 |
| seq.17777.31 | Serine dehydratase-like | 0.789 | 0.389 |
| seq.17778.19 | Ras-related protein Rab-32 | 0.060 | 0.000 |
| seq.17781.191 | Microtubule-associated proteins 1A/1B light chain 3A | 0.000 | 0.142 |
| seq.17782.23 | 3-ketoacyl-CoA thiolase, peroxisomal | 0.430 | 0.530 |
| seq.17783.9 | Cob(I)yrinic acid a,c-diamide adenosyltransferase, mitochondrial | 0.398 | 0.000 |
| seq.17784.23 | Glia maturation factor beta | 0.196 | 0.208 |
| seq.17785.11 | 2'-deoxynucleoside 5'-phosphate N-hydrolase 1 | 0.394 | 0.197 |
| seq.17786.5 | Geranylgeranyl pyrophosphate synthase | 0.469 | 0.393 |
| seq.17787.1 | Enoyl-CoA hydratase, mitochondrial | 0.337 | 0.024 |
| seq.17789.1 | Nicotinate-nucleotide pyrophosphorylase [carboxylating] | 0.602 | 0.806 |
| seq.17791.25 | Hippocalcin-like protein 1 | 0.509 | 0.775 |
| seq.17792.158 | Succinate-semialdehyde dehydrogenase, mitochondrial | 0.936 | 0.858 |
| seq.17793.4 | GTP-binding protein Di-Ras1 | 0.938 | 0.201 |
| seq.17794.6 | Phosphomannomutase 2 | 0.000 | 0.000 |
| seq.17795.176 | U6 snRNA-associated Sm-like protein LSm1 | 0.717 | 0.648 |
| seq.17796.15 | Inositol monophosphatase 1 | 0.767 | 0.531 |
| seq.17797.1 | Cullin-1 | 0.454 | 0.486 |
| seq.17799.9 | 6-phosphogluconolactonase | 0.059 | 0.000 |
| seq.17800.33 | SCAN domain-containing protein 1 | 0.977 | 0.157 |
| seq.17802.4 | Sialic acid synthase | 0.218 | 0.111 |
| seq.17804.102 | Pyridoxine-5'-phosphate oxidase | 0.225 | 0.464 |
| seq.17805.35 | Survival of motor neuron-related-splicing factor 30 | 0.147 | 0.316 |
| seq.17806.6 | Syntenin-1 | 0.305 | 0.428 |
| seq.17808.37 | Omega-amidase NIT2 | 0.070 | 0.000 |
| seq.17811.78 | Sulfotransferase 1C2 | 0.229 | 0.668 |
| seq.17812.2 | Protein tyrosine phosphatase type IVA 1 | 0.749 | 0.708 |
| seq.17813.21 | Valacyclovir hydrolase | 0.663 | 0.723 |
| seq.17814.8 | 3'(2'),5'-bisphosphate nucleotidase 1 | 0.030 | 0.000 |
| seq.17816.58 | Neurocalcin-delta | 0.379 | 0.662 |
| seq.17817.22 | DNA damage-inducible transcript 4 protein | 0.184 | 0.930 |
| seq.17818.22 | Protein SGT1 homolog | 0.000 | 0.000 |
| seq.17819.30 | Acylpyruvase FAHD1, mitochondrial | 0.445 | 0.018 |
| seq.17820.170 | Myeloid leukemia factor 1 | Did not converge | 0.533 |
| seq.17821.20 | Glycylpeptide N-tetradecanoyltransferase 2 | 0.236 | 0.402 |
| seq.17822.57 | Ras-related and estrogen-regulated growth inhibitor | 0.000 | 0.503 |
| seq.17823.40 | Small nuclear ribonucleoprotein Sm D2 | 0.774 | 0.190 |
| seq.17826.341 | Elongation factor 1-delta | 0.000 | 0.000 |
| seq.17827.53 | Dynein light chain roadblock-type 2 | 0.530 | 0.699 |
| seq.17828.3 | Protein S100-A14 | 0.775 | 0.384 |
| seq.17829.2 | Three prime repair exonuclease 2 | 0.951 | 0.578 |
| seq.17832.12 | Isopentenyl-diphosphate delta-isomerase 2 | 0.941 | 0.827 |
| seq.17835.28 | Annexin A13 | 0.940 | 0.725 |
| seq.17836.17 | Protein S100-A16 | 0.534 | 0.498 |
| seq.17837.5 | Chloride intracellular channel protein 2 | 0.399 | 0.081 |
| seq.17843.30 | Phosphopantothenate--cysteine ligase | 0.029 | 0.000 |
| seq.17849.6 | Developmentally-regulated GTP-binding protein 1 | 0.017 | 0.000 |
| seq.17850.42 | Krueppel-like factor 4 | 0.977 | 0.672 |
| seq.17852.5 | Protein phosphatase methylesterase 1 | 0.289 | 0.139 |
| seq.17854.33 | N-alpha-acetyltransferase 10 | 0.179 | 0.000 |
| seq.17855.28 | Regulator of G-protein signaling 4 | 0.286 | 0.124 |
| seq.17856.23 | 5'(3')-deoxyribonucleotidase, mitochondrial | 0.567 | 0.000 |
| seq.17857.6 | Long-chain specific acyl-CoA dehydrogenase, mitochondrial | 0.867 | 0.785 |
| seq.18156.7 | Ubiquitin-like-conjugating enzyme ATG3 | 0.567 | 0.183 |
| seq.18158.45 | Caspase-8 | 0.876 | 0.737 |
| seq.18160.2 | Protein-L-isoaspartate(D-aspartate) O-methyltransferase | 0.917 | 0.474 |
| seq.18162.167 | Interleukin-1 receptor-associated kinase 4 | 0.424 | 0.000 |
| seq.18165.181 | Ubiquitin-conjugating enzyme E2 variant 2 | 0.229 | 0.110 |
| seq.18166.4 | Rho-related GTP-binding protein RhoB | 0.342 | 0.320 |
| seq.18170.46 | Pyrroline-5-carboxylate reductase 1, mitochondrial | 0.784 | 0.472 |
| seq.18171.25 | C-X-C motif chemokine 11 | 0.568 | 0.690 |
| seq.18172.71 | Histone chaperone ASF1A | 0.989 | 0.784 |
| seq.18173.11 | Aflatoxin B1 aldehyde reductase member 3 | 0.167 | 0.433 |
| seq.18174.79 | Programmed cell death 6-interacting protein | 0.142 | 0.000 |
| seq.18175.65 | Synaptic vesicle membrane protein VAT-1 homolog | 0.809 | 0.810 |
| seq.18177.49 | NEDD8-activating enzyme E1 regulatory subunit | 0.707 | 0.702 |
| seq.18178.13 | Serine--tRNA ligase, cytoplasmic | 0.540 | 0.687 |
| seq.18179.56 | Spermine synthase | 0.269 | 0.425 |
| seq.18180.58 | Reticulon-4-interacting protein 1, mitochondrial | 0.741 | 0.186 |
| seq.18181.2 | Protein TSSC4 | 0.416 | 0.000 |
| seq.18182.24 | Phosphoenolpyruvate carboxykinase, cytosolic [GTP] | 0.698 | 0.392 |
| seq.18183.3 | Low density lipoprotein receptor adapter protein 1 | 0.055 | 0.000 |
| seq.18184.28 | GMP synthase [glutamine-hydrolyzing] | 0.000 | 0.008 |
| seq.18185.118 | Fructose-bisphosphate aldolase B | 0.435 | 0.806 |
| seq.18186.15 | Threonine--tRNA ligase, cytoplasmic | 0.000 | 0.000 |
| seq.18187.16 | Aspartoacylase | 0.304 | 0.663 |
| seq.18188.12 | Glycine amidinotransferase, mitochondrial | 0.256 | 0.000 |
| seq.18189.12 | Vacuolar protein-sorting-associated protein 25 | 0.285 | 0.013 |
| seq.18190.15 | Caspase recruitment domain-containing protein 18 | 0.819 | 0.630 |
| seq.18191.23 | Ran-specific GTPase-activating protein | 0.293 | 0.167 |
| seq.18192.69 | Melanoma-associated antigen 5 | 0.642 | 0.432 |
| seq.18193.165 | Mortality factor 4-like protein 1 | 0.073 | 0.487 |
| seq.18194.18 | Fatty acid-binding protein 9 | 0.645 | 0.469 |
| seq.18195.3 | Oral cancer-overexpressed protein 1 | 0.557 | 0.454 |
| seq.18196.8 | Afamin | 0.725 | 0.767 |
| seq.18197.97 | Creatine kinase S-type, mitochondrial | 0.593 | 0.757 |
| seq.18198.51 | Hemoglobin subunit theta-1 | 0.619 | 0.257 |
| seq.18202.22 | Leukocyte antigen CD37 | 0.357 | 0.288 |
| seq.18203.9 | Gamma-crystallin C | 0.533 | 0.644 |
| seq.18204.1 | Transcription elongation factor A protein 1 | 0.359 | 0.202 |
| seq.18205.123 | Melanoma-associated antigen 6 | 0.652 | 0.799 |
| seq.18206.18 | Alcohol dehydrogenase 6 | 0.089 | 0.000 |
| seq.18207.6 | Charged multivesicular body protein 1b | 0.155 | 0.000 |
| seq.18208.3 | G2/mitotic-specific cyclin-B2 | 0.628 | 0.537 |
| seq.18210.12 | Spindlin-1 | 0.523 | 0.671 |
| seq.18212.43 | Glutathione S-transferase Mu 5 | 0.542 | 0.858 |
| seq.18213.30 | NEDD8-conjugating enzyme UBE2F | 0.192 | 0.385 |
| seq.18214.2 | Glutamate--cysteine ligase regulatory subunit | 0.333 | 0.092 |
| seq.18215.5 | Probable tRNA(His) guanylyltransferase | 0.350 | 0.000 |
| seq.18216.22 | Interleukin-11 receptor subunit alpha | 0.157 | 0.000 |
| seq.18218.48 | CB1 cannabinoid receptor-interacting protein 1 | 0.477 | 0.116 |
| seq.18220.141 | Steroid receptor RNA activator 1 | 0.185 | 0.088 |
| seq.18222.34 | Endophilin-B2 | 0.372 | 0.315 |
| seq.18224.11 | Charged multivesicular body protein 1a | 0.000 | 0.000 |
| seq.18225.13 | Heme-binding protein 1 | 0.434 | 0.203 |
| seq.18226.148 | Cytochrome c oxidase subunit 5A, mitochondrial | 0.274 | 0.000 |
| seq.18227.3 | Cyclin-dependent kinase 2-associated protein 2 | 0.555 | 0.274 |
| seq.18228.30 | Nucleolar protein 16 | 0.441 | 0.196 |
| seq.18231.147 | Prion-like protein doppel | 0.619 | 0.356 |
| seq.18232.42 | Phosphatidylinositol 5-phosphate 4-kinase type-2 beta | 0.042 | 0.000 |
| seq.18233.10 | Aspartate aminotransferase, mitochondrial | 0.315 | 0.000 |
| seq.18235.16 | Glycerol-3-phosphate phosphatase | 0.607 | 0.424 |
| seq.18236.3 | Galectin-related protein | 0.071 | 0.000 |
| seq.18237.29 | Nuclear receptor-interacting protein 3 | 0.621 | 0.236 |
| seq.18240.6 | Olfactory marker protein | 0.367 | 0.537 |
| seq.18241.18 | Oxygen-dependent coproporphyrinogen-III oxidase, mitochondrial | 0.838 | 0.566 |
| seq.18242.8 | Sperm surface protein Sp17 | 0.214 | 0.418 |
| seq.18243.9 | UMP-CMP kinase | 0.367 | 0.000 |
| seq.18244.1 | Annexin A7 | 0.004 | 0.002 |
| seq.18253.8 | Single-stranded DNA-binding protein, mitochondrial | 0.525 | 0.753 |
| seq.18255.6 | NADH-cytochrome b5 reductase 1 | 0.340 | 0.338 |
| seq.18257.64 | Signal recognition particle 19 kDa protein | 0.939 | 0.681 |
| seq.18259.15 | Homeobox protein MOX-2 | 0.537 | 0.430 |
| seq.18261.34 | Protein NATD1 | 0.289 | 0.303 |
| seq.18264.12 | Coiled-coil domain-containing protein 25 | 0.975 | 0.703 |
| seq.18265.18 | Zinc finger protein 34 | 0.969 | 0.837 |
| seq.18267.74 | Caspase recruitment domain-containing protein 17 | 0.950 | 0.377 |
| seq.18268.5 | G antigen 2A | 0.931 | 0.846 |
| seq.18270.10 | Transcription initiation factor TFIID subunit 10 | 0.600 | 0.497 |
| seq.18271.43 | 3-oxoacyl-[acyl-carrier-protein] synthase, mitochondrial | 0.629 | 0.000 |
| seq.18273.14 | G antigen 2D | 0.951 | 0.984 |
| seq.18274.2 | Small EDRK-rich factor 2 | 0.208 | 0.082 |
| seq.18275.5 | Cysteine-rich protein 1 | 0.488 | 0.519 |
| seq.18276.34 | T-cell leukemia/lymphoma protein 1B | 0.361 | 0.501 |
| seq.18277.28 | TP53-regulated inhibitor of apoptosis 1 | 0.955 | 0.890 |
| seq.18280.29 | Putative D-tyrosyl-tRNA(Tyr) deacylase 2 | 0.223 | 0.046 |
| seq.18282.1 | Guanine nucleotide-binding protein G(I)/G(S)/G(O) subunit gamma-11 | 0.430 | 0.492 |
| seq.18284.77 | Protein KIBRA | 0.985 | 0.972 |
| seq.18285.6 | Small VCP/p97-interacting protein | 0.604 | 0.676 |
| seq.18286.3 | ZW10 interactor | 0.255 | 0.053 |
| seq.18289.16 | C-C motif chemokine 15 | 0.836 | 0.700 |
| seq.18290.6 | Annexin A8 | 0.412 | 0.147 |
| seq.18291.8 | Cyclin-dependent kinase inhibitor 1 | 0.564 | 0.318 |
| seq.18294.26 | Transcription factor SOX-2 | 0.575 | 0.831 |
| seq.18295.102 | Glyoxylate reductase/hydroxypyruvate reductase | 0.509 | 0.171 |
| seq.18297.8 | Ras-related protein M-Ras | 0.730 | 0.371 |
| seq.18299.13 | Calpain small subunit 1 | 0.168 | 0.000 |
| seq.18300.39 | Transcriptional activator protein Pur-beta | 0.167 | 0.013 |
| seq.18301.10 | Nuclear apoptosis-inducing factor 1 | 0.166 | 0.167 |
| seq.18302.204 | Protein zwilch homolog | 0.357 | 0.484 |
| seq.18303.39 | Neurogranin | 0.000 | 0.000 |
| seq.18304.19 | BRCA2 and CDKN1A-interacting protein | 0.198 | 0.139 |
| seq.18306.1 | Paralemmin-1 | 0.340 | 0.314 |
| seq.18307.71 | Inorganic pyrophosphatase 2, mitochondrial | 0.078 | 0.000 |
| seq.18308.30 | Syntaxin-binding protein 6 | 0.687 | 0.937 |
| seq.18309.18 | Citrate synthase, mitochondrial | 0.103 | 0.000 |
| seq.18310.26 | Selenoprotein W | 0.254 | 0.000 |
| seq.18311.44 | Dynactin subunit 6 | 0.000 | 0.000 |
| seq.18312.68 | Protein NDRG3 | 0.896 | 0.933 |
| seq.18313.4 | Isoaspartyl peptidase/L-asparaginase | 0.726 | 0.508 |
| seq.18314.88 | Paired box protein Pax-8 | 0.238 | 0.199 |
| seq.18315.38 | Receptor-transporting protein 4 | 0.614 | 0.000 |
| seq.18316.75 | Neuronal-specific septin-3 | 0.833 | 0.000 |
| seq.18317.111 | AN1-type zinc finger protein 5 | 0.632 | 0.792 |
| seq.18318.98 | Endophilin-A3 | 0.000 | 0.000 |
| seq.18319.7 | Pyruvate dehydrogenase protein X component, mitochondrial | 0.236 | 0.000 |
| seq.18321.38 | Bifunctional methylenetetrahydrofolate dehydrogenase/cyclohydrolase, mitochondrial | 0.234 | 0.178 |
| seq.18322.15 | Bifunctional arginine demethylase and lysyl-hydroxylase JMJD6 | 0.695 | 0.600 |
| seq.18323.39 | Dihydropyrimidinase-related protein 1 | 0.973 | 0.930 |
| seq.18324.61 | Sulfite oxidase, mitochondrial | 0.955 | 0.705 |
| seq.18326.50 | Melanoma-associated antigen 8 | 0.686 | 0.335 |
| seq.18327.6 | Sorting nexin-5 | 0.426 | 0.000 |
| seq.18328.36 | Pyrroline-5-carboxylate reductase 2 | 0.325 | 0.515 |
| seq.18329.4 | Ribose-phosphate pyrophosphokinase 2 | 0.215 | 0.152 |
| seq.18330.7 | Phosphoglucomutase-2 | 0.782 | 0.869 |
| seq.18331.3 | Glucosylceramidase | 0.598 | 0.139 |
| seq.18332.17 | Complexin-1 | 0.681 | 0.455 |
| seq.18336.31 | Cytidine deaminase | 0.753 | 0.618 |
| seq.18337.4 | GDP-mannose 4,6 dehydratase | 0.785 | 0.911 |
| seq.18338.26 | Isocitrate dehydrogenase [NADP] cytoplasmic | 0.373 | 0.000 |
| seq.18339.207 | Proteasome subunit beta type-3 | 0.692 | 0.504 |
| seq.18340.2 | Proteasome subunit beta type-4 | 0.159 | 0.455 |
| seq.18342.2 | Phosphoserine aminotransferase | 0.537 | 0.466 |
| seq.18343.10 | Peroxisomal 2,4-dienoyl-CoA reductase | 0.406 | 0.000 |
| seq.18347.15 | Laminin-2 | 0.692 | 0.401 |
| seq.18348.89 | Heterogeneous nuclear ribonucleoprotein D0 | 0.071 | 0.341 |
| seq.18373.13 | Radixin | 0.038 | 0.091 |
| seq.18375.28 | Interleukin-36 receptor antagonist protein | 0.633 | 0.495 |
| seq.18376.19 | Myosin light chain 3 | 0.861 | 0.581 |
| seq.18380.78 | Serum albumin | 0.680 | 0.528 |
| seq.18381.16 | Aldehyde dehydrogenase, mitochondrial | 0.625 | 0.150 |
| seq.18382.109 | Catechol O-methyltransferase | 0.463 | 0.485 |
| seq.18383.9 | Peptidyl-prolyl cis-trans isomerase FKBP3 | 0.000 | 0.000 |
| seq.18385.4 | 26S proteasome non-ATPase regulatory subunit 10 | 0.670 | 0.000 |
| seq.18386.36 | Glutaredoxin-1 | 0.692 | 0.539 |
| seq.18387.7 | Hsc70-interacting protein | 0.192 | 0.168 |
| seq.18389.11 | Interferon alpha-1/13 | 0.563 | 0.691 |
| seq.18392.19 | Methionine adenosyltransferase 2 subunit beta | 0.570 | 0.828 |
| seq.18395.5 | [Protein ADP-ribosylarginine] hydrolase | 0.175 | 0.352 |
| seq.18396.10 | Amino-terminal enhancer of split | 0.880 | 0.495 |
| seq.18397.5 | Aldo-keto reductase family 1 member C4 | 0.244 | 0.295 |
| seq.18398.1 | 3-oxo-5-beta-steroid 4-dehydrogenase | 0.162 | 0.246 |
| seq.18399.1 | A-kinase anchor protein 7 isoforms alpha and beta | 0.295 | 0.437 |
| seq.18400.52 | DNA oxidative demethylase ALKBH2 | 0.966 | 0.681 |
| seq.18401.18 | Alpha-ketoglutarate-dependent dioxygenase alkB homolog 3 | 0.894 | 0.366 |
| seq.18402.1 | AMMECR1-like protein | 0.274 | 0.703 |
| seq.18403.25 | AMP deaminase 2 | 0.096 | 0.000 |
| seq.18404.22 | Ankyrin repeat domain-containing protein 54 | 0.671 | 0.097 |
| seq.18405.117 | AP-1 complex subunit sigma-2 | 0.563 | 0.144 |
| seq.18407.36 | ADP-ribosylation factor-like protein 2-binding protein | 0.423 | 0.571 |
| seq.18408.26 | ADP-ribosylation factor 4 | 0.000 | 0.058 |
| seq.18409.61 | ADP-ribosylation factor 5 | 0.411 | 0.970 |
| seq.18410.26 | ADP-ribosylation factor-like protein 14 | 0.109 | 0.000 |
| seq.18411.83 | ADP-ribosylation factor-like protein 15 | 0.408 | 0.477 |
| seq.18413.24 | ADP-ribosylation factor-like protein 4D | 0.599 | 0.475 |
| seq.18414.26 | ADP-ribosylation factor-like protein 5A | 0.179 | 0.678 |
| seq.18415.16 | ADP-ribosylation factor-like protein 6 | 0.003 | 0.000 |
| seq.18416.3 | ADP-ribosylation factor-like protein 9 | 0.840 | 0.756 |
| seq.18417.3 | Arsenite methyltransferase | 0.256 | 0.081 |
| seq.18419.20 | Actin-related protein 2/3 complex subunit 5 | 0.774 | 0.769 |
| seq.18422.41 | Diphosphomevalonate decarboxylase | 0.891 | 0.955 |
| seq.18429.10 | Cadherin-1 | 0.822 | 0.577 |
| seq.18432.32 | Ras-related protein Ral-B | 0.000 | 0.000 |
| seq.18434.141 | TATA box-binding protein-like protein 1 | 0.557 | 0.692 |
| seq.18435.40 | UBX domain-containing protein 2B | 0.069 | 0.000 |
| seq.18449.33 | Peptidyl-prolyl cis-trans isomerase G | 0.467 | 0.528 |
| seq.18458.4 | Prostaglandin E synthase 2 | 0.287 | 0.283 |
| seq.18483.36 | Guanine nucleotide exchange factor MSS4 | 0.658 | 0.611 |
| seq.18813.15 | ATP-dependent RNA helicase DDX19A | 0.053 | 0.000 |
| seq.18814.21 | Inhibin beta A chain:Inhibin beta C chain heterodimer | 0.832 | 0.312 |
| seq.18817.50 | Chromobox protein homolog 1 | 0.894 | 0.511 |
| seq.18819.21 | Peptidyl-prolyl cis-trans isomerase C | 0.672 | 0.507 |
| seq.18821.9 | C4a anaphylatoxin | 0.495 | 0.423 |
| seq.18823.52 | Histone H2B type 3-B | 0.571 | 0.631 |
| seq.18824.7 | Eukaryotic initiation factor 4A-II | 0.354 | 0.184 |
| seq.18829.4 | Eukaryotic initiation factor 4A-I | 0.237 | 0.151 |
| seq.18830.1 | Intelectin-1 | 0.634 | 0.324 |
| seq.18831.6 | Leucine-rich repeats and immunoglobulin-like domains protein 1 | 0.807 | 0.435 |
| seq.18832.65 | Serum amyloid A-2 protein | 0.510 | 0.149 |
| seq.18833.76 | Astrocytic phosphoprotein PEA-15 | 0.461 | 0.379 |
| seq.18837.9 | Small glutamine-rich tetratricopeptide repeat-containing protein beta | 0.615 | 0.792 |
| seq.18839.24 | Thyroglobulin | 0.820 | 0.460 |
| seq.18840.205 | SH2 domain-containing protein 1B | 0.436 | 0.729 |
| seq.18841.1 | Serpin B13 | 0.543 | 0.307 |
| seq.18842.24 | Ubiquitin-conjugating enzyme E2 D2 | 0.000 | 0.933 |
| seq.18859.7 | Eukaryotic translation initiation factor 4E-binding protein 3 | 0.984 | 0.801 |
| seq.18860.2 | Calcium/calmodulin-dependent protein kinase kinase 1 | 0.424 | 0.000 |
| seq.18863.176 | Carbohydrate sulfotransferase 2 | 0.949 | 0.247 |
| seq.18864.7 | Trypsin-3 | 0.616 | 0.784 |
| seq.18866.8 | Calcineurin subunit B type 2 | 0.807 | 0.416 |
| seq.18868.7 | Chromobox protein homolog 3 | 0.864 | 0.565 |
| seq.18870.1 | T-cell surface glycoprotein CD3 epsilon chain | 0.768 | 0.534 |
| seq.18871.24 | Allograft inflammatory factor 1-like | 0.443 | 0.672 |
| seq.18873.8 | Carcinoembryonic antigen-related cell adhesion molecule 8 | 0.863 | 0.606 |
| seq.18874.66 | CCAAT/enhancer-binding protein alpha | 0.667 | 0.823 |
| seq.18875.125 | Chondrocalcin | 0.842 | 0.626 |
| seq.18876.77 | Carbohydrate sulfotransferase 4 | 0.489 | 0.483 |
| seq.18877.15 | Calponin-2 | 0.331 | 0.226 |
| seq.18878.15 | Gremlin-1 | 0.829 | 0.696 |
| seq.18880.81 | Collagen Type III | 0.697 | 0.186 |
| seq.18881.7 | CD97 antigen | 0.604 | 0.411 |
| seq.18882.7 | Calsyntenin-2 | 0.846 | 0.335 |
| seq.18883.4 | Destrin | 0.026 | 0.039 |
| seq.18884.22 | DnaJ homolog subfamily B member 4 | 0.476 | 0.199 |
| seq.18886.28 | Down syndrome cell adhesion molecule-like protein 1 | 0.956 | 0.232 |
| seq.18887.7 | Ectonucleoside triphosphate diphosphohydrolase 2 | 0.586 | 0.615 |
| seq.18888.37 | Fatty acid-binding protein 12 | 0.870 | 0.609 |
| seq.18890.227 | Fibrinogen beta chain | 0.004 | 0.139 |
| seq.18891.98 | Guanylate-binding protein 2 | 0.912 | 0.296 |
| seq.18892.48 | Glypican-4 | 0.827 | 0.562 |
| seq.18893.26 | Adhesion G-protein coupled receptor G1 | 0.635 | 0.698 |
| seq.18894.1 | Glutathione peroxidase 2 | 0.676 | 0.187 |
| seq.18895.54 | Glutathione S-transferase Mu 4 | 0.694 | 0.215 |
| seq.18896.23 | Heparan-sulfate 6-O-sulfotransferase 3 | 0.672 | 0.338 |
| seq.18897.31 | Histone deacetylase 2 | 0.451 | 0.140 |
| seq.18898.36 | Hepatoma-derived growth factor-like protein 1 | 0.694 | 0.317 |
| seq.18899.82 | Hepatoma-derived growth factor-related protein 3 | 0.667 | 0.581 |
| seq.18900.37 | GTPase HRas | 0.023 | 0.193 |
| seq.18901.26 | Heat shock 70 kDa protein 1B | 0.240 | 0.543 |
| seq.18904.23 | Calcium/calmodulin-dependent protein kinase type IV | 0.832 | 0.045 |
| seq.18905.5 | Keratin, type I cytoskeletal 16 | 0.756 | 0.068 |
| seq.18907.97 | Killer cell immunoglobulin-like receptor 3DL1 | 0.453 | 0.528 |
| seq.18909.11 | Exosome complex component RRP43 | 0.633 | 0.470 |
| seq.18910.45 | Protein lin-7 homolog A | 0.749 | 0.925 |
| seq.18913.3 | Cyclin-H | 0.657 | 0.318 |
| seq.18914.188 | Platelet-activating factor acetylhydrolase 2, cytoplasmic | 0.826 | 0.715 |
| seq.18916.25 | Inosine triphosphate pyrophosphatase | 0.781 | 0.673 |
| seq.18917.53 | Pancreatic alpha-amylase | 0.719 | 0.749 |
| seq.18918.86 | cAMP-specific 3',5'-cyclic phosphodiesterase 4A | 0.744 | 0.502 |
| seq.18921.30 | Phosphate-regulating neutral endopeptidase | 0.466 | 0.295 |
| seq.18922.27 | Macrosialin | 0.785 | 0.330 |
| seq.18925.24 | Proteasome subunit alpha type-5 | 0.494 | 0.304 |
| seq.18926.7 | Proteasome subunit alpha type-3 | 0.509 | 0.536 |
| seq.18927.14 | Retinoic acid early transcript 1G protein | 0.605 | 0.490 |
| seq.18928.10 | Protein S100-Z | 0.981 | 0.756 |
| seq.18930.28 | Slit homolog 2 protein | 0.579 | 0.393 |
| seq.18931.40 | Slit homolog 3 protein | 0.656 | 0.926 |
| seq.18932.84 | Nicotinamide/nicotinic acid mononucleotide adenylyltransferase 1 | 0.751 | 0.533 |
| seq.18933.4 | Protein-glutamine gamma-glutamyltransferase 4 | 0.524 | 0.732 |
| seq.18934.50 | Protein-glutamine gamma-glutamyltransferase 2 | 0.223 | 0.000 |
| seq.18935.14 | Toll-like receptor 5 | 0.717 | 0.546 |
| seq.18938.3 | Very low-density lipoprotein receptor | 0.350 | 0.536 |
| seq.18942.11 | Proteasome subunit beta type-9 | 0.546 | 0.271 |
| seq.18943.4 | Prostaglandin reductase 2 | 0.562 | 0.272 |
| seq.18945.11 | Regulator of G-protein signaling 1 | 0.529 | 0.425 |
| seq.18947.3 | Leucine-rich repeat-containing protein 3B | 0.385 | 0.664 |
| seq.18950.13 | Ras-related C3 botulinum toxin substrate 2 | 0.000 | 0.000 |
| seq.19108.50 | Methyl-CpG-binding protein 2 | 0.493 | 0.952 |
| seq.19109.32 | Transcription elongation factor A protein-like 8 | 0.575 | 0.808 |
| seq.19110.6 | Protein MEMO1 | 0.377 | 0.228 |
| seq.19111.10 | NEDD8-conjugating enzyme Ubc12 | 0.456 | 0.514 |
| seq.19112.2 | Transcription elongation factor A protein-like 3 | 0.965 | 0.971 |
| seq.19113.66 | SH3 domain-binding glutamic acid-rich-like protein 2 | 0.017 | 0.000 |
| seq.19114.8 | Thymidine kinase 2, mitochondrial | 0.965 | 0.499 |
| seq.19115.13 | Carboxymethylenebutenolidase homolog | 0.606 | 0.670 |
| seq.19116.1 | Biogenesis of lysosome-related organelles complex 1 subunit 5 | 0.377 | 0.916 |
| seq.19117.3 | Protein phosphatase 1 regulatory subunit 14A | 0.262 | 0.098 |
| seq.19118.47 | Osteoclast-stimulating factor 1 | 0.348 | 0.842 |
| seq.19119.10 | DNA damage-inducible transcript 3 protein | 0.701 | 0.843 |
| seq.19120.33 | D-amino-acid oxidase | 0.884 | 0.157 |
| seq.19121.3 | Ubiquilin-2 | 0.135 | 0.000 |
| seq.19122.47 | Myosin regulatory light chain 12B | 0.324 | 0.018 |
| seq.19123.6 | NF-kappa-B inhibitor delta | 0.415 | 0.152 |
| seq.19124.9 | Ubiquitin-like domain-containing CTD phosphatase 1 | 0.164 | 0.182 |
| seq.19125.26 | cAMP-dependent protein kinase type II-alpha regulatory subunit | 0.585 | 0.750 |
| seq.19126.4 | Regulation of nuclear pre-mRNA domain-containing protein 1B | 0.473 | 0.607 |
| seq.19127.1 | Heat shock protein beta-6 | 0.622 | 0.264 |
| seq.19129.15 | Methenyltetrahydrofolate synthase domain-containing protein | 0.000 | 0.000 |
| seq.19130.81 | Serpin B8 | 0.291 | 0.524 |
| seq.19131.184 | Nucleolysin TIAR | 0.377 | 0.566 |
| seq.19132.1 | 39S ribosomal protein L2, mitochondrial | 0.723 | 0.897 |
| seq.19134.66 | DNA-directed RNA polymerases I, II, and III subunit RPABC4 | 0.246 | 0.000 |
| seq.19135.5 | 7-methylguanosine phosphate-specific 5'-nucleotidase | 0.956 | 0.741 |
| seq.19136.22 | Methylmalonate-semialdehyde dehydrogenase [acylating], mitochondrial | 0.212 | 0.000 |
| seq.19139.3 | Heat shock factor 2-binding protein | 0.631 | 0.726 |
| seq.19141.22 | Death-associated protein 1 | 0.965 | 0.979 |
| seq.19142.39 | DNA (cytosine-5)-methyltransferase 3-like | 0.219 | 0.107 |
| seq.19143.38 | NADH-cytochrome b5 reductase 2 | 0.370 | 0.644 |
| seq.19144.9 | Transcription factor 4 | 0.359 | 0.207 |
| seq.19145.4 | Melanoregulin | 0.793 | 0.641 |
| seq.19147.6 | Galactoside-binding soluble lectin 13 | 0.659 | 0.361 |
| seq.19148.58 | Mitochondrial import inner membrane translocase subunit Tim8 A | 0.333 | 0.000 |
| seq.19150.20 | Phosphoribosyl pyrophosphate synthase-associated protein 2 | 0.252 | 0.090 |
| seq.19152.4 | Protein phosphatase inhibitor 2 | 0.241 | 0.066 |
| seq.19153.53 | Metallophosphoesterase MPPED2 | 0.677 | 0.193 |
| seq.19154.41 | Glia-derived nexin | 0.837 | 0.332 |
| seq.19158.1 | PCNA-associated factor | 0.709 | 0.505 |
| seq.19159.9 | DNA polymerase delta subunit 4 | 0.577 | 0.637 |
| seq.19161.1 | Ubiquitin carboxyl-terminal hydrolase 15 | 0.281 | 0.185 |
| seq.19163.26 | Leucine-rich repeat-containing protein 59 | 0.063 | 0.000 |
| seq.19166.15 | 40S ribosomal protein S19 | 0.083 | 0.463 |
| seq.19168.71 | Proline-serine-threonine phosphatase-interacting protein 1 | 0.000 | 0.701 |
| seq.19169.88 | Acidic fibroblast growth factor intracellular-binding protein | 0.278 | 0.000 |
| seq.19170.25 | DNA polymerase epsilon subunit 3 | 0.529 | 0.057 |
| seq.19173.5 | AN1-type zinc finger protein 1 | 0.000 | 0.000 |
| seq.19174.141 | Golgin subfamily A member 7 | 0.000 | 0.533 |
| seq.19175.18 | MARCKS-related protein | 0.658 | 0.692 |
| seq.19176.27 | Protein FAM49B | 0.179 | 0.053 |
| seq.19177.7 | MOB kinase activator 1B | 0.004 | 0.000 |
| seq.19180.38 | PTB domain-containing engulfment adapter protein 1 | 0.440 | 0.388 |
| seq.19183.164 | 60S ribosomal protein L12 | 0.243 | 0.854 |
| seq.19187.21 | STAM-binding protein | 0.282 | 0.000 |
| seq.19188.21 | nucleosome assembly protein 1-like 4 | 0.069 | 0.000 |
| seq.19189.2 | Ubiquitin carboxyl-terminal hydrolase 14 | 0.282 | 0.329 |
| seq.19190.4 | Ubiquitin/ISG15-conjugating enzyme E2 L6 | 0.080 | 0.292 |
| seq.19193.18 | Prefoldin subunit 3 | 0.448 | 0.415 |
| seq.19194.9 | D-tyrosyl-tRNA(Tyr) deacylase 1 | 0.051 | 0.000 |
| seq.19195.85 | 40S ribosomal protein S5 | 0.219 | 0.523 |
| seq.19196.73 | Homeodomain-only protein | 0.958 | 0.593 |
| seq.19197.95 | Acetyl-CoA acetyltransferase, mitochondrial | 0.278 | 0.000 |
| seq.19199.3 | DNA-directed RNA polymerase II subunit RPB11-a | 0.328 | 0.260 |
| seq.19200.16 | NADH dehydrogenase [ubiquinone] 1 alpha subcomplex subunit 5 | 0.567 | 0.097 |
| seq.19202.10 | Tyrosine-protein kinase BTK | 0.021 | 0.000 |
| seq.19206.20 | 7,8-dihydro-8-oxoguanine triphosphatase | 0.786 | 0.512 |
| seq.19207.119 | Adenosine kinase | 0.390 | 0.127 |
| seq.19208.8 | Activin receptor type-2A | 0.358 | 0.196 |
| seq.19209.6 | NF-kappa-B inhibitor alpha | 0.828 | 0.918 |
| seq.19212.4 | Deleted in malignant brain tumors 1 protein | 0.490 | 0.856 |
| seq.19213.1 | Serine protease inhibitor Kazal-type 4 | 0.824 | 0.615 |
| seq.19215.7 | Protein NDRG1 | 0.957 | 0.748 |
| seq.19219.71 | Nuclear transport factor 2 | 0.217 | 0.453 |
| seq.19222.124 | Ras-related protein Rab-5B | 0.000 | 0.001 |
| seq.19223.6 | Ras-related protein Rab-1A | 0.066 | 0.000 |
| seq.19224.5 | Eukaryotic translation initiation factor 1A, Y-chromosomal | 0.000 | 0.020 |
| seq.19225.11 | B-cell linker protein | 0.452 | 0.092 |
| seq.19227.18 | Basic leucine zipper transcriptional factor ATF-like | 0.465 | 0.819 |
| seq.19228.11 | BAG family molecular chaperone regulator 2 | 0.763 | 0.440 |
| seq.19229.92 | Homer protein homolog 1 | 0.331 | 0.000 |
| seq.19230.12 | Glutathione S-transferase theta-1 | 0.750 | 0.844 |
| seq.19231.22 | Protein tyrosine phosphatase type IVA 3 | 0.530 | 0.697 |
| seq.19233.75 | Copper transport protein ATOX1 | 0.737 | 0.880 |
| seq.19236.24 | Activated RNA polymerase II transcriptional coactivator p15 | 0.322 | 0.523 |
| seq.19237.17 | D-dopachrome decarboxylase | 0.588 | 0.400 |
| seq.19238.12 | Glutamine synthetase | 0.189 | 0.396 |
| seq.19239.5 | ADP-ribosylation factor 1 | 0.836 | 0.952 |
| seq.19240.265 | 28 kDa heat- and acid-stable phosphoprotein | 0.000 | 0.000 |
| seq.19241.31 | Retinol-binding protein 5 | 0.638 | 0.246 |
| seq.19242.21 | Prefoldin subunit 1 | 0.609 | 0.212 |
| seq.19243.2 | Prefoldin subunit 2 | 0.969 | 0.432 |
| seq.19247.1 | Ubiquitin-conjugating enzyme E2 D1 | 0.484 | 0.567 |
| seq.19249.18 | Lysine--tRNA ligase | 0.004 | 0.000 |
| seq.19250.50 | C-Myc-binding protein | 0.000 | 0.000 |
| seq.19251.56 | Serglycin | 0.632 | 0.035 |
| seq.19252.67 | Proteasome subunit beta type-2 | 0.809 | 0.971 |
| seq.19253.82 | Ras-related protein Rab-2B | 0.695 | 0.853 |
| seq.19254.125 | GMP reductase 1 | 0.793 | 0.847 |
| seq.19255.124 | NTF2-related export protein 2 | 0.557 | 0.422 |
| seq.19257.11 | Clathrin light chain A | 0.521 | 0.017 |
| seq.19258.24 | Glutaryl-CoA dehydrogenase, mitochondrial | 0.368 | 0.000 |
| seq.19259.176 | Eukaryotic translation initiation factor 2 subunit 1 | 0.001 | 0.000 |
| seq.19260.4 | PDZ domain-containing protein GIPC1 | 0.592 | 0.876 |
| seq.19261.12 | Syntenin-2 | 0.242 | 0.661 |
| seq.19262.219 | Very long-chain specific acyl-CoA dehydrogenase, mitochondrial | 0.196 | 0.000 |
| seq.19263.147 | Eukaryotic translation initiation factor 4E | 0.000 | 0.160 |
| seq.19264.6 | Replication protein A 32 kDa subunit | 0.865 | 0.969 |
| seq.19265.9 | T-complex protein 1 subunit alpha | 0.615 | 0.172 |
| seq.19266.35 | Ras GTPase-activating protein-binding protein 1 | 0.000 | 0.000 |
| seq.19267.14 | Hydroxyacylglutathione hydrolase, mitochondrial | 0.493 | 0.320 |
| seq.19270.26 | Tyrosine--tRNA ligase, cytoplasmic | 0.000 | 0.000 |
| seq.19271.64 | Guanine nucleotide-binding protein G(i) subunit alpha-1 | 0.889 | 0.368 |
| seq.19272.9 | Peflin | 0.476 | 0.529 |
| seq.19273.3 | Glutathione reductase, mitochondrial | 0.612 | 0.477 |
| seq.19274.80 | Alpha-1-syntrophin | 0.110 | 0.000 |
| seq.19275.68 | Isocitrate dehydrogenase [NAD] subunit gamma, mitochondrial | 0.912 | 0.188 |
| seq.19276.124 | Small nuclear ribonucleoprotein G | 0.245 | 0.000 |
| seq.19277.4 | Thiosulfate sulfurtransferase/rhodanese-like domain-containing protein 1 | 0.298 | 0.208 |
| seq.19278.19 | Ras-related protein Rab-1B | 0.009 | 0.000 |
| seq.19279.42 | Retinol-binding protein 1 | 0.654 | 0.032 |
| seq.19280.29 | Ubiquitin-conjugating enzyme E2 D3 | 0.000 | 0.679 |
| seq.19281.86 | Transcription factor MafG | 0.629 | 0.449 |
| seq.19282.3 | Ras-related protein Rab-13 | 0.563 | 0.578 |
| seq.19286.30 | Myosin light chain 5 | 0.758 | 0.916 |
| seq.19287.59 | Density-regulated protein | 0.439 | 0.000 |
| seq.19289.29 | Uroporphyrinogen decarboxylase | 0.297 | 0.612 |
| seq.19290.5 | Hypoxanthine-guanine phosphoribosyltransferase | 0.441 | 0.233 |
| seq.19291.2 | Calsequestrin-2 | 0.492 | 0.768 |
| seq.19293.6 | Vacuolar protein sorting-associated protein 26A | 0.000 | 0.000 |
| seq.19294.26 | Cysteine-rich PDZ-binding protein | 0.140 | 0.427 |
| seq.19295.32 | Serine/threonine-protein phosphatase PP1-gamma catalytic subunit | 0.022 | 0.000 |
| seq.19296.51 | Myosin regulatory light chain 2, atrial isoform | 0.809 | 0.700 |
| seq.19297.4 | Glucose-6-phosphate 1-dehydrogenase | 0.065 | 0.000 |
| seq.19302.7 | GTPase IMAP family member 6 | 0.348 | 0.184 |
| seq.19303.64 | Protein PET117 homolog, mitochondrial | 0.851 | 0.414 |
| seq.19310.81 | Coatomer subunit epsilon | 0.813 | 0.224 |
| seq.19311.15 | Beta-crystallin S | 0.516 | 0.235 |
| seq.19316.2 | Iron-sulfur cluster co-chaperone protein HscB, mitochondrial | 0.605 | 0.462 |
| seq.19317.114 | Prostatic acid phosphatase | 0.728 | 0.082 |
| seq.19323.1 | Receptor-binding cancer antigen expressed on SiSo cells | 0.937 | 0.919 |
| seq.19325.21 | Protein ABHD14B | 0.853 | 0.740 |
| seq.19327.31 | Histone acetyltransferase type B catalytic subunit | 0.273 | 0.212 |
| seq.19328.51 | Serine/threonine-protein kinase Chk2 | 0.393 | 0.259 |
| seq.19329.31 | Homeodomain-interacting protein kinase 3 | 0.784 | 0.610 |
| seq.19331.18 | Brain-specific angiogenesis inhibitor 1-associated protein 2 | 0.373 | 0.484 |
| seq.19332.1 | MOB-like protein phocein | 0.566 | 0.194 |
| seq.19333.4 | Heterogeneous nuclear ribonucleoprotein K | 0.242 | 0.021 |
| seq.19334.62 | Thioredoxin domain-containing protein 12 | 0.000 | 0.000 |
| seq.19335.2 | Hematological and neurological expressed 1 protein | 0.442 | 0.665 |
| seq.19338.3 | Protein LZIC | 0.613 | 0.103 |
| seq.19341.36 | Acyl-CoA-binding domain-containing protein 6 | 0.000 | 0.015 |
| seq.19347.37 | Carbonic anhydrase 12 | 0.283 | 0.549 |
| seq.19353.25 | Osteopetrosis-associated transmembrane protein 1 | 0.116 | 0.384 |
| seq.19356.20 | TOM1-like protein 2 | 0.520 | 0.518 |
| seq.19357.11 | Vesicle-associated membrane protein 7 | 0.433 | 0.613 |
| seq.19360.22 | Leucine-rich repeat transmembrane neuronal protein 1 | 0.709 | 0.585 |
| seq.19361.78 | Matrilin-3 | 0.568 | 0.387 |
| seq.19364.163 | Proliferating cell nuclear antigen | 0.597 | 0.561 |
| seq.19365.11 | Branched-chain-amino-acid aminotransferase, mitochondrial | 0.427 | 0.000 |
| seq.19366.8 | Peroxisome assembly protein 26 | 0.133 | 0.314 |
| seq.19367.34 | Enoyl-CoA delta isomerase 1, mitochondrial | 0.490 | 0.000 |
| seq.19369.17 | Histone acetyltransferase KAT2A | 0.683 | 0.607 |
| seq.19370.30 | Heparan sulfate glucosamine 3-O-sulfotransferase 4 | 0.294 | 0.838 |
| seq.19371.18 | Microtubule-associated protein RP/EB family member 1 | 0.000 | 0.000 |
| seq.19372.7 | MAM domain-containing glycosylphosphatidylinositol anchor protein 2 | 0.645 | 0.573 |
| seq.19373.3 | Myosin regulatory light chain 12A | 0.113 | 0.000 |
| seq.19374.72 | Protein p13 MTCP-1 | 0.806 | 0.705 |
| seq.19376.74 | Nicotinamide N-methyltransferase | 0.920 | 0.266 |
| seq.19377.14 | Noelin-2 | 0.652 | 0.636 |
| seq.19379.154 | Ras-related protein Rab-3D | 0.196 | 0.000 |
| seq.19381.7 | Regulator of G-protein signaling 21 | 0.860 | 0.908 |
| seq.19383.131 | Cyclin-dependent kinase 2-interacting protein | 0.979 | 0.796 |
| seq.19388.2 | Cadherin-12:Extracellular domain | 0.936 | 0.851 |
| seq.19392.6 | N(G),N(G)-dimethylarginine dimethylaminohydrolase 1 | 0.375 | 0.086 |
| seq.19437.61 | Isoform L-VEGF165 | 0.707 | 0.568 |
| seq.19438.68 | Pre-mRNA-splicing factor SYF2 | 0.901 | 0.895 |
| seq.19446.1 | GMP reductase 2 | 0.567 | 0.145 |
| seq.19448.104 | Uridine phosphorylase 1 | 0.430 | 0.819 |
| seq.19467.3 | Ribokinase | 0.164 | 0.203 |
| seq.19482.11 | Haloacid dehalogenase-like hydrolase domain-containing protein 3 | 0.004 | 0.095 |
| seq.19483.16 | 3-ketodihydrosphingosine reductase | 0.478 | 0.630 |
| seq.19488.1 | Programmed cell death protein 6 | 0.567 | 0.583 |
| seq.19491.11 | Estradiol 17-beta-dehydrogenase 8 | 0.205 | 0.230 |
| seq.19492.5 | Kidney-associated antigen 1 | 0.633 | 0.620 |
| seq.19496.1 | N-acetylneuraminate lyase | 0.752 | 0.429 |
| seq.19503.2 | Sorting nexin-3 | 0.232 | 0.000 |
| seq.19504.22 | Thymidylate kinase | 0.315 | 0.018 |
| seq.19506.6 | Glycine N-acyltransferase | 0.311 | 0.198 |
| seq.19511.8 | DCN1-like protein 2 | 0.817 | 0.251 |
| seq.19516.10 | High mobility group protein HMGI-C | 0.597 | 0.660 |
| seq.19518.12 | Hexokinase-3 | 0.555 | 0.469 |
| seq.19523.215 | Protein DJ-1 | 0.014 | 0.000 |
| seq.19545.145 | Oxidized Protein deglycase DJ-1 | 0.178 | 0.274 |
| seq.19553.14 | Syntaxin-1A | 0.913 | 0.697 |
| seq.19555.1 | Small ubiquitin-related modifier 2 | 0.439 | 0.477 |
| seq.19556.12 | Complement receptor type 1 | 0.758 | 0.525 |
| seq.19557.3 | Beta-klotho | 0.797 | 0.584 |
| seq.19558.10 | Low-density lipoprotein receptor-related protein 4 | 0.943 | 0.463 |
| seq.19560.23 | Plexin-A4 | 0.686 | 0.485 |
| seq.19561.216 | Plexin-D1 | 0.753 | 0.420 |
| seq.19562.8 | Ubiquitin carboxyl-terminal hydrolase 28 | 0.496 | 0.000 |
| seq.19563.3 | Seizure 6-like protein | 0.609 | 0.181 |
| seq.19564.61 | Interferon regulatory factor 4 | 0.710 | 0.816 |
| seq.19567.1 | Epidermal growth factor receptor variant III | 0.925 | 0.934 |
| seq.19568.17 | Interleukin-15 | 0.584 | 0.340 |
| seq.19570.12 | Fibroblast growth factor 8 | 0.749 | 0.264 |
| seq.19572.10 | CCAAT/enhancer-binding protein gamma | 0.804 | 0.634 |
| seq.19574.5 | Integrin alpha-IIb: beta-3 complex | 0.587 | 0.000 |
| seq.19575.4 | Erythropoietin receptor | 0.614 | 0.393 |
| seq.19578.19 | Docking protein 2 | 0.017 | 0.000 |
| seq.19579.5 | Neutrophil defensin 1 | 0.837 | 0.204 |
| seq.19581.15 | Insulin-like growth factor-binding protein 5 | 0.601 | 0.600 |
| seq.19584.33 | Fibroblast growth factor 9 | 0.586 | 0.555 |
| seq.19586.89 | Ras-related protein Rab-3C | 0.000 | 0.000 |
| seq.19587.12 | Importin subunit alpha-5 | 0.694 | 0.240 |
| seq.19590.46 | Pulmonary surfactant-associated protein D | 0.911 | 0.876 |
| seq.19596.18 | Transcription factor IIIB 90 kDa subunit | 0.509 | 0.451 |
| seq.19601.15 | Ankyrin repeat and SOCS box protein 9 | 0.704 | 0.426 |
| seq.19602.36 | Transcription factor jun-D | 0.569 | 0.744 |
| seq.19606.28 | Indian hedgehog protein | 0.533 | 0.311 |
| seq.19612.3 | CD81 antigen | 0.897 | 0.498 |
| seq.19613.16 | Retinol dehydrogenase 10 | 0.507 | 0.133 |
| seq.19614.8 | Transcobalamin-1 | 0.631 | 0.866 |
| seq.19615.213 | Cytosolic 5'-nucleotidase 3A | 0.557 | 0.000 |
| seq.19616.100 | EF-hand domain-containing protein D1 | 0.382 | 0.141 |
| seq.19617.5 | Prostaglandin reductase 1 | 0.534 | 0.151 |
| seq.19620.16 | Disks large homolog 2 | 0.000 | 0.000 |
| seq.19622.7 | Activin A | 0.731 | 0.236 |
| seq.19623.26 | 40S ribosomal protein SA | 0.273 | 0.701 |
| seq.19630.2 | Gamma-synuclein | 0.558 | 0.794 |
| seq.19631.13 | Kininostatin | 0.760 | 0.526 |
| seq.19635.69 | Fibroleukin | 0.435 | 0.052 |
| seq.19636.23 | HLA class I histocompatibility antigen, alpha chain G | 0.314 | 0.419 |
| seq.19637.9 | Corticoliberin | 0.952 | 0.953 |
| seq.19638.9 | Dynorphin A (1-17) | 0.841 | 0.584 |
| seq.19639.53 | Islet amyloid polypeptide | 0.508 | 0.603 |
| seq.19640.2 | Parathyroid Hormone1-34 | 0.925 | 0.647 |
| seq.19742.3 | Septin-5 | 0.070 | 0.128 |
| seq.19743.12 | V-set and transmembrane domain-containing protein 1 | 0.608 | 0.702 |
| seq.19748.3 | Diphosphomevalonate decarboxylase | 0.606 | 0.329 |
| seq.19751.21 | Adenosine deaminase | 0.521 | 0.556 |
| seq.19752.197 | Methionine aminopeptidase 1D, mitochondrial | 0.834 | 0.432 |
| seq.19755.38 | ATPase inhibitor, mitochondrial | 0.562 | 0.245 |
| seq.19760.26 | Carboxypeptidase A1 | 0.369 | 0.129 |
| seq.19765.17 | C-type lectin domain family 4 member G | 0.162 | 0.144 |
| seq.19767.20 | G1/S-specific cyclin-E1 | 0.324 | 0.247 |
| seq.19768.13 | Cystatin B | 0.894 | 0.423 |
| seq.19774.8 | Hemoglobin subunit gamma-2 | 0.717 | 0.725 |
| seq.19784.16 | Lymphocyte antigen 6D | 0.744 | 0.449 |
| seq.19786.26 | Meprin A subunit alpha | 0.856 | 0.406 |
| seq.19787.14 | Proto-oncogene tyrosine-protein kinase MER | 0.630 | 0.687 |
| seq.19788.6 | S-adenosylmethionine synthetase isoform type-2 | 0.000 | 0.089 |
| seq.19794.10 | Uridine diphosphate glucose pyrophosphatase | 0.122 | 0.701 |
| seq.19797.4 | Histone acetyltransferase KAT2A | 0.670 | 0.000 |
| seq.19799.2 | Probable phosphoglycerate mutase 4 | 0.314 | 0.526 |
| seq.19802.22 | Receptor-binding cancer antigen expressed on SiSo cells | 0.605 | 0.000 |
| seq.19803.34 | Retinol-binding protein 2 | 0.902 | 0.418 |
| seq.19808.26 | Ras-related protein Rab-3D | 0.446 | 0.000 |
| seq.19809.47 | DNA-directed RNA polymerase II subunit RPB9 | 0.000 | 0.445 |
| seq.19819.7 | Regulator of G-protein signaling 21 | 0.000 | 0.146 |
| seq.19821.10 | Peptidoglycan recognition protein I-beta | 0.067 | 0.478 |
| seq.19823.75 | NAD-dependent protein deacetylase sirtuin-1 | 0.515 | 0.656 |
| seq.19824.3 | Ubiquitin-conjugating enzyme E2 R2 | 0.264 | 0.234 |
| seq.20054.28 | Cytoplasmic aconitate hydratase | 0.490 | 0.304 |
| seq.20055.40 | Retinal dehydrogenase 2 | 0.807 | 0.794 |
| seq.20056.7 | Frizzled-10:Frizzled domain | 0.843 | 0.407 |
| seq.20057.177 | Histone chaperone ASF1B | 0.779 | 0.514 |
| seq.20064.24 | Fibronectin-1 Fragment 2 | 0.233 | 0.209 |
| seq.20066.19 | Frizzled-5 | 0.685 | 0.637 |
| seq.20067.26 | Factor seven-activating protease | 0.702 | 0.528 |
| seq.20068.61 | G antigen 12F | 0.396 | 0.749 |
| seq.20069.23 | Glycine N-acyltransferase-like protein 2 | 0.910 | 0.911 |
| seq.20071.53 | I-kappa-B-epsilon | 0.439 | 0.642 |
| seq.20073.22 | GTPase KRas | 0.346 | 0.413 |
| seq.20074.3 | Laminin subunit alpha-3 | 0.700 | 0.476 |
| seq.20075.130 | Melanoma-associated antigen 4 | 0.931 | 0.703 |
| seq.20076.7 | Myeloid cell nuclear differentiation antigen | 0.122 | 0.304 |
| seq.20078.6 | Myosin light chain 6B | 0.408 | 0.342 |
| seq.20079.6 | Phosphoenolpyruvate carboxykinase [GTP], mitochondrial | 0.298 | 0.496 |
| seq.20081.14 | Serine/threonine-protein kinase Pim-2 | 0.408 | 0.040 |
| seq.20083.1 | Ras-related protein Rab-6A | 0.112 | 0.000 |
| seq.20086.5 | Ras-related protein Rab-11B | 0.075 | 0.000 |
| seq.20087.3 | 15 kDa selenoprotein | 0.352 | 0.363 |
| seq.20089.172 | Sulfotransferase 1C4 | 0.725 | 0.305 |
| seq.20090.63 | Ubiquitin-conjugating enzyme E2 E3 | 0.232 | 0.129 |
| seq.20091.138 | Ephrin-A1 | 0.731 | 0.328 |
| seq.20093.9 | Vascular cell adhesion protein 1 | 0.807 | 0.634 |
| seq.20103.176 | N-glycosylase/DNA lyase | 0.098 | 0.000 |
| seq.20105.7 | Myosin light polypeptide 6 | 0.264 | 0.000 |
| seq.20106.80 | Scavenger mRNA-decapping enzyme DcpS | 0.472 | 0.850 |
| seq.20107.11 | Myosin light polypeptide 4 | 0.458 | 0.000 |
| seq.20110.25 | Hematological and neurological expressed 1-like protein | 0.497 | 0.000 |
| seq.20111.5 | Brain acid soluble protein 1 | 0.887 | 0.408 |
| seq.20116.30 | Serine/threonine-protein kinase OSR1 | 0.306 | 0.015 |
| seq.20117.5 | Eukaryotic translation initiation factor 4E-binding protein 2 | 0.521 | 0.506 |
| seq.20120.101 | Amphoterin-induced protein 1:Extracellular domain | 0.874 | 0.396 |
| seq.20126.19 | Glial fibrillary acidic protein | 0.701 | 0.866 |
| seq.20127.102 | Hephaestin | 0.925 | 0.641 |
| seq.20128.1 | cAMP response element-binding protein | 0.962 | 0.000 |
| seq.20130.144 | Casein kinase I isoform gamma-1 | 0.020 | 0.000 |
| seq.20133.1 | Melanoma-associated antigen 3 | 0.724 | 0.590 |
| seq.20134.27 | Macrophage receptor MARCO | 0.668 | 0.525 |
| seq.20135.85 | mRNA-capping enzyme | 0.000 | 0.243 |
| seq.20137.49 | Heterogeneous nuclear ribonucleoprotein A1 | 0.110 | 0.161 |
| seq.20139.57 | Endophilin-A1 | 0.039 | 0.000 |
| seq.20141.42 | Transcription elongation factor A protein 2 | 0.626 | 0.711 |
| seq.20142.42 | Ubiquitin-conjugating enzyme E2 C | 0.526 | 0.377 |
| seq.20159.1 | Neuritin-like protein | 0.754 | 0.506 |
| seq.20161.41 | Neural cell adhesion molecule 1 | 0.717 | 0.212 |
| seq.20165.4 | Dihydropyrimidinase | 0.590 | 0.462 |
| seq.20167.6 | Ras-related protein Rab-8B | 0.000 | 0.000 |
| seq.20173.39 | Calpain-9 | 0.405 | 0.093 |
| seq.20175.17 | Collagen alpha-3(IX) chain | 0.958 | 0.533 |
| seq.20181.17 | Integrin alpha-11 | 0.153 | 0.386 |
| seq.20183.48 | Homeobox protein MOX-1 | 0.073 | 0.000 |
| seq.20185.44 | Dihydrolipoyllysine-residue acetyltransferase component of pyruvate dehydrogenase complex, mitochondrial | 0.880 | 0.054 |
| seq.20187.10 | Integrin alpha V beta 3 | 0.716 | 0.532 |
| seq.20189.28 | Integrin alpha V beta 6 | 0.559 | 0.757 |
| seq.20189.4 | Integrin alpha V beta 6 | 0.141 | 0.000 |
| seq.20191.13 | Integrin alpha V beta 8 | 0.361 | 0.731 |
| seq.20195.13 | Interferon-induced helicase C domain-containing protein 1 | 0.841 | 0.115 |
| seq.20197.14 | Visinin-like protein 1 | 0.277 | 0.697 |
| seq.20203.45 | Complement C1s subcomponent | 0.641 | 0.628 |
| seq.20205.55 | Dual specificity tyrosine-phosphorylation-regulated kinase 2 | 0.343 | 0.058 |
| seq.20211.75 | Guanylate-binding protein 5 | 0.400 | 0.391 |
| seq.20213.82 | Heat shock 70 kDa protein 6 | 0.804 | 0.691 |
| seq.20215.45 | Integrin alpha-V: beta-1 complex | 0.613 | 0.704 |
| seq.20217.26 | Lamin-B2 | 0.699 | 0.615 |
| seq.20219.25 | Neprilysin | 0.918 | 0.248 |
| seq.20221.26 | cAMP-specific 3',5'-cyclic phosphodiesterase 4C | 0.304 | 0.744 |
| seq.20225.119 | Signal transducer and activator of transcription 5A | 0.301 | 0.572 |
| seq.20229.67 | TM2 domain-containing protein 1 | 0.984 | 0.729 |
| seq.20231.23 | Uridine phosphorylase 2 | 0.672 | 0.658 |
| seq.20237.38 | Ornithine aminotransferase, mitochondrial | 0.382 | 0.653 |
| seq.20241.9 | Synaptosomal-associated protein 23 | 0.412 | 0.294 |
| seq.20243.26 | Profilin-1 | 0.000 | 0.000 |
| seq.20245.13 | Retinoic acid receptor RXR-alpha | 0.000 | 0.289 |
| seq.20247.17 | Dihydropyrimidinase-related protein 4 | 0.576 | 0.209 |
| seq.20367.6 | L-amino-acid oxidase | 0.631 | 0.914 |
| seq.20370.6 | Acylphosphatase-1 | 0.136 | 0.176 |
| seq.20373.141 | Glucose-induced degradation protein 8 homolog | 0.611 | 0.863 |
| seq.20374.41 | Ubiquinone biosynthesis protein COQ9, mitochondrial | 0.190 | 0.474 |
| seq.20376.64 | Putative deoxyribonuclease TATDN1 | 0.453 | 0.067 |
| seq.20378.110 | Leucine zipper transcription factor-like protein 1 | 0.445 | 0.049 |
| seq.20379.59 | Small vasohibin-binding protein | 0.823 | 0.699 |
| seq.20380.50 | Protein LSM12 homolog | 0.890 | 0.821 |
| seq.20381.21 | PIH1 domain-containing protein 2 | 0.466 | 0.468 |
| seq.20382.8 | Motile sperm domain-containing protein 1 | 0.198 | 0.811 |
| seq.20383.31 | Probable RNA-binding protein 18 | 0.702 | 0.376 |
| seq.20385.21 | Ceramide-1-phosphate transfer protein | 0.693 | 0.035 |
| seq.20386.15 | Putative peptidyl-tRNA hydrolase PTRHD1 | 0.156 | 0.000 |
| seq.20387.277 | Coiled-coil-helix-coiled-coil-helix domain-containing protein 7 | 0.607 | 0.448 |
| seq.20388.8 | Pre-rRNA-processing protein TSR2 homolog | 0.383 | 0.269 |
| seq.20389.36 | Mitochondrial inner membrane protease subunit 2 | 0.871 | 0.639 |
| seq.20390.4 | High mobility group nucleosome-binding domain-containing protein 3 | 0.342 | 0.208 |
| seq.20393.10 | Retinal cone rhodopsin-sensitive cGMP 3',5'-cyclic phosphodiesterase subunit gamma | 0.446 | 0.703 |
| seq.20396.70 | COMM domain-containing protein 6 | 0.000 | 0.347 |
| seq.20398.60 | Ribonuclease P protein subunit p20 | 0.413 | 0.624 |
| seq.20399.7 | Oxidoreductase-like domain-containing protein 1 | 0.842 | 0.251 |
| seq.20401.19 | Protein BUD31 homolog | 0.000 | 0.369 |
| seq.20402.11 | Trafficking protein particle complex subunit 2 | 0.684 | 0.000 |
| seq.20408.11 | Mitochondrial import receptor subunit TOM20 homolog | 0.166 | 0.000 |
| seq.20411.52 | Tetratricopeptide repeat protein 33 | 0.825 | 0.510 |
| seq.20423.40 | Calcium and integrin-binding protein 1 | 0.446 | 0.563 |
| seq.20425.12 | Hematopoietically-expressed homeobox protein HHEX | 0.869 | 0.778 |
| seq.20426.34 | Protein FAM84A | 0.000 | 0.706 |
| seq.20427.18 | Mediator of RNA polymerase II transcription subunit 20 | 0.543 | 0.551 |
| seq.20428.5 | Phosphoethanolamine/phosphocholine phosphatase | 0.088 | 0.526 |
| seq.20430.8 | C-type natriuretic peptide | 0.763 | 0.438 |
| seq.20432.6 | Pyroglutamyl-peptidase 1 | 0.195 | 0.901 |
| seq.20433.19 | 39S ribosomal protein L1, mitochondrial | 0.187 | 0.000 |
| seq.20434.23 | Purkinje cell protein 4-like protein 1 | 0.966 | 0.854 |
| seq.20435.41 | ER membrane protein complex subunit 2 | 0.509 | 0.528 |
| seq.20436.93 | Tubulin polyglutamylase complex subunit 2 | 0.000 | 0.039 |
| seq.20437.9 | Axonemal dynein light intermediate polypeptide 1 | 0.261 | 0.551 |
| seq.20439.14 | Proline synthase co-transcribed bacterial homolog protein | 0.090 | 0.000 |
| seq.20440.36 | Tetratricopeptide repeat protein 32 | 0.785 | 0.875 |
| seq.20441.35 | DNA-directed RNA polymerases I, II, and III subunit RPABC1 | 0.395 | 0.272 |
| seq.20442.12 | Calcipressin-3 | 0.829 | 0.796 |
| seq.20443.37 | CST complex subunit TEN1 | 0.658 | 0.263 |
| seq.20444.12 | Tubulin polymerization-promoting protein family member 3 | 0.503 | 0.572 |
| seq.20445.29 | AP-1 complex-associated regulatory protein | 0.543 | 0.408 |
| seq.20447.11 | Homeobox protein TGIF2LY | 0.691 | 0.000 |
| seq.20448.7 | DNA-directed RNA polymerases I, II, and III subunit RPABC2 | 0.832 | 0.953 |
| seq.20449.72 | Josephin-1 | 0.511 | 0.300 |
| seq.20451.126 | Intraflagellar transport protein 22 homolog | 0.169 | 0.059 |
| seq.20453.9 | Non-structural maintenance of chromosomes element 1 homolog | 0.977 | 0.630 |
| seq.20454.24 | SOSS complex subunit B2 | 0.703 | 0.252 |
| seq.20457.13 | Cytochrome c oxidase assembly factor 4 homolog, mitochondrial | 0.891 | 0.332 |
| seq.20458.22 | NADH dehydrogenase [ubiquinone] 1 alpha subcomplex subunit 2 | 0.377 | 0.060 |
| seq.20460.22 | Intraflagellar transport protein 20 homolog | 0.953 | 0.306 |
| seq.20461.58 | Protein FAM50A | 0.596 | 0.522 |
| seq.20463.315 | Protein IMPACT | 0.182 | 0.085 |
| seq.20464.7 | Putative KHDC1-like protein | 0.252 | 0.731 |
| seq.20511.3 | Junctional adhesion molecule A | 0.626 | 0.191 |
| seq.20512.2 | Melanoma-associated antigen MUC18 | 0.719 | 0.737 |
| seq.20514.8 | BMP and activin membrane-bound inhibitor homolog:Extracellular domain | 0.873 | 0.362 |
| seq.20516.11 | Sodium channel subunit beta-3 | 0.832 | 0.176 |
| seq.20517.1 | Desmoglein-2 | 0.466 | 0.156 |
| seq.20518.11 | Tumor necrosis factor receptor superfamily member 25 | 0.774 | 0.734 |
| seq.20519.7 | CD6 | 0.252 | 0.000 |
| seq.20520.14 | Protein FAM3C | 0.489 | 0.406 |
| seq.20521.83 | CD28 | 0.659 | 0.354 |
| seq.20522.2 | Interleukin-1 receptor accessory protein-like 1 | 0.677 | 0.878 |
| seq.20524.38 | Pro-neuregulin-3, membrane-bound isoform | 0.458 | 0.851 |
| seq.20525.200 | Protein disulfide-isomerase A4 | 0.188 | 0.000 |
| seq.20526.3 | Retinoic acid early transcript 1L protein | 0.778 | 0.420 |
| seq.20527.47 | Semaphorin-5B | 0.718 | 0.795 |
| seq.20528.23 | Macrosialin | 0.844 | 0.405 |
| seq.20529.7 | Cadherin-10 | 0.844 | 0.713 |
| seq.20530.2 | Semaphorin-6D | 0.440 | 0.219 |
| seq.20531.5 | SLAM family member 9 | 0.444 | 0.872 |
| seq.20533.39 | Interleukin-35 | 0.878 | 0.430 |
| seq.20534.6 | Scavenger receptor class B member 1 | 0.837 | 0.608 |
| seq.20535.68 | Interleukin-17 receptor E | 0.481 | 0.289 |
| seq.20536.11 | Endothelial cell-selective adhesion molecule | 0.709 | 0.335 |
| seq.20538.71 | Sialic acid-binding Ig-like lectin 11:Extracellular domain, Isoform 1 | 0.917 | 0.814 |
| seq.20539.4 | Neutrophil defensin 3 | 0.769 | 0.742 |
| seq.20540.65 | Semaphorin-4G | 0.658 | 0.503 |
| seq.20541.73 | T-cell surface glycoprotein CD1b | 0.396 | 0.251 |
| seq.20542.47 | Calcitonin receptor | 0.806 | 0.932 |
| seq.20543.19 | Beta-defensin 127 | 0.518 | 0.444 |
| seq.20544.103 | Glutamate receptor ionotropic, kainate 2 | 0.905 | 0.704 |
| seq.20545.17 | Microfibril-associated glycoprotein 3 | 0.835 | 0.832 |
| seq.20546.71 | Reticulon-4 receptor-like 1 | 0.751 | 0.516 |
| seq.20547.5 | Neuronal acetylcholine receptor subunit beta-3 | 0.349 | 0.688 |
| seq.20549.1 | Izumo sperm-egg fusion protein 4 | 0.914 | 0.741 |
| seq.20550.38 | Neurotrimin | 0.643 | 0.211 |
| seq.20553.2 | Phosphoinositide-3-kinase-interacting protein 1 | 0.960 | 0.714 |
| seq.20557.19 | V-set and immunoglobulin domain-containing protein 8 | 0.482 | 0.776 |
| seq.20558.4 | Ephrin type-A receptor 8 | 0.337 | 0.968 |
| seq.20561.15 | Contactin-6 | 0.779 | 0.618 |
| seq.20562.78 | Killer cell lectin-like receptor subfamily B member 1 | 0.795 | 0.926 |
| seq.20563.48 | Toll-like receptor 10 | 0.546 | 0.409 |
| seq.20564.53 | Thy-1 membrane glycoprotein | 0.594 | 0.403 |
| seq.20568.3 | FRAS1-related extracellular matrix protein 1 | 0.558 | 0.513 |
| seq.20570.18 | V-set and transmembrane domain-containing protein 1 | 0.841 | 0.718 |
| seq.20572.6 | Proto-oncogene tyrosine-protein kinase MER | 0.470 | 0.582 |
| seq.20574.8 | CD81 antigen | 0.712 | 0.947 |
| seq.20575.82 | C-type lectin domain family 9 member A | 0.728 | 0.878 |
| seq.20576.71 | Frizzled-7 | 0.633 | 0.301 |
| seq.20577.5 | Frizzled-8 | 0.664 | 0.373 |
| seq.20578.10 | Latrophilin-3 | 0.636 | 0.212 |
| seq.20579.50 | Myelin-associated glycoprotein | 0.524 | 0.470 |
| seq.20581.42 | Glucagon-like peptide 1 receptor:Extracellular domain | 0.921 | 0.368 |
| seq.20584.4 | Nectin-1, isoform gamma:Extracellular domain | 0.677 | 0.245 |
| seq.20585.5 | CMRF35-like molecule 9 | 0.856 | 0.720 |
| seq.20585.9 | CMRF35-like molecule 9 | 0.828 | 0.275 |
| seq.20586.18 | Contactin-3 | 0.818 | 0.402 |
| seq.20589.5 | Cadherin-4 | 0.881 | 0.357 |
| seq.20590.13 | Neuropeptide Y | 0.714 | 0.581 |
| seq.20591.48 | Gliomedin | 0.667 | 0.447 |
| seq.20592.8 | Latexin | 0.611 | 0.473 |
| seq.20593.10 | Beta-1,3-galactosyltransferase 5 | 0.381 | 0.896 |
| seq.20912.10 | Pseudouridine-5'-phosphatase | 0.702 | 0.492 |
| seq.20913.27 | Eukaryotic translation initiation factor 1 | 0.889 | 0.789 |
| seq.20915.68 | Adaptin ear-binding coat-associated protein 2 | 0.156 | 0.000 |
| seq.20918.28 | U1 small nuclear ribonucleoprotein C | 0.898 | 0.917 |
| seq.20921.1 | PDZ domain-containing protein GIPC2 | 0.578 | 0.009 |
| seq.20922.4 | Profilin-4 | 0.412 | 0.799 |
| seq.20923.10 | Magnesium-dependent phosphatase 1 | 0.000 | 0.000 |
| seq.20924.85 | Transcription elongation factor A protein-like 1 | 0.572 | 0.427 |
| seq.20926.31 | Cyclic AMP-dependent transcription factor ATF-1 | 0.589 | 0.185 |
| seq.20927.43 | General transcription factor IIF subunit 2 | 0.595 | 0.722 |
| seq.20928.39 | Transcription elongation factor A protein-like 7 | 0.783 | 0.276 |
| seq.20929.4 | CTD small phosphatase-like protein | 0.217 | 0.000 |
| seq.20931.156 | Methylglutaconyl-CoA hydratase, mitochondrial | 0.675 | 0.572 |
| seq.20932.10 | Coiled-coil domain-containing protein 69 | 0.266 | 0.069 |
| seq.20934.13 | Ras-related protein Rab-24 | 0.299 | 0.000 |
| seq.20935.4 | Phosducin-like protein 3 | 0.791 | 0.000 |
| seq.20936.8 | Putative deoxyribonuclease TATDN3 | 0.485 | 0.407 |
| seq.20937.43 | Ribosomal RNA small subunit methyltransferase NEP1 | 0.951 | 0.851 |
| seq.20939.113 | H/ACA ribonucleoprotein complex subunit 2 | 0.427 | 0.892 |
| seq.20941.7 | Ubiquitin-like protein 3 | 0.388 | 0.651 |
| seq.20942.4 | DnaJ homolog subfamily B member 8 | 0.315 | 0.000 |
| seq.20943.14 | N-alpha-acetyltransferase 50 | 0.000 | 0.000 |
| seq.20946.41 | Gametocyte-specific factor 1 | 0.650 | 0.328 |
| seq.20947.50 | Protein Dr1 | 0.066 | 0.179 |
| seq.20948.16 | U8 snoRNA-decapping enzyme | 0.199 | 0.000 |
| seq.20950.159 | Phosphoribosyltransferase domain-containing protein 1 | 0.000 | 0.000 |
| seq.20952.15 | Ethylmalonyl-CoA decarboxylase | 0.124 | 0.061 |
| seq.20953.34 | Methylmalonic aciduria and homocystinuria type C protein | 0.274 | 0.282 |
| seq.20954.27 | Protein LDOC1L | 0.896 | 0.787 |
| seq.20955.20 | tRNA-specific adenosine deaminase 2 | 0.692 | 0.735 |
| seq.20956.13 | Prefoldin subunit 4 | 0.334 | 0.165 |
| seq.20957.57 | Diphosphoinositol polyphosphate phosphohydrolase 3-alpha | 0.936 | 0.750 |
| seq.20958.13 | Ras-related protein Rab-23 | 0.431 | 0.000 |
| seq.20959.12 | Dual specificity protein phosphatase 18 | 0.815 | 0.982 |
| seq.20960.47 | Far upstream element-binding protein 1 | 0.210 | 0.174 |
| seq.20964.13 | 40S ribosomal protein S14 | 0.189 | 0.441 |
| seq.20965.18 | Microtubule-associated proteins 1A/1B light chain 3 beta 2 | 0.570 | 0.781 |
| seq.20966.7 | Sentrin-specific protease 8 | 0.518 | 0.821 |
| seq.20967.137 | Proteasome assembly chaperone 4 | 0.437 | 0.818 |
| seq.20968.22 | Exosome complex component RRP46 | 0.860 | 0.779 |
| seq.20969.114 | RING1 and YY1-binding protein | 0.734 | 0.669 |
| seq.20970.14 | Dysbindin domain-containing protein 1 | 0.973 | 0.906 |
| seq.20971.1 | Methionine-R-sulfoxide reductase B1 | 0.034 | 0.178 |
| seq.20972.37 | Cleavage and polyadenylation specificity factor subunit 5 | 0.203 | 0.003 |
| seq.20975.2 | Calcipressin-2 | 0.918 | 0.972 |
| seq.20976.19 | U6 snRNA-associated Sm-like protein LSm3 | 0.895 | 0.000 |
| seq.20977.2 | Phosducin-like protein | 0.979 | 0.695 |
| seq.20979.86 | Transcription initiation factor IIB | 0.718 | 0.331 |
| seq.20982.29 | U11/U12 small nuclear ribonucleoprotein 25 kDa protein | 0.760 | 0.849 |
| seq.20984.142 | Dual specificity protein phosphatase 19 | 0.109 | 0.054 |
| seq.20986.58 | Egl nine homolog 3 | 0.383 | 0.582 |
| seq.20987.21 | Homeobox protein SIX6 | 0.984 | 0.296 |
| seq.20988.63 | Retinitis pigmentosa 9 protein | 0.748 | 0.656 |
| seq.20990.48 | Thyrotroph embryonic factor | 0.758 | 0.377 |
| seq.20991.2 | LIM domain-containing protein 2 | 0.174 | 0.079 |
| seq.20993.13 | BEN domain-containing protein 6 | 0.670 | 0.426 |
| seq.20994.37 | Pleckstrin homology domain-containing family F member 2 | 0.000 | 0.000 |
| seq.20995.47 | DNA-directed RNA polymerase II subunit RPB3 | 0.878 | 0.859 |
| seq.20996.107 | Succinate dehydrogenase assembly factor 1, mitochondrial | 0.764 | 0.354 |
| seq.20998.19 | Nicotinamide riboside kinase 2; Short=NRK 2; Short=NmR-K 2; EC=2.7.1.22 | 0.892 | 0.909 |
| seq.20999.12 | 60S ribosomal protein L5 | 0.314 | 0.628 |
| seq.21000.40 | DNA-directed RNA polymerase II subunit RPB11-b1 | 0.000 | 0.255 |
| seq.21001.393 | Protein BRICK1 | 0.656 | 0.582 |
| seq.21002.1 | NudC domain-containing protein 2 | 0.340 | 0.132 |
| seq.21008.113 | Protein mago nashi homolog | 0.246 | 0.167 |
| seq.21009.28 | Sentan | 0.885 | 0.878 |
| seq.21104.37 | Ubiquitin-like protein 5 | 0.184 | 0.102 |
| seq.21105.23 | Protein unc-119 homolog B | 0.816 | 0.424 |
| seq.21106.206 | Centrin-1 | 0.835 | 0.688 |
| seq.21107.5 | Complex I intermediate-associated protein 30, mitochondrial | 0.921 | 0.768 |
| seq.21108.5 | Uncharacterized metallophosphoesterase CSTP1 | 0.767 | 0.889 |
| seq.21109.1 | Cilia- and flagella-associated protein 36 | 0.492 | 0.333 |
| seq.21110.5 | Ubiquitin-conjugating enzyme E2 Z | 0.310 | 0.340 |
| seq.21111.49 | Hsp70-binding protein 1 | 0.828 | 0.634 |
| seq.21112.6 | Myosin regulatory light chain 2, skeletal muscle isoform | 0.657 | 0.452 |
| seq.21113.3 | TRAF-interacting protein with FHA domain-containing protein A | 0.473 | 0.669 |
| seq.21114.18 | DnaJ homolog subfamily C member 12 | 0.029 | 0.432 |
| seq.21115.48 | Pre-miRNA 5'-monophosphate methyltransferase | 0.868 | 0.641 |
| seq.21116.13 | Polycomb protein EED | 0.885 | 0.728 |
| seq.21117.18 | Zinc finger C4H2 domain-containing protein | 0.858 | 0.967 |
| seq.21118.48 | GTP cyclohydrolase I feedback regulatory protein | 0.458 | 0.428 |
| seq.21119.1 | Carbonyl reductase family member 4 | 0.921 | 0.945 |
| seq.21120.3 | Mimitin, mitochondrial | 0.231 | 0.000 |
| seq.21121.31 | NEDD8-activating enzyme E1 catalytic subunit | 0.173 | 0.688 |
| seq.21122.3 | Dysbindin domain-containing protein 2 | 0.509 | 0.629 |
| seq.21123.1 | 39S ribosomal protein L28, mitochondrial | 0.828 | 0.916 |
| seq.21124.17 | Yae1 domain-containing protein 1 | 0.928 | 0.866 |
| seq.21126.27 | Glutamine--fructose-6-phosphate aminotransferase [isomerizing] 1 | 0.356 | 0.000 |
| seq.21128.2 | CDK2 | 0.294 | 0.077 |
| seq.21129.95 | MAD2L1-binding protein | 0.376 | 0.127 |
| seq.21130.82 | Inositol-tetrakisphosphate 1-kinase | 0.779 | 0.668 |
| seq.21131.109 | tRNA wybutosine-synthesizing protein 5 | 0.614 | 0.082 |
| seq.21132.9 | Synaptotagmin-13 | 0.642 | 0.539 |
| seq.21133.33 | Cx9C motif-containing protein 4 | 0.936 | 0.965 |
| seq.21134.9 | CREB/ATF bZIP transcription factor | 0.891 | 0.793 |
| seq.21135.16 | Splicing factor 3B subunit 6 | 0.258 | 0.847 |
| seq.21136.1 | Proteasome assembly chaperone 2 | 0.866 | 0.799 |
| seq.21138.2 | Nucleoplasmin-2 | 0.387 | 0.108 |
| seq.21140.19 | Calcium signal-modulating cyclophilin ligand | 0.277 | 0.186 |
| seq.21141.9 | Protein FAM84B | 0.718 | 0.693 |
| seq.21143.10 | Calcyphosin | 0.896 | 0.930 |
| seq.21144.160 | Serine/threonine-protein kinase Sgk1 | 0.418 | 0.105 |
| seq.21146.187 | Tubby-related protein 1 | 0.610 | 0.783 |
| seq.21147.9 | Fructosamine-3-kinase | 0.416 | 0.284 |
| seq.21149.27 | CUE domain-containing protein 1 | 0.341 | 0.573 |
| seq.21152.25 | Eyes absent homolog 2 | 0.211 | 0.660 |
| seq.21153.5 | Chromatin complexes subunit BAP18 | 0.239 | 0.000 |
| seq.21154.8 | Calcium-binding protein 39-like | 0.353 | 0.027 |
| seq.21156.5 | Putative glutathione-specific gamma-glutamylcyclotransferase 2 | 0.791 | 0.456 |
| seq.21157.6 | Hydroxylysine kinase | 0.688 | 0.795 |
| seq.21160.4 | Charged multivesicular body protein 6 | 0.753 | 0.000 |
| seq.21161.16 | rRNA methyltransferase 2, mitochondrial | 0.676 | 0.475 |
| seq.21162.30 | Tubulin-specific chaperone cofactor E-like protein | 0.333 | 0.090 |
| seq.21163.21 | TBC1 domain family member 13 | 0.000 | 0.000 |
| seq.21164.83 | Homeobox protein TGIF2LX | 0.800 | 0.731 |
| seq.21166.1 | Carboxy-terminal domain RNA polymerase II polypeptide A small phosphatase 1 | 0.132 | 0.000 |
| seq.21167.33 | Phosphatidylglycerophosphatase and protein-tyrosine phosphatase 1 | 0.498 | 0.633 |
| seq.21172.11 | Mesoderm development candidate 1 | 0.441 | 0.309 |
| seq.21173.25 | ELAV-like protein 2 | 0.861 | 0.336 |
| seq.21178.8 | Probable cytosolic iron-sulfur protein assembly protein CIAO1 | 0.067 | 0.175 |
| seq.21180.16 | PI-PLC X domain-containing protein 3 | 0.774 | 0.665 |
| seq.21181.10 | LRP2-binding protein | 0.000 | 0.534 |
| seq.21182.8 | tRNA-splicing endonuclease subunit Sen15 | 0.811 | 0.779 |
| seq.21183.1 | Dual specificity protein phosphatase 10 | 0.540 | 0.836 |
| seq.21184.1 | Biogenesis of lysosome-related organelles complex 1 subunit 2 | 0.952 | 0.933 |
| seq.21188.38 | RWD domain-containing protein 4 | 0.000 | 0.000 |
| seq.21189.9 | Nucleotide-binding protein 1 | 0.298 | 0.000 |
| seq.21190.4 | Ubiquitin-conjugating enzyme E2 W | 0.964 | 0.855 |
| seq.21191.24 | Dual specificity protein phosphatase 21 | 0.492 | 0.761 |
| seq.21193.1 | Dimethyladenosine transferase 2, mitochondrial | 0.717 | 0.000 |
| seq.21194.39 | POU domain, class 6, transcription factor 1 | 0.586 | 0.855 |
| seq.21196.29 | SAM domain-containing protein SAMSN-1 | 0.118 | 0.369 |
| seq.21203.14 | MORF4 family-associated protein 1-like 1 | 0.712 | 0.586 |
| seq.21204.70 | Desumoylating isopeptidase 1 | 0.975 | 0.328 |
| seq.21206.218 | HCLS1-associated protein X-1 | 0.000 | 0.264 |
| seq.21207.1 | Bifunctional coenzyme A synthase | 0.644 | 0.760 |
| seq.21208.163 | CCAAT/enhancer-binding protein epsilon | 0.559 | 0.338 |
| seq.21210.33 | DNA polymerase beta | 0.656 | 0.873 |
| seq.21217.20 | Prohibitin-2 | 0.560 | 0.523 |
| seq.21219.7 | RPA-interacting protein | 0.702 | 0.032 |
| seq.21220.11 | GTP-binding protein SAR1b | 0.858 | 0.831 |
| seq.21221.67 | Serpin B9 | 0.209 | 0.000 |
| seq.21227.18 | SULT 1A1*2 | 0.530 | 0.244 |
| seq.21229.5 | Transmembrane and immunoglobulin domain-containing protein 2 | 0.869 | 0.826 |
| seq.21231.3 | Neuronal acetylcholine receptor subunit alpha-5 | 0.694 | 0.241 |
| seq.21232.39 | Benign Prostate specific Antigen | 0.860 | 0.954 |
| seq.21234.7 | Ankyrin repeat and SOCS box protein 13 | 0.214 | 0.098 |
| seq.21235.11 | Importin subunit alpha-6 | 0.000 | 0.000 |
| seq.21236.12 | Transmembrane emp24 domain-containing protein 9 | 0.436 | 0.202 |
| seq.21237.24 | Protein-arginine deiminase type-4 | 0.629 | 0.367 |
| seq.21238.40 | CDK5 regulatory subunit-associated protein 3 | 0.543 | 0.522 |
| seq.21239.31 | Cytohesin-1 | 0.197 | 0.228 |
| seq.21240.6 | Aminomethyltransferase, mitochondrial | 0.200 | 0.141 |
| seq.21241.1 | N-acetyltransferase 6 | 0.180 | 0.000 |
| seq.21244.57 | Nucleophosmin | 0.876 | 0.333 |
| seq.21247.16 | S-adenosylmethionine synthase isoform type-1 | 0.186 | 0.116 |
| seq.21249.115 | DNA fragmentation factor subunit alpha | 0.754 | 0.415 |
| seq.21255.2 | Regulator of G-protein signaling 16 | 0.818 | 0.546 |
| seq.21260.74 | Small nuclear ribonucleoprotein E | 0.927 | 0.000 |
| seq.21269.198 | Epoxide hydrolase 2 | 0.926 | 0.441 |
| seq.21271.53 | Aldo-keto reductase family 1 member C2 | 0.718 | 0.877 |
| seq.21272.100 | UPF0568 protein C14orf166 | 0.971 | 0.761 |
| seq.21276.11 | CD133 antigen | 0.473 | 0.508 |
| seq.21280.13 | TSC22 domain family protein 3 | 0.983 | 0.871 |
| seq.21281.13 | Calcium/calmodulin-dependent protein kinase II inhibitor 2 | 0.310 | 0.115 |
| seq.21282.21 | TNFAIP3-interacting protein 1 | 0.655 | 0.742 |
| seq.21286.29 | 60S ribosomal protein L11 | 0.495 | 0.285 |
| seq.21289.36 | Sperm-associated antigen 7 | 0.853 | 0.585 |
| seq.21290.66 | Checkpoint protein HUS1 | 0.000 | 0.000 |
| seq.21306.7 | HAUS augmin-like complex subunit 1 | 0.933 | 0.956 |
| seq.21308.44 | Dehydrogenase/reductase SDR family member 4 | 0.584 | 0.000 |
| seq.21310.37 | mRNA turnover protein 4 homolog | 0.219 | 0.536 |
| seq.21311.22 | Jun dimerization protein 2 | 0.807 | 0.421 |
| seq.21314.11 | Ubiquitin-like modifier-activating enzyme 5 | 0.584 | 0.253 |
| seq.21317.25 | NF-kappa-B inhibitor-interacting Ras-like protein 1 | 0.883 | 0.900 |
| seq.21319.196 | rRNA methyltransferase 1, mitochondrial | 0.752 | 0.597 |
| seq.21321.2 | U2 small nuclear ribonucleoprotein AAE | 0.905 | 0.572 |
| seq.21322.60 | Transcription initiation factor IIE subunit beta | 0.755 | 0.842 |
| seq.21323.2 | RWD domain-containing protein 1 | 0.000 | 0.000 |
| seq.21326.28 | Tubulin-specific chaperone C | 0.260 | 0.000 |
| seq.21327.12 | Modulator of retrovirus infection homolog | 0.886 | 0.375 |
| seq.21328.2 | Synaptotagmin-1 | 0.728 | 0.407 |
| seq.21329.66 | Pyridoxal phosphate phosphatase PHOSPHO2 | 0.166 | 0.000 |
| seq.21330.13 | Tyrosyl-tRNA synthetase, mitochondrial | 0.860 | 0.889 |
| seq.21331.19 | B-cell CLL/lymphoma 7 protein family member A | 0.682 | 0.565 |
| seq.21338.44 | Nucleotide exchange factor SIL1 | 0.835 | 0.809 |
| seq.21339.19 | Alanine aminotransferase 2 | 0.657 | 0.390 |
| seq.21340.38 | Hepatic leukemia factor | 0.780 | 0.660 |
| seq.21341.19 | Charged multivesicular body protein 4a | 0.284 | 0.000 |
| seq.21342.25 | Regulator of G-protein signaling 14 | 0.861 | 0.351 |
| seq.21343.3 | Activity-regulated cytoskeleton-associated protein | 0.310 | 0.471 |
| seq.21344.31 | Dimethyladenosine transferase 1, mitochondrial | 0.361 | 0.000 |
| seq.21345.93 | Retinol dehydrogenase 12 | 0.707 | 0.000 |
| seq.21346.71 | Dihydroorotate dehydrogenase (quinone), mitochondrial | 0.385 | 0.868 |
| seq.21348.22 | tRNA 2'-phosphotransferase 1 | 0.491 | 0.954 |
| seq.21349.2 | Serine/threonine/tyrosine-interacting protein | 0.479 | 0.650 |
| seq.21350.29 | Protein lin-28 homolog B | 0.708 | 0.206 |
| seq.21351.8 | Glycyl t-RNA synthetase | 0.019 | 0.000 |
| seq.21354.92 | Uracil phosphoribosyltransferase homolog | 0.689 | 0.865 |
| seq.21355.4 | Upstream stimulatory factor 1 | 0.213 | 0.738 |
| seq.21356.6 | B9 domain-containing protein 2 | 0.771 | 0.476 |
| seq.21357.12 | Filamin-binding LIM protein 1 | 0.224 | 0.724 |
| seq.21359.17 | Neuronal regeneration-related protein | 0.809 | 0.312 |
| seq.21360.27 | Sperm antigen with calponin homology and coiled-coil domains 1 | 0.000 | 0.616 |
| seq.21361.8 | Protein TXNRD3NB | 0.448 | 0.294 |
| seq.21365.5 | Cytohesin-3 | 0.316 | 0.238 |
| seq.21366.7 | RNA-binding protein Musashi homolog 2 | 0.092 | 0.000 |
| seq.21367.63 | Nuclear receptor-binding factor 2 | 0.904 | 0.509 |
| seq.21368.46 | DNA-directed RNA polymerase III subunit RPC10 | 0.889 | 0.723 |
| seq.21369.15 | RNA 3'-terminal phosphate cyclase | 0.299 | 0.162 |
| seq.21370.45 | Spindle and kinetochore-associated protein 1 | 0.851 | 0.758 |
| seq.21371.12 | Bifunctional lysine-specific demethylase and histidyl-hydroxylase MINA | 0.705 | 0.152 |
| seq.21373.8 | Queuine tRNA-ribosyltransferase subunit QTRTD1 | 0.339 | 0.000 |
| seq.21375.34 | Guanine nucleotide exchange factor for Rab-3A | 0.950 | 0.518 |
| seq.21378.7 | Polyribonucleotide nucleotidyltransferase 1, mitochondrial | 0.293 | 0.000 |
| seq.21379.30 | Ethanolamine kinase 2 | 0.012 | 0.758 |
| seq.21380.77 | Queuine tRNA-ribosyltransferase | 0.779 | 0.922 |
| seq.21381.73 | TBC1 domain family member 22B | 0.385 | 0.880 |
| seq.21382.70 | Zinc phosphodiesterase ELAC protein 1 | 0.900 | 0.606 |
| seq.21383.37 | Asparaginyl-tRNA synthetase, cytoplasmic | 0.000 | 0.000 |
| seq.21384.2 | Alpha-L-fucoside fucohydrolase | 0.876 | 0.566 |
| seq.21385.5 | SLIT and NTRK-like protein 2 | 0.761 | 0.213 |
| seq.21387.64 | Meprin A subunit alpha | 0.288 | 0.532 |
| seq.21389.32 | Serine/threonine-protein kinase 3 | 0.194 | 0.165 |
| seq.21390.68 | L-dopachrome tautomerase | 0.606 | 0.497 |
| seq.21391.17 | Choriogonadotropin subunit beta 3 | 0.747 | 0.380 |
| seq.21392.15 | Melanoma-associated antigen D1 | 0.802 | 0.976 |
| seq.21393.62 | Adenosylhomocysteinase | 0.776 | 0.802 |
| seq.21395.23 | Protocadherin-17 | 0.650 | 0.575 |
| seq.21397.21 | Protogenin | 0.576 | 0.608 |
| seq.21403.39 | Tuberin | 0.664 | 0.238 |
| seq.21406.25 | Mitogen-activated protein kinase kinase kinase 11 | 0.510 | 0.419 |
| seq.21415.212 | Mitogen-activated protein kinase kinase kinase 3 | 0.191 | 0.000 |
| seq.21426.88 | Ceramide-1-phosphate transfer protein | 0.109 | 0.000 |
| seq.21429.50 | Thimet oligopeptidase | 0.414 | 0.468 |
| seq.21430.4 | Cytosolic phospholipase A2 alpha | 0.087 | 0.000 |
| seq.21433.3 | Butyrophilin subfamily 3 member A2 | 0.927 | 0.841 |
| seq.21435.143 | Serine/threonine-protein kinase SRPK2 | 0.968 | 0.794 |
| seq.21436.56 | Cysteine protease ATG4A | 0.590 | 0.295 |
| seq.21437.77 | Methylated-DNA--protein-cysteine methyltransferase | 0.335 | 0.009 |
| seq.21438.45 | Tripartite motif-containing protein 5 | 0.798 | 0.871 |
| seq.21439.37 | Hematopoietic lineage cell-specific protein | 0.049 | 0.061 |
| seq.21440.9 | ADAM 8 | 0.325 | 0.967 |
| seq.21441.20 | gp75 | 0.815 | 0.535 |
| seq.21444.40 | Kallikrein 2 | 0.503 | 0.000 |
| seq.21445.40 | Ubiquitin-like protein Nedd8 | 0.000 | 0.000 |
| seq.21450.333 | Caspase-10:region 2 | 0.775 | 0.938 |
| seq.21452.3 | Dynamin-1-like protein | 0.703 | 0.755 |
| seq.21457.141 | Coenzyme Q-binding protein COQ10 homolog A, mitochondrial | 0.574 | 0.635 |
| seq.21464.2 | Baculoviral IAP repeat-containing protein 2 | 0.032 | 0.079 |
| seq.21475.137 | Thioredoxin-like protein 1 | 0.531 | 0.509 |
| seq.21476.43 | Endonuclease 8-like 2 | 0.659 | 0.672 |
| seq.21477.105 | Macrophage erythroblast attacher | 0.883 | 0.713 |
| seq.21478.20 | SMAD5 | 0.284 | 0.000 |
| seq.21480.2 | CD82 antigen | 0.951 | 0.935 |
| seq.21483.155 | cAMP-dependent protein kinase type I-alpha regulatory subunit | 0.656 | 0.818 |
| seq.21487.20 | Biogenesis of lysosome-related organelles complex 1 subunit 6 | 0.918 | 0.763 |
| seq.21491.7 | Vascular adhesion protein-1 | 0.748 | 0.342 |
| seq.21492.19 | Clusterin-like protein 1 | 0.723 | 0.536 |
| seq.21495.134 | Prosaposin receptor GPR37 | 0.843 | 0.704 |
| seq.21497.32 | Hypoxia up-regulated protein 1 | 0.410 | 0.630 |
| seq.21498.3 | Alpha-aminoadipic semialdehyde dehydrogenase | 0.532 | 0.783 |
| seq.21499.17 | 5-demethoxyubiquinone hydroxylase, mitochondrial | 0.417 | 0.784 |
| seq.21501.30 | Farnesyl pyrophosphate synthetase | 0.419 | 0.870 |
| seq.21503.12 | Thyrotropin receptor | 0.829 | 0.802 |
| seq.21504.41 | Eukaryotic translation initiation factor 2C 2 | 0.739 | 0.655 |
| seq.21507.48 | COMM domain-containing protein 8 | 0.288 | 0.355 |
| seq.21508.7 | Alpha-N-acetylneuraminide alpha-2,8-sialyltransferase | 0.756 | 0.275 |
| seq.21509.29 | Glycosyltransferase 8 domain-containing protein 2 | 0.543 | 0.608 |
| seq.21510.24 | G protein-coupled receptor kinase 5 | 0.676 | 0.421 |
| seq.21512.6 | ER degradation-enhancing alpha-mannosidase-like 2 | 0.935 | 0.948 |
| seq.21513.1 | Caspase-7 | 0.852 | 0.708 |
| seq.21514.1 | Platelet glycoprotein Ib beta chain | 0.878 | 0.830 |
| seq.21515.61 | Acetoacetyl-CoA synthetase | 0.832 | 0.701 |
| seq.21521.17 | Tryptophan 5-hydroxylase 1 | 0.846 | 0.629 |
| seq.21523.71 | Integrin-linked kinase-associated serine/threonine phosphatase 2C | 0.833 | 0.425 |
| seq.21524.14 | Ubiquitin carboxyl-terminal hydrolase 5 | 0.252 | 0.940 |
| seq.21526.88 | Izumo sperm-egg fusion protein 1 | 0.559 | 0.734 |
| seq.21528.12 | Bcl-2-modifying factor | 0.691 | 0.711 |
| seq.21529.6 | Ubiquitin-associated and SH3 domain-containing protein A | 0.072 | 0.815 |
| seq.21533.51 | Lymphocyte-specific protein 1 | 0.221 | 0.625 |
| seq.21535.5 | Alanyl-tRNA editing protein Aarsd1 | 0.359 | 0.230 |
| seq.21536.65 | Complexin-3 | 0.970 | 0.866 |
| seq.21537.33 | Selenocysteine lyase | 0.652 | 0.732 |
| seq.21539.139 | Synaptotagmin-6 | 0.822 | 0.372 |
| seq.21540.73 | Bridging integrator 2 | 0.043 | 0.000 |
| seq.21544.4 | Atlastin-3 | 0.711 | 0.891 |
| seq.21545.51 | Musculoskeletal embryonic nuclear protein 1 | 0.482 | 0.322 |
| seq.21546.20 | Adhesion G-protein coupled receptor D1 | 0.883 | 0.505 |
| seq.21547.6 | Dentin matrix protein 4 | 0.720 | 0.287 |
| seq.21548.20 | Gamma-glutamyltransferase 5 | 0.766 | 0.198 |
| seq.21549.144 | Beta-tectorin | 0.924 | 0.360 |
| seq.21552.9 | Peroxisomal acyl-coenzyme A oxidase 1 | 0.000 | 0.000 |
| seq.21555.17 | Protein FAM3A | 0.388 | 0.000 |
| seq.21562.3 | Secretoglobin family 1D member 1 | 0.421 | 0.774 |
| seq.21563.3 | sPLA(2)-XII | 0.362 | 0.000 |
| seq.21567.214 | Complement C1q tumor necrosis factor-related protein 4 | 0.614 | 0.723 |
| seq.21569.49 | Serine/threonine kinase 10 | 0.738 | 0.468 |
| seq.21572.91 | Xylosyltransferase 2 | 0.584 | 0.068 |
| seq.21574.106 | S100 calcium-binding protein A15 | 0.403 | 0.000 |
| seq.21577.35 | FK506-binding protein 5 | 0.123 | 0.000 |
| seq.21579.35 | Myosin-binding protein C, cardiac-type | 0.963 | 0.704 |
| seq.21581.87 | Cyclin-dependent kinase 15; EC=2.7.11.22 | 0.000 | 0.572 |
| seq.21583.14 | 60S ribosomal protein L26-like 1 | 0.366 | 0.603 |
| seq.21588.4 | BTB/POZ domain-containing protein KCTD4 | 0.567 | 0.186 |
| seq.21589.80 | Developmental pluripotency-associated protein 4 | 0.863 | 0.736 |
| seq.21590.9 | Ankyrin repeat family A protein 2 | 0.607 | 0.463 |
| seq.21592.8 | Cytosolic beta-glucosidase | 0.743 | 0.450 |
| seq.21595.8 | Coiled-coil domain-containing protein 43 | 0.718 | 0.714 |
| seq.21599.6 | Cystathionine gamma-lyase | 0.981 | 0.814 |
| seq.21600.10 | Beta-catenin-interacting protein 1 | 0.018 | 0.000 |
| seq.21604.2 | Protein CutA | 0.981 | 0.952 |
| seq.21614.2 | Gem-associated protein 6 | 0.612 | 0.009 |
| seq.21624.64 | NF-kappa-B inhibitor-interacting Ras-like protein 2 | 0.829 | 0.277 |
| seq.21628.6 | Eukaryotic translation elongation factor 1 epsilon-1 | 0.452 | 0.000 |
| seq.21636.63 | Nuclear cap-binding protein subunit 2 | 0.527 | 0.614 |
| seq.21638.5 | Peptidyl-prolyl cis-trans isomerase-like 3 | 0.979 | 0.913 |
| seq.21643.8 | 40S ribosomal protein S20 | 0.122 | 0.478 |
| seq.21645.6 | Smad nuclear-interacting protein 1 | 0.893 | 0.679 |
| seq.21647.9 | Spermidine synthase | 0.771 | 0.882 |
| seq.21649.12 | Syntaxin-17 | 0.771 | 0.159 |
| seq.21651.9 | Thioredoxin-like protein 4A | 0.712 | 0.538 |
| seq.21653.205 | Ubiquitin-related modifier 1 | 0.000 | 0.066 |
| seq.21655.18 | LisH domain-containing protein FOPNL | 0.495 | 0.367 |
| seq.21658.15 | Prohibitin-2 | 0.709 | 0.878 |
| seq.21660.4 | GTP-binding protein SAR1b | 0.373 | 0.454 |
| seq.21661.82 | Dentin matrix protein 4 | 0.530 | 0.121 |
| seq.21662.121 | Dual specificity tyrosine-phosphorylation-regulated kinase 3 | 0.000 | 0.000 |
| seq.21663.149 | Alpha-N-acetylneuraminide alpha-2,8-sialyltransferase | 0.300 | 0.369 |
| seq.21664.6 | Core histone macro-H2A.1 | 0.880 | 0.703 |
| seq.21667.57 | Calcium/calmodulin-dependent 3',5'-cyclic nucleotide phosphodiesterase 1B | 0.758 | 0.670 |
| seq.21670.52 | NEDD4-like E3 ubiquitin-protein ligase WWP2 | 0.478 | 0.912 |
| seq.21674.132 | Butyrophilin subfamily 1 member A1 | 0.903 | 0.802 |
| seq.21676.17 | PAI-2 | 0.548 | 0.174 |
| seq.21679.16 | Cadherin-20 | 0.616 | 0.493 |
| seq.21681.10 | ADAM 28 | 0.439 | 0.254 |
| seq.21685.29 | DCC | 0.761 | 0.601 |
| seq.21687.3 | Protein phosphatase 1 regulatory subunit 29 | 0.848 | 0.398 |
| seq.21688.50 | Very Late Antigen-4 | 0.887 | 0.710 |
| seq.21690.31 | Leucine-rich repeat-containing protein 4 | 0.443 | 0.341 |
| seq.21691.27 | Leucine-rich repeat and fibronectin type-III domain-containing protein 3 | 0.197 | 0.878 |
| seq.21692.12 | CD8A/CD8B Complex | 0.555 | 0.227 |
| seq.21693.14 | Cadherin-13 | 0.443 | 0.075 |
| seq.21696.80 | Leucine-rich repeat and fibronectin type-III domain-containing protein 4 | 0.961 | 0.404 |
| seq.21697.57 | Adhesion G-protein coupled receptor F2 | 0.861 | 0.903 |
| seq.21698.11 | Integrin a11b1 | 0.814 | 0.690 |
| seq.21703.31 | Activin RIA | 0.392 | 0.393 |
| seq.21704.8 | Protocadherin-12 | 0.842 | 0.904 |
| seq.21705.33 | Meteorin-like protein | 0.652 | 0.326 |
| seq.21706.29 | Leukocyte immunoglobulin-like receptor subfamily A member 1 | 0.754 | 0.817 |
| seq.21707.15 | Complement C1q-like protein 3 | 0.597 | 0.455 |
| seq.21708.149 | IgG receptor FcRn large subunit p51 | 0.778 | 0.707 |
| seq.21710.16 | Neuroligin-4, Y-linked | 0.601 | 0.051 |
| seq.21711.86 | Brain-specific angiogenesis inhibitor 1 | 0.707 | 0.235 |
| seq.21713.11 | Reelin | 0.846 | 0.314 |
| seq.21715.40 | Sentrin-specific protease 1 | 0.899 | 0.583 |
| seq.21716.29 | Alpha-(1,3)-fucosyltransferase | 0.736 | 0.021 |
| seq.21717.44 | Ubiquitin-conjugating enzyme E2 Q2 | 0.274 | 0.000 |
| seq.21718.150 | Hyaluronidase-4 | 0.744 | 0.000 |
| seq.21720.13 | Alpha-(1,3)-fucosyltransferase 11 | 0.955 | 0.759 |
| seq.21721.6 | Carbohydrate sulfotransferase 10 | 0.326 | 0.023 |
| seq.21722.21 | Polypeptide N-acetylgalactosaminyltransferase 4 | 0.743 | 0.523 |
| seq.21724.22 | N-acetylgalactosamine-6-sulfatase | 0.808 | 0.723 |
| seq.21726.73 | Alpha-N-acetyl-neuraminyl-2,3-beta-galactosyl-1,3-N-acetyl-galactosaminide alpha-2,6-sialyltransferase | 0.816 | 0.648 |
| seq.21727.15 | Ubiquitin-conjugating enzyme E2 Q1 | 0.369 | 0.000 |
| seq.21728.4 | Ubiquitin carboxyl-terminal hydrolase 2 | 0.961 | 0.828 |
| seq.21730.56 | Ubiquitin carboxyl-terminal hydrolase 19 | 0.564 | 0.225 |
| seq.21732.78 | Ubiquitin-conjugating enzyme E2 Q1 | 0.944 | 0.360 |
| seq.21733.11 | UDP-N-acetylglucosamine--peptide N-acetylglucosaminyltransferase 110 kDa subunit | 0.929 | 0.565 |
| seq.21734.36 | C1GLT/C1GLC Complex | 0.399 | 0.372 |
| seq.21736.60 | Heparan sulfate 2-O-sulfotransferase 1 | 0.892 | 0.501 |
| seq.21737.20 | Beta-galactoside alpha-2,6-sialyltransferase 2 | 0.891 | 0.645 |
| seq.21739.7 | Bifunctional UDP-N-acetylglucosamine 2-epimerase/N-acetylmannosamine kinase | 0.602 | 0.530 |
| seq.21740.26 | Ubiquitin carboxyl-terminal hydrolase 1 | 0.875 | 0.603 |
| seq.21742.43 | OTU domain-containing protein 7B | 0.409 | 0.000 |
| seq.21743.1 | WD repeat-containing protein 48 | 0.183 | 0.000 |
| seq.21746.13 | Ubiquitin carboxyl-terminal hydrolase 10 | 0.428 | 0.451 |
| seq.21747.114 | UBE2N (Ubc13)/Uev1a Complex | 0.155 | 0.182 |
| seq.21747.8 | UBE2N (Ubc13)/Uev1a Complex | 0.108 | 0.000 |
| seq.21748.36 | UBP46/WDR48 Complex | 0.225 | 0.000 |
| seq.21750.25 | Ubiquitin carboxyl-terminal hydrolase BAP1 | 0.942 | 0.836 |
| seq.21751.6 | USP12/WDR48 Complex | 0.581 | 0.457 |
| seq.21752.10 | Mitochondrial Rho GTPase 1 | 0.072 | 0.000 |
| seq.21754.5 | Ubiquitin thioesterase ZRANB1 | 0.383 | 0.000 |
| seq.21755.27 | Ubiquitin carboxyl-terminal hydrolase 12 | 0.745 | 0.872 |
| seq.21756.5 | UBE2N/UBE2V2 Complex | 0.116 | 0.000 |
| seq.21757.49 | Ubiquitin carboxyl-terminal hydrolase 22 | 0.565 | 0.000 |
| seq.21758.65 | Sentrin-specific protease 2 | 0.901 | 0.855 |
| seq.21759.23 | UBP1/WDR48 Complex | 0.739 | 0.287 |
| seq.21760.22 | Ubiquitin-activating enzyme E1 | 0.109 | 0.000 |
| seq.21761.213 | Ubiquitin carboxyl-terminal hydrolase 46 | 0.516 | 0.592 |
| seq.21762.32 | Ubiquitin carboxyl-terminal hydrolase 11 | 0.608 | 0.110 |
| seq.21763.46 | Ubiquitin carboxyl-terminal hydrolase 4 | 0.085 | 0.067 |
| seq.21764.99 | Ubiquitin carboxyl-terminal hydrolase 30 | 0.851 | 0.780 |
| seq.21765.10 | Ubiquitin carboxyl-terminal hydrolase CYLD | 0.221 | 0.000 |
| seq.21766.50 | N-sulphoglucosamine sulphohydrolase | 0.839 | 0.888 |
| seq.21767.129 | Polypeptide N-acetylgalactosaminyltransferase 14 | 0.729 | 0.508 |
| seq.21768.9 | Alpha-1,6-mannosylglycoprotein 6-beta-N-acetylglucosaminyltransferase A | 0.796 | 0.301 |
| seq.21769.10 | Xylulose kinase | 0.933 | 0.479 |
| seq.21770.18 | Galactosylgalactosylxylosylprotein 3-beta-glucuronosyltransferase 1 | 0.848 | 0.833 |
| seq.21771.47 | Beta-1,4-mannosyl-glycoprotein 4-beta-N-acetylglucosaminyltransferase | 0.754 | 0.746 |
| seq.21780.15 | Protein ABHD4 | 0.683 | 0.540 |
| seq.21781.9 | Aspartoacylase-2 | 0.717 | 0.852 |
| seq.21786.25 | Cyclin-dependent kinase-like 2 | 0.070 | 0.000 |
| seq.21793.4 | FGFR1 oncogene partner | 0.217 | 0.370 |
| seq.21796.43 | Glutathione peroxidase 3 | 0.471 | 0.279 |
| seq.21797.4 | HLA-C | 0.975 | 0.948 |
| seq.21799.15 | Homer protein homolog 3 | 0.490 | 0.491 |
| seq.21802.53 | Endonuclease 8-like 1 | 0.000 | 0.232 |
| seq.21804.15 | Putative KHDC1-like protein | 0.262 | 0.568 |
| seq.21810.50 | MART-1 | 0.510 | 0.233 |
| seq.21811.20 | Nucleolar protein of 40 kDa | 0.813 | 0.559 |
| seq.21813.171 | Alpha-1,6-mannosylglycoprotein 6-beta-N-acetylglucosaminyltransferase A | 0.721 | 0.182 |
| seq.21814.13 | Methylmalonic aciduria and homocystinuria type D protein, mitochondrial | 0.726 | 0.633 |
| seq.21815.7 | MOB kinase activator 3B | 0.000 | 0.609 |
| seq.21817.5 | Myosin, light chain 9, regulatory | 0.117 | 0.000 |
| seq.21819.9 | Negative elongation factor E | 0.913 | 0.940 |
| seq.2182.54 | Complement C4b | 0.892 | 0.360 |
| seq.21821.9 | Peptidyl-prolyl cis-trans isomerase A-like 4D | 0.613 | 0.568 |
| seq.21823.5 | Protein phosphatase 1G | 0.319 | 0.399 |
| seq.21827.7 | Rho-related GTP-binding protein RhoQ | 0.061 | 0.000 |
| seq.21828.6 | Ribophorin II | 0.368 | 0.473 |
| seq.21829.8 | 40S ribosomal protein S12 | 0.291 | 0.391 |
| seq.21832.31 | Squamous cell carcinoma antigen 1 | 0.452 | 0.225 |
| seq.21833.6 | silent mating type information regulation 2 homolog | 0.148 | 0.718 |
| seq.21834.8 | Vacuolar-sorting protein SNF8 | 0.372 | 0.175 |
| seq.21836.9 | Somatostatin receptor type 1 | 0.839 | 0.739 |
| seq.21837.3 | Translocon-associated protein subunit beta | 0.071 | 0.875 |
| seq.21839.3 | POU domain class 2-associating factor 1 | 0.475 | 0.000 |
| seq.21848.18 | Ubiquitin-conjugating enzyme E2 R2 | 0.420 | 0.549 |
| seq.21849.2 | Ubiquilin-3 | 0.000 | 0.000 |
| seq.21853.3 | CLEC-1 | 0.572 | 0.640 |
| seq.21856.59 | Ketohexokinase | 0.329 | 0.612 |
| seq.21857.26 | Guanine nucleotide-binding protein subunit beta-2-like 1 | 0.127 | 0.499 |
| seq.21858.25 | E3 ubiquitin-protein ligase SIAH1 | 0.815 | 0.936 |
| seq.21861.8 | Proteasome subunit beta type-10 | 0.579 | 0.404 |
| seq.21862.145 | LIM and SH3 domain protein 1 | 0.027 | 0.000 |
| seq.21863.11 | Cytosolic Fe-S cluster assembly factor NUBP2 | 0.616 | 0.813 |
| seq.21864.20 | Protein MAK16 homolog | 0.196 | 0.305 |
| seq.21875.31 | AN1-type zinc finger protein 3 | 0.920 | 0.906 |
| seq.21876.266 | PRKCA-binding protein | 0.921 | 0.185 |
| seq.21883.17 | Bone morphogenetic protein 5 | 0.613 | 0.214 |
| seq.21885.196 | Nucleolysin TIA-1 isoform p40 | 0.000 | 0.000 |
| seq.21887.2 | Cerebellin-2 | 0.875 | 0.164 |
| seq.21889.2 | Uncharacterized protein C7orf24 | 0.931 | 0.152 |
| seq.21891.31 | Fibulin-7 | 0.748 | 0.615 |
| seq.21895.36 | Interferon lambda-4 | 0.097 | 0.687 |
| seq.21897.4 | IL-17/IL-17F | 0.364 | 0.409 |
| seq.21899.36 | inhibin A | 0.952 | 0.739 |
| seq.2190.55 | Coagulation Factor XI | 0.519 | 0.676 |
| seq.21901.14 | Integrin a3b1 | 0.337 | 0.344 |
| seq.21903.6 | Integrin alpha L beta 2 | 0.591 | 0.721 |
| seq.21905.10 | Integrin alpha-2/b1 | 0.708 | 0.880 |
| seq.21909.10 | Integrin a5b1 | 0.077 | 0.579 |
| seq.21909.2 | Integrin a5b1 | 0.133 | 0.000 |
| seq.21911.17 | Norrin | 0.579 | 0.723 |
| seq.21913.5 | Transcription factor YY2 | 0.149 | 0.837 |
| seq.21915.2 | OTU domain-containing protein 3 | 0.823 | 0.329 |
| seq.21916.82 | Ubiquitin thioesterase otulin | 0.000 | 0.493 |
| seq.2192.63 | C-C motif chemokine 27 | 0.931 | 0.264 |
| seq.21923.24 | SAE1/SAE2 | 0.000 | 0.310 |
| seq.21925.15 | Transforming growth factor beta-1 | 0.478 | 0.000 |
| seq.21926.24 | UB2D1/PolyUbiquitin K48 | 0.000 | 0.067 |
| seq.21929.53 | UB2D3/PolyUbiquitin K48 | 0.013 | 0.000 |
| seq.21931.16 | UB2L3/PolyUbiquitin K48 | 0.514 | 0.599 |
| seq.21931.27 | UB2L3/PolyUbiquitin K48 | 0.131 | 0.083 |
| seq.21933.7 | Ubiquitin-like modifier-activating enzyme 7 | 0.010 | 0.000 |
| seq.21935.16 | Ubiquitin-conjugating enzyme E2 H | 0.575 | 0.079 |
| seq.21939.6 | ULA1/UBA3 | 0.147 | 0.000 |
| seq.21940.12 | Protein Wnt-10b | 0.884 | 0.858 |
| seq.21942.14 | Protein Wnt-16 | 0.384 | 0.916 |
| seq.21943.170 | Protein Wnt-16 | 0.341 | 0.032 |
| seq.21945.4 | Protein Wnt-5b | 0.778 | 0.524 |
| seq.21946.79 | IL-6/IL-6 sRa Complex | 0.704 | 0.538 |
| seq.21949.4 | Amyloid beta A4 precursor protein-binding family B member 1-interacting protein | 0.239 | 0.649 |
| seq.21951.32 | Aryl hydrocarbon receptor nuclear translocator | 0.397 | 0.680 |
| seq.21955.36 | Large proline-rich protein BAT3 | 0.005 | 0.063 |
| seq.21957.12 | Phosphoinositide 3-kinase adapter protein 1 | 0.536 | 0.541 |
| seq.21958.4 | Active breakpoint cluster region-related protein | 0.097 | 0.000 |
| seq.21961.14 | CD2-associated protein | 0.197 | 0.000 |
| seq.21963.48 | Disabled homolog 2 | 0.032 | 0.000 |
| seq.21967.20 | Corticosteroid 11-beta-dehydrogenase isozyme 1 | 0.606 | 0.455 |
| seq.21969.5 | Dipeptidyl aminopeptidase-like protein 6 | 0.766 | 0.868 |
| seq.21971.47 | Friend leukemia integration 1 transcription factor | 0.000 | 0.000 |
| seq.21975.22 | Islet cell autoantigen 1 | 0.335 | 0.012 |
| seq.21976.4 | I-kappa-B kinase gamma | 0.435 | 0.184 |
| seq.21979.12 | Integrin alpha-6 | 0.648 | 0.725 |
| seq.21979.8 | Integrin alpha-6 | 0.599 | 0.000 |
| seq.21981.2 | Integrin alpha-M | 0.220 | 0.778 |
| seq.21985.61 | Integrin beta-1-binding protein 2 | 0.227 | 0.000 |
| seq.21987.76 | Lipoprotein lipase | 0.485 | 0.444 |
| seq.21989.12 | Mitogen-activated protein kinase kinase kinase kinase 5 | 0.049 | 0.000 |
| seq.21991.79 | Neurabin-2 | 0.023 | 0.000 |
| seq.21995.20 | NOS | 0.035 | 0.373 |
| seq.21996.28 | NEDD8 ultimate buster 1 | 0.779 | 0.067 |
| seq.21999.61 | Nucleobindin-2 | 0.185 | 0.103 |
| seq.22001.23 | Protein-arginine deiminase type-2 | 0.576 | 0.956 |
| seq.22003.4 | Paraoxonase 2 | 0.346 | 0.065 |
| seq.22005.8 | [Pyruvate dehydrogenase [acetyl-transferring]]-phosphatase 1, mitochondrial | 0.736 | 0.935 |
| seq.22007.1 | Resistin-like beta | 0.556 | 0.560 |
| seq.22009.1 | Protein phosphatase 1B | 0.120 | 0.403 |
| seq.2201.17 | Endostatin | 0.721 | 0.327 |
| seq.22010.36 | Interferon-inducible double-stranded RNA-dependent protein kinase activator A | 0.440 | 0.850 |
| seq.22013.6 | Ras association domain-containing protein 2 | 0.151 | 0.490 |
| seq.22015.2 | E3 ubiquitin-protein ligase TRIM21 | 0.785 | 0.341 |
| seq.22019.21 | SH2B adapter protein 3 | 0.919 | 0.784 |
| seq.22023.3 | Kynurenine/alpha-aminoadipate aminotransferase, mitochondrial | 0.009 | 0.210 |
| seq.22027.13 | Transforming acidic coiled-coil-containing protein 3 | 0.649 | 0.593 |
| seq.22033.32 | Wiskott-Aldrich syndrome protein | 0.066 | 0.000 |
| seq.22033.7 | Wiskott-Aldrich syndrome protein | 0.379 | 0.546 |
| seq.22034.61 | Zinc finger and BTB domain-containing protein 16 | 0.931 | 0.243 |
| seq.22037.47 | EF-hand domain-containing protein D2 | 0.503 | 0.260 |
| seq.22041.26 | Dual specificity mitogen-activated protein kinase kinase 5 | 0.037 | 0.177 |
| seq.22043.174 | Heat shock-related 70 kDa protein 2 | 0.389 | 0.317 |
| seq.22045.8 | Small ubiquitin-related modifier 4 | 0.302 | 0.000 |
| seq.22047.46 | Collagen alpha-1(V) chain | 0.508 | 0.485 |
| seq.22049.24 | 14-3-3 protein eta | 0.000 | 0.000 |
| seq.22050.19 | KxDL motif-containing protein 1 | 0.337 | 0.000 |
| seq.22055.31 | Forkhead box protein O1A | 0.787 | 0.881 |
| seq.22057.9 | Aldehyde dehydrogenase X, mitochondrial | 0.000 | 0.377 |
| seq.22059.56 | Palmdelphin | 0.571 | 0.757 |
| seq.22063.20 | Calpain small subunit 2 | 0.467 | 0.812 |
| seq.22065.32 | Interferon regulatory factor 8 | 0.000 | 0.647 |
| seq.22068.5 | Serine/threonine-protein phosphatase 2A 56 kDa regulatory subunit delta isoform | 0.504 | 0.148 |
| seq.22070.5 | Rho guanine nucleotide exchange factor 16 | 0.000 | 0.621 |
| seq.22071.38 | Ankyrin repeat and SOCS box protein 8 | 0.654 | 0.228 |
| seq.22073.47 | ATP synthase B chain, mitochondrial | 0.559 | 0.901 |
| seq.22074.35 | Ataxin-2-binding protein 1 | 0.922 | 0.104 |
| seq.22075.16 | Cyclic AMP-dependent transcription factor ATF-3 | 0.686 | 0.924 |
| seq.22076.34 | Protein atonal homolog 1 | 0.392 | 0.574 |
| seq.22077.46 | Mitochondrial inner membrane protease ATP23 homolog | 0.877 | 0.698 |
| seq.22078.4 | Tyrosine aminotransferase | 0.272 | 0.371 |
| seq.22080.22 | BTB/POZ domain-containing adapter for CUL3-mediated RhoA degradation protein 1 | 0.000 | 0.335 |
| seq.22082.25 | Protein BEX2 | 0.971 | 0.975 |
| seq.22083.60 | Protein BEX5 | 0.658 | 0.878 |
| seq.22085.86 | Polycomb complex protein BMI-1 | 0.922 | 0.578 |
| seq.22086.2 | Breast cancer metastasis-suppressor 1-like protein | 0.921 | 0.142 |
| seq.22088.3 | Protein BTG1 | 0.360 | 0.519 |
| seq.22089.13 | Protein BTG4 | 0.635 | 0.791 |
| seq.22091.14 | Beta-ureidopropionase | 0.107 | 0.182 |
| seq.22092.43 | Caspase-4 | 0.715 | 0.188 |
| seq.22094.15 | Uncharacterized protein C2orf73 | 0.833 | 0.596 |
| seq.22095.29 | Chromobox protein homolog 2 | 0.405 | 0.378 |
| seq.22098.10 | Cyclin-Y-like protein 1 | 0.688 | 0.736 |
| seq.22099.1 | Uncharacterized protein C4orf36 | 0.931 | 0.628 |
| seq.22103.25 | Cyclin-dependent kinase 20 | 0.844 | 0.904 |
| seq.22104.39 | Homeobox protein CDX-1 | 0.791 | 0.726 |
| seq.2211.9 | Metalloproteinase inhibitor 1 | 0.576 | 0.483 |
| seq.22112.30 | 2-methoxy-6-polyprenyl-1,4-benzoquinol methylase, mitochondrial | 0.000 | 0.027 |
| seq.22113.2 | Ubiquinone biosynthesis monooxygenase COQ6, mitochondrial | 0.857 | 0.511 |
| seq.22114.4 | Uncharacterized protein C18orf54 | 0.930 | 0.197 |
| seq.22115.2 | cAMP-responsive element-binding protein-like 2 | 0.743 | 0.538 |
| seq.22116.9 | cAMP-responsive element modulator | 0.875 | 0.258 |
| seq.22118.7 | Chondrosarcoma-associated gene 2/3 protein | 0.638 | 0.878 |
| seq.22119.18 | COP9 signalosome complex subunit 8 | 0.220 | 0.120 |
| seq.2212.69 | Tissue-type plasminogen activator | 0.501 | 0.671 |
| seq.22120.4 | DDB1- and CUL4-associated factor 11 | 0.653 | 0.900 |
| seq.22121.43 | DDB1- and CUL4-associated factor 12 | 0.436 | 0.957 |
| seq.22123.15 | Homeobox protein DLX-2 | 0.924 | 0.956 |
| seq.22124.94 | Dystrotelin | 0.712 | 0.525 |
| seq.22125.9 | E2F5 | 0.671 | 0.861 |
| seq.22128.8 | ETS domain-containing protein Elk-4 | 0.836 | 0.761 |
| seq.22129.55 | RNA polymerase II elongation factor ELL3 | 0.970 | 0.974 |
| seq.22134.1 | ETS translocation variant 4 | 0.442 | 0.225 |
| seq.22136.9 | Nuclease EXOG, mitochondrial | 0.767 | 0.507 |
| seq.22137.3 | Protein FAM102B | 0.631 | 0.616 |
| seq.22140.6 | Protein FAM204A | 0.690 | 0.583 |
| seq.22141.59 | 6-phosphofructo-2-kinase/fructose-2,6-bisphosphatase 1 | 0.603 | 0.955 |
| seq.22142.4 | Putative protein N-methyltransferase FAM86B1 | 0.362 | 0.915 |
| seq.22143.6 | F-box only protein 28 | 0.702 | 0.000 |
| seq.22144.65 | F-box only protein 48 | 0.078 | 0.914 |
| seq.22146.20 | Fos-related antigen 2 | 0.461 | 0.695 |
| seq.22147.19 | Forkhead box protein O4 | 0.772 | 0.622 |
| seq.22148.135 | Forkhead box protein P3 | 0.049 | 0.692 |
| seq.22149.2 | FUN14 domain-containing protein 1 | 0.818 | 0.562 |
| seq.22150.2 | GA-binding protein subunit beta-1 | 0.694 | 0.180 |
| seq.22151.48 | Homeobox protein goosecoid-2 | 0.961 | 0.526 |
| seq.22152.162 | Polypeptide N-acetylgalactosaminyltransferase 9 | 0.768 | 0.791 |
| seq.22154.37 | GATA zinc finger domain-containing protein 1 | 0.603 | 0.461 |
| seq.22155.44 | Gem-associated protein 7 | 0.531 | 0.469 |
| seq.22363.31 | tRNA-specific adenosine deaminase 1 | 0.935 | 0.636 |
| seq.22365.52 | Cyclic AMP-dependent transcription factor ATF-5 | 0.446 | 0.834 |
| seq.22367.17 | UPF0705 protein C11orf49 | 0.735 | 0.591 |
| seq.22369.12 | Doublesex- and mab-3-related transcription factor C2 | 0.774 | 0.335 |
| seq.22371.46 | Protein FAM110A | 0.098 | 0.112 |
| seq.22374.56 | Hairy/enhancer-of-split related with YRPW motif protein 1 | 0.460 | 0.942 |
| seq.22375.15 | Homeobox protein Hox-A11 | 0.714 | 0.969 |
| seq.22376.95 | Homeobox protein Hox-A5 | 0.911 | 0.516 |
| seq.22377.27 | Immediate early response gene 2 protein | 0.687 | 0.454 |
| seq.22378.2 | Keratin 34 | 0.745 | 0.031 |
| seq.22381.1 | Endoribonuclease LACTB2 | 0.000 | 0.000 |
| seq.22383.21 | MITF | 0.272 | 0.562 |
| seq.22385.2 | PWWP domain-containing protein MUM1 | 0.910 | 0.879 |
| seq.22386.11 | Homeobox protein OTX1 | 0.597 | 0.950 |
| seq.22387.26 | Pituitary homeobox 3 | 0.930 | 0.648 |
| seq.22391.34 | Histone deacetylase complex subunit SAP30L | 0.903 | 0.832 |
| seq.22392.5 | SERTA domain-containing protein 3 | 0.948 | 0.644 |
| seq.22393.130 | Thyroid transcription factor 1-associated protein 26 | 0.456 | 0.054 |
| seq.22394.8 | Transcription factor 21 | 0.741 | 0.790 |
| seq.22395.7 | Tristetraproline | 0.837 | 0.866 |
| seq.22397.2 | X-box-binding protein 1 | 0.859 | 0.315 |
| seq.22398.5 | Acidic leucine-rich nuclear phosphoprotein 32 family member C | 0.987 | 0.963 |
| seq.22401.26 | Guanylyl cyclase-activating protein 2 | 0.787 | 0.932 |
| seq.22402.12 | Histone H2A type 1-A | 0.639 | 0.628 |
| seq.22403.13 | Histone H2B type 1-K | 0.600 | 0.654 |
| seq.22404.4 | Inositol hexakisphosphate kinase 1 | 0.294 | 0.019 |
| seq.22405.61 | Phosphoenolpyruvate carboxykinase [GTP], mitochondrial | 0.200 | 0.381 |
| seq.22414.127 | Serine/threonine-protein kinase tousled-like 1 | 0.973 | 0.574 |
| seq.22417.10 | E3 ubiquitin-protein ligase CBL-C | 0.927 | 0.229 |
| seq.22421.79 | E3 ubiquitin-protein ligase pellino homolog 1 | 0.770 | 0.108 |
| seq.22429.13 | Lysine-specific histone demethylase 1A | 0.426 | 0.397 |
| seq.22430.15 | N-chimaerin | 0.905 | 0.128 |
| seq.22431.164 | Serum response factor-binding protein 1 | 0.086 | 0.203 |
| seq.22432.83 | Serine/threonine-protein kinase SRPK1 | 0.197 | 0.080 |
| seq.22445.2 | Calcium-binding protein 5 | 0.989 | 0.209 |
| seq.22465.43 | Transcription factor HES-1 | 0.667 | 0.452 |
| seq.22466.27 | Transcription factor HES-5 | 0.437 | 0.581 |
| seq.22467.94 | HERV-H LTR-associating protein 3 | 0.912 | 0.502 |
| seq.22468.54 | Histone H2A type 1 | 0.743 | 0.693 |
| seq.22469.103 | Homeobox protein HMX2 | 0.739 | 0.697 |
| seq.2247.20 | Prokineticin-1 | 0.976 | 0.293 |
| seq.22472.4 | Transcription factor 12 | 0.594 | 0.224 |
| seq.22474.28 | Homeobox protein Hox-C11 | 0.561 | 0.547 |
| seq.22476.115 | Homeobox protein Hox-D4 | 0.141 | 0.876 |
| seq.22479.64 | Inositol hexakisphosphate kinase 2 | 0.393 | 0.824 |
| seq.22481.15 | IQ domain-containing protein D | 0.149 | 0.273 |
| seq.22482.87 | Keratin, type I cytoskeletal 14 | 0.866 | 0.652 |
| seq.22483.109 | Keratin, type II cytoskeletal 71 | 0.581 | 0.438 |
| seq.22484.17 | Casein kinase I isoform alpha-like | 0.210 | 0.873 |
| seq.22485.1 | BTB/POZ domain-containing protein KCTD7 | 0.899 | 0.930 |
| seq.22486.58 | Keratin, type II cytoskeletal 6A | 0.876 | 0.906 |
| seq.22488.17 | Krueppel-like factor 9 | 0.589 | 0.544 |
| seq.22490.16 | Lipocalin-like 1 protein | 0.882 | 0.437 |
| seq.22491.10 | LIM domain-binding protein 2 | 0.859 | 0.645 |
| seq.22492.108 | LIM domain only protein 3 | 0.377 | 0.455 |
| seq.22496.21 | LYR motif-containing protein 1 | 0.971 | 0.789 |
| seq.22499.15 | Mediator of RNA polymerase II transcription subunit 28 | 0.548 | 0.574 |
| seq.22500.9 | Mediator of RNA polymerase II transcription subunit 11 | 0.738 | 0.381 |
| seq.22503.24 | Homeobox protein Mohawk | 0.033 | 0.257 |
| seq.22504.3 | NEDD4-binding protein 2-like 2 | 0.539 | 0.898 |
| seq.22505.24 | NAD kinase 2, mitochondrial | 0.835 | 0.336 |
| seq.22506.25 | N-acetyltransferase 5 | 0.320 | 0.493 |
| seq.22508.2 | NADH dehydrogenase | 0.735 | 0.956 |
| seq.22509.9 | Arginine-hydroxylase NDUFAF5, mitochondrial | 0.306 | 0.239 |
| seq.22510.6 | Nuclear factor erythroid 2-related factor 2 | 0.271 | 0.082 |
| seq.22511.28 | Nuclear transcription factor Y subunit alpha | 0.744 | 0.000 |
| seq.22512.18 | Neuroguidin | 0.303 | 0.873 |
| seq.22514.12 | Protein naked cuticle homolog 2 | 0.820 | 0.909 |
| seq.22516.30 | Neural retina-specific leucine zipper protein | 0.485 | 0.549 |
| seq.22517.106 | E3 SUMO-protein ligase NSE2 | 0.000 | 0.170 |
| seq.22518.54 | Kinetochore-associated protein NSL1 homolog | 0.517 | 0.456 |
| seq.22521.13 | Homeobox protein OTX2 | 0.608 | 0.903 |
| seq.22523.4 | Polyadenylate-binding protein 5 | 0.336 | 0.289 |
| seq.22525.9 | Paired box protein Pax-3 | 0.726 | 0.405 |
| seq.22526.81 | Pyruvate dehydrogenase E1 component beta subunit | 0.000 | 0.679 |
| seq.22527.4 | PDZ and LIM domain protein 3 | 0.595 | 0.302 |
| seq.22528.1 | PHD finger protein 11 | 0.765 | 0.403 |
| seq.22529.31 | PHD finger protein 6 | 0.510 | 0.625 |
| seq.22530.111 | Phytanoyl-CoA dioxygenase domain-containing protein 1 | 0.864 | 0.736 |
| seq.22531.44 | Pituitary homeobox 2 | 0.302 | 0.433 |
| seq.22532.59 | PLAC8-like protein 1 | 0.307 | 0.250 |
| seq.22533.95 | Serine/threonine-protein phosphatase 1 regulatory subunit 10 | 0.926 | 0.630 |
| seq.22534.14 | Serine/threonine-protein phosphatase 4 catalytic subunit | 0.692 | 0.771 |
| seq.22536.1 | Protein phosphatase 1 regulatory subunit 42 | 0.738 | 0.783 |
| seq.22537.5 | Protein phosphatase PTC7 homolog | 0.624 | 0.583 |
| seq.22540.129 | Proteasome subunit beta type 7 | 0.116 | 0.820 |
| seq.22541.46 | DNA replication complex GINS protein PSF1 | 0.958 | 0.832 |
| seq.22544.10 | DNA repair protein RAD51 homolog 3 | 0.091 | 0.541 |
| seq.22545.20 | RNA-binding Raly-like protein | 0.467 | 0.630 |
| seq.22547.17 | RNA-binding motif, single-stranded-interacting protein 1 | 0.231 | 0.521 |
| seq.22548.18 | Regulator of G-protein signaling 13 | 0.531 | 0.461 |
| seq.22549.73 | RIP | 0.596 | 0.372 |
| seq.22553.4 | 60S ribosomal protein L38 | 0.886 | 0.928 |
| seq.22554.101 | 39S ribosomal protein L10, mitochondrial | 0.329 | 0.000 |
| seq.22557.68 | 39S ribosomal protein L50, mitochondrial | 0.923 | 0.614 |
| seq.22560.1 | Protein EVI2A | 0.869 | 0.742 |
| seq.22561.3 | Uncharacterized protein UNQ511/PRO1026 | 0.572 | 0.568 |
| seq.22563.4 | Kin of IRRE-like protein 1 | 0.301 | 0.218 |
| seq.22564.5 | Low-density lipoprotein receptor class A domain-containing protein 3 | 0.671 | 0.946 |
| seq.22566.53 | Pituitary adenylate cyclase-activating polypeptide type I receptor | 0.391 | 0.735 |
| seq.22568.7 | Desmoglein-4 | 0.912 | 0.702 |
| seq.22569.55 | TGF-beta receptor type-1 | 0.303 | 0.198 |
| seq.22572.19 | Pepsin A-4 | 0.928 | 0.841 |
| seq.22576.1 | CD209 antigen | 0.777 | 0.859 |
| seq.22576.2 | CD209 antigen | 0.728 | 0.917 |
| seq.22577.24 | Sialic acid-binding Ig-like lectin 14 | 0.815 | 0.870 |
| seq.22578.17 | Roundabout homolog 2 | 0.616 | 0.190 |
| seq.22579.93 | Neurexin-1-beta | 0.870 | 0.173 |
| seq.22580.29 | Ephrin-A2 | 0.818 | 0.713 |
| seq.22583.47 | NKG2D ligand 2 | 0.646 | 0.345 |
| seq.22584.2 | Low-density lipoprotein receptor-related protein 6 | 0.801 | 0.590 |
| seq.22585.5 | Frizzled-4 | 0.892 | 0.944 |
| seq.22586.24 | Butyrophilin subfamily 2 member A2 | 0.839 | 0.852 |
| seq.22587.37 | Ephrin type-A receptor 6 | 0.580 | 0.342 |
| seq.22588.35 | Adhesion G protein-coupled receptor B2 | 0.528 | 0.525 |
| seq.22589.3 | Frizzled-2 | 0.592 | 0.297 |
| seq.22590.68 | Frizzled-1 | 0.853 | 0.448 |
| seq.22591.4 | Frizzled-9 | 0.841 | 0.855 |
| seq.22770.22 | Solute carrier family 25 member 38 | 0.794 | 0.686 |
| seq.22771.6 | Sterile alpha motif domain-containing protein 12 | 0.198 | 0.402 |
| seq.22772.125 | Sorting and assembly machinery component 50 homolog | 0.485 | 0.497 |
| seq.22773.69 | Succinate dehydrogenase [ubiquinone] iron-sulfur subunit, mitochondrial | 0.862 | 0.570 |
| seq.22774.20 | tRNA-splicing endonuclease subunit Sen34 | 0.401 | 0.069 |
| seq.22776.40 | Nuclear receptor 0B2 | 0.124 | 0.415 |
| seq.22777.46 | S-phase kinase-associated protein 2 | 0.981 | 0.941 |
| seq.22779.75 | Single-pass membrane and coiled-coil domain-containing protein 1 | 0.134 | 0.000 |
| seq.2278.61 | Metalloproteinase inhibitor 2 | 0.441 | 0.255 |
| seq.22782.80 | Kinetochore protein Spc25 | 0.930 | 0.891 |
| seq.22783.40 | Transcription factor PU.1 | 0.046 | 0.352 |
| seq.22784.4 | Spermatogenesis-associated protein 22 | 0.180 | 0.958 |
| seq.22786.57 | Spermatogenesis-associated protein 46 | 0.925 | 0.884 |
| seq.22787.39 | Sperm protein associated with the nucleus on the X chromosome N3 | 0.971 | 0.238 |
| seq.22788.10 | Sulfotransferase 6B1 | 0.757 | 0.792 |
| seq.22792.21 | Tryptophan--tRNA ligase, mitochondrial | 0.431 | 0.452 |
| seq.22793.110 | Transcription initiation factor IIA subunit 2 | 0.431 | 0.321 |
| seq.22795.17 | Transcriptional adapter 2-alpha | 0.429 | 0.445 |
| seq.22796.17 | Transcriptional adapter 1 | 0.467 | 0.909 |
| seq.22797.20 | Transcription initiation factor TFIID subunit 12 | 0.792 | 0.696 |
| seq.22798.111 | Transcription initiation factor TFIID subunit 8 | 0.350 | 0.068 |
| seq.22799.17 | TBC1 domain family member 28 | 0.857 | 0.180 |
| seq.22800.24 | Telethonin | 0.716 | 0.000 |
| seq.22801.83 | Telomere repeats-binding bouquet formation protein 2 | 0.820 | 0.581 |
| seq.22802.13 | Testis-expressed protein 30 | 0.776 | 0.438 |
| seq.22805.18 | THAP domain-containing protein 11 | 0.547 | 0.501 |
| seq.22806.1 | THAP domain-containing protein 2 | 0.393 | 0.814 |
| seq.22808.59 | Thyroid hormone receptor beta | 0.662 | 0.560 |
| seq.22809.32 | Mitochondrial import inner membrane translocase subunit Tim23 | 0.512 | 0.823 |
| seq.22810.41 | TIMELESS-interacting protein | 0.481 | 0.930 |
| seq.22811.5 | Translation machinery-associated protein 16 | 0.665 | 0.466 |
| seq.22812.4 | Trafficking protein particle complex subunit 6B | 0.906 | 0.252 |
| seq.22813.5 | TPT1-like protein | 0.372 | 0.906 |
| seq.22814.1 | Zinc finger protein RFP | 0.472 | 0.298 |
| seq.22815.4 | Tripartite motif-containing protein 40 | 0.245 | 0.693 |
| seq.22816.4 | Tripartite motif-containing protein 54 | 0.007 | 0.456 |
| seq.22817.126 | E3 ubiquitin-protein ligase TRIM9 | 0.016 | 0.955 |
| seq.22818.4 | Tricarboxylate transport protein, mitochondrial | 0.505 | 0.886 |
| seq.22821.50 | Uracil-DNA glycosylase | 0.846 | 0.791 |
| seq.22824.53 | V-type proton ATPase subunit C 2 | 0.000 | 0.000 |
| seq.22826.2 | Von Hippel-Lindau-like protein | 0.715 | 0.399 |
| seq.22831.11 | Z-DNA-binding protein 1 | 0.082 | 0.439 |
| seq.22832.9 | Zinc finger CCCH domain-containing protein 8 | 0.064 | 0.481 |
| seq.22833.74 | Zinc finger CCHC domain-containing protein 18 | 0.059 | 0.244 |
| seq.22834.21 | Probable palmitoyltransferase ZDHHC4 | 0.848 | 0.000 |
| seq.22838.39 | Zinc finger protein 230 | 0.311 | 0.041 |
| seq.22839.23 | Zinc finger protein 263 | 0.077 | 0.663 |
| seq.22841.16 | Zinc finger protein 696 | 0.416 | 0.000 |
| seq.22842.85 | Zinc finger protein 69 | 0.092 | 0.768 |
| seq.22844.4 | Zinc finger SWIM domain-containing protein 7 | 0.195 | 0.523 |
| seq.22845.3 | 28S ribosomal protein S14, mitochondrial | 0.911 | 0.658 |
| seq.22847.18 | Serine/threonine-protein kinase 4 | 0.633 | 0.264 |
| seq.22857.5 | BTB/POZ domain-containing protein KCTD7 | 0.462 | 0.772 |
| seq.22858.3 | Mitogen-activated protein kinase 6 | 0.594 | 0.000 |
| seq.22862.44 | Protein phosphatase 1M | 0.266 | 0.822 |
| seq.22946.55 | Alpha-1-syntrophin | 0.555 | 0.187 |
| seq.22948.13 | Bone morphogenetic protein 3 | 0.801 | 0.689 |
| seq.22950.6 | Butyrophilin subfamily 3 member A3 | 0.400 | 0.418 |
| seq.22952.28 | Cyclin-dependent kinase 2-associated protein 2 | 0.974 | 0.371 |
| seq.22953.85 | CCAAT/enhancer-binding protein beta | 0.846 | 0.075 |
| seq.22954.10 | Chloride intracellular channel protein 2 | 0.436 | 0.231 |
| seq.22955.28 | Retinol-binding protein 1 | 0.490 | 0.133 |
| seq.22958.6 | Destrin | 0.000 | 0.000 |
| seq.22959.32 | Dickkopf-related protein 4 | 0.188 | 0.601 |
| seq.22960.8 | Low affinity immunoglobulin gamma Fc region receptor II-b | 0.949 | 0.908 |
| seq.22961.7 | Ficolin-1 | 0.775 | 0.603 |
| seq.22963.3 | Vesicle-associated membrane protein 2 | 0.938 | 0.655 |
| seq.22966.20 | Gamma-aminobutyric acid receptor-associated protein | 0.345 | 0.287 |
| seq.22967.15 | Growth/differentiation factor 7 | 0.915 | 0.524 |
| seq.22968.9 | G antigen 2D | 0.671 | 0.738 |
| seq.22969.12 | C-C motif chemokine 7 | 0.882 | 0.235 |
| seq.22970.8 | PDZ domain-containing protein GIPC1 | 0.704 | 0.000 |
| seq.22972.26 | Gro-beta | 0.696 | 0.608 |
| seq.22973.8 | Gro-gamma | 0.482 | 0.197 |
| seq.22974.25 | Histone H2B type 2-E | 0.651 | 0.682 |
| seq.22977.18 | Protein HEXIM1 | 0.977 | 0.831 |
| seq.22978.13 | High mobility group protein B2 | 0.509 | 0.398 |
| seq.22980.37 | Homer protein homolog 1 | 0.458 | 0.056 |
| seq.22981.3 | Hippocalcin-like protein 1 | 0.639 | 0.270 |
| seq.22984.10 | Eukaryotic initiation factor 4A-II | 0.000 | 0.000 |
| seq.22985.160 | Insulin-like growth factor-binding protein 2 | 0.844 | 0.551 |
| seq.22987.69 | Mitogen-activated protein kinase 9 | 0.781 | 0.722 |
| seq.22989.24 | Protein lin-7 homolog A | 0.667 | 0.838 |
| seq.22990.20 | Protein lin-7 homolog B | 0.872 | 0.607 |
| seq.22991.9 | Melanoma-associated antigen 6 | 0.953 | 0.870 |
| seq.22992.6 | Microtubule-associated protein RP/EB family member 3 | 0.979 | 0.766 |
| seq.22993.9 | C-C motif chemokine 22 | 0.444 | 0.676 |
| seq.23000.22 | Neuroligin-1 | 0.598 | 0.358 |
| seq.23002.27 | Neurexophilin-1 | 0.762 | 0.000 |
| seq.23006.19 | Phosphatidylinositol 5-phosphate 4-kinase type-2 beta | 0.215 | 0.000 |
| seq.23007.8 | Junction plakoglobin | 0.532 | 0.385 |
| seq.23008.4 | Ras-related protein Rab-2A | 0.625 | 0.000 |
| seq.23016.24 | TATA-binding protein-associated factor 2N | 0.546 | 0.605 |
| seq.23017.17 | Reticulocalbin-3 | 0.424 | 0.795 |
| seq.23018.4 | Lithostathine-1-beta | 0.494 | 0.610 |
| seq.23020.18 | Regulator of G-protein signaling 4 | 0.574 | 0.284 |
| seq.23022.5 | Rab GDP dissociation inhibitor alpha | 0.977 | 0.466 |
| seq.23023.21 | Protein S100-A14 | 0.840 | 0.353 |
| seq.23024.25 | GTP-binding protein SAR1a | 0.888 | 0.529 |
| seq.23028.49 | Syntenin-2 | 0.861 | 0.568 |
| seq.23029.3 | Endophilin-A3 | 0.910 | 0.767 |
| seq.23030.4 | Sialic acid-binding Ig-like lectin 5 | 0.703 | 0.927 |
| seq.23031.6 | NAD-dependent protein deacetylase sirtuin-2 | 0.942 | 0.678 |
| seq.23034.13 | VPS10 domain-containing receptor SorCS3 | 0.663 | 0.000 |
| seq.23037.37 | Ubiquitin-conjugating enzyme E2 D3 | 0.000 | 0.758 |
| seq.23038.63 | Netrin receptor UNC5B | 0.582 | 0.479 |
| seq.23039.56 | Homeobox protein MOX-2 | 0.997 | 0.076 |
| seq.23148.100 | Endothelial cell-derived lipase | 0.643 | 0.238 |
| seq.23152.49 | BTB/POZ domain-containing adapter for CUL3-mediated RhoA degradation protein 2 | 0.587 | 0.799 |
| seq.23153.14 | BIK | 0.873 | 0.801 |
| seq.23156.146 | Caspase-10:region 2 | 0.484 | 0.364 |
| seq.23161.3 | BTB/POZ domain-containing protein KCTD6 | 0.827 | 0.190 |
| seq.23162.36 | Mitogen-activated protein kinase kinase kinase 10 | 0.853 | 0.411 |
| seq.23164.8 | Mitochondrial pyruvate carrier 1 | 0.838 | 0.348 |
| seq.23173.3 | Metalloproteinase inhibitor 1 | 0.793 | 0.727 |
| seq.23176.17 | Beta-dystroglycan | 0.248 | 0.000 |
| seq.23178.95 | Histamine N-methyltransferase | 0.520 | 0.505 |
| seq.23181.2 | Leukocyte tyrosine kinase receptor | 0.681 | 0.562 |
| seq.23183.6 | E3 ubiquitin-protein ligase RNF31 | 0.296 | 0.000 |
| seq.23187.9 | Deubiquitinating protein VCIP135 | 0.028 | 0.000 |
| seq.23200.25 | Complement C1q tumor necrosis factor-related protein 4 | 0.660 | 0.444 |
| seq.23202.78 | Ecto-ADP-ribosyltransferase 5 | 0.736 | 0.354 |
| seq.23203.3 | Retinoid-inducible serine carboxypeptidase | 0.653 | 0.147 |
| seq.23210.4 | ETS translocation variant 2 | 0.703 | 0.764 |
| seq.23212.8 | Protein EURL homolog | 0.973 | 0.591 |
| seq.23213.21 | Uncharacterized protein C11orf70 | 0.630 | 0.275 |
| seq.23224.11 | Regucalcin | 0.638 | 0.665 |
| seq.23225.26 | E3 ubiquitin-protein ligase TRIM62 | 0.832 | 0.785 |
| seq.23226.42 | ATP synthase B chain, mitochondrial | 0.000 | 0.355 |
| seq.23228.2 | Uncharacterized protein C12orf76 | 0.868 | 0.729 |
| seq.23241.27 | Mitochondrial import inner membrane translocase subunit Tim10 B | 0.259 | 0.000 |
| seq.23242.2 | Sjoegren syndrome nuclear autoantigen 1 | 0.751 | 0.669 |
| seq.23243.120 | 40S ribosomal protein S25 | 0.275 | 0.233 |
| seq.23245.5 | Speriolin-like protein | 0.472 | 0.616 |
| seq.23246.67 | Biogenesis of lysosome-related organelles complex 1 subunit 1 | 0.736 | 0.473 |
| seq.23248.1 | X antigen family member 2 | 0.516 | 0.000 |
| seq.23250.3 | Transcription elongation factor A protein-like 4 | 0.604 | 0.389 |
| seq.23252.4 | DnaJ homolog subfamily B member 3 | 0.968 | 0.947 |
| seq.23253.5 | Catechol O-methyltransferase domain-containing protein 1 | 0.757 | 0.911 |
| seq.23254.31 | NADH dehydrogenase [ubiquinone] 1 alpha subcomplex assembly factor 3 | 0.797 | 0.989 |
| seq.23255.7 | Ras-related protein Rab-33A | 0.428 | 0.514 |
| seq.23256.21 | TLD domain-containing protein 2 | 0.164 | 0.061 |
| seq.23257.14 | COMM domain-containing protein 10 | 0.100 | 0.000 |
| seq.23258.56 | Cytochrome c oxidase assembly factor 7 | 0.920 | 0.755 |
| seq.23259.23 | EEF1A lysine methyltransferase 1 | 0.398 | 0.170 |
| seq.23261.27 | Uncharacterized protein C1orf50 | 0.820 | 0.580 |
| seq.23262.11 | Beta-crystallin B3 | 0.919 | 0.764 |
| seq.23263.9 | BTB/POZ domain-containing protein KCTD15 | 0.653 | 0.548 |
| seq.23264.42 | Mediator of RNA polymerase II transcription subunit 10 | 0.226 | 0.000 |
| seq.23265.15 | Tumor protein D54 | 0.000 | 0.000 |
| seq.23266.62 | MIT domain-containing protein 1 | 0.145 | 0.000 |
| seq.23267.5 | BTB/POZ domain-containing protein KCTD6 | 0.453 | 0.719 |
| seq.23268.15 | Protein FRG1 | 0.833 | 0.890 |
| seq.23269.40 | CKLF-like MARVEL transmembrane domain-containing protein 4 | 0.583 | 0.392 |
| seq.23270.14 | Spermatogenesis-associated protein 33 | 0.414 | 0.777 |
| seq.23271.20 | 3-beta-hydroxysteroid-Delta(8),Delta(7)-isomerase | 0.000 | 0.000 |
| seq.23272.8 | Protein DPCD | 0.442 | 0.132 |
| seq.23273.42 | UPF0193 protein EVG1 | 0.580 | 0.866 |
| seq.23274.27 | EF-hand calcium-binding domain-containing protein 1 | 0.000 | 0.084 |
| seq.23275.9 | Sarcospan | 0.478 | 0.000 |
| seq.23276.14 | UPF0561 protein C2orf68 | 0.277 | 0.983 |
| seq.23278.13 | 39S ribosomal protein L12, mitochondrial | 0.176 | 0.000 |
| seq.23280.9 | Akirin-2 | 0.747 | 0.414 |
| seq.23281.29 | ADP-ribosylation factor-like protein 8A | 0.000 | 0.000 |
| seq.23282.19 | Ras-related protein Rab-37 | 0.829 | 0.801 |
| seq.23283.9 | Ras-related protein Rab-43 | 0.513 | 0.334 |
| seq.23284.5 | LIM domain only transcription factor protein 4 | 0.985 | 0.537 |
| seq.23285.51 | BTB/POZ domain-containing protein KCTD2 | 0.227 | 0.884 |
| seq.23286.2 | RING finger protein 141 | 0.852 | 0.433 |
| seq.23287.109 | NADH dehydrogenase | 0.000 | 0.000 |
| seq.23288.28 | Leucine-rich repeat-containing protein 20 | 0.339 | 0.724 |
| seq.23289.25 | Neurogenin-1 | 0.634 | 0.629 |
| seq.23290.3 | Survival motor neuron protein | 0.295 | 0.426 |
| seq.23291.18 | PTB-containing, cubilin and LRP1-interacting protein | 0.730 | 0.087 |
| seq.23292.1 | Centrosomal protein of 112 kDa | 0.663 | 0.687 |
| seq.23293.15 | Glutathione-specific gamma-glutamylcyclotransferase 1 | 0.716 | 0.284 |
| seq.23294.19 | Max-like protein X | 0.280 | 0.000 |
| seq.23296.29 | SARP-3 | 0.274 | 0.629 |
| seq.23298.148 | Acyl-coenzyme A thioesterase THEM4 | 0.341 | 0.000 |
| seq.23299.52 | Tubulin polymerization-promoting protein | 0.396 | 0.465 |
| seq.2330.2 | Stromal cell-derived factor 1 | 0.474 | 0.425 |
| seq.23300.3 | RNA-binding protein with multiple splicing 2 | 0.029 | 0.000 |
| seq.23301.2 | MSX-2 | 0.699 | 0.820 |
| seq.23302.19 | Proline-rich AKT1 substrate 1 | 0.353 | 0.000 |
| seq.23303.131 | Thioredoxin domain-containing protein 9 | 0.123 | 0.000 |
| seq.23304.1 | Protein NipSnap homolog 3B | 0.248 | 0.000 |
| seq.23305.38 | Receptor expression-enhancing protein 2 | 0.162 | 0.093 |
| seq.23306.37 | Polyamine-modulated factor 1 | 0.824 | 0.834 |
| seq.23307.7 | Clusterin-associated protein 1 | 0.946 | 0.507 |
| seq.23308.31 | BTB/POZ domain-containing protein KCTD1 | 0.966 | 0.380 |
| seq.23309.11 | Homologous-pairing protein 2 homolog | 0.834 | 0.608 |
| seq.23310.51 | Ras-related protein Rab-38 | 0.407 | 0.689 |
| seq.23311.19 | Ribonuclease P protein subunit p25 | 0.806 | 0.165 |
| seq.23312.16 | Rab-like protein 3 | 0.305 | 0.278 |
| seq.23314.46 | Coiled-coil domain-containing protein 103 | 0.627 | 0.637 |
| seq.23315.30 | Protein FAM9B | 0.814 | 0.887 |
| seq.23317.10 | Dehydrogenase/reductase SDR family member 11 | 0.314 | 0.092 |
| seq.23318.60 | Phosphatidylcholine transfer protein | 0.120 | 0.000 |
| seq.23319.6 | AN1-type zinc finger protein 2B | 0.237 | 0.006 |
| seq.23320.11 | Multivesicular body subunit 12B | 0.112 | 0.044 |
| seq.23321.42 | Coiled-coil domain-containing protein 95 | 0.720 | 0.647 |
| seq.23322.2 | Musculin | 0.147 | 0.000 |
| seq.23323.25 | Dual specificity phosphatase DUPD1 | 0.771 | 0.788 |
| seq.23324.6 | Heat shock protein beta-3 | 0.910 | 0.729 |
| seq.23325.18 | Uncharacterized protein C16orf45 | 0.340 | 0.903 |
| seq.23326.10 | Glutathione S-transferase A2 | 0.345 | 0.461 |
| seq.23327.1 | BLOC-1-related complex subunit 5 | 0.119 | 0.158 |
| seq.23329.52 | Ras-related protein Rab-4B | 0.000 | 0.000 |
| seq.2333.72 | Transforming growth factor beta-1 | 0.738 | 0.413 |
| seq.23330.12 | Uncharacterized protein C22orf13 | 0.415 | 0.119 |
| seq.23334.21 | Late cornified envelope protein 3C | 0.957 | 0.929 |
| seq.23335.31 | Protein FAM229A | 0.886 | 0.626 |
| seq.23336.2 | Putative potassium channel regulatory protein | 0.417 | 0.821 |
| seq.23337.54 | BTB/POZ domain-containing adapter for CUL3-mediated RhoA degradation protein 3 | 0.660 | 0.225 |
| seq.23339.46 | Transcription factor 24 | 0.936 | 0.983 |
| seq.23340.37 | Methylthioribose-1-phosphate isomerase | 0.616 | 0.705 |
| seq.23341.16 | Meiotic recombination protein DMC1/LIM15 homolog | 0.000 | 0.623 |
| seq.23342.4 | Arrestin domain-containing protein 5 | 0.000 | 0.689 |
| seq.23343.6 | Protein rogdi homolog | 0.600 | 0.953 |
| seq.23345.2 | N-terminal EF-hand calcium-binding protein 1 | 0.512 | 0.432 |
| seq.23349.5 | Choline-phosphate cytidylyltransferase A | 0.598 | 0.844 |
| seq.23350.66 | Protein FAM118B | 0.000 | 0.000 |
| seq.23351.59 | Protein arginine N-methyltransferase 1 | 0.454 | 0.380 |
| seq.23352.9 | Dysbindin | 0.531 | 0.866 |
| seq.23354.12 | Beta-crystallin A2 | 0.565 | 0.842 |
| seq.23356.32 | Coiled-coil domain-containing protein 24 | 0.780 | 0.936 |
| seq.23358.42 | Protein FAM118A | 0.524 | 0.903 |
| seq.23359.25 | Pre-mRNA-splicing regulator WTAP | 0.676 | 0.181 |
| seq.23361.20 | RNA-binding protein 4 | 0.229 | 0.000 |
| seq.23362.26 | Cytosolic 5'-nucleotidase 1A | 0.214 | 0.000 |
| seq.23363.41 | Poly(rC)-binding protein 2 | 0.000 | 0.000 |
| seq.23364.3 | Uridine-cytidine kinase 1 | 0.046 | 0.000 |
| seq.23365.7 | Fibronectin type III domain-containing protein 8 | 0.745 | 0.878 |
| seq.23366.15 | Ubiquitin-like protein 7 | 0.553 | 0.000 |
| seq.23367.8 | Ankyrin repeat domain-containing protein 40 | 0.423 | 0.225 |
| seq.23369.17 | Phytanoyl-CoA dioxygenase, peroxisomal | 0.490 | 0.102 |
| seq.23370.39 | Voltage-gated potassium channel subunit beta-3 | 0.025 | 0.895 |
| seq.23371.5 | NIF3-like protein 1 | 0.424 | 0.193 |
| seq.23372.112 | Endoplasmic reticulum-Golgi intermediate compartment protein 1 | 0.235 | 0.043 |
| seq.23373.77 | Diphthine methyl ester synthase | 0.000 | 0.089 |
| seq.23374.42 | N-terminal EF-hand calcium-binding protein 3 | 0.612 | 0.912 |
| seq.23375.4 | Ret finger protein-like 3 | 0.646 | 0.577 |
| seq.23376.56 | Actin-related protein 2/3 complex subunit 2 | 0.157 | 0.000 |
| seq.23378.29 | CGG triplet repeat-binding protein 1 | 0.742 | 0.692 |
| seq.23379.60 | UPF0472 protein C16orf72 | 0.606 | 0.729 |
| seq.23380.16 | Trans-3-hydroxy-L-proline dehydratase | 0.631 | 0.700 |
| seq.23381.8 | Septin-1 | 0.481 | 0.198 |
| seq.23382.30 | Variable charge X-linked protein 3 | 0.452 | 0.475 |
| seq.23384.19 | BTB/POZ domain-containing protein KCTD17 | 0.958 | 0.767 |
| seq.23385.18 | Cytosolic arginine sensor for mTORC1 subunit 1 | 0.698 | 0.385 |
| seq.23386.38 | T-complex protein 10A homolog 2 | 0.681 | 0.214 |
| seq.23387.18 | TIP41-like protein | 0.000 | 0.068 |
| seq.23389.28 | Fumarylacetoacetate hydrolase domain-containing protein 2A | 0.792 | 0.757 |
| seq.23390.6 | Coiled-coil domain-containing protein 107 | 0.282 | 0.000 |
| seq.23391.20 | Protein XRP2 | 0.478 | 0.070 |
| seq.23392.41 | Pleckstrin homology domain-containing family A member 3 | 0.470 | 0.000 |
| seq.23393.56 | 1-acylglycerol-3-phosphate O-acyltransferase ABHD5 | 0.727 | 0.000 |
| seq.23394.125 | Growth arrest-specific protein 2 | 0.483 | 0.295 |
| seq.23395.5 | KH domain-containing, RNA-binding, signal transduction-associated protein 2 | 0.294 | 0.034 |
| seq.23396.21 | Twinfilin-2 | 0.012 | 0.000 |
| seq.23398.1 | DNA-directed RNA polymerase II subunit GRINL1A | 0.625 | 0.538 |
| seq.23399.35 | Nucleoporin NUP53 | 0.614 | 0.398 |
| seq.23401.3 | DAZ-associated protein 1 | 0.757 | 0.941 |
| seq.23402.147 | Ganglioside-induced differentiation-associated protein 1-like 1 | 0.530 | 0.193 |
| seq.23403.64 | Cdc42 effector protein 4 | 0.884 | 0.485 |
| seq.23404.16 | Mitotic spindle assembly checkpoint protein MAD2B | 0.429 | 0.253 |
| seq.23405.33 | HORMA domain-containing protein 2 | 0.850 | 0.706 |
| seq.23406.3 | Ras association domain-containing protein 5 | 0.894 | 0.683 |
| seq.23407.9 | S-acyl fatty acid synthase thioesterase, medium chain | 0.596 | 0.227 |
| seq.23408.1 | Sorting nexin 16 | 0.679 | 0.194 |
| seq.23409.83 | Synapse-associated protein 1 | 0.308 | 0.000 |
| seq.23410.46 | Eukaryotic translation initiation factor 3 subunit M | 0.251 | 0.070 |
| seq.23411.108 | Paraneoplastic antigen Ma2 | 0.426 | 0.861 |
| seq.23413.38 | Ankyrin repeat domain-containing protein 45 | 0.928 | 0.843 |
| seq.23414.12 | Zinc finger protein 483 | 0.708 | 0.417 |
| seq.23416.47 | Sorting nexin-15 | 0.125 | 0.092 |
| seq.23418.66 | Coiled-coil domain-containing protein 92 | 0.302 | 0.000 |
| seq.23422.47 | Replication factor C 37 kDa subunit | 0.649 | 0.450 |
| seq.23424.4 | Ankyrin repeat domain-containing protein 63 | 0.611 | 0.482 |
| seq.23425.19 | Ermin | 0.601 | 0.545 |
| seq.23426.1 | Variable charge X-linked protein 1 | 0.791 | 0.076 |
| seq.23518.7 | Protein FAM151B | 0.210 | 0.348 |
| seq.23519.91 | Transmembrane protein 221 | 0.676 | 0.196 |
| seq.23520.60 | Beta-lactamase-like protein FLJ75971 | 0.765 | 0.713 |
| seq.23521.29 | Nucleoside diphosphate kinase 7 | 0.407 | 0.000 |
| seq.23522.1 | GTPase IMAP family member GIMD1 | 0.977 | 0.613 |
| seq.23523.14 | Protein FAM49A | 0.245 | 0.679 |
| seq.23524.2 | Protein N-terminal asparagine amidohydrolase | 0.508 | 0.000 |
| seq.23525.27 | Nitric oxide synthase-interacting protein | 0.943 | 0.093 |
| seq.23526.25 | Protein KTI12 homolog | 0.415 | 0.000 |
| seq.23528.199 | Phytanoyl-CoA hydroxylase-interacting protein-like | 0.684 | 0.346 |
| seq.23529.11 | StAR-related lipid transfer protein 7, mitochondrial | 0.924 | 0.990 |
| seq.23530.3 | Tropomodulin-4 | 0.726 | 0.928 |
| seq.23531.79 | Hematopoietic SH2 domain-containing protein | 0.562 | 0.195 |
| seq.23534.27 | Protein RTF2 homolog | 0.007 | 0.000 |
| seq.23535.3 | Protein FAM45A | 0.180 | 0.020 |
| seq.23536.14 | Uncharacterized protein C19orf84 | 0.770 | 0.248 |
| seq.23537.4 | Transcription factor Sp6 | 0.384 | 0.509 |
| seq.23540.3 | CDK-activating kinase assembly factor MAT1 | 0.894 | 0.936 |
| seq.23541.61 | Citrate lyase subunit beta-like protein, mitochondrial | 0.548 | 0.020 |
| seq.23542.8 | Golgi phosphoprotein 3-like | 0.000 | 0.000 |
| seq.23543.92 | Probable tRNA pseudouridine synthase 1 | 0.390 | 0.314 |
| seq.23544.10 | T-complex protein 10A homolog | 0.919 | 0.745 |
| seq.23545.6 | Nuclear distribution protein nudE-like 1 | 0.915 | 0.544 |
| seq.23546.9 | Hydroxyacylglutathione hydrolase-like protein | 0.381 | 0.844 |
| seq.23547.21 | Transcription elongation factor A protein 3 | 0.318 | 0.396 |
| seq.23550.10 | Keratinocyte differentiation factor 1 | 0.978 | 0.888 |
| seq.23551.15 | Transcriptional adapter 3 | 0.239 | 0.000 |
| seq.23553.1 | ABI gene family member 3 | 0.987 | 0.924 |
| seq.23554.2 | SH2 domain-containing adapter protein D | 0.792 | 0.391 |
| seq.23555.11 | GTP-binding protein RAD | 0.582 | 0.869 |
| seq.23556.4 | Transcription cofactor vestigial-like protein 4 | 0.641 | 0.593 |
| seq.23557.110 | F-actin-capping protein subunit beta | 0.378 | 0.892 |
| seq.23558.33 | Methyltransferase-like protein 2B | 0.823 | 0.617 |
| seq.23559.10 | DDRGK domain-containing protein 1 | 0.195 | 0.000 |
| seq.23560.154 | Centrosomal protein of 41 kDa | 0.000 | 0.000 |
| seq.23561.4 | Coiled-coil domain-containing protein 97 | 0.553 | 0.101 |
| seq.23564.83 | Protein-lysine methyltransferase METTL21C | 0.861 | 0.573 |
| seq.23566.6 | Apolipoprotein-L2 | 0.504 | 0.743 |
| seq.23567.37 | Guanine nucleotide-binding protein G(q) subunit alpha | 0.052 | 0.000 |
| seq.23568.41 | Homeobox protein Meis2 | 0.113 | 0.000 |
| seq.23569.53 | START domain-containing protein 10 | 0.502 | 0.462 |
| seq.23570.87 | Zinc finger protein with KRAB and SCAN domains 7 | 0.655 | 0.594 |
| seq.23571.93 | Coiled-coil domain-containing protein 94 | 0.891 | 0.530 |
| seq.23572.186 | tRNA wybutosine-synthesizing protein 3 homolog | 0.673 | 0.287 |
| seq.23575.58 | Cytochrome c oxidase assembly protein COX19 | 0.054 | 0.000 |
| seq.23576.46 | Protein BEX4 | 0.971 | 0.652 |
| seq.23578.6 | Small integral membrane protein 3 | 0.719 | 0.383 |
| seq.23579.3 | Kita-kyushu lung cancer antigen 1 | 0.340 | 0.525 |
| seq.2358.19 | Vascular endothelial growth factor receptor 3 | 0.849 | 0.663 |
| seq.23580.2 | Proline-rich protein 15 | 0.554 | 0.822 |
| seq.23581.131 | Obestatin | 0.768 | 0.781 |
| seq.23584.2 | Nucleotide triphosphate diphosphatase NUDT15 | 0.346 | 0.474 |
| seq.23586.32 | Cancer/testis antigen 1B | 0.833 | 0.186 |
| seq.23588.9 | Hydroxysteroid dehydrogenase-like protein 2 | 0.796 | 0.565 |
| seq.23589.21 | Protein phosphatase 1 regulatory subunit 27 | 0.705 | 0.901 |
| seq.23591.9 | piRNA biogenesis protein EXD1 | 0.845 | 0.000 |
| seq.23593.14 | Protein MB21D2 | 0.630 | 0.917 |
| seq.23594.2 | Nuclear receptor 2C2-associated protein | 0.542 | 0.000 |
| seq.23595.6 | Laccase domain-containing protein 1 | 0.468 | 0.000 |
| seq.23596.17 | Coiled-coil domain-containing protein 89 | 0.948 | 0.533 |
| seq.23597.11 | Arf-GAP domain and FG repeat-containing protein 2 | 0.255 | 0.000 |
| seq.23601.43 | Acyl-coenzyme A thioesterase 12 | 0.643 | 0.736 |
| seq.23602.35 | N-acyl-phosphatidylethanolamine-hydrolyzing phospholipase D | 0.974 | 0.679 |
| seq.23605.3 | T-complex protein 11-like protein 1 | 0.548 | 0.874 |
| seq.23606.18 | Sterile alpha motif domain-containing protein 4B | 0.168 | 0.000 |
| seq.23607.1 | Uncharacterized protein C16orf71 | 0.425 | 0.000 |
| seq.23611.16 | MARVEL domain-containing protein 2 | 0.942 | 0.000 |
| seq.23615.4 | TRM112-like protein | 0.536 | 0.325 |
| seq.23616.4 | Protein-arginine deiminase type-1 | 0.471 | 0.406 |
| seq.23617.15 | Huntingtin-interacting protein K | 0.931 | 0.716 |
| seq.23618.23 | Zinc finger protein 593 | 0.966 | 0.739 |
| seq.23619.235 | Leydig cell tumor 10 kDa protein homolog | 0.543 | 0.909 |
| seq.23620.16 | CapZ-interacting protein | 0.005 | 0.382 |
| seq.23621.326 | Mitochondrial import inner membrane translocase subunit Tim13 | 0.539 | 0.435 |
| seq.23622.128 | SOSS complex subunit C | 0.643 | 0.921 |
| seq.23623.48 | Diphosphoinositol polyphosphate phosphohydrolase 3-beta | 0.502 | 0.281 |
| seq.23624.34 | Gamma-secretase subunit PEN-2 | 0.407 | 0.467 |
| seq.23625.44 | Uncharacterized protein KIAA1143 | 0.031 | 0.312 |
| seq.23627.3 | UPF0369 protein C6orf57 | 0.760 | 0.861 |
| seq.23629.4 | Vimentin-type intermediate filament-associated coiled-coil protein | 0.603 | 0.809 |
| seq.23631.1 | N-acetylgalactosamine kinase | 0.145 | 0.303 |
| seq.23632.22 | Schlafen-like protein 1 | 0.485 | 0.422 |
| seq.23637.55 | E3 ubiquitin-protein ligase RNF25 | 0.622 | 0.816 |
| seq.23638.3 | Carnosine N-methyltransferase | 0.967 | 0.499 |
| seq.23639.93 | Probable aminopeptidase NPEPL1 | 0.717 | 0.560 |
| seq.23640.10 | EH domain-containing protein 2 | 0.657 | 0.660 |
| seq.23643.22 | Peroxisomal multifunctional enzyme type 2 | 0.000 | 0.000 |
| seq.23644.19 | Dihydroxyacetone kinase | 0.341 | 0.508 |
| seq.23645.49 | 2-hydroxyacyl-CoA lyase 1 | 0.681 | 0.821 |
| seq.23646.5 | Aryl-hydrocarbon-interacting protein-like 1 | 0.023 | 0.178 |
| seq.23648.36 | Protein DDI1 homolog 1 | 0.270 | 0.192 |
| seq.23649.6 | Kelch-like protein 2 | 0.235 | 0.588 |
| seq.23650.2 | Dematin | 0.238 | 0.000 |
| seq.23652.15 | Peroxisomal sarcosine oxidase | 0.750 | 0.622 |
| seq.23654.6 | Alpha-1,3-mannosyltransferase ALG2 | 0.101 | 0.020 |
| seq.23656.9 | Pseudouridylate synthase 7 homolog | 0.100 | 0.060 |
| seq.23657.102 | Heterogeneous nuclear ribonucleoprotein L-like | 0.603 | 0.526 |
| seq.23658.1 | Probable asparagine--tRNA ligase, mitochondrial | 0.316 | 0.312 |
| seq.23659.2 | Basic leucine zipper and W2 domain-containing protein 2 | 0.000 | 0.000 |
| seq.23660.112 | Kelch-like protein 41 | 0.786 | 0.199 |
| seq.23661.61 | Probable Xaa-Pro aminopeptidase 3 | 0.863 | 0.789 |
| seq.23662.10 | SH3 domain-binding protein 5 | 0.593 | 0.709 |
| seq.23663.28 | Vacuolar ATP synthase subunit C 1 | 0.213 | 0.008 |
| seq.23664.17 | Rhotekin | 0.569 | 0.691 |
| seq.23665.35 | 26S protease regulatory subunit 6A | 0.563 | 0.202 |
| seq.23666.35 | Bifunctional purine biosynthesis protein PURH | 0.217 | 0.100 |
| seq.23668.281 | Mitochondrial dynamics protein MID51 | 0.533 | 0.609 |
| seq.23669.20 | Glutamine--fructose-6-phosphate aminotransferase [isomerizing] 2 | 0.930 | 0.466 |
| seq.23670.11 | 2-(3-amino-3-carboxypropyl)histidine synthase subunit 2 | 0.318 | 0.873 |
| seq.23671.11 | Protein Spindly | 0.279 | 0.717 |
| seq.23672.10 | 26S proteasome non-ATPase regulatory subunit 6 | 0.979 | 0.213 |
| seq.23673.9 | Lipoamide acyltransferase component of branched-chain alpha-keto acid dehydrogenase complex, mitochondrial | 0.550 | 0.019 |
| seq.23675.16 | Centrosomal protein of 76 kDa | 0.529 | 0.355 |
| seq.23677.30 | NCK-interacting protein with SH3 domain | 0.052 | 0.000 |
| seq.23678.132 | Transcription factor SOX-10 | 0.826 | 0.287 |
| seq.23679.43 | Sister chromatid cohesion protein DCC1 | 0.788 | 0.985 |
| seq.23680.1 | MCP-1 Induced Protein | 0.349 | 0.261 |
| seq.23682.79 | Small RNA 2'-O-methyltransferase | 0.973 | 0.494 |
| seq.23683.79 | Tripartite motif-containing protein 55 | 0.653 | 0.766 |
| seq.23685.132 | Meiosis 1 arrest protein | 0.790 | 0.753 |
| seq.23686.44 | CWF19-like protein 1 | 0.000 | 0.362 |
| seq.23687.27 | TBC1 domain family member 20 | 0.152 | 0.000 |
| seq.23689.52 | Copine-6 | 0.814 | 0.717 |
| seq.23690.228 | RNA-binding protein 41 | 0.705 | 0.968 |
| seq.23691.2 | Tubulin--tyrosine ligase-like protein 12 | 0.498 | 0.148 |
| seq.23692.19 | Acyl-CoA synthetase short-chain family member 3, mitochondrial | 0.124 | 0.954 |
| seq.23693.4 | MAP kinase-interacting serine/threonine-protein kinase 1 | 0.691 | 0.000 |
| seq.23694.3 | Asparagine synthetase | 0.492 | 0.445 |
| seq.23695.1 | Fibronectin type III and SPRY domain-containing protein 1 | 0.486 | 0.908 |
| seq.23696.256 | tRNA (adenine(58)-N(1))-methyltransferase non-catalytic subunit TRM6 | 0.000 | 0.378 |
| seq.23699.61 | Dihydrolipoyllysine-residue succinyltransferase component of 2-oxoglutarate dehydrogenase complex, mitochondrial | 0.791 | 0.374 |
| seq.23700.42 | ATP-binding cassette sub-family F member 3 | 0.263 | 0.058 |
| seq.23702.98 | SH3KBP1-binding protein 1 | 0.930 | 0.965 |
| seq.23703.8 | PDZ and LIM domain protein 5 | 0.000 | 0.000 |
| seq.23704.11 | Transmembrane protein 24 | 0.000 | 0.000 |
| seq.23705.42 | CTP synthase 1 | 0.102 | 0.030 |
| seq.23706.13 | tRNA (adenine(58)-N(1))-methyltransferase, mitochondrial | 0.698 | 0.276 |
| seq.23767.1 | UPF0705 protein C11orf49 | 0.631 | 0.257 |
| seq.23771.17 | Cyclic AMP-dependent transcription factor ATF-5 | 0.304 | 0.276 |
| seq.23783.22 | Protein FAM110A | 0.453 | 0.443 |
| seq.2381.52 | Complement C5 | 0.233 | 0.433 |
| seq.23903.3 | Aspartate aminotransferase, mitochondrial | 0.367 | 0.000 |
| seq.23915.18 | Ras-related protein Rab-7 | 0.047 | 0.000 |
| seq.23923.26 | Protein phosphatase 1F | 0.027 | 0.000 |
| seq.23929.159 | Proline-rich transmembrane protein 2 | 0.993 | 0.968 |
| seq.23949.12 | CD29 | 0.062 | 0.000 |
| seq.23967.8 | PAXIP1-associated protein 1 | 0.000 | 0.000 |
| seq.23981.172 | Carboxypeptidase A1 | 0.393 | 0.429 |
| seq.24011.27 | V-set and immunoglobulin domain-containing protein 10-like | 0.378 | 0.258 |
| seq.24013.6 | Metabotropic glutamate receptor 4 | 0.458 | 0.736 |
| seq.24023.35 | soluble Endothelial protein C receptor | 0.731 | 0.777 |
| seq.24027.32 | Interferon epsilon-1 | 0.000 | 0.562 |
| seq.24050.26 | Glycogen synthase kinase-3 beta | 0.017 | 0.000 |
| seq.24055.44 | Cleavage stimulation factor 50 kDa subunit | 0.512 | 0.404 |
| seq.24111.10 | Elongation factor 1-gamma | 0.332 | 0.183 |
| seq.24179.48 | Phosphotriesterase-related protein | 0.797 | 0.419 |
| seq.2418.55 | Apolipoprotein E | 0.523 | 0.569 |
| seq.24201.86 | G antigen family C member 1 | 0.311 | 0.755 |
| seq.2421.7 | Brain-derived neurotrophic factor | 0.677 | 0.436 |
| seq.24211.25 | Hydroxymethylglutaryl-CoA lyase, mitochondrial | 0.308 | 0.000 |
| seq.24215.8 | Paired mesoderm homeobox protein 1 | 0.959 | 0.975 |
| seq.24216.30 | Mth938 domain-containing protein | 0.351 | 0.163 |
| seq.24217.2 | Calgranulin A | 0.048 | 0.452 |
| seq.24221.3 | Peroxisome proliferator activated receptor gamma | 0.162 | 0.627 |
| seq.24222.10 | Negative elongation factor A | 0.702 | 0.681 |
| seq.24223.5 | Glutathione S-transferase theta-2 | 0.406 | 0.928 |
| seq.24226.30 | Fascin | 0.339 | 0.521 |
| seq.24235.2 | EH domain-containing protein 1 | 0.000 | 0.000 |
| seq.24236.46 | Synaptic vesicle membrane protein VAT-1 homolog-like | 0.639 | 0.766 |
| seq.24237.115 | Voltage-dependent L-type calcium channel subunit beta-3 | 0.615 | 0.537 |
| seq.24244.81 | Rab-interacting lysosomal protein | 0.433 | 0.224 |
| seq.24245.2 | Kinesin light chain 3 | 0.337 | 0.206 |
| seq.24247.13 | UPF0235 protein C15orf40 | 0.780 | 0.000 |
| seq.24251.44 | Ribonuclease P protein subunit p25-like protein | 0.237 | 0.319 |
| seq.24252.85 | Probable peptide chain release factor C12orf65, mitochondrial | 0.445 | 0.136 |
| seq.24253.5 | Eukaryotic peptide chain release factor GTP-binding subunit ERF3B | 0.778 | 0.701 |
| seq.24255.38 | Epsin-1 | 0.000 | 0.000 |
| seq.24256.7 | Kelch-like protein 40 | 0.608 | 0.826 |
| seq.24259.24 | Forkhead box protein P4 | 0.436 | 0.087 |
| seq.24260.4 | Histone-lysine N-methyltransferase setd3 | 0.117 | 0.000 |
| seq.24261.202 | Death-associated protein kinase 3 | 0.578 | 0.000 |
| seq.24263.6 | Occludin | 0.653 | 0.429 |
| seq.24265.6 | MAGUK p55 subfamily member 2 | 0.491 | 0.917 |
| seq.24266.2 | Myocyte-specific enhancer factor 2D | 0.569 | 0.851 |
| seq.24267.24 | PWWP domain-containing protein 2B | 0.386 | 0.370 |
| seq.24268.21 | GRB2-associated-binding protein 1 | 0.252 | 0.064 |
| seq.24271.9 | Mannose-1-phosphate guanyltransferase alpha | 0.000 | 0.258 |
| seq.24273.43 | Thioredoxin domain-containing protein 3 | 0.715 | 0.885 |
| seq.24276.171 | Tetratricopeptide repeat protein 25 | 0.618 | 0.050 |
| seq.24277.22 | Copine-7 | 0.546 | 0.762 |
| seq.24278.4 | Ubiquitin carboxyl-terminal hydrolase 3 | 0.973 | 0.940 |
| seq.24279.30 | U3 small nucleolar RNA-associated protein 6 homolog | 0.869 | 0.943 |
| seq.24286.48 | Meiosis expressed gene 1 protein homolog | 0.961 | 0.638 |
| seq.24289.1 | Coiled-coil domain-containing transmembrane protein C7orf53 | 0.342 | 0.220 |
| seq.2429.27 | Complement component C8 | 0.727 | 0.675 |
| seq.24290.5 | Uncharacterized protein C20orf202 | 0.112 | 0.901 |
| seq.24293.39 | Nonspecific lipid-transfer protein:isoform 6 | 0.000 | 0.000 |
| seq.24294.13 | Chromosome transmission fidelity protein 8 homolog | 0.917 | 0.644 |
| seq.24299.1 | Carcinoembryonic antigen-related cell adhesion molecule 16 | 0.122 | 0.025 |
| seq.24304.3 | EF-hand calcium-binding domain-containing protein 4B | 0.971 | 0.734 |
| seq.24307.22 | Lysine-specific demethylase 8 | 0.671 | 0.815 |
| seq.24309.14 | Synaptotagmin-like protein 2 | 0.945 | 0.837 |
| seq.2431.17 | Cathepsin G | 0.562 | 0.427 |
| seq.24314.9 | Trafficking protein particle complex subunit 13 | 0.329 | 0.061 |
| seq.24316.10 | Probable methyltransferase BMT2 homolog | 0.665 | 0.925 |
| seq.24318.13 | FYVE, RhoGEF and PH domain-containing protein 2 | 0.702 | 0.654 |
| seq.24319.19 | D-ribitol-5-phosphate cytidylyltransferase | 0.888 | 0.372 |
| seq.24320.3 | Tripartite motif-containing protein 72 | 0.699 | 0.566 |
| seq.24321.67 | Nuclear prelamin A recognition factor | 0.607 | 0.481 |
| seq.24322.1 | General transcription factor IIE subunit 1 | 0.095 | 0.922 |
| seq.24323.43 | BRO1 domain-containing protein BROX | 0.000 | 0.247 |
| seq.2436.49 | C-X-C motif chemokine 16 | 0.705 | 0.552 |
| seq.24407.31 | Kinesin-like protein KIF3C | 0.551 | 0.324 |
| seq.24408.30 | Muscleblind-like protein 1 | 0.045 | 0.000 |
| seq.24409.2 | Retinol dehydrogenase 13 | 0.897 | 0.738 |
| seq.2441.2 | Fibroblast growth factor 10 | 0.881 | 0.749 |
| seq.24411.144 | E3 ubiquitin-protein ligase LRSAM1 | 0.171 | 0.307 |
| seq.24412.1 | Synaptonemal complex central element protein 1-like | 0.984 | 0.919 |
| seq.24413.96 | Peroxisomal coenzyme A diphosphatase NUDT7 | 0.903 | 0.200 |
| seq.24414.3 | Glycogen phosphorylase, brain form | 0.203 | 0.000 |
| seq.24416.20 | Staphylococcal nuclease domain-containing protein 1 | 0.352 | 0.360 |
| seq.24417.22 | Rab GTPase-binding effector protein 1 | 0.111 | 0.000 |
| seq.24418.14 | Homeobox protein HMX3 | 0.850 | 0.050 |
| seq.24419.3 | Molybdenum cofactor sulfurase | 0.888 | 0.669 |
| seq.24420.57 | Deoxyhypusine hydroxylase | 0.750 | 0.840 |
| seq.24422.36 | Spermatogenesis-associated protein 24 | 0.777 | 0.821 |
| seq.24423.9 | Protein phosphatase 1 regulatory subunit 3G | 0.919 | 0.777 |
| seq.24424.25 | TBC1 domain family member 25 | 0.776 | 0.481 |
| seq.24425.8 | Methionyl-tRNA synthetase, cytoplasmic | 0.039 | 0.000 |
| seq.24426.15 | Catenin alpha-1 | 0.619 | 0.186 |
| seq.24426.191 | Catenin alpha-1 | 0.653 | 0.245 |
| seq.24427.33 | Type II inositol-3,4-bisphosphate 4-phosphatase | 0.254 | 0.050 |
| seq.24428.76 | Lysophospholipase D GDPD1 | 0.491 | 0.246 |
| seq.2443.10 | Fibroblast growth factor 8 isoform B | 0.456 | 0.371 |
| seq.24430.11 | Zinc finger protein 580 | 0.654 | 0.853 |
| seq.24431.32 | Cap-specific mRNA (nucleoside-2'-O-)-methyltransferase 1 | 0.802 | 0.369 |
| seq.24432.3 | GRIP1-associated protein 1 | 0.026 | 0.000 |
| seq.24434.16 | Phytanoyl-CoA hydroxylase-interacting protein | 0.460 | 0.842 |
| seq.24435.1 | Dynamin-1 | 0.095 | 0.000 |
| seq.24436.23 | Disks large-associated protein 4 | 0.000 | 0.000 |
| seq.24437.6 | YjeF N-terminal domain-containing protein 3 | 0.884 | 0.913 |
| seq.24440.11 | BTB/POZ domain-containing protein KCTD3 | 0.389 | 0.174 |
| seq.24441.7 | Nuclear cap-binding protein subunit 1 | 0.000 | 0.429 |
| seq.24443.8 | Mortality factor 4-like protein 2 | 0.137 | 0.715 |
| seq.24444.10 | Tetratricopeptide repeat protein 27 | 0.357 | 0.000 |
| seq.24445.38 | Actin filament-associated protein 1-like 2 | 0.471 | 0.244 |
| seq.24446.65 | Muscleblind-like protein 2 | 0.052 | 0.000 |
| seq.24447.26 | Protein FAM221A | 0.617 | 0.000 |
| seq.24449.11 | Gamma-crystallin A | 0.801 | 0.272 |
| seq.24450.96 | UPF0692 protein C19orf54 | 0.850 | 0.255 |
| seq.24453.75 | Cytochrome c-type heme lyase | 0.693 | 0.902 |
| seq.24454.27 | Probable bifunctional methylenetetrahydrofolate dehydrogenase/cyclohydrolase 2 | 0.739 | 0.119 |
| seq.24455.2 | Arf-GAP with SH3 domain, ANK repeat and PH domain-containing protein 3 | 0.672 | 0.512 |
| seq.24456.3 | Visual system homeobox 1 | 0.542 | 0.112 |
| seq.24457.12 | USF2 | 0.633 | 0.677 |
| seq.24459.15 | Coatomer subunit beta' | 0.000 | 0.000 |
| seq.24462.4 | Cyclic nucleotide-gated cation channel beta-1 | 0.909 | 0.132 |
| seq.24463.1 | Protein NDRG2 | 0.896 | 0.937 |
| seq.24465.28 | synovial sarcoma, X breakpoint 4 | 0.838 | 0.630 |
| seq.24468.36 | Beta-adducin | 0.764 | 0.000 |
| seq.24469.10 | Phosphatidate phosphatase LPIN1 | 0.111 | 0.385 |
| seq.2447.7 | Group IIE secretory phospholipase A2 | 0.620 | 0.681 |
| seq.24470.20 | Poly(rC)-binding protein 3 | 0.034 | 0.000 |
| seq.24471.2 | SET-binding protein | 0.754 | 0.002 |
| seq.24472.28 | Sharpin | 0.817 | 0.000 |
| seq.24473.11 | Cancer/testis antigen family 45 member A3 | 0.822 | 0.055 |
| seq.24474.12 | Tetratricopeptide repeat protein 9A | 0.439 | 0.177 |
| seq.24475.45 | Sorting nexin-12 | 0.000 | 0.000 |
| seq.24476.18 | Activating signal cointegrator 1 complex subunit 2 | 0.368 | 0.591 |
| seq.24479.25 | Transmembrane protein 225B | 0.819 | 0.222 |
| seq.24485.32 | B-cell receptor-associated protein 31 | 0.939 | 0.877 |
| seq.24486.1 | B-cell lymphoma/leukemia 11A | 0.861 | 0.438 |
| seq.24487.95 | Biogenesis of lysosome-related organelles complex 1 subunit 3 | 0.477 | 0.385 |
| seq.24488.8 | Protein HID1 | 0.180 | 0.853 |
| seq.24489.34 | Transcription factor HES-3 | 0.617 | 0.783 |
| seq.2449.1 | Calcium-dependent phospholipase A2 | 0.731 | 0.070 |
| seq.24490.16 | THAP domain-containing protein 4 | 0.644 | 0.553 |
| seq.24493.10 | Myotubularin-related protein 7 | 0.547 | 0.941 |
| seq.24494.67 | Katanin p60 ATPase-containing subunit A-like 1 | 0.421 | 0.651 |
| seq.24496.26 | RILP-like protein 1 | 0.578 | 0.000 |
| seq.24497.8 | Probable E3 ubiquitin-protein ligase TRIML1 | 0.651 | 0.888 |
| seq.24499.36 | Lengsin | 0.721 | 0.607 |
| seq.24634.1 | Protein ripply3 | 0.967 | 0.898 |
| seq.24635.25 | Trafficking protein particle complex subunit 6A | 0.716 | 0.293 |
| seq.24636.9 | Dr1-associated corepressor | 0.903 | 0.747 |
| seq.24637.3 | Transcription elongation factor A protein-like 5 | 0.486 | 0.335 |
| seq.24638.3 | Leucine repeat adapter protein 25 | 0.123 | 0.690 |
| seq.24640.63 | Hairy/enhancer-of-split related with YRPW motif protein 1 | 0.521 | 0.085 |
| seq.24641.10 | Claudin-1 | 0.182 | 0.615 |
| seq.24642.21 | Protein FAM92A | 0.904 | 0.945 |
| seq.24643.21 | Lambda-crystallin homolog | 0.545 | 0.000 |
| seq.24645.2 | Bridging integrator 3 | 0.747 | 0.314 |
| seq.24646.47 | Serine--pyruvate aminotransferase | 0.419 | 0.411 |
| seq.24647.3 | C->U-editing enzyme APOBEC-2 | 0.912 | 0.654 |
| seq.24648.8 | Histone RNA hairpin-binding protein | 0.562 | 0.112 |
| seq.24649.11 | Protein N-terminal glutamine amidohydrolase | 0.451 | 0.901 |
| seq.24651.1 | Reticulon-1 | 0.749 | 0.688 |
| seq.24654.13 | Transcription cofactor HES-6 | 0.003 | 0.793 |
| seq.24655.23 | RILP-like protein 2 | 0.150 | 0.000 |
| seq.24656.6 | Sesquipedalian-2 | 0.199 | 0.373 |
| seq.24658.98 | Coiled-coil domain-containing protein 115 | 0.198 | 0.107 |
| seq.24659.6 | DnaJ homolog subfamily B member 13 | 0.879 | 0.361 |
| seq.24661.4 | MAP6 domain-containing protein 1 | 0.414 | 0.046 |
| seq.24662.78 | Tetratricopeptide repeat protein 36 | 0.693 | 0.461 |
| seq.24664.3 | UPF0510 protein INM02 | 0.775 | 0.347 |
| seq.24665.48 | GTP-binding protein REM 1 | 0.614 | 0.913 |
| seq.24667.27 | Cancer/testis antigen family 45 member A1 | 0.000 | 0.685 |
| seq.24669.12 | DnaJ homolog subfamily C member 5B | 0.857 | 0.851 |
| seq.24670.1 | PIH1 domain-containing protein 1 | 0.142 | 0.000 |
| seq.24671.15 | Apoptosis-associated speck-like protein containing a CARD | 0.451 | 0.422 |
| seq.24674.22 | Egl nine homolog 2 | 0.255 | 0.460 |
| seq.24675.2 | Microtubule-associated serine/threonine-protein kinase 4 | 0.706 | 0.581 |
| seq.24676.105 | Uncharacterized protein C21orf59 | 0.839 | 0.310 |
| seq.24677.106 | Protein RER1 | 0.294 | 0.000 |
| seq.24678.10 | Cell death regulator Aven | 0.792 | 0.032 |
| seq.24679.35 | Transcription factor ETV7 | 0.762 | 0.654 |
| seq.2468.62 | C-C motif chemokine 20 | 0.906 | 0.431 |
| seq.24680.51 | Thioredoxin domain-containing protein 1 | 0.706 | 0.621 |
| seq.24681.2 | Phosphoserine phosphatase | 0.188 | 0.157 |
| seq.24682.35 | Cytochrome b reductase 1 | 0.355 | 0.116 |
| seq.24683.11 | GTPase IMAP family member 7 | 0.613 | 0.298 |
| seq.24684.7 | GTPase IMAP family member 4 | 0.371 | 0.489 |
| seq.24685.12 | Sorting nexin-11 | 0.179 | 0.000 |
| seq.24686.19 | Acyl-CoA-binding domain-containing protein 4 | 0.610 | 0.450 |
| seq.24687.18 | Alpha-tocopherol transfer protein-like | 0.289 | 0.031 |
| seq.24688.9 | Doublesex- and mab-3-related transcription factor B1 | 0.941 | 0.799 |
| seq.24689.9 | Polymerase I and transcript release factor | 0.115 | 0.000 |
| seq.24690.1 | Calcium-binding protein 2 | 0.641 | 0.551 |
| seq.24693.5 | Chloride intracellular channel protein 3 | 0.551 | 0.491 |
| seq.24694.158 | Ezrin-radixin-moesin-binding phosphoprotein 50 | 0.077 | 0.000 |
| seq.24695.8 | Pleckstrin homology domain-containing family B member 1 | 0.875 | 0.970 |
| seq.24696.14 | PDZ domain-containing protein MAGIX | 0.971 | 0.444 |
| seq.24697.48 | Heterogeneous nuclear ribonucleoprotein A0 | 0.124 | 0.415 |
| seq.24698.12 | Pre-mRNA-splicing factor 18 | 0.454 | 0.187 |
| seq.24699.20 | ADP-ribosylation factor-like protein 6-interacting protein 1 | 0.275 | 0.000 |
| seq.24701.21 | Dystrobrevin alpha | 0.635 | 0.665 |
| seq.24702.31 | Coiled-coil domain-containing protein 149 | 0.104 | 0.057 |
| seq.24704.38 | COMM domain-containing protein 5 | 0.318 | 0.349 |
| seq.24706.73 | Craniofacial development protein 1 | 0.640 | 0.287 |
| seq.24707.6 | Uncharacterized protein CXorf38 | 0.194 | 0.000 |
| seq.24708.7 | Short-chain dehydrogenase/reductase 3 | 0.495 | 0.275 |
| seq.24710.1 | Microtubule-associated tumor suppressor candidate 2 | 0.836 | 0.800 |
| seq.24711.21 | Short stature homeobox protein | 0.710 | 0.928 |
| seq.24712.6 | GTP-binding protein 10 | 0.879 | 0.592 |
| seq.24713.4 | PDZ domain-containing protein MAGIX | 0.842 | 0.550 |
| seq.24717.26 | Serine/threonine-protein phosphatase 2A regulatory subunit B' | 0.335 | 0.000 |
| seq.24718.8 | Leucine-rich repeat-containing protein 75A | 0.804 | 0.977 |
| seq.24720.10 | Ankyrin repeat domain-containing protein 16 | 0.445 | 0.010 |
| seq.24721.17 | Golgi phosphoprotein 3 | 0.483 | 0.080 |
| seq.24722.13 | Spindlin-3 | 0.878 | 0.110 |
| seq.24723.58 | Eukaryotic translation initiation factor 2 subunit 2 | 0.223 | 0.000 |
| seq.24724.4 | Vacuolar protein sorting-associated protein 26B | 0.469 | 0.274 |
| seq.24725.4 | COP9 signalosome complex subunit 5 | 0.273 | 0.590 |
| seq.2474.54 | Serum amyloid P-component | 0.591 | 0.703 |
| seq.2475.1 | Mast/stem cell growth factor receptor Kit | 0.776 | 0.386 |
| seq.2480.58 | Metalloproteinase inhibitor 3 | 0.764 | 0.533 |
| seq.24891.54 | Epidermal growth factor receptor kinase substrate 8-like protein 2 | 0.720 | 0.560 |
| seq.24892.8 | Testicular spindle-associated protein SHCBP1L | 0.475 | 0.215 |
| seq.24895.25 | F-BAR and double SH3 domains protein 1 | 0.698 | 0.933 |
| seq.24896.5 | Menin | 0.585 | 0.221 |
| seq.24898.39 | Sec1 family domain-containing protein 1 | 0.396 | 0.609 |
| seq.24899.13 | Epidermal growth factor receptor kinase substrate 8-like protein 1 | 0.281 | 0.146 |
| seq.24900.46 | VPS9 domain-containing protein 1 | 0.992 | 0.365 |
| seq.24901.4 | Villin-1 | 0.206 | 0.000 |
| seq.24902.84 | Gephyrin | 0.491 | 0.576 |
| seq.24903.7 | Kelch-like protein 14 | 0.414 | 0.758 |
| seq.24904.121 | Syntaxin-binding protein 4 | 0.543 | 0.300 |
| seq.24905.22 | Protein CXorf40A | 0.584 | 0.913 |
| seq.24907.3 | Mitochondrial-processing peptidase subunit alpha | 0.077 | 0.159 |
| seq.24908.19 | Amphiphysin | 0.642 | 0.726 |
| seq.24909.40 | 1-phosphatidylinositol-4,5-bisphosphate phosphodiesterase delta-1 | 0.544 | 0.595 |
| seq.24910.18 | Non-POU domain-containing octamer-binding protein | 0.568 | 0.130 |
| seq.24911.57 | LRP | 0.477 | 0.000 |
| seq.24912.40 | WD repeat-containing protein 26 | 0.920 | 0.790 |
| seq.24914.10 | Enhancer of zeste homolog 2 | 0.000 | 0.616 |
| seq.24915.123 | Calpain-13 | 0.361 | 0.357 |
| seq.24917.95 | DnaJ homolog subfamily C member 1 | 0.183 | 0.000 |
| seq.24920.6 | Glutaminase kidney isoform, mitochondrial | 0.527 | 0.058 |
| seq.24921.73 | HIRA-interacting protein 3 | 0.944 | 0.376 |
| seq.24922.19 | NHL repeat-containing protein 2 | 0.108 | 0.364 |
| seq.24923.27 | Secernin-3 | 0.769 | 0.567 |
| seq.24926.9 | Far upstream element-binding protein 2 | 0.421 | 0.427 |
| seq.24928.20 | Mitotic-spindle organizing protein 2A | 0.415 | 0.674 |
| seq.24929.26 | Transcription factor SOX-6 | 0.832 | 0.581 |
| seq.24931.9 | Epidermal growth factor receptor kinase substrate 8-like protein 3 | 0.737 | 0.757 |
| seq.24934.96 | Opioid growth factor receptor | 0.731 | 0.657 |
| seq.24935.62 | Forkhead box protein P1 | 0.456 | 0.712 |
| seq.24937.7 | Rho GTPase-activating protein 26 | 0.382 | 0.759 |
| seq.24938.7 | AP-2 complex subunit beta-1 | 0.079 | 0.000 |
| seq.24940.3 | Inhibitor of nuclear factor kappa B kinase beta subunit | 0.200 | 0.000 |
| seq.24941.14 | Clustered mitochondria protein homolog | 0.282 | 0.201 |
| seq.24942.22 | Protein unc-13 homolog D | 0.049 | 0.000 |
| seq.24944.12 | RalBP1-associated Eps domain-containing protein 2 | 0.850 | 0.743 |
| seq.24945.16 | Sorting nexin-27 | 0.082 | 0.035 |
| seq.24946.9 | Rho GTPase-activating protein 22 | 0.429 | 0.000 |
| seq.24947.40 | Numb-like protein | 0.804 | 0.227 |
| seq.24948.79 | Paraspeckle component 1 | 0.677 | 0.519 |
| seq.24950.21 | Specifically androgen-regulated gene protein | 0.299 | 0.000 |
| seq.24951.3 | Protein VAC14 homolog | 0.487 | 0.794 |
| seq.24952.28 | Far upstream element-binding protein 3 | 0.078 | 0.000 |
| seq.24953.27 | Kinesin-like protein KIF3B | 0.859 | 0.073 |
| seq.24954.83 | AP-1 complex subunit beta-1 | 0.487 | 0.330 |
| seq.24955.116 | Zinc fingers and homeoboxes protein 2 | 0.806 | 0.959 |
| seq.24956.1 | N6-adenosine-methyltransferase 70 kDa subunit | 0.394 | 0.675 |
| seq.24957.6 | Espin | 0.473 | 0.372 |
| seq.24958.3 | DNA-binding protein RFX5 | 0.693 | 0.714 |
| seq.24959.20 | Cytochrome b5 reductase 4 | 0.366 | 0.000 |
| seq.24960.48 | Huntingtin-interacting protein 1-related protein | 0.494 | 0.349 |
| seq.24962.7 | SH3 domain-containing kinase-binding protein 1 | 0.251 | 0.272 |
| seq.24963.1 | Armadillo repeat-containing protein 3 | 0.570 | 0.678 |
| seq.24967.4 | Protocadherin-1 | 0.902 | 0.515 |
| seq.24968.10 | T-lymphoma invasion and metastasis-inducing protein 2 | 0.819 | 0.000 |
| seq.24970.117 | Serrate RNA effector molecule homolog | 0.442 | 0.838 |
| seq.24971.2 | Phosphatidylinositide phosphatase SAC2 | 0.390 | 0.178 |
| seq.24973.11 | MICAL-like protein 1 | 0.478 | 0.000 |
| seq.24975.3 | Fc receptor-like A | 0.680 | 0.402 |
| seq.24977.18 | Villin-like protein | 0.759 | 0.358 |
| seq.24979.17 | Eukaryotic translation initiation factor 3 subunit B | 0.129 | 0.000 |
| seq.24981.8 | Cytoplasmic dynein 1 light intermediate chain 1 | 0.000 | 0.211 |
| seq.24982.33 | MAGUK p55 subfamily member 5 | 0.728 | 0.837 |
| seq.24983.119 | ELKS/RAB6-interacting/CAST family member 1 | 0.059 | 0.000 |
| seq.2500.2 | Angiopoietin-4 | 0.835 | 0.211 |
| seq.2501.51 | Cadherin-1 | 0.317 | 0.256 |
| seq.25033.194 | Transitional endoplasmic reticulum ATPase | 0.185 | 0.213 |
| seq.25036.10 | Ran GTPase-activating protein 1 | 0.235 | 0.349 |
| seq.25037.4 | Aspartyl aminopeptidase | 0.901 | 0.722 |
| seq.25038.5 | Wee1-like protein kinase 2 | 0.281 | 0.848 |
| seq.25039.10 | Kelch-like protein 3 | 0.453 | 0.000 |
| seq.25041.11 | 6-phosphofructo-2-kinase/fructose-2,6-bisphosphatase 4 | 0.347 | 0.364 |
| seq.25042.3 | NAD-dependent malic enzyme, mitochondrial | 0.186 | 0.000 |
| seq.25043.74 | Succinyl-CoA:3-ketoacid-coenzyme A transferase 1, mitochondrial | 0.246 | 0.000 |
| seq.25048.30 | Protein kinase C and casein kinase substrate in neurons protein 2 | 0.000 | 0.000 |
| seq.25049.8 | Nonspecific lipid-transfer protein:isoform SCPx | 0.000 | 0.000 |
| seq.2505.49 | GDNF family receptor alpha-3 | 0.784 | 0.450 |
| seq.25050.82 | Tripeptidyl-peptidase 2 | 0.661 | 0.222 |
| seq.25051.104 | Fat mass and obesity-associated protein | 0.411 | 0.668 |
| seq.25052.3 | Cytokine receptor-like factor 3 | 0.872 | 0.547 |
| seq.25053.1 | Cytoplasmic dynein 1 light intermediate chain 2 | 0.156 | 0.000 |
| seq.25054.15 | Pre-mRNA-processing factor 19 | 0.777 | 0.158 |
| seq.25055.56 | Oxysterol-binding protein 1 | 0.000 | 0.000 |
| seq.25057.2 | Alpha-actinin-4 | 0.256 | 0.000 |
| seq.25058.23 | Membrane-associated phosphatidylinositol transfer protein 3 | 0.491 | 0.092 |
| seq.25059.18 | Histone deacetylase 6 | 0.489 | 0.000 |
| seq.25060.18 | NADP-dependent malic enzyme | 0.374 | 0.477 |
| seq.25061.8 | Engulfment and cell motility protein 2 | 0.309 | 0.168 |
| seq.25064.34 | Type I inositol-3,4-bisphosphate 4-phosphatase | 0.832 | 0.636 |
| seq.25065.22 | Myosin-binding protein H | 0.685 | 0.208 |
| seq.25066.32 | Glycogenin-2 | 0.594 | 0.842 |
| seq.25068.131 | Glutamate dehydrogenase 2, mitochondrial | 0.101 | 0.283 |
| seq.25070.18 | Protein-methionine sulfoxide oxidase MICAL1 | 0.096 | 0.000 |
| seq.25072.11 | Chromodomain-helicase-DNA-binding protein 1-like | 0.850 | 0.489 |
| seq.25073.13 | Ubiquitin conjugation factor E4 A | 0.728 | 0.592 |
| seq.25074.119 | Putative glycerol kinase 5 | 0.374 | 0.589 |
| seq.25075.2 | Zinc finger and BTB domain-containing protein 10 | 0.949 | 0.728 |
| seq.25076.2 | Selenium-binding protein 1 | 0.365 | 0.831 |
| seq.25078.13 | Cysteine protease ATG4C | 0.256 | 0.000 |
| seq.25080.16 | UDP-glucuronosyltransferase 2B15 | 0.811 | 0.681 |
| seq.25081.6 | Protein FAM160B1 | 0.192 | 0.000 |
| seq.25082.3 | Neural Wiskott-Aldrich syndrome protein | 0.559 | 0.487 |
| seq.25083.26 | Protein diaphanous homolog 1 | 0.202 | 0.000 |
| seq.25085.37 | Tether containing UBX domain for GLUT4 | 0.345 | 0.000 |
| seq.25086.83 | GRAM domain-containing protein 2B | 0.669 | 0.717 |
| seq.25087.11 | Dystrobrevin alpha | 0.437 | 0.961 |
| seq.25088.42 | Phosphorylase b kinase regulatory subunit alpha, skeletal muscle isoform | 0.565 | 0.762 |
| seq.25089.21 | Transcription intermediary factor 1-beta | 0.785 | 0.800 |
| seq.25091.83 | SLIT-ROBO Rho GTPase-activating protein 2 | 0.796 | 0.340 |
| seq.25092.32 | ATP-dependent RNA helicase DDX1 | 0.279 | 0.000 |
| seq.25093.96 | Vang-like protein 1 | 0.616 | 0.000 |
| seq.25094.9 | Acetyl-coenzyme A synthetase, cytoplasmic | 0.178 | 0.175 |
| seq.25095.1 | Synphilin-1 | 0.891 | 0.775 |
| seq.25096.58 | Myosin-binding protein C, fast-type | 0.763 | 0.708 |
| seq.25099.4 | Calcium-dependent secretion activator 2 | 0.791 | 0.146 |
| seq.25100.11 | C-Maf-inducing protein | 0.353 | 0.000 |
| seq.25102.23 | Elongator complex protein 1 | 0.927 | 0.925 |
| seq.25103.45 | Vacuolar protein sorting-associated protein 53 homolog | 0.186 | 0.000 |
| seq.25104.10 | Transcription factor CP2-like protein 1 | 0.000 | 0.000 |
| seq.25105.70 | Eukaryotic translation initiation factor 2A | 0.653 | 0.440 |
| seq.25105.87 | Eukaryotic translation initiation factor 2A | 0.025 | 0.000 |
| seq.25108.6 | Arginyl-tRNA--protein transferase 1 | 0.085 | 0.000 |
| seq.25110.19 | Exocyst complex component 7 | 0.811 | 0.894 |
| seq.25111.24 | E3 ubiquitin-protein ligase RAD18 | 0.231 | 0.344 |
| seq.25112.6 | Ribosomal protein S6 kinase beta-2 | 0.712 | 0.448 |
| seq.25114.256 | TBC1 domain family member 24 | 0.003 | 0.228 |
| seq.25115.21 | FH1/FH2 domain-containing protein 1 | 0.057 | 0.000 |
| seq.25116.11 | Coiled-coil domain-containing protein 9 | 0.378 | 0.000 |
| seq.25117.17 | PGM5 | 0.634 | 0.463 |
| seq.25118.45 | Protein bicaudal D homolog 1 | 0.502 | 0.778 |
| seq.25121.7 | Nodal modulator 2 | 0.782 | 0.560 |
| seq.25123.198 | DNA-(apurinic or apyrimidinic site) lyase 2 | 0.627 | 0.928 |
| seq.25124.21 | Zinc finger and BTB domain-containing protein 7A | 0.690 | 0.723 |
| seq.25125.7 | SH3 and cysteine-rich domain-containing protein | 0.981 | 0.476 |
| seq.25126.19 | E3 ubiquitin-protein ligase HECW2 | 0.894 | 0.870 |
| seq.2514.65 | Ephrin-B3:Extracellular domain | 0.694 | 0.455 |
| seq.2515.14 | GDNF family receptor alpha-2 | 0.836 | 0.450 |
| seq.2516.57 | C-C motif chemokine 21 | 0.721 | 0.613 |
| seq.25209.15 | Sodium/potassium-transporting ATPase gamma chain | 0.740 | 0.768 |
| seq.25211.14 | Guanine nucleotide-binding protein G(I)/G(S)/G(O) gamma-12 subunit | 0.479 | 0.550 |
| seq.25215.1 | Mitochondrial import inner membrane translocase subunit Tim10 | 0.284 | 0.000 |
| seq.25216.8 | Calgranulin A | 0.178 | 0.507 |
| seq.25217.35 | Sperm protein associated with the nucleus on the X chromosome A | 0.891 | 0.923 |
| seq.25218.10 | Signal peptidase complex subunit 1 | 0.622 | 0.882 |
| seq.25219.17 | V-type proton ATPase subunit F | 0.267 | 0.000 |
| seq.25220.8 | Cysteine-rich tail protein 1 | 0.789 | 0.746 |
| seq.25225.14 | Ferritin heavy polypeptide-like 17 | 0.889 | 0.409 |
| seq.25227.20 | Transcription elongation factor A protein-like 2 | 0.155 | 0.902 |
| seq.2523.31 | C-C motif chemokine 5 | 0.693 | 0.584 |
| seq.25232.4 | EH domain-containing protein 3 | 0.075 | 0.000 |
| seq.25233.2 | Mannose-6-phosphate isomerase | 0.271 | 0.096 |
| seq.25235.2 | Diphthine methyltransferase | 0.600 | 0.375 |
| seq.25236.11 | Methyltransferase-like 26 | 0.463 | 0.450 |
| seq.25238.6 | Apoptosis facilitator Bcl-2-like protein 14 | 0.420 | 0.552 |
| seq.2524.56 | High mobility group protein B1 | 0.052 | 0.265 |
| seq.25240.1 | Protein-lysine N-methyltransferase EEF2KMT | 0.280 | 0.928 |
| seq.25242.12 | Ciliogenesis-associated TTC17-interacting protein | 0.446 | 0.559 |
| seq.25244.9 | Probable E3 ubiquitin-protein ligase HECTD3 | 0.956 | 0.389 |
| seq.25245.22 | Synembryn-A | 0.628 | 0.684 |
| seq.25247.12 | Armadillo repeat-containing protein 8 | 0.602 | 0.766 |
| seq.25248.28 | Protein numb homolog | 0.000 | 0.000 |
| seq.25249.33 | Pyruvate dehydrogenase E1 component subunit alpha, testis-specific form, mitochondrial | 0.446 | 0.202 |
| seq.25251.6 | Ectonucleotide pyrophosphatase/phosphodiesterase family member 3 | 0.377 | 0.104 |
| seq.25252.29 | MTSS1-like protein | 0.010 | 0.000 |
| seq.25253.17 | Guanine nucleotide-binding protein G(s) subunit alpha isoforms | 0.250 | 0.026 |
| seq.25255.4 | Transmembrane and ubiquitin-like domain-containing protein 1 | 0.808 | 0.527 |
| seq.25256.153 | Elongation factor 1-alpha 1 | 0.093 | 0.229 |
| seq.25256.23 | Elongation factor 1-alpha 1 | 0.000 | 0.000 |
| seq.25257.162 | Oligodendrocyte transcription factor 1 | 0.526 | 0.220 |
| seq.25258.41 | Spermatogenesis-associated protein 5 | 0.828 | 0.834 |
| seq.25259.7 | Uncharacterized protein C17orf62 | 0.644 | 0.366 |
| seq.25260.62 | Uncharacterized protein C15orf17 | 0.396 | 0.900 |
| seq.25261.42 | Ankyrin repeat and SAM domain-containing protein 3 | 0.881 | 0.858 |
| seq.25262.6 | Centromere/kinetochore protein zw10 homolog | 0.474 | 0.208 |
| seq.25263.4 | Rho GTPase-activating protein 24 | 0.233 | 0.000 |
| seq.25264.102 | SHIP | 0.272 | 0.208 |
| seq.25265.8 | Formin-binding protein 1 | 0.291 | 0.423 |
| seq.25266.14 | Oxysterol-binding protein-related protein 1 | 0.554 | 0.004 |
| seq.25267.10 | Heat shock 70 kDa protein 12A | 0.081 | 0.151 |
| seq.25269.10 | Nardilysin | 0.848 | 0.716 |
| seq.25270.3 | Probable transcription factor PML | 0.606 | 0.880 |
| seq.25272.17 | Cadherin-23 | 0.307 | 0.576 |
| seq.25273.80 | Caldesmon | 0.002 | 0.000 |
| seq.25274.2 | Ankyrin repeat domain-containing protein 2 | 0.000 | 0.004 |
| seq.25275.30 | Histone deacetylase 4 | 0.660 | 0.976 |
| seq.25276.32 | Rab-like protein 6 | 0.170 | 0.043 |
| seq.25277.51 | Pantothenate kinase 1 | 0.442 | 0.154 |
| seq.25278.41 | Rho GTPase-activating protein 6 | 0.000 | 0.000 |
| seq.25279.44 | Nuclear valosin-containing protein-like | 0.822 | 0.797 |
| seq.25280.2 | LIM domain-containing protein 1 | 0.045 | 0.000 |
| seq.25281.50 | Dipeptidyl peptidase 8 | 0.861 | 0.866 |
| seq.25282.6 | Antizyme inhibitor 1 | 0.887 | 0.600 |
| seq.25283.2 | Catenin alpha-3 | 0.879 | 0.796 |
| seq.25284.47 | Ubiquitin-like modifier-activating enzyme 6 | 0.000 | 0.000 |
| seq.25285.14 | Kelch repeat and BTB domain-containing protein 11 | 0.546 | 0.571 |
| seq.25286.33 | Rab GTPase-activating protein 1-like | 0.197 | 0.007 |
| seq.25287.7 | Eukaryotic translation initiation factor 4 gamma 1 | 0.046 | 0.000 |
| seq.25288.16 | Dimethylaniline monooxygenase [N-oxide-forming] 3 | 0.379 | 0.606 |
| seq.25291.27 | Pleckstrin homology domain-containing family M member 2 | 0.399 | 0.506 |
| seq.25292.4 | Carboxylesterase 3 | 0.003 | 0.000 |
| seq.25292.6 | Carboxylesterase 3 | 0.000 | 0.381 |
| seq.25296.3 | Angiomotin | 0.624 | 0.929 |
| seq.25297.11 | SH3 and PX domain-containing protein 2B | 0.437 | 0.163 |
| seq.25298.53 | Lysine-specific demethylase 4C | 0.000 | 0.063 |
| seq.25299.11 | Nuclear receptor coactivator 7 | 0.112 | 0.118 |
| seq.25300.39 | Mucosa-associated lymphoid tissue lymphoma translocation protein 1 | 0.197 | 0.912 |
| seq.25301.48 | Dual specificity tyrosine-phosphorylation-regulated kinase 1A | 0.782 | 0.943 |
| seq.25306.51 | DAXX | 0.608 | 0.809 |
| seq.25307.8 | WD repeat-containing protein 18 | 0.198 | 0.398 |
| seq.25308.8 | LIM domain kinase 1 | 0.109 | 0.715 |
| seq.25409.5 | Coiled-coil domain-containing protein 140 | 0.900 | 0.578 |
| seq.25412.53 | FXYD domain-containing ion transport regulator 6 | 0.626 | 0.256 |
| seq.25413.80 | G antigen 2 | 0.436 | 0.580 |
| seq.25414.11 | Late cornified envelope protein 3B | 0.902 | 0.793 |
| seq.25416.5 | Periaxin | 0.887 | 0.804 |
| seq.25419.59 | Sperm protein associated with the nucleus on the X chromosome N4 | 0.424 | 0.512 |
| seq.25422.58 | Ribonuclease P protein subunit p40 | 0.851 | 0.332 |
| seq.25424.234 | Synaptotagmin-12 | 0.656 | 0.621 |
| seq.25428.103 | Interferon-related developmental regulator 1 | 0.231 | 0.200 |
| seq.25433.25 | Putative methyltransferase NSUN6 | 0.927 | 0.917 |
| seq.25436.44 | Saccharopine dehydrogenase-like oxidoreductase | 0.965 | 0.469 |
| seq.25437.18 | Ankyrin repeat and MYND domain-containing protein 2 | 0.793 | 0.483 |
| seq.25438.288 | 5'-nucleotidase domain-containing protein 1 | 0.525 | 0.744 |
| seq.25444.29 | Type I inositol 1,4,5-trisphosphate 5-phosphatase | 0.050 | 0.000 |
| seq.25446.34 | Protein DDI1 homolog 2 | 0.421 | 0.011 |
| seq.25451.39 | Transcriptional coactivator YAP1 | 0.622 | 0.822 |
| seq.25452.16 | Myb/SANT-like DNA-binding domain-containing protein 2 | 0.981 | 0.364 |
| seq.25453.57 | Ropporin-1B | 0.948 | 0.763 |
| seq.25454.38 | Protein arginine N-methyltransferase 2 | 0.450 | 0.464 |
| seq.25456.73 | 26S protease regulatory subunit 8 | 0.385 | 0.000 |
| seq.25459.3 | O-acetyl-ADP-ribose deacetylase MACROD2 | 0.440 | 0.777 |
| seq.25460.36 | Interferon regulatory factor 5 | 0.449 | 0.386 |
| seq.25461.3 | S-arrestin | 0.799 | 0.560 |
| seq.25463.3 | cAMP-dependent protein kinase type II-beta regulatory subunit | 0.562 | 0.410 |
| seq.25464.1 | Cytochrome P450 2C19 | 0.558 | 0.485 |
| seq.25465.42 | Muscular LMNA-interacting protein | 0.857 | 0.819 |
| seq.25466.84 | Kynurenine 3-monooxygenase | 0.869 | 0.275 |
| seq.25468.5 | SOX-9 | 0.459 | 0.782 |
| seq.25472.12 | Brain-enriched guanylate kinase-associated protein | 0.492 | 0.813 |
| seq.25473.62 | Protein farnesyltransferase/geranylgeranyltransferase type-1 subunit alpha | 0.621 | 0.797 |
| seq.25479.8 | Leucine-rich repeat flightless-interacting protein 2 | 0.000 | 0.000 |
| seq.25480.21 | Ubiquitin-associated domain-containing protein 1 | 0.382 | 0.216 |
| seq.25481.66 | Ataxin-10 | 0.222 | 0.185 |
| seq.25482.5 | Nuclear factor 1 A-type | 0.535 | 0.016 |
| seq.25484.120 | AT-rich interactive domain-containing protein 3C | 0.590 | 0.000 |
| seq.25486.38 | 39S ribosomal protein L38, mitochondrial | 0.705 | 0.509 |
| seq.25488.6 | Pleckstrin homology domain-containing family O member 2 | 0.513 | 0.000 |
| seq.25491.54 | Endoplasmic reticulum junction formation protein lunapark | 0.953 | 0.514 |
| seq.25492.7 | Target of Myb protein 1 | 0.867 | 0.462 |
| seq.25493.11 | Protein FAM221B | 0.444 | 0.000 |
| seq.25497.35 | SEC14-like protein 4 | 0.538 | 0.340 |
| seq.25499.37 | Phospholipase DDHD2 | 0.023 | 0.522 |
| seq.25500.6 | Rab11 family-interacting protein 3 | 0.411 | 0.000 |
| seq.25501.128 | Cytokine-dependent hematopoietic cell linker | 0.761 | 0.507 |
| seq.2558.51 | Beta-endorphin | 0.864 | 0.402 |
| seq.2567.5 | Complement factor I | 0.180 | 0.303 |
| seq.2570.72 | Insulin-like growth factor-binding protein 2 | 0.837 | 0.646 |
| seq.2571.12 | Insulin-like growth factor-binding protein 3 | 0.771 | 0.609 |
| seq.2573.20 | Interleukin-6 | 0.861 | 0.146 |
| seq.2575.5 | Leptin | 0.787 | 0.503 |
| seq.2578.67 | C-C motif chemokine 2 | 0.922 | 0.000 |
| seq.2579.17 | Matrix metalloproteinase-9 | 0.620 | 0.477 |
| seq.2580.83 | Myeloperoxidase | 0.642 | 0.704 |
| seq.2585.2 | Prolactin | 0.263 | 0.153 |
| seq.25879.6 | Histone acetyltransferase KAT5 | 0.000 | 0.000 |
| seq.25880.14 | 5-Lipoxygenase | 0.811 | 0.440 |
| seq.25886.11 | ATP-binding cassette sub-family D member 4 | 0.800 | 0.637 |
| seq.25889.108 | Absent in melanoma 2 | 0.841 | 0.445 |
| seq.25895.29 | Caspase-5 | 0.375 | 0.677 |
| seq.25898.4 | Chitinase domain-containing protein 1 | 0.827 | 0.949 |
| seq.2590.69 | Inactive tyrosine-protein kinase transmembrane receptor ROR1 | 0.459 | 0.312 |
| seq.25901.3 | CREB-regulated transcription coactivator 3 | 0.969 | 0.408 |
| seq.25902.27 | Glutamate dehydrogenase 1, mitochondrial | 0.398 | 0.765 |
| seq.25904.17 | Dedicator of cytokinesis protein 2 | 0.366 | 0.374 |
| seq.25906.5 | Elongation factor 1-alpha 1 | 0.944 | 0.452 |
| seq.25907.47 | Echinoderm microtubule-associated protein-like 2 | 0.907 | 0.545 |
| seq.25910.1 | IgGFc-binding protein | 0.192 | 0.192 |
| seq.25912.131 | FK506 binding protein 1A | 0.876 | 0.864 |
| seq.25913.17 | Ferritin heavy chain | 0.887 | 0.354 |
| seq.25917.12 | Beta-hexosaminidase alpha chain | 0.915 | 0.242 |
| seq.25918.60 | HLA class I histocompatibility antigen, alpha chain E | 0.000 | 0.421 |
| seq.25921.3 | Inhibin a subunit | 0.668 | 0.000 |
| seq.25922.7 | Low-density lipoprotein receptor-related protein 5 | 0.840 | 0.365 |
| seq.25926.29 | LIM domain-binding protein 1 | 0.944 | 0.170 |
| seq.25938.13 | Neurogenin-3 | 0.750 | 0.837 |
| seq.25941.4 | 2'-5'-oligoadenylate synthetase like protein | 0.531 | 0.174 |
| seq.25947.116 | Phosphatidylinositol 3-kinase catalytic subunit type 3 | 0.636 | 0.912 |
| seq.25948.18 | Serine/threonine-protein kinase N2 | 0.938 | 0.930 |
| seq.25949.3 | 1-phosphatidylinositol 4,5-bisphosphate phosphodiesterase delta-3 | 0.945 | 0.468 |
| seq.25951.17 | Preferentially expressed antigen of melanoma | 0.758 | 0.474 |
| seq.25954.3 | Receptor-type tyrosine-protein phosphatase-like N | 0.973 | 0.975 |
| seq.25956.9 | Rap guanine nucleotide exchange factor 1 | 0.657 | 0.393 |
| seq.25957.5 | Sex-determining region Y protein | 0.401 | 0.359 |
| seq.25960.15 | Plastin-3 | 0.393 | 0.660 |
| seq.25962.51 | Serine/threonine-protein kinase WNK1 | 0.000 | 0.057 |
| seq.25963.2 | Exportin-5 | 0.516 | 0.546 |
| seq.25964.12 | Complement C1q tumor necrosis factor-related protein 4 | 0.694 | 0.408 |
| seq.25967.34 | Metalloproteinase inhibitor 1 | 0.489 | 0.356 |
| seq.2597.8 | Vascular endothelial growth factor A | 0.311 | 0.452 |
| seq.2598.9 | Tumor necrosis factor receptor superfamily member 9 | 0.694 | 0.092 |
| seq.2599.51 | Tumor necrosis factor ligand superfamily member 9 | 0.000 | 0.541 |
| seq.2602.2 | Angiopoietin-2 | 0.650 | 0.574 |
| seq.2603.61 | T-lymphocyte activation antigen CD80 | 0.498 | 0.503 |
| seq.2605.49 | Tumor necrosis factor receptor superfamily member 8 | 0.335 | 0.471 |
| seq.2607.54 | Cytokine receptor-like factor 1:Cardiotrophin-like cytokine factor 1 Complex | 0.933 | 0.366 |
| seq.2609.59 | Cystatin-C | 0.680 | 0.689 |
| seq.2611.72 | Tyrosine-protein kinase receptor TYRO3 | 0.749 | 0.341 |
| seq.2612.5 | Eukaryotic translation initiation factor 5 | 0.483 | 0.000 |
| seq.2614.28 | Ephrin-A4 | 0.684 | 0.266 |
| seq.2615.60 | Ephrin-A5 | 0.655 | 0.106 |
| seq.2616.23 | Receptor tyrosine-protein kinase erbB-2 | 0.690 | 0.693 |
| seq.2617.56 | Receptor tyrosine-protein kinase erbB-3 | 0.374 | 0.478 |
| seq.2618.10 | Receptor tyrosine-protein kinase erbB-4 | 0.849 | 0.370 |
| seq.2619.72 | Tumor-associated calcium signal transducer 2 | 0.762 | 0.326 |
| seq.2620.4 | Interleukin-6 receptor subunit beta | 0.555 | 0.276 |
| seq.2622.18 | Heme oxygenase 2 | 0.653 | 0.406 |
| seq.2625.53 | Hsp90alpha | 0.047 | 0.000 |
| seq.2630.12 | Interleukin-1 Receptor accessory protein | 0.907 | 0.699 |
| seq.2631.50 | Interleukin-10 receptor subunit beta | 0.374 | 0.748 |
| seq.2632.5 | Interleukin-12 receptor subunit beta-1 | 0.453 | 0.000 |
| seq.2633.52 | Interleukin-13 receptor subunit alpha-1 | 0.243 | 0.432 |
| seq.2634.2 | Cytokine receptor common subunit gamma | 0.533 | 0.021 |
| seq.2635.61 | Layilin | 0.648 | 0.415 |
| seq.2636.10 | Tumor necrosis factor receptor superfamily member 3 | 0.601 | 0.585 |
| seq.2637.77 | Macrophage mannose receptor 1 | 0.722 | 0.263 |
| seq.2638.12 | Macrophage colony-stimulating factor 1 receptor | 0.639 | 0.714 |
| seq.2640.3 | Macrophage-stimulating protein receptor | 0.672 | 0.416 |
| seq.2642.4 | Platelet-activating factor acetylhydrolase IB subunit beta | 0.511 | 0.250 |
| seq.2643.57 | Cadherin-3 | 0.391 | 0.670 |
| seq.2644.11 | Protein kinase C alpha type | 0.023 | 0.000 |
| seq.2645.54 | Protein kinase C zeta type | 0.737 | 0.680 |
| seq.2647.66 | Rab GDP dissociation inhibitor beta | 0.196 | 0.000 |
| seq.2649.77 | Intercellular adhesion molecule 3 | 0.381 | 0.347 |
| seq.2652.15 | Urokinase plasminogen activator surface receptor | 0.681 | 0.349 |
| seq.2654.19 | Tumor necrosis factor receptor superfamily member 1A | 0.739 | 0.421 |
| seq.2658.27 | NT-3 growth factor receptor | 0.617 | 0.384 |
| seq.2665.26 | Tumor necrosis factor receptor superfamily member 17 | 0.878 | 0.600 |
| seq.2666.53 | Decorin | 0.675 | 0.335 |
| seq.2668.70 | Calpain I | 0.000 | 0.000 |
| seq.2670.67 | Creatine kinase M-type | 0.702 | 0.588 |
| seq.2677.1 | Epidermal growth factor receptor | 0.656 | 0.324 |
| seq.2681.23 | Hepatocyte growth factor | 0.858 | 0.510 |
| seq.2682.68 | 60 kDa heat shock protein, mitochondrial | 0.338 | 0.000 |
| seq.2683.1 | Complement C3b, inactivated | 0.536 | 0.329 |
| seq.2685.21 | Insulin-like growth factor-binding protein 5 | 0.550 | 0.260 |
| seq.2686.67 | Insulin-like growth factor-binding protein 6 | 0.659 | 0.364 |
| seq.2687.2 | Melanoma-derived growth regulatory protein | 0.695 | 0.152 |
| seq.2692.74 | Phospholipase A2, membrane associated | 0.722 | 0.576 |
| seq.2693.20 | Oncostatin-M | 0.543 | 0.569 |
| seq.2695.25 | Platelet endothelial cell adhesion molecule | 0.404 | 0.367 |
| seq.2696.87 | Persephin | 0.741 | 0.615 |
| seq.2697.7 | Platelet factor 4 | 0.670 | 0.324 |
| seq.2700.56 | Vitamin K-dependent protein S | 0.588 | 0.754 |
| seq.2704.74 | Tumor necrosis factor receptor superfamily member 13B | 0.714 | 0.841 |
| seq.2705.5 | C-C motif chemokine 25 | 0.648 | 0.619 |
| seq.2706.69 | Thyroxine-binding globulin | 0.576 | 0.524 |
| seq.2708.54 | Tumor necrosis factor ligand superfamily member 18 | 0.464 | 0.307 |
| seq.2711.6 | Ciliary neurotrophic factor receptor subunit alpha | 0.356 | 0.451 |
| seq.2714.78 | Endothelial monocyte-activating polypeptide 2 | 0.337 | 0.310 |
| seq.2715.25 | Erythropoietin receptor | 0.429 | 0.357 |
| seq.2719.3 | Granulocyte colony-stimulating factor receptor | 0.895 | 0.293 |
| seq.2723.9 | Interleukin-37 | 0.368 | 0.568 |
| seq.2728.62 | Tenascin | 0.413 | 0.457 |
| seq.2730.58 | MHC class I polypeptide-related sequence A | 0.909 | 0.822 |
| seq.2731.29 | NADPH--cytochrome P450 reductase | 0.224 | 0.711 |
| seq.2732.58 | Homeobox protein NANOG | 0.691 | 0.000 |
| seq.2734.49 | Natural cytotoxicity triggering receptor 2 | 0.683 | 0.132 |
| seq.2737.22 | Protein NOV homolog | 0.548 | 0.406 |
| seq.2741.22 | Sialic acid-binding Ig-like lectin 6 | 0.880 | 0.508 |
| seq.2742.68 | Sialic acid-binding Ig-like lectin 7 | 0.714 | 0.325 |
| seq.2743.5 | Sonic hedgehog protein | 0.418 | 0.264 |
| seq.2744.57 | Immunoglobulin G | 0.361 | 0.000 |
| seq.2746.56 | Cytokine receptor-like factor 2 | 0.666 | 0.391 |
| seq.2747.3 | NKG2D ligand 3 | 0.575 | 0.439 |
| seq.2748.3 | Activin A | 0.659 | 0.316 |
| seq.2750.3 | Apolipoprotein A-I | 0.549 | 0.509 |
| seq.2751.16 | Azurocidin | 0.514 | 0.175 |
| seq.2752.62 | Growth/differentiation factor 5 | 0.452 | 0.246 |
| seq.2753.2 | Complement C1q subcomponent | 0.309 | 0.492 |
| seq.2754.50 | Complement C3 | 0.305 | 0.270 |
| seq.2755.8 | C3a anaphylatoxin des Arginine | 0.227 | 0.272 |
| seq.2760.2 | Protein FAM107A | 0.165 | 0.000 |
| seq.2761.49 | Fibroblast growth factor 18 | 0.481 | 0.102 |
| seq.2762.30 | Fibroblast growth factor 19 | 0.306 | 0.302 |
| seq.2763.66 | Fibroblast growth factor 20 | 0.111 | 0.589 |
| seq.2765.4 | Growth/differentiation factor 11/8 | 0.717 | 0.457 |
| seq.2768.56 | Hemopexin | 0.942 | 0.932 |
| seq.2770.51 | C-C motif chemokine 1 | 0.664 | 0.563 |
| seq.2771.35 | Insulin-like growth factor-binding protein 1 | 0.528 | 0.576 |
| seq.2773.50 | Interleukin-10 | 0.855 | 0.342 |
| seq.2774.10 | Interleukin-16 | 0.691 | 0.495 |
| seq.2775.54 | Interleukin-17F | 0.793 | 0.765 |
| seq.2778.10 | Interleukin-22 | 0.673 | 0.744 |
| seq.2780.35 | Lactotransferrin | 0.665 | 0.526 |
| seq.2781.63 | C-C motif chemokine 4-like | 0.702 | 0.853 |
| seq.2783.18 | C-C motif chemokine 3-like 1 | 0.538 | 0.554 |
| seq.2785.15 | C-C motif chemokine 8 | 0.369 | 0.000 |
| seq.2788.55 | Stromelysin-1 | 0.302 | 0.022 |
| seq.2789.26 | Matrilysin | 0.438 | 0.587 |
| seq.2790.54 | Neutrophil-activating peptide 2 | 0.722 | 0.184 |
| seq.2794.60 | Superoxide dismutase [Cu-Zn] | 0.521 | 0.321 |
| seq.2796.62 | Fibrinogen | 0.000 | 0.088 |
| seq.2797.56 | Apolipoprotein B | 0.594 | 0.192 |
| seq.2805.6 | Angiotensin-converting enzyme 2 | 0.972 | 0.447 |
| seq.2806.49 | Activin receptor type-1B | 0.691 | 0.484 |
| seq.2809.25 | A disintegrin and metalloproteinase with thrombospondin motifs 4 | 0.832 | 0.208 |
| seq.2811.27 | Angiopoietin-1 | 0.773 | 0.427 |
| seq.2813.11 | Agouti-related protein | 0.649 | 0.419 |
| seq.2816.50 | Basal Cell Adhesion Molecule | 0.647 | 0.414 |
| seq.2819.23 | Cadherin-5 | 0.714 | 0.524 |
| seq.2822.56 | CD97 antigen | 0.644 | 0.562 |
| seq.2823.7 | COMM domain-containing protein 7 | 0.380 | 0.000 |
| seq.2826.53 | Ectodysplasin-A, secreted form | 0.815 | 0.640 |
| seq.2827.23 | Fractalkine | 0.542 | 0.640 |
| seq.2828.82 | Kunitz-type protease inhibitor 1 | 0.792 | 0.555 |
| seq.2829.19 | Interleukin-27 | 0.891 | 0.538 |
| seq.2831.29 | Kallikrein-11 | 0.820 | 0.538 |
| seq.2833.20 | Kallikrein-4 | 0.801 | 0.142 |
| seq.2834.54 | Kallikrein-8 | 0.804 | 0.283 |
| seq.2835.1 | X-ray repair cross-complementing protein 6 | 0.531 | 0.567 |
| seq.2836.68 | Neutrophil gelatinase-associated lipocalin | 0.627 | 0.390 |
| seq.2837.3 | Hepatocyte growth factor receptor | 0.788 | 0.251 |
| seq.2838.53 | Matrix metalloproteinase-17 | 0.911 | 0.419 |
| seq.2839.2 | Tumor necrosis factor ligand superfamily member 4 | 0.975 | 0.679 |
| seq.2841.13 | Secreted frizzled-related protein 3 | 0.699 | 0.477 |
| seq.2843.13 | Kunitz-type protease inhibitor 2 | 0.754 | 0.503 |
| seq.2844.53 | Tyrosine-protein kinase receptor Tie-1, soluble | 0.674 | 0.560 |
| seq.2846.24 | Ubiquitin+1, truncated mutation for UbB | 0.291 | 0.084 |
| seq.2848.2 | Wnt inhibitory factor 1 | 0.605 | 0.576 |
| seq.2849.49 | Allograft inflammatory factor 1 | 0.622 | 0.109 |
| seq.2851.63 | C5a anaphylatoxin | 0.629 | 0.385 |
| seq.2853.68 | Serine/threonine-protein kinase Chk1 | 0.784 | 0.243 |
| seq.2855.49 | Mitogen-activated protein kinase 3 | 0.000 | 0.000 |
| seq.2857.70 | Glucocorticoid receptor | 0.961 | 0.365 |
| seq.2858.29 | Histone acetyltransferase type B catalytic subunit | 0.941 | 0.626 |
| seq.2859.69 | Histone deacetylase 8 | 0.132 | 0.218 |
| seq.2860.19 | Importin subunit alpha-1 | 0.725 | 0.561 |
| seq.2864.2 | Dual specificity mitogen-activated protein kinase kinase 1 | 0.331 | 0.000 |
| seq.2867.52 | RAC-alpha serine/threonine-protein kinase | 0.000 | 0.000 |
| seq.2869.68 | Protein kinase C delta type | 0.199 | 0.000 |
| seq.2870.29 | Ras-related C3 botulinum toxin substrate 1 | 0.000 | 0.000 |
| seq.2871.73 | DNA repair protein RAD51 homolog 1 | 0.789 | 0.542 |
| seq.2875.15 | TATA-box-binding protein | 0.717 | 0.558 |
| seq.2876.74 | DNA topoisomerase 1 | 0.457 | 0.727 |
| seq.2877.3 | SUMO-conjugating enzyme UBC9 | 0.065 | 0.061 |
| seq.2878.66 | Tyrosine-protein kinase Yes | 0.920 | 0.246 |
| seq.2879.9 | Alpha-1-antichymotrypsin | 0.398 | 0.059 |
| seq.2888.49 | Complement component C7 | 0.055 | 0.056 |
| seq.2889.37 | Cardiotrophin-1 | 0.140 | 0.511 |
| seq.2890.59 | C-C motif chemokine 28 | 0.797 | 0.561 |
| seq.2891.1 | B-cell receptor CD22 | 0.416 | 0.452 |
| seq.2900.53 | C-C motif chemokine 14 | 0.783 | 0.434 |
| seq.2906.55 | Interleukin-4 | 0.610 | 0.196 |
| seq.2911.27 | Midkine | 0.192 | 0.852 |
| seq.2913.1 | C-C motif chemokine 23 | 0.774 | 0.621 |
| seq.2915.6 | Proliferating cell nuclear antigen | 0.804 | 0.598 |
| seq.2917.3 | Tumor necrosis factor ligand superfamily member 11 | 0.695 | 0.796 |
| seq.2925.9 | Plasminogen activator inhibitor 1 | 0.832 | 0.525 |
| seq.2937.10 | Apolipoprotein E (isoform E3) | 0.655 | 0.551 |
| seq.2938.55 | Apolipoprotein E (isoform E4) | 0.587 | 0.471 |
| seq.2939.10 | Artemin | 0.762 | 0.632 |
| seq.2942.50 | Cytochrome c | 0.788 | 0.772 |
| seq.2943.5 | Cytochrome P450 3A4 | 0.532 | 0.465 |
| seq.2944.66 | Neuroblastoma suppressor of tumorigenicity 1 | 0.464 | 0.097 |
| seq.2946.52 | Complement factor D | 0.593 | 0.209 |
| seq.2948.58 | Growth hormone receptor | 0.839 | 0.676 |
| seq.2949.6 | Group 10 secretory phospholipase A2 | 0.540 | 0.160 |
| seq.2950.57 | Insulin-like growth factor-binding protein 4 | 0.454 | 0.459 |
| seq.2952.75 | Insulin-like growth factor I | 0.843 | 0.728 |
| seq.2953.31 | Luteinizing hormone | 0.718 | 0.800 |
| seq.2954.56 | Neutrophil collagenase | 0.854 | 0.791 |
| seq.2960.66 | Properdin | 0.696 | 0.301 |
| seq.2961.1 | Vitamin K-dependent protein C | 0.578 | 0.317 |
| seq.2962.50 | Parathyroid hormone-related protein | 0.624 | 0.685 |
| seq.2966.65 | Stem cell growth factor-beta | 0.628 | 0.752 |
| seq.2967.8 | Vascular cell adhesion protein 1 | 0.720 | 0.637 |
| seq.2968.61 | Tumor necrosis factor ligand superfamily member 15 | 0.666 | 0.319 |
| seq.2969.11 | Serine/threonine-protein kinase receptor R3 | 0.080 | 0.686 |
| seq.2970.60 | Amphiregulin | 0.553 | 0.120 |
| seq.2972.57 | Bone morphogenetic protein 7 | 0.588 | 0.535 |
| seq.2973.15 | Platelet glycoprotein 4 | 0.738 | 0.771 |
| seq.2974.61 | Contactin-1 | 0.765 | 0.186 |
| seq.2975.19 | Connective tissue growth factor | 0.945 | 0.914 |
| seq.2976.58 | Desmoglein-1 | 0.617 | 0.562 |
| seq.2977.7 | Tumor necrosis factor receptor superfamily member EDAR | 0.656 | 0.186 |
| seq.2979.8 | C-X-C motif chemokine 5 | 0.575 | 0.145 |
| seq.2981.9 | Endothelial cell-selective adhesion molecule | 0.808 | 0.507 |
| seq.2982.82 | Galectin-4 | 0.758 | 0.631 |
| seq.2985.35 | Growth-regulated alpha protein | 0.853 | 0.839 |
| seq.2986.49 | Gro-gamma | 0.670 | 0.459 |
| seq.2987.37 | Histone H1.2 | 0.357 | 0.512 |
| seq.2989.17 | Interferon gamma | 0.491 | 0.229 |
| seq.2991.9 | Interleukin-1 receptor type 1 | 0.303 | 0.421 |
| seq.2992.59 | Interleukin-17 receptor A | 0.725 | 0.738 |
| seq.2993.1 | Interleukin-18 receptor accessory protein | 0.771 | 0.438 |
| seq.2994.71 | Interleukin-1 receptor-like 2 | 0.538 | 0.387 |
| seq.2997.8 | Junctional adhesion molecule B | 0.765 | 0.694 |
| seq.2998.53 | Junctional adhesion molecule C | 0.393 | 0.128 |
| seq.2999.6 | Limbic system-associated membrane protein | 0.713 | 0.259 |
| seq.3000.66 | Mannose-binding protein C | 0.873 | 0.856 |
| seq.3003.29 | Natural cytotoxicity triggering receptor 3 | 0.494 | 0.402 |
| seq.3004.67 | Programmed cell death 1 ligand 2 | 0.596 | 0.461 |
| seq.3005.5 | Tyrosine-protein phosphatase non-receptor type 1 | 0.081 | 0.000 |
| seq.3007.7 | Sialic acid-binding Ig-like lectin 9 | 0.987 | 0.989 |
| seq.3009.3 | Transforming growth factor beta receptor type 3 | 0.662 | 0.318 |
| seq.3010.53 | Thymic stromal lymphopoietin | 0.800 | 0.351 |
| seq.3022.4 | Cytotoxic T-lymphocyte protein 4 | 0.958 | 0.860 |
| seq.3024.18 | Alpha-2-antiplasmin | 0.456 | 0.570 |
| seq.3025.50 | Fibroblast growth factor 2 | 0.953 | 0.975 |
| seq.3026.5 | Calpastatin | 0.959 | 0.905 |
| seq.3028.36 | Ck-beta-8-1 | 0.614 | 0.489 |
| seq.3029.52 | CD209 antigen | 0.863 | 0.816 |
| seq.3030.3 | C-type lectin domain family 4 member M | 0.221 | 0.154 |
| seq.3032.11 | Follicle stimulating hormone | 0.760 | 0.722 |
| seq.3033.57 | Galectin-2 | 0.925 | 0.757 |
| seq.3034.1 | Glial fibrillary acidic protein | 0.107 | 0.953 |
| seq.3035.80 | Interleukin-19 | 0.832 | 0.334 |
| seq.3037.62 | Interleukin-1 beta | 0.748 | 0.427 |
| seq.3038.9 | C-X-C motif chemokine 11 | 0.768 | 0.710 |
| seq.3040.59 | C-C motif chemokine 3 | 0.768 | 0.776 |
| seq.3041.55 | C-type mannose receptor 2 | 0.588 | 0.441 |
| seq.3042.7 | Myoglobin | 0.684 | 0.409 |
| seq.3043.49 | SPARC | 0.752 | 0.558 |
| seq.3044.3 | C-C motif chemokine 18 | 0.637 | 0.633 |
| seq.3045.72 | Pleiotrophin | 0.388 | 0.603 |
| seq.3046.31 | Resistin | 0.541 | 0.344 |
| seq.3049.61 | Trypsin-1 | 0.702 | 0.633 |
| seq.3050.7 | von Willebrand factor | 0.468 | 0.414 |
| seq.3052.8 | Tumor necrosis factor ligand superfamily member 6, soluble form | 0.439 | 0.383 |
| seq.3053.49 | Fms-related tyrosine kinase 3 ligand | 0.000 | 0.237 |
| seq.3054.3 | Haptoglobin | 0.823 | 0.775 |
| seq.3055.54 | Interleukin-4 receptor subunit alpha | 0.231 | 0.353 |
| seq.3056.11 | NKG2-D type II integral membrane protein | 0.693 | 0.461 |
| seq.3057.55 | WNT1-inducible-signaling pathway protein 1 | 0.000 | 0.122 |
| seq.3059.50 | Tumor necrosis factor ligand superfamily member 13B | 0.563 | 0.628 |
| seq.3060.43 | Complement component C9 | 0.131 | 0.544 |
| seq.3061.61 | Cathepsin B | 0.772 | 0.525 |
| seq.3065.65 | Fibroblast growth factor 5 | 0.880 | 0.610 |
| seq.3066.12 | Galectin-3 | 0.840 | 0.487 |
| seq.3067.67 | Growth/differentiation factor 9 | 0.546 | 0.654 |
| seq.3069.52 | Immunoglobulin M | 0.862 | 0.941 |
| seq.3070.1 | Interleukin-2 | 0.607 | 0.574 |
| seq.3072.4 | Interleukin-13 | 0.867 | 0.180 |
| seq.3073.51 | Interleukin-18-binding protein | 0.637 | 0.392 |
| seq.3074.6 | Lipopolysaccharide-binding protein | 0.344 | 0.711 |
| seq.3077.66 | Coagulation factor Xa | 0.736 | 0.761 |
| seq.3078.1 | Placenta growth factor | 0.649 | 0.964 |
| seq.3079.62 | Retinoic acid receptor responder protein 2 | 0.555 | 0.746 |
| seq.3081.70 | NKG2D ligand 1 | 0.688 | 0.456 |
| seq.3082.9 | NKG2D ligand 2 | 0.645 | 0.217 |
| seq.3083.71 | Tumor necrosis factor receptor superfamily member 27 | 0.748 | 0.428 |
| seq.3091.70 | Aurora kinase A | 0.506 | 0.922 |
| seq.3115.64 | Mitogen-activated protein kinase 1 | 0.053 | 0.000 |
| seq.3122.6 | Diablo homolog, mitochondrial | 0.682 | 0.786 |
| seq.3132.1 | Vascular endothelial growth factor C | 0.633 | 0.477 |
| seq.3143.3 | T-cell surface glycoprotein CD4 | 0.658 | 0.575 |
| seq.3148.49 | Gro-beta | 0.561 | 0.349 |
| seq.3151.6 | Interleukin-2 receptor subunit alpha | 0.707 | 0.822 |
| seq.3152.57 | Tumor necrosis factor receptor superfamily member 1B | 0.770 | 0.450 |
| seq.3166.92 | Myeloid cell surface antigen CD33 | 0.848 | 0.715 |
| seq.3168.8 | A disintegrin and metalloproteinase with thrombospondin motifs 5 | 0.516 | 0.639 |
| seq.3169.70 | Alpha-L-iduronidase | 0.784 | 0.883 |
| seq.3170.6 | Methionine aminopeptidase 2 | 0.048 | 0.000 |
| seq.3171.57 | Amyloid beta A4 protein | 0.713 | 0.501 |
| seq.3172.28 | Arylsulfatase B | 0.196 | 0.706 |
| seq.3173.49 | N-acylethanolamine-hydrolyzing acid amidase | 0.461 | 0.457 |
| seq.3174.2 | A disintegrin and metalloproteinase with thrombospondin motifs 1 | 0.745 | 0.523 |
| seq.3175.51 | A disintegrin and metalloproteinase with thrombospondin motifs 13 | 0.642 | 0.619 |
| seq.3177.49 | Carbonic anhydrase 4 | 0.654 | 0.578 |
| seq.3178.5 | Dipeptidyl peptidase 1 | 0.453 | 0.796 |
| seq.3179.51 | Lysosomal protective protein | 0.712 | 0.754 |
| seq.3181.50 | Cathepsin S | 0.590 | 0.208 |
| seq.3182.38 | Ectonucleoside triphosphate diphosphohydrolase 1 | 0.505 | 0.952 |
| seq.3184.25 | Coagulation factor VII | 0.605 | 0.401 |
| seq.3186.2 | Complement C2 | 0.599 | 0.577 |
| seq.3187.52 | Cysteine-rich secretory protein 3 | 0.789 | 0.325 |
| seq.3189.61 | Enteropeptidase | 0.660 | 0.000 |
| seq.3191.50 | WAP, kazal, immunoglobulin, kunitz and NTR domain-containing protein 1 | 0.533 | 0.080 |
| seq.3192.3 | Cytosolic non-specific dipeptidase | 0.355 | 0.489 |
| seq.3194.36 | Platelet glycoprotein VI | 0.168 | 0.000 |
| seq.3195.50 | Granulysin | 0.862 | 0.773 |
| seq.3196.6 | Hyaluronan and proteoglycan link protein 1 | 0.694 | 0.668 |
| seq.3197.70 | Insulin-degrading enzyme | 0.326 | 0.175 |
| seq.3198.4 | Iduronate 2-sulfatase | 0.367 | 0.388 |
| seq.3199.54 | Kallikrein-12 | 0.483 | 0.527 |
| seq.3200.49 | Kallikrein-13 | 0.712 | 0.332 |
| seq.3201.49 | Kallikrein-5 | 0.784 | 0.265 |
| seq.3202.28 | Kremen protein 2 | 0.386 | 0.311 |
| seq.3204.2 | Leukotriene A-4 hydrolase | 0.336 | 0.539 |
| seq.3206.4 | Lymphatic vessel endothelial hyaluronic acid receptor 1 | 0.645 | 0.569 |
| seq.3208.2 | Matrilin-3 | 0.266 | 0.388 |
| seq.3209.69 | Matrix extracellular phosphoglycoprotein | 0.824 | 0.327 |
| seq.3210.1 | Methionine aminopeptidase 1 | 0.050 | 0.000 |
| seq.3212.30 | Neutral ceramidase | 0.770 | 0.631 |
| seq.3213.65 | Nidogen-1 | 0.359 | 0.445 |
| seq.3214.3 | Neuropilin-1 | 0.458 | 0.000 |
| seq.3216.2 | Polymeric immunoglobulin receptor | 0.785 | 0.752 |
| seq.3217.74 | Glia-derived nexin | 0.795 | 0.427 |
| seq.3218.8 | Glutamate carboxypeptidase 2 | 0.393 | 0.545 |
| seq.3220.40 | Proto-oncogene tyrosine-protein kinase receptor Ret | 0.952 | 0.607 |
| seq.3221.54 | Secreted frizzled-related protein 1 | 0.688 | 0.696 |
| seq.3222.11 | Semaphorin-3A | 0.890 | 0.520 |
| seq.3232.28 | Tartrate-resistant acid phosphatase type 5 | 0.730 | 0.714 |
| seq.3234.23 | Coiled-coil domain-containing protein 80 | 0.604 | 0.215 |
| seq.3235.50 | WAP, Kazal, immunoglobulin, Kunitz and NTR domain-containing protein 2 | 0.624 | 0.309 |
| seq.3236.12 | Glycogen synthase kinase-3 beta | 0.085 | 0.000 |
| seq.3280.49 | Aggrecan core protein | 0.646 | 0.778 |
| seq.3283.21 | Transforming growth factor-beta-induced protein ig-h3 | 0.372 | 0.073 |
| seq.3284.75 | Biglycan | 0.410 | 0.526 |
| seq.3285.23 | Complement C1r subcomponent | 0.426 | 0.000 |
| seq.3289.19 | Carbonic anhydrase-related protein 10 | 0.636 | 0.407 |
| seq.3290.50 | CD109 antigen | 0.874 | 0.612 |
| seq.3291.30 | Low affinity immunoglobulin epsilon Fc receptor | 0.920 | 0.688 |
| seq.3292.75 | CD48 antigen | 0.687 | 0.342 |
| seq.3293.2 | CD5 antigen-like | 0.875 | 0.739 |
| seq.3294.55 | Cryptic protein | 0.741 | 0.511 |
| seq.3296.92 | Contactin-2 | 0.629 | 0.552 |
| seq.3298.52 | Contactin-4 | 0.798 | 0.158 |
| seq.3299.29 | Contactin-5 | 0.772 | 0.313 |
| seq.3302.58 | Cystatin-F | 0.820 | 0.490 |
| seq.3303.23 | Cystatin-M | 0.773 | 0.610 |
| seq.3305.6 | Delta-like protein 4 | 0.511 | 0.422 |
| seq.3309.2 | Low affinity immunoglobulin gamma Fc region receptor II-a | 0.970 | 0.955 |
| seq.3310.62 | Low affinity immunoglobulin gamma Fc region receptor II-b | 0.960 | 0.931 |
| seq.3311.27 | Low affinity immunoglobulin gamma Fc region receptor III-B | 0.832 | 0.923 |
| seq.3312.64 | High affinity immunoglobulin gamma Fc receptor I | 0.737 | 0.572 |
| seq.3313.21 | Ficolin-2 | 0.446 | 0.698 |
| seq.3314.74 | GDNF family receptor alpha-1 | 0.579 | 0.286 |
| seq.3315.15 | Glypican-2 | 0.901 | 0.734 |
| seq.3316.58 | Heparin cofactor 2 | 0.771 | 0.714 |
| seq.3317.33 | Serine protease HTRA2, mitochondrial | 0.410 | 0.000 |
| seq.3320.49 | Insulin-like growth factor-binding protein 7 | 0.725 | 0.386 |
| seq.3321.2 | Interleukin-24 | 0.687 | 0.143 |
| seq.3322.52 | Leucine-rich repeats and immunoglobulin-like domains protein 3 | 0.472 | 0.000 |
| seq.3323.37 | Low-density lipoprotein receptor-related protein 8 | 0.763 | 0.566 |
| seq.3324.51 | T-lymphocyte surface antigen Ly-9 | 0.819 | 0.704 |
| seq.3325.2 | Matrilin-2 | 0.595 | 0.310 |
| seq.3326.58 | Cell adhesion molecule 1 | 0.435 | 0.436 |
| seq.3327.27 | Netrin-4 | 0.575 | 0.527 |
| seq.3329.14 | Peptidoglycan recognition protein 1 | 0.926 | 0.911 |
| seq.3331.8 | RGM domain family member B | 0.671 | 0.055 |
| seq.3332.57 | Hemojuvelin | 0.640 | 0.020 |
| seq.3336.50 | Tissue factor pathway inhibitor | 0.773 | 0.652 |
| seq.3339.33 | Thrombospondin-2 | 0.672 | 0.552 |
| seq.3340.53 | Thrombospondin-4 | 0.610 | 0.512 |
| seq.3341.33 | Tyrosine-protein kinase ABL1 | 0.352 | 0.047 |
| seq.3342.76 | Abelson tyrosine-protein kinase 2 | 0.869 | 0.396 |
| seq.3343.1 | Aminoacylase-1 | 0.178 | 0.745 |
| seq.3344.60 | Antithrombin-III | 0.265 | 0.090 |
| seq.3346.72 | Aurora kinase B | 0.329 | 0.525 |
| seq.3347.9 | beta-adrenergic receptor kinase 1 | 0.000 | 0.000 |
| seq.3348.49 | Bone morphogenetic protein 1 | 0.335 | 0.527 |
| seq.3350.53 | Calcium/calmodulin-dependent protein kinase type II subunit alpha | 0.237 | 0.000 |
| seq.3351.1 | Calcium/calmodulin-dependent protein kinase type II subunit beta | 0.036 | 0.000 |
| seq.3352.80 | Carbonic anhydrase 6 | 0.879 | 0.775 |
| seq.3356.50 | Carbonic anhydrase 7 | 0.607 | 0.335 |
| seq.3357.67 | Cyclin-dependent kinase 2:Cyclin-A2 complex | 0.816 | 0.302 |
| seq.3358.51 | Cyclin-dependent kinase 5:Cyclin-dependent kinase 5 activator 1 complex | 0.543 | 0.536 |
| seq.3359.11 | Cyclin-dependent kinase 8:Cyclin-C complex | 0.775 | 0.160 |
| seq.3360.50 | Serine/threonine-protein kinase Chk2 | 0.962 | 0.809 |
| seq.3361.26 | C-type lectin domain family 4 member K | 0.203 | 0.090 |
| seq.3362.61 | Chordin-like protein 1 | 0.580 | 0.558 |
| seq.3363.31 | Tyrosine-protein kinase CSK | 0.000 | 0.000 |
| seq.3364.76 | Cathepsin L2 | 0.561 | 0.736 |
| seq.3365.7 | Dickkopf-related protein 4 | 0.764 | 0.505 |
| seq.3366.51 | Extracellular matrix protein 1 | 0.147 | 0.340 |
| seq.3367.8 | Fetuin-B | 0.863 | 0.673 |
| seq.3373.5 | Granzyme H | 0.631 | 0.733 |
| seq.3374.49 | Tyrosine-protein kinase HCK | 0.790 | 0.171 |
| seq.3376.49 | Interleukin-17 receptor D | 0.776 | 0.248 |
| seq.3378.49 | Kallikrein-7 | 0.414 | 0.102 |
| seq.3379.29 | Protein kinase C iota type | 0.213 | 0.015 |
| seq.3381.24 | Tyrosine-protein kinase Lyn, isoform B | 0.065 | 0.000 |
| seq.3387.1 | Serine/threonine-protein kinase PAK 3 | 0.711 | 0.548 |
| seq.3388.58 | Serine/threonine-protein kinase PAK 5 | 0.276 | 0.299 |
| seq.3389.7 | Plasma serine protease inhibitor | 0.578 | 0.530 |
| seq.3390.72 | PIK3CA/PIK3R1 | 0.083 | 0.000 |
| seq.3391.10 | Phosphatidylinositol 4,5-bisphosphate 3-kinase catalytic subunit gamma isoform | 0.633 | 0.302 |
| seq.3392.68 | Protein kinase B gamma | 0.000 | 0.000 |
| seq.3394.81 | Serine/threonine-protein kinase PLK1 | 0.371 | 0.042 |
| seq.3396.54 | Renin | 0.895 | 0.633 |
| seq.3397.7 | Tyrosine-protein phosphatase non-receptor type 11 | 0.350 | 0.056 |
| seq.3399.31 | Stabilin-2 | 0.212 | 0.388 |
| seq.3400.49 | Serine/threonine-protein kinase TBK1 | 0.863 | 0.499 |
| seq.3401.8 | Tyrosine-protein phosphatase non-receptor type 2 | 0.385 | 0.472 |
| seq.3403.1 | Tryptase beta-2 | 0.832 | 0.624 |
| seq.3404.51 | Tryptase gamma | 0.303 | 0.131 |
| seq.3405.6 | Ubiquitin-fold modifier-conjugating enzyme 1 | 0.000 | 0.000 |
| seq.3412.7 | Apoptosis regulator Bcl-2 | 0.592 | 0.250 |
| seq.3413.50 | Bcl-2-related protein A1 | 0.000 | 0.084 |
| seq.3414.40 | Cytoplasmic tyrosine-protein kinase BMX | 0.498 | 0.029 |
| seq.3415.61 | Bone sialoprotein 2 | 0.811 | 0.843 |
| seq.3416.2 | Tyrosine-protein kinase BTK | 0.043 | 0.000 |
| seq.3418.12 | Calcium/calmodulin-dependent protein kinase type 1D | 0.326 | 0.477 |
| seq.3419.49 | Calcium/calmodulin-dependent protein kinase type II subunit delta | 0.044 | 0.000 |
| seq.3420.21 | Carbonic anhydrase 13 | 0.061 | 0.000 |
| seq.3421.54 | Tumor necrosis factor ligand superfamily member 8 | 0.887 | 0.781 |
| seq.3422.4 | Cyclin-dependent kinase 1:G2/mitotic-specific cyclin-B1 complex | 0.496 | 0.103 |
| seq.3423.59 | Chymase | 0.310 | 0.294 |
| seq.3427.63 | Casein kinase II subunit alpha | 0.456 | 0.327 |
| seq.3431.54 | Ephrin type-A receptor 1 | 0.794 | 0.676 |
| seq.3432.21 | Ephrin type-A receptor 3 | 0.727 | 0.492 |
| seq.3434.34 | Fibronectin Fragment 3 | 0.233 | 0.203 |
| seq.3435.53 | Fibronectin Fragment 4 | 0.237 | 0.216 |
| seq.3437.80 | Receptor-type tyrosine-protein kinase FLT3 | 0.214 | 0.335 |
| seq.3438.10 | Follistatin-related protein 3 | 0.716 | 0.475 |
| seq.3440.7 | Granzyme A | 0.798 | 0.543 |
| seq.3441.64 | Glycogen synthase kinase-3 alpha | 0.000 | 0.000 |
| seq.3445.53 | Interleukin-15 receptor subunit alpha | 0.576 | 0.516 |
| seq.3446.7 | Interleukin-18 receptor 1 | 0.546 | 0.289 |
| seq.3447.64 | Interleukin-8 | 0.176 | 0.527 |
| seq.3448.13 | Insulin receptor | 0.571 | 0.374 |
| seq.3449.58 | Kallistatin | 0.472 | 0.708 |
| seq.3450.4 | Kallikrein-6 | 0.422 | 0.553 |
| seq.3452.17 | Tyrosine-protein kinase Lck | 0.656 | 0.254 |
| seq.3453.87 | Tyrosine-protein kinase Lyn | 0.000 | 0.000 |
| seq.3457.57 | Periostin | 0.703 | 0.496 |
| seq.3459.49 | Platelet-derived growth factor receptor beta | 0.953 | 0.897 |
| seq.3461.58 | Brevican core protein | 0.826 | 0.732 |
| seq.3466.8 | cAMP-dependent protein kinase catalytic subunit alpha | 0.140 | 0.000 |
| seq.3469.74 | Ribosomal protein S6 kinase alpha-3 | 0.124 | 0.025 |
| seq.3470.1 | E-selectin | 0.898 | 0.780 |
| seq.3471.49 | Serine/threonine-protein kinase 16 | 0.719 | 0.426 |
| seq.3472.40 | Baculoviral IAP repeat-containing protein 5 | 0.260 | 0.461 |
| seq.3473.78 | Thrombopoietin Receptor | 0.569 | 0.002 |
| seq.3474.19 | Thrombospondin-1 | 0.723 | 0.021 |
| seq.3477.63 | High affinity nerve growth factor receptor | 0.856 | 0.320 |
| seq.3479.71 | Trypsin-3 | 0.852 | 0.625 |
| seq.3480.7 | Dual specificity protein phosphatase 3 | 0.647 | 0.056 |
| seq.3481.87 | Xaa-Pro aminopeptidase 1 | 0.262 | 0.000 |
| seq.3484.60 | Angiotensinogen | 0.786 | 0.621 |
| seq.3485.28 | Beta-2-microglobulin | 0.721 | 0.815 |
| seq.3486.58 | Fibroblast growth factor 1 | 0.283 | 0.296 |
| seq.3487.32 | C-X-C motif chemokine 13 | 0.299 | 0.589 |
| seq.3488.64 | Catalase | 0.411 | 0.215 |
| seq.3489.9 | Ciliary neurotrophic factor | 0.328 | 0.000 |
| seq.3494.71 | Fibroblast growth factor 17 | 0.371 | 0.185 |
| seq.3495.15 | C-X-C motif chemokine 6 | 0.745 | 0.870 |
| seq.3497.13 | Interferon alpha-2 | 0.197 | 0.377 |
| seq.3498.53 | Interleukin-17A | 0.226 | 0.492 |
| seq.3499.77 | Interleukin-17B | 0.693 | 0.234 |
| seq.3503.4 | Integrin alpha-I: beta-1 complex | 0.299 | 0.135 |
| seq.3504.58 | Hepcidin | 0.757 | 0.579 |
| seq.3505.6 | Lymphotoxin alpha1:beta2 | 0.541 | 0.190 |
| seq.3506.49 | Lymphotoxin alpha2:beta1 | 0.809 | 0.404 |
| seq.3508.78 | C-C motif chemokine 22 | 0.424 | 0.600 |
| seq.3509.1 | C-C motif chemokine 15 | 0.740 | 0.581 |
| seq.3514.49 | Myeloblastin | 0.710 | 0.593 |
| seq.3516.60 | Stromal cell-derived factor 1 | 0.472 | 0.371 |
| seq.3518.54 | Carboxypeptidase B2 | 0.455 | 0.628 |
| seq.3519.3 | C-C motif chemokine 17 | 0.732 | 0.318 |
| seq.3520.58 | Transforming growth factor beta-3 | 0.963 | 0.164 |
| seq.3521.16 | Thyroid Stimulating Hormone | 0.572 | 0.752 |
| seq.3522.57 | Vasoactive Intestinal Peptide | 0.967 | 0.525 |
| seq.3534.14 | CD40 ligand | 0.674 | 0.457 |
| seq.3535.84 | Dickkopf-related protein 1 | 0.767 | 0.508 |
| seq.3538.26 | Aromatic-L-amino-acid decarboxylase | 0.444 | 0.268 |
| seq.3554.24 | Adiponectin | 0.774 | 0.741 |
| seq.3580.25 | Alpha-1-antitrypsin | 0.827 | 0.554 |
| seq.3581.53 | Alpha-2-HS-glycoprotein | 0.422 | 0.547 |
| seq.3583.54 | Arylsulfatase A | 0.713 | 0.591 |
| seq.3585.54 | Basigin | 0.331 | 0.417 |
| seq.3587.53 | Bone morphogenetic protein 10 | 0.819 | 0.285 |
| seq.3591.51 | Cadherin-6 | 0.645 | 0.312 |
| seq.3592.4 | Calcium/calmodulin-dependent protein kinase type 1 | 0.794 | 0.548 |
| seq.3593.72 | Caspase-3 | 0.069 | 0.000 |
| seq.3600.2 | Chitotriosidase-1 | 0.841 | 0.917 |
| seq.3601.54 | Neural cell adhesion molecule L1-like protein | 0.434 | 0.411 |
| seq.3603.60 | C-type lectin domain family 7 member A | 0.596 | 0.450 |
| seq.3605.77 | Mannan-binding lectin serine protease 1:Mannan-binding lectin serine protease 1 light chain | 0.171 | 0.250 |
| seq.3607.71 | Dickkopf-related protein 3 | 0.620 | 0.198 |
| seq.3611.70 | Endothelin-converting enzyme 1:Extracellular domain | 0.606 | 0.437 |
| seq.3613.62 | Ficolin-1 | 0.728 | 0.722 |
| seq.3616.3 | N-acetylglucosamine-6-sulfatase | 0.740 | 0.762 |
| seq.3617.80 | Hepatocyte growth factor activator | 0.972 | 0.967 |
| seq.3620.67 | Interleukin-22 receptor subunit alpha-1 | 0.661 | 0.373 |
| seq.3622.33 | Legumain | 0.208 | 0.476 |
| seq.3623.84 | Lymphocyte antigen 86 | 0.000 | 0.510 |
| seq.3624.3 | Serine protease 27 | 0.260 | 0.652 |
| seq.3627.71 | Membrane metallo-endopeptidase-like 1 | 0.605 | 0.841 |
| seq.3628.3 | Dual specificity mitogen-activated protein kinase kinase 2 | 0.752 | 0.808 |
| seq.3629.60 | Serine/threonine-protein kinase MRCK beta | 0.138 | 0.275 |
| seq.3630.27 | Cell adhesion molecule 3 | 0.571 | 0.142 |
| seq.3633.70 | Nidogen-2 | 0.387 | 0.288 |
| seq.3634.5 | Opioid-binding protein/cell adhesion molecule | 0.251 | 0.424 |
[truncated: 182,120 more chars]
